# Facile synthesis of 1-alkoxy-1*H*-benzo- and 7-azabenzotriazoles from peptide coupling agents, mechanistic studies, and synthetic applications

**DOI:** 10.3762/bjoc.10.200

**Published:** 2014-08-19

**Authors:** Mahesh K Lakshman, Manish K Singh, Mukesh Kumar, Raghu Ram Chamala, Vijayender R Yedulla, Domenick Wagner, Evan Leung, Lijia Yang, Asha Matin, Sadia Ahmad

**Affiliations:** 1 Department of Chemistry, The City College and The City University of New York, 160 Convent Avenue, New York, NY 10031, USA

**Keywords:** alkoxy, azabenzotriazole, benzotriazole, peptide-coupling, phosphonium

## Abstract

(1*H*-Benzo[*d*][1,2,3]triazol-1-yloxy)tris(dimethylamino)phosphonium hexafluorophosphate (BOP), 1*H*-benzo[*d*][1,2,3]triazol-1-yl 4-methylbenzenesulfonate (Bt-OTs), and 3*H*-[1,2,3]triazolo[4,5-*b*]pyridine-3-yl 4-methylbenzenesulfonate (At-OTs) are classically utilized in peptide synthesis for amide-bond formation. However, a previously undescribed reaction of these compounds with alcohols in the presence of a base, leads to 1-alkoxy-1*H*-benzo- (Bt-OR) and 7-azabenzotriazoles (At-OR). Although BOP undergoes reactions with alcohols to furnish 1-alkoxy-1*H*-benzotriazoles, Bt-OTs proved to be superior. Both, primary and secondary alcohols undergo reaction under generally mild reaction conditions. Correspondingly, 1-alkoxy-1*H*-7-azabenzotriazoles were synthesized from At-OTs. Mechanistically, there are three pathways by which these peptide-coupling agents can react with alcohols. From ^31^P{^1^H}, [^18^O]-labeling, and other chemical experiments, phosphonium and tosylate derivatives of alcohols seem to be intermediates. These then react with BtO^−^ and AtO^−^ produced in situ. In order to demonstrate broader utility, this novel reaction has been used to prepare a series of acyclic nucleoside-like compounds. Because BtO^−^ is a nucleofuge, several Bt-OCH_2_Ar substrates have been evaluated in nucleophilic substitution reactions. Finally, the possible formation of Pd π–allyl complexes by departure of BtO^−^ has been queried. Thus, alpha-allylation of three cyclic ketones was evaluated with 1-(cinnamyloxy)-1*H*-benzo[*d*][1,2,3]triazole, via in situ formation of pyrrolidine enamines and Pd catalysis.

## Introduction

Benzotriazole derivatives are of importance in diverse contexts. As examples, in medicinal chemistry substituted benzotriazoles have been evaluated as inhibitors of respiratory syncytial virus [1], halogenated benzotriazoles have been shown to inhibit helicase activity of hepatitis C [2], *N*-alkylbenzotriazoles were shown to be active and selective towards HCV NTPase/helicase [3]. Benzotriazoles also possess anti-amoebic properties, particularly against the human pathogen *Acanthamoeba* that can infect a variety of organs such as brain, eyes, skin, and lungs [4]. Triazole and benzotriazole derivatives have been evaluated as antitumor agents, with several showing high activities [5], and a benzotriazole derivative was shown to inhibit proliferation of hepatocarcinoma [6]. Several *N*-alkylbenzotriazoles show potent antimicrobial action [7] and others have been evaluated as aromatase inhibitors [8]. Benzotriazole derivatives have also been reported to be inhibitors of MAP kinases [9]. Although esters of BtOH are generally intermediates in amide synthesis, stable ones have recently been evaluated against the new coronavirus responsible for SARS, and several compounds were shown to be irreversible inhibitors of the viral proteinase 3CL^pro^ (also called M^pro^) [10]. Benzotriazoles with ether linkages on the phenyl ring have been reported to be promising entities in the treatment of glutamate mGluR2 receptor dysfunction-related diseases, such as neurological and psychological disorders [11].

Benzotriazole-derived compounds also have applications in materials chemistry. For example, 5-alkyl- and 5-alkanoylaminobenzotriazoles have been developed to prevent corrosion at metal surfaces, as metal deactivators, and to prevent degradation of lubricants and coatings [12–13]. Esters of benzotriazole and alkylbenzotriazoles have been reported as components in organic lubricating compositions and in turbine lubricants [14–15]. Of relevance to this work a single ether of hydroxybenzotriazole has been evaluated in lubricant compositions [16].

**Scheme 1:**
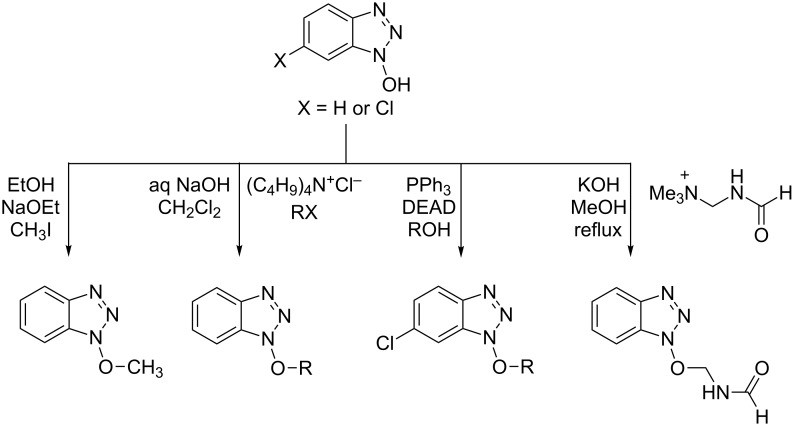
Examples of methods for the synthesis of 1-alkoxy-1*H*-benzo[*d*][1,2,3]triazoles.

New approaches to benzotriazole derivatives are therefore expected to have a broad-ranging impact. Among the various N-substituted benzotriazolyl derivatives, as compared to *N*-alkyl and *N*-acyl compounds, those with a C–O–N bond are less common. Typically the latter are synthesized by the alkylation of BtOH with alkyl halides [17–18], quaternary alkyl ammonium salts [19], or via a Mitsunobu reaction (Scheme 1) [20].

**Figure 1:**
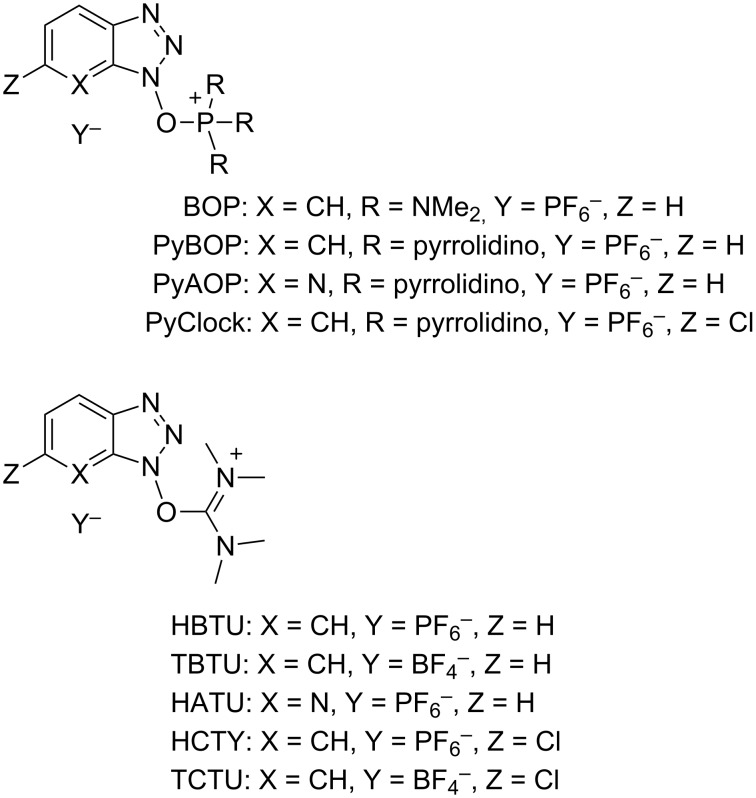
Examples of commercially available reagents for amidation reactions.

Herein, we report a facile approach to 1-alkoxy-1*H*-benzo- (Bt-OR) and 7-azabenzotriazoles (At-OR) by a previously unstudied reaction of benzotriazole-based peptide-coupling reagents with alcohols [21]. We also describe studies on the underlying mechanism and a preliminary disclosure of the potential synthetic applications of these products. Figure 1 shows examples of commercially available phosphonium (e.g., BOP, PyBOP, PyAOP, and PyClock) and iminium reagents (e.g., HBTU, TBTU, HATU, HCTU, and TCTU – uronium forms are shown) that are commonly used for amide-bond formation.

## Results and Discussion

### Synthesis of 1-alkoxy-1*H*-benzo- and 7-azabenzotriazoles (Bt-OR and At-OR)

**Scheme 2:**
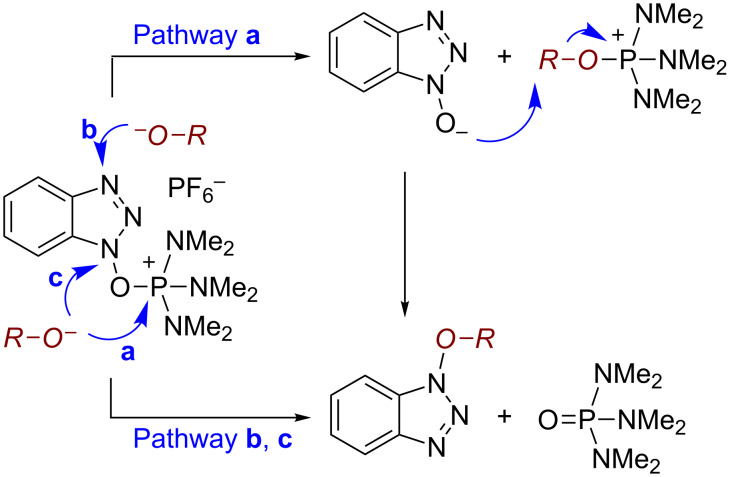
Three possible mechanisms for the reaction of BOP with oxygen nucleophiles.

We have previously described the use of (benzotriazol-1-yloxy)tris(dimethylamino)phosphonium hexafluorophosphate (BOP) for the dehydration of aldoximes to cyanides [22]. In that work, analysis of the reaction course by ^31^P{^1^H} NMR did not show the formation of a new phosphorus signal other than that for HMPA, which is a reaction byproduct. However, prior investigations by others [23–24] and by us [25–27] have demonstrated the formation of phosphonium ions by the reaction of BOP with the oxygen atoms in amide linkages of purines and related heterocycles. In fact, related to these observations we have demonstrated the isolation and synthetic utility of a nucleoside phosphonium salt [28]. Thus, to us the reaction of oximes with BOP was an intriguing result, leading us to query whether a benzotriazolyl intermediate, rather than a phosphonium ion, was formed en route to the cyanide. This line of reasoning would then suggest that 1-alkoxy-1*H*-benzotriazoles may indeed be obtainable from the reactions of alcohols with BOP, and that different reaction pathways may be operative depending upon the nature of the nucleophile (Scheme 2). However, the formation of 1-alkoxy-1*H*-benzotriazoles by such an approach appeared implausible on the basis of prior observations, where no reaction of BOP with the free hydroxy groups of nucleosides was observed [23,25].

Our recent work on a two-step one-pot etherification of purine nucleosides, quinazoline, and pyrimidines, had led some interesting preliminary observations [29]. Although BOP did not react with MeOH in the absence of a base, in the presence of Cs_2_CO_3_ rapid formation of HMPA was observed and 1-methoxy-1*H*-benzotriazole (1-methoxy-1*H*-benzo[*d*][1,2,3]triazole) was isolated [29]. This evidence clearly showed that alcohols are capable of reaction with BOP in the presence of a base. Thus, we first evaluated whether the reaction of BOP with alcohols was general and we elected to use DBU as base for cost considerations. Table 1 shows the results of this analysis.

**Table 1:** Reactions of alcohols with BOP and DBU.^a^

Entry	Alcohol	Product	Time (h at rt)	Compound: yield^b^

1	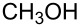	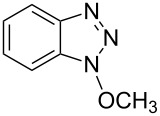	24 24	**1a**: 48% **1a**: 50%^c^ **1a**: 47%^d^
2	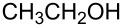	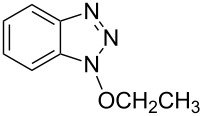	24	**1b**: 52%
3	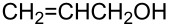	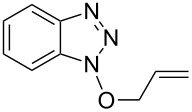	48	**1c**: 52%
4	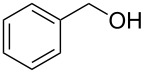	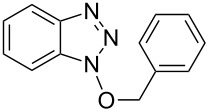	48	**1d**: 39%
5	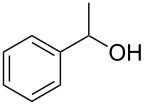	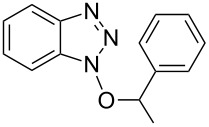	72	**1e**: 43%
6	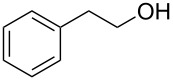	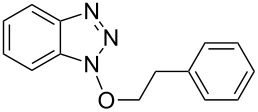	48	**1f**: 39%

^a^Reactions were conducted with 1.1 mmol of BOP (ca. 0.2 M in THF), 1.2 molar equiv of alcohols, and DBU, at room temperature. ^b^Yields are of isolated and purified products. ^c^Yield obtained with 2.7 molar equiv of MeOH. ^d^Yield obtained with MeOH as reaction solvent.

Reaction of BOP with either 1.4 or 2.7 molar equiv of MeOH, in the presence of 1.4 molar equiv of DBU, gave comparable yields of Bt-OMe (**1a**, 48% and 50%, respectively). Use of MeOH as reaction solvent itself resulted in a 47% yield of **1a**. These results seem to imply that maximal conversion of BOP to the *N*-alkoxybenzotriazoles is around 50%, possibly due to a competing reaction between BOP and DBU (see below). Nevertheless, 1° and 2° alcohols appear to react with BOP in the presence of a base, leading to the direct formation of *N*-alkoxybenzotriazoles.

Because reactions with BOP produce HMPA, a suspected nasal carcinogen, and the modest yields of the *N*-alkoxybenzotriazoles obtained, we decided to investigate other reactive BtOH derivatives for this reaction. A variety of phosphorus and sulfonate derivatives of BtOH has been synthesized and studied as peptide-coupling agents [30]. Amongst these we selected the easily synthesized tosylates of BtOH (Bt-OTs) and its 7-aza analogue AtOH (At-OTs). Furthermore, we have used Bt-OTs for conversion of aldoximes to nitriles and this reagent was generally comparable to BOP in those reactions. This factor additionally favored the selection of these two tosylate derivatives.

As can be seen from Table 2, Bt-OTs is superior to BOP, and a wide range of 1° and 2° alcohols underwent reaction with Bt-OTs giving good to excellent yields of 1-alkoxy-1*H*-benzotriazoles. Some notable results are as follows. Despite the leaving group ability of BtO^−^, elimination to styrene does not appear to be a significant problem in the reactions with the isomeric phenylethanols (Table 2, entries 5 and 6). Not unexpectedly, reaction with 1,3-butanediol occurred predominantly at the 1° hydroxy group (Table 2, entry 12). The reaction with propargyl alcohol proceeded uneventfully (Table 2, entry 14). The reaction of 4-nitrobenzyl alcohol (Table 2, entry 15) was complicated by the formation of 4-nitrobenzaldehyde. This can potentially occur by benzylic deprotonation, due to the enhanced acidity of these protons in **1o** or a reactive intermediate. Such a problem was not encountered in the reactions of other benzylic alcohols. Use of K_2_CO_3_ in place of DBU did not ameliorate this problem encountered with 4-nitrobenzyl alcohol. However, use of slightly modified conditions resulted in the formation of the desired product **1o** in good yield (see Supporting Information File 1 for details). Reaction of phenol with Bt-OTs resulted in the formation of the phenyl tosylate (Table 2, entry 16), indicating the potential use of this reagent as a tosylating agent for phenols. The outcome in the phenol reaction may be linked to the potential reaction pathway, an aspect that is described below. However, because phenoxide is a softer nucleophile as compared to alkoxide, we had to consider whether this was a factor in the reaction mechanism.

**Table 2:** Reactions of various alcohols with Bt-OTs.

Entry	Alcohol	Product	Time, temp	Compound: yield^a^

1	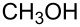	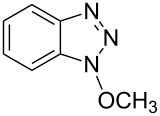	3 h, rt	**1a**: 66%
2	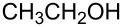	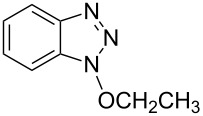	3 h, rt	**1b**: 85%
3	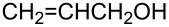	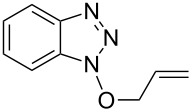	3 h, rt	**1c**: 73%
4	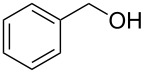	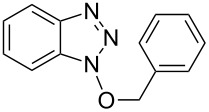	1.5 h, rt	**1d**: 91%
5	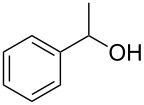	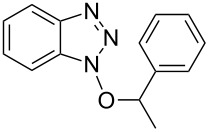	24 h, rt	**1e**: 77%
6	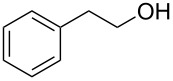	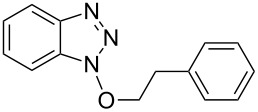	8 h, rt	**1f**: 90%
7	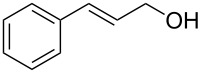	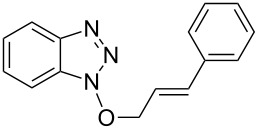	3 h, rt	**1g**: 83%
8	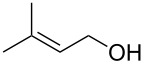	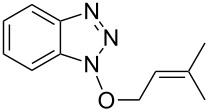	2.5 h, rt	**1h**: 74%
9	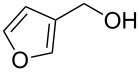	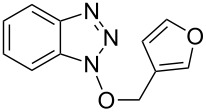	4 h, rt	**1i**:72%
10	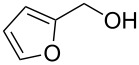	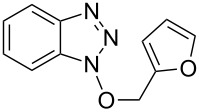	24 h, rt	**1j**: 51%
11	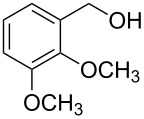	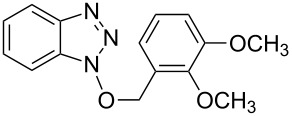	2.5 h, rt	**1k**: 87%
12	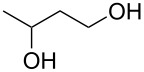	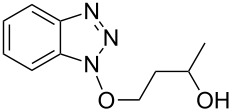	4 h, rt	**1l**: 48%
13	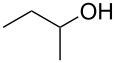	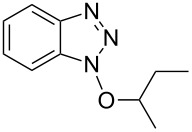	24 h, 60 °C	**1m**: 53%
14	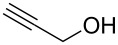	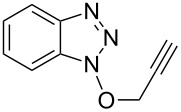	3.5 h, rt	**1n**: 79%
15	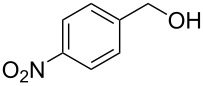	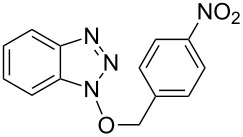	6 h, rt	**1o**: 68%
16	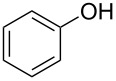	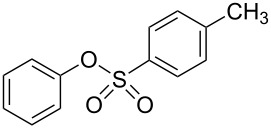	3 h, rt	**1p**: 84%

^a^Yields are of isolated and purified products.

In reactions of alcohols with BOP or Bt-OTs, the product structure is independent of the mechanism since all possible mechanisms result in the same product. However, for reactions with unsymmetrical peptide-coupling agents, an understanding of the mechanistic details would be required. Thus, the next stage in these investigations focused on this aspect.

### Mechanistic studies

**Figure 2:**
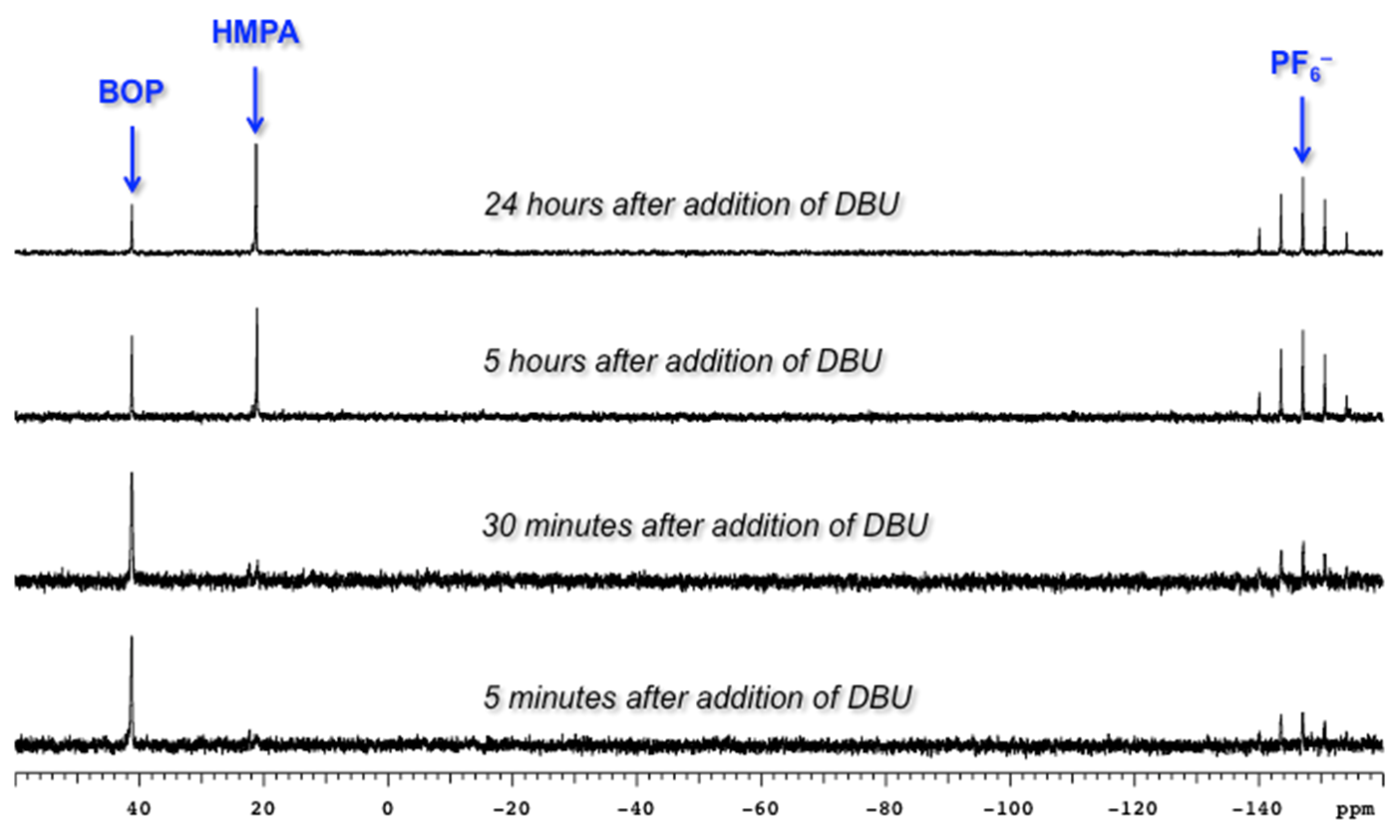
Evaluation of the reaction between 2-phenylethanol and BOP by ^31^P{^1^H} NMR.

Our investigations began with the reactions of BOP. We reasoned that among the three pathways shown in Scheme 2, ^31^P{^1^H} NMR may allow for distinguishing pathway **a** from **b** and **c**. Thus, we conducted experiments with 2-phenylethanol as a representative 1° alcohol. BOP and 2-phenylethanol were mixed in a 1:1 molar ratio at −78 °C in THF. The mixture was then transferred to the NMR probe maintained at −30 °C, and a spectrum was acquired. The only resonances observed were those of BOP (δ = 42.7 and −145.5 ppm). DBU (1 molar equiv) was added and the reaction was monitored every five minutes at −30 °C. The only observable resonance that began to emerge was that of HMPA (δ = 22.9 ppm). Reacquisition of data after leaving the mixture at room temperature overnight only showed an increase in the HMPA resonance (Figure 2).

We also conducted ^31^P{^1^H} experiments where BOP was independently exposed to DBU and Cs_2_CO_3_. In both cases, rapid disappearance of BOP was observed with concomitant formation of HMPA. Although at the present time we do not know the exact nature of the interaction of these bases with BOP, it is clear that a reaction occurs, and this may be responsible for the lower yields in the reactions of alcohols with BOP. These data additionally supported the use of Bt-OTs as an alternative.

**Scheme 3:**
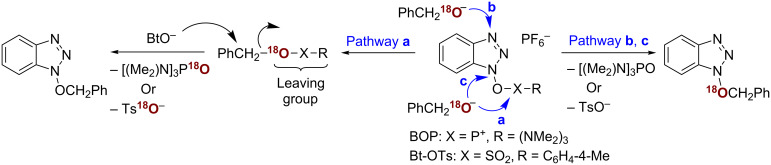
Possible products in the [^18^O]-labeling experiments.

Although no new phosphonium resonance from a new reactive species was observed in the NMR experiments, this did not necessarily exclude pathway **a**. Thus, we decided to pursue a second line of investigation via [^18^O]-labeling. For this experiment, we prepared PhCH_2_[^18^O]H via a known procedure [31]. As shown in Scheme 3, we reasoned that exclusive reaction via pathway **a** should produce an unlabeled product, reaction via pathway **b** and/or **c** should result in the [^18^O]-labeled product, and competing pathways should result in a mixture of labeled and unlabeled products.

With this mechanistic rationale two reactions of BOP were conducted in parallel with PhCH_2_OH as well as PhCH_2_[^18^O]H and DBU. The product yields from both reactions were nearly identical (ca 39%). High-resolution mass spectrometric analysis indicated that the ratio of the peak areas [M + 2]^+^/[M]^+^ was 0.015 for products from both reactions. Pathway **a** alone can account for this result and it appears that no leakage occurs via pathways **b** and **c**. A comparable [^18^O]-labeling experiment was conducted with Bt-OTs, where again no incorporation of the label was observed in the product, and the ratio of the peak areas [M + 2]^+^/[M]^+^ was 0.014. These results showed that both reagents, BOP and Bt-OTs, appear to react via similar pathways, not involving direct reactions at either the N1 or N3 atom in the benzotriazole ring. In support of this inference, a reaction of Bt-OTs with allyl alcohol was conducted at −78 °C. Quenching this reaction after 30 min, followed by preparative TLC of a portion of the mixture, led to the isolation of the allyl alcohol tosylate [32–33]. However, even at this low temperature, formation of 1-(allyloxy)-1*H*-benzotriazole (**1c**) was clearly evident, indicating the ease of this transformation. In the light of these results, the reaction of phenol with Bt-OTs is consistent with the proposed pathway **a**, and it appears that both alkoxides and phenoxides react in a similar manner. The mechanistic basis in reactions of alcohols with BOP and Bt-OTs described above appears to parallel that reported for the activation of carboxylic acids by BOP. In the carboxylic acid activation studies, only two mechanisms were proposed, namely reaction of the carboxylate at the phosphorus center (equivalent to pathway **a** in Scheme 3) or a S_N_2’ reaction at the N3 atom (equivalent to pathway **b** in Scheme 3) [34]. Experiments with [^18^O]-labeled benzoate indicated that conversion of carboxylic acids to the acyl HOBt derivatives occurs by a two-step process, via an intermediate acyloxyphosphonium ion [34].

**Scheme 4:**
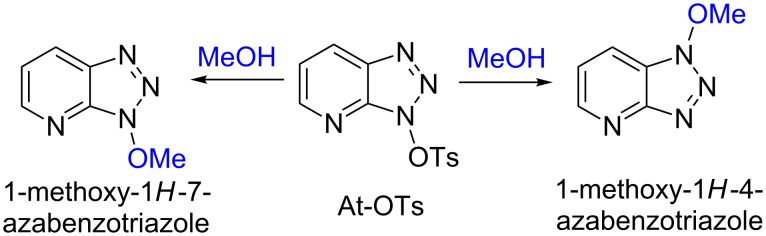
Two possible products from the reaction of At-OTs with MeOH.

One final set of experiments was conducted to evaluate the mechanism in the context of a desymmetrized benzotriazole. For this we considered the reaction of At-OTs [30] with MeOH, where we believed location of the OMe moiety could be ascertained relative to the aromatic protons via NOE experiments (Scheme 4).

Reaction of At-OTs with MeOH yielded a 1-methoxyazabenzotriazole (At-OMe), and the ^1^H NMR spectrum of the crude reaction mixture indicated the presence of only one product. Comparison of the ^1^H NMR data of the purified material to those reported [35] for 1-methoxy-1*H*-4-azabenzotriazole (1-methoxy-1*H*-[1,2,3]triazolo[4,5-*b*]pyridine) and 1-methoxy-1*H*-7-azabenzotriazole (3-methoxy-3*H*-[1,2,3]triazolo[4,5-*b*]pyridine) did not allow for ready identification. A NOE experiment did not result in observable interactions of the OMe resonance with the aromatic system. Because neither experiment allowed for unambiguous discrimination between the two structures, 1-methoxy-1*H*-7-azabenzotriazole was prepared via a known procedure [35]. The chemical shifts for the aromatic protons in the authentic 1-methoxy-1*H*-7-azabenzotriazole prepared, the product obtained from the reaction of At-OTs and MeOH, as well as the literature data are shown in Table 3. From the Δδ values in this table, it becomes clear that the product obtained in Scheme 4 is in fact 1-methoxy-1*H*-7-azabenzotriazole. Additionally, the melting point of this product was 94.5–95.5 °C, which is consistent with that reported for 1-methoxy-1*H*-7-azabenzotriazole (93–94 °C [35]) and the authentic material synthesized herein (94–95 °C). By contrast, the reported melting point of 1-methoxy-1*H*-4-azabenzotriazole is significantly higher (140–144 °C) [35].

**Table 3:** Comparison of chemical shifts for the aromatic protons in the isomeric 1-methoxyazabenzotriazoles.^a^

Compound	Ar–H chemical shift (δ ppm)	Δδ (ppm)

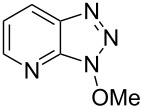	8.73	8.73 − 8.37 = 0.36
	8.37	8.37 − 7.41 = 0.96
	7.41	8.73 − 7.41 = 1.32
1-Methoxy-1*H*-azabenzotriazole obtained by reaction of At-OTs + MeOH	8.67	8.67 − 8.31 = 0.36
	8.31	8.31 − 7.36 = 0.95
	7.36	8.67 − 7.36 = 1.31
	
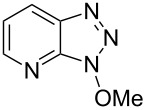 ^b^	8.75	8.75 − 8.40 = 0.35
	8.40	8.40 − 7.43 = 0.97
	7.43	8.75 − 7.43 = 1.32
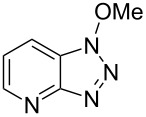 ^b^	8.80	8.80 − 8.04 = 0.36
	8.04	8.04 − 7.52 = 0.52
	7.52	8.80 − 7.52 = 1.28

^a^Spectra were obtained in CDCl_3_. ^b^Reference [35].

Having ascertained the overall mechanism by which alcohols react with Bt-OTs as well as At-OTs, and that this mechanism is not altered by the presence of the additional nitrogen atom in At-OTs, the reactions of At-OTs with alcohols were then evaluated (Table 4). Reactions with At-OTs appear to be more temperature sensitive than those with Bt-OTs, and reaction mixtures can turn to dark colors at elevated temperatures.

**Table 4:** Reactions of various alcohols with At-OTs.

Entry	Alcohol	Product	Time, temp	Compound: yield^a^

1	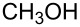	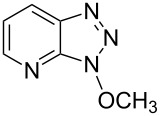	24 h, rt	**2a**: 74%
2	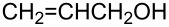	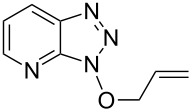	24 h, rt	**2b**: 69%
3	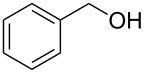	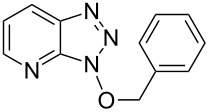	24 h, rt	**2c**: 80%
4	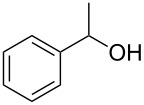	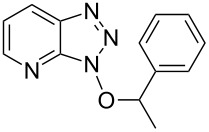	24 h, 60 °C	**2d**: 64%
5	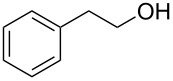	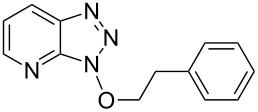	24 h, rt	**2e**: 68%
6	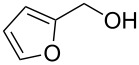	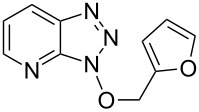	24 h, rt	**2f**: 67%
7	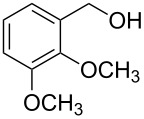	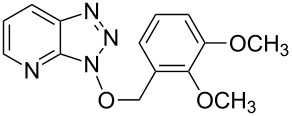	24 h, rt	**2g**: 68%
8	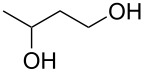	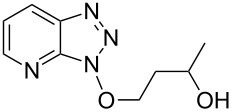	24 h, 60 °C	**2h**: 59%
9	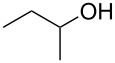	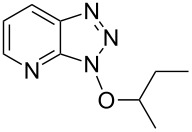	24 h, 60 °C	**2i**: 34%
10	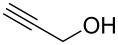	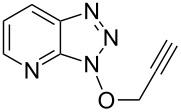	2 h, rt	**2j**: 73%

^a^Yields are of isolated and purified products.

**Scheme 5:**
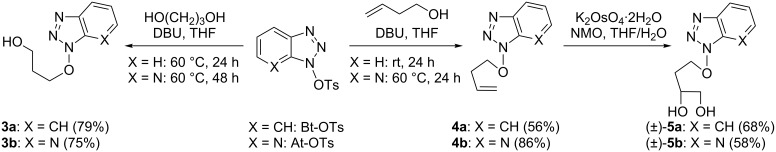
Synthesis of acyclic nucleoside-like compounds.

After demonstrating the one-step preparation of 1-alkoxy-1*H*-benzo- and 7-azabenzotriazoles, our next focus was the potential applications of this chemistry. Since the heterocyclic moiety resembles a nucleobase, we first considered synthesis of nucleoside-like entities (Scheme 5). In this context, *N*-alkylbenzotriazolyl derivatives and ribonucleoside analogues containing a benzotriazole as a nucleobase surrogate have shown interesting activities towards NTPase/helicase of *Flaviviridae* viruses [1–2]. Compounds **3a**,**b** and **5a**,**b** shown in Scheme 5 are both N-substituted benzotriazoles and, upon appropriate folding of the aliphatic chain, they resemble acyclic nucleosides (**3a**,**b**) and ribonucleosides (**5a**,**b**).

Reaction of Bt-OTs and At-OTs with 1,3-propanediol gave products **3a** and **3b**, arising from reaction at one hydroxy group, in good yields. In these reactions minor amounts of products arising by reaction at both hydroxy groups were observed. Similarly, reactions of 3-butenol gave products **4a** and **4b** in good to high yields, which were converted to the acyclic ribonucleoside-like diols (±)-**5a** and (±)-**5b**, respectively.

**Figure 3:**
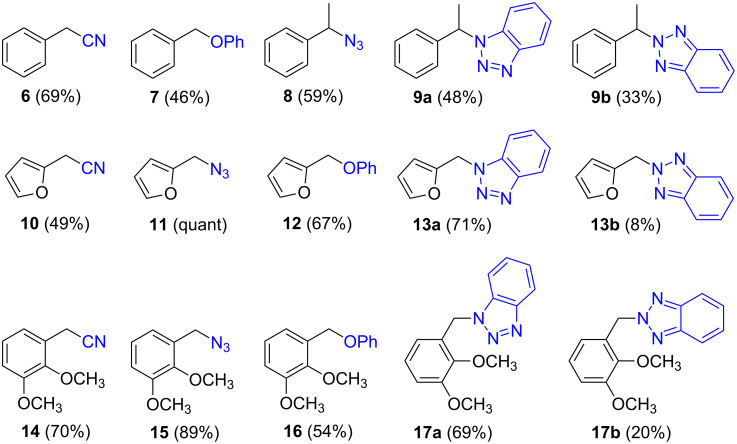
Products from the nucleophilic substitution reactions.

We next assessed the leaving group ability of the benzotriazolyloxy group. Although carboxylic acid esters of benzotriazole react efficiently with nucleophiles, this is mechanistically distinct from direct displacement. Thus, four of the 1-alkoxy-1*H*-benzotriazoles (three 1° and one 2°) were utilized in substitution reactions with cyanide, azide, phenoxide, and benzotriazole. These reactions were conducted in DMSO at 100 °C and the results are shown in Figure 3. In the presence of Cs_2_CO_3_, reactions with benzotriazole as nucleophile yielded the N1- and N2-alkyl products in variable ratios, but in good overall yields (>75%). Whereas the 1-alkoxy-1*H*-benzotriazoles used in these reactions were benzylic, and therefore more reactive, we also assessed the reactivity of 1-phenethoxy-1*H*-benzotriazole (**1f**). Reaction of compound **1f** with NaN_3_ in DMSO at 100 °C for 28 h led to the formation of (2-azidoethyl)benzene, as assessed by ^1^H NMR. However, this reaction was incomplete and about 12% of **1f** remained unreacted. Nevertheless, these results indicate the leaving group ability of BtO^−^ from benzylic sp^3^ carbon centers and are interesting in the context of the previously unknown reactivity of this class of compounds. Whether the At-OR derivatives are more reactive in such reactions will be interesting to evaluate in the future.

**Figure 4:**
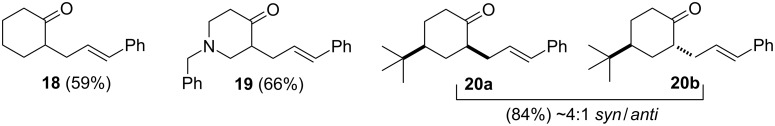
γ,δ-Unsaturated cycloalkanones obtained from **1g**.

On the basis of the leaving group ability of BtO^−^ in the substitution reactions shown above, our final question was about the reactivity of an allylic benzotriazolyl derivative under palladium-catalyzed conditions. The benzoyl ester of BtOH has been evaluated in a decarboxylative Pd-mediated Heck reaction, leading to a modest product yield [36]. However, this appears to be the only example of a BtOH derivative in Pd-mediated reactions. In principle, formation of π–allyl complexes from allylic benzotriazolyloxy products described herein should be feasible. In this context, *N*-allylbenzotriazole derivatives undergo reaction with preformed enamines, in the presence of Pd(OAc)_2_ and PPh_3_ [37]. Super-stoichiometric ZnBr_2_ was necessary for these reactions, in the absence of which no reaction was observed [37]. With these data in mind, we decided to evaluate a few reactions of cinnamyloxy benzotriazolyl derivative **1g**. However, instead of using preformed enamines, we chose to utilize a combination of metal- and organocatalysis, wherein the enamine is formed in situ [38]. Exposure of 1.5 molar equiv each of cyclohexanone, *N*-benzylpiperidone, and 4-*tert*-butylcyclohexanone to compound **1g**, Pd(PPh_3_)_4_ (5 mol %), and pyrrolidine (30 mol %) in DMSO at room temperature, led to the corresponding γ,δ-unsaturated cycloalkanones (Figure 4).

Good yields of products **18** and **19** were obtained (literature yields are superior, ca. 90% [37–38]) and the yield of compound **20** was excellent. Although the present conditions are not optimized, these results appear to indicate that the yields may be dependent upon the nature of the cycloalkanone and not solely upon the reactivity of the allylic benzotriazolyl derivative. However, there are some notable factors. ZnBr_2_ is essential to the formation of π–allyl complexes from *N*-allylbenzotriazole derivatives, by assisting in the departure of the benzotriazolyl anion [37]. In the current cases, no additive is necessary for the departure of BtO^−^. Furthermore, the enamine was formed in situ in this study, with catalytic pyrrolidine. These results appear to indicate that the easily synthesized allylic benzotriazolyl derivatives described herein may be promising reagents for the α-allylation of carbonyl compounds.

**Scheme 6:**
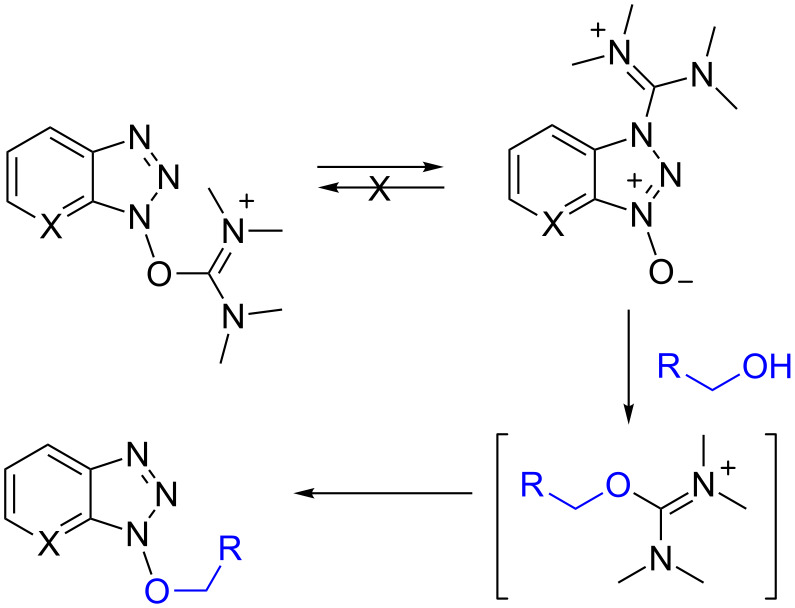
Formation of Bt-OR and At-OR from HBTU and HATU type of compounds.

As a final note, while this work was in progress, synthesis of pyridopyrazine-1,6-diones was reported, beginning from 6-hydroxypicolinic acids and amino ethanols [39]. Here, HATU (Figure 1) not only functioned in the conventional role of carboxylic acid activating agent for amide formation, but it was serendipitously discovered that HATU also caused an unusual activation of the alcohol moiety, leading to N-alkylation. In the presence of iPr_2_NEt, reaction of HATU with benzyl-, *n*-butyl-, and *p*-nitrobenzyl alcohol led to the formation of the corresponding 1-alkoxy-1*H*-7-azabenzotriazoles [39]. The regiochemistry in these reactions was identical to that reported here. What is notable about reagents such as HBTU and HATU is that they are commonly encountered in the guanidinium (N form) rather than the uronium (O form). Although the uronium form can be synthesized and is a more reactive species, it undergoes rapid isomerization to the guanidinium form in the presence of bases [40]. Thus, it can be reasonably anticipated that formation of Bt-OR and At-OR from the reactions of alcohols with HBTU and HATU would proceed via the intermediacy of a uronium salt of the alcohol, leading to a regiochemical outcome with unsymmetrical reagents as shown in Scheme 6.

Because the guanidinum forms of HBTU and HATU have lower reactivity than the uronium forms [40] slightly more forcing conditions may be needed to obtain satisfactory reactivity. Consistent with this, compound **2c** was obtained in 83% yield from a reaction of benzyl alcohol, HATU, and iPr_2_NEt, in refluxing CH_2_Cl_2_ for 16–20 h [39]. By contrast, compound **2c** was obtained in a comparable 80% yield in a 24 h reaction with At-OTs and DBU at room temperature. In our hands however, reaction of HATU with some other alcohols of interest have not been successful so far.

## Conclusion

In this study we have investigated a one-step alkylation 1-hydroxy-1*H*-benzotriazole (BtOH) and its 7-aza analogue (AtOH), via a previously unknown reaction of alcohols with benzotriazole-based peptide-coupling agents. Although reactions of alcohols proceeded with (benzotriazol-1-yloxy)tris(dimethylamino)phosphonium hexafluorophosphate (BOP) and DBU, Bt-OTs was a more effective reagent. Correspondingly, the 7-aza derivatives were synthesized from At-OTs. Methodologically, this one-step process allows for the direct conversion of alcohols to *N*-alkoxy benzo- and azabenzotriazoles, eliminating the necessity for alkyl halides or more cumbersome methods. Mechanistic studies indicate that reactions of alcohols with these peptide-coupling reagents, in the presence of a base, appear to proceed by formation of alcohol phosphonium derivatives (with BOP), or alcohol tosylates (with Bt-OTs), and not through direct displacement of the leaving group by attack at the N1 or by a S_N_2’-type of process by reaction at the N3 of the triazolyl moiety. The mechanistic analyses were conducted by a combination of ^31^P{^1^H}, [^18^O]-labeling, and other chemical experiments. The reaction of phenol with Bt-OTs yielded only the phenyl tosylate, which is consistent with this mechanism. This reaction also shows that Bt-OTs (and At-OTs) could serve as tosylating agents for phenols as well. Further, the utilities of this reaction, as well as some of the products have been explored. In this vein, acyclic nucleoside-like compounds containing benzo- and azabenzotriazole as a nucleobase surrogate have been synthesized. Because benzotriazole derivatives have potentially important pharmacological applications, we anticipate expansion of this chemistry in the future to a broader range of nucleoside-like entities for biological assays. The ability of BtO^−^ to function as a nucleofuge, led us to explore its displacement. In this context, several 1-alkoxy-1*H*-benzotriazoles obtained from benzylic alcohols underwent substitution reaction with a range of nucleophiles, and a simple alkyl derivative also underwent reaction although the reaction was slightly incomplete. Finally, we have evaluated the departure of BtO^−^ from an allylic position leading to a putative Pd π–allyl complex. In unoptimized preliminary results, Pd-catalyzed α-allylation of three cyclic ketones was accomplished with a cinnamyloxy benzotriazolyl derivative, through in situ formed pyrrolidine enamines. Overall, the potential scope of this new chemistry appears promising, ranging from the development of novel molecules with new applications, to synthetic methodology. We anticipate reporting additional developments in this area in the future.

## Experimental

### General experimental considerations

Thin-layer chromatography was performed on 200 μm aluminum-foil-backed silica gel plates. Column chromatographic purifications were performed on 200–300 mesh silica gel. THF was distilled from LAH and then redistilled from Na prior to use. Ethyl acetate (EtOAc) and hexanes were distilled from CaSO_4_, commercial CH_2_Cl_2_ was redistilled. Other commercially available compounds were used without further purification. ^1^H NMR spectra were recorded at 500 MHz and are referenced to the residual protonated solvent resonance. ^13^C NMR spectra were recorded at 125 MHz and are referenced to the solvent resonance. Chemical shifts (δ) are reported in parts per million (ppm) and coupling constants (*J*) are in hertz (Hz). Standard abbreviations are used to designate resonance multiplicities.

#### General procedure for the reactions of alcohols with BOP

In a dry vial equipped with a stirring bar was placed BOP (0.486 mg, 1.1 mmol) in anhydrous THF (5 mL). The alcohol (1.36 mmol) was added, followed by the dropwise addition of DBU (1.36 mmol). The reaction mixture was stirred at room temperature for the duration indicated in Table 1. The mixture was diluted with EtOAc, washed with brine, and then with water. The organic layer was separated, dried over anhydrous Na_2_SO_4_, and evaporated under reduced pressure. Products were purified by chromatography on a silica gel column using a gradient of EtOAc in hexanes. The products from these reactions were identical to those produced from the reactions of Bt-OTs for which full characterization is provided.

#### General procedure for the reactions of alcohols with Bt-OTs

In a dry vial equipped with a stirring bar was placed Bt-OTs in anhydrous THF. The alcohol was added, followed by the dropwise addition of DBU. The reaction mixture was stirred either at room temperature or at 60 °C for the duration indicated in Table 2 and then worked up. The stoichiometry of reactants was dependent upon the volatility of the alcohol. Generally, with lower boiling alcohols, Bt-OTs was the limiting reagent, whereas Bt-OTs was used in excess with higher boiling ones. Also, if initial experiments gave poorer results with a lower amount of an alcohol, then reactions were conducted with higher excesses of the alcohol. Specific experimental and work-up details are provided under the individual compound headings.

### Representative examples

#### 1-(1-Phenylethoxy)-1*H*-benzo[*d*][1,2,3]triazole (**1e**)

The compound was synthesized from Bt-OTs (1.16 g, 4.0 mmol), 1-phenylethanol (580 μL, 4.8 mmol), and DBU (720 μL, 4.8 mmol) in anhydrous THF (20 mL) over 24 h at room temperature. The volatiles were evaporated and the crude material was purified on a silica gel column using 6% EtOAc in hexanes as eluting solvent. Compound **1e** was obtained as a white solid (0.282 g, 77% yield). *R*_f_ 0.38 (SiO_2_/30% EtOAc in hexanes); ^1^H NMR (500 MHz, CDCl_3_) δ 7.91 (d, *J* = 8.3 Hz, 1H, Ar-H), 7.36 (m, 2H, Ar-H), 7.29–7.27 (m, 5H, Ar-H), 7.13 (d, *J* = 8.3 Hz, 1H, Ar-H), 5.76 (q, *J* = 6.6 Hz, 1H, OCH), 1.86 (d, *J* = 6.3 Hz, 3H, CH_3_); ^13^C NMR (125 MHz, CDCl_3_) δ 143.1, 138.1, 129.4, 128.7, 128.2, 127.6, 127.4, 124.2, 119.8, 108.9, 88.9, 19.9; HRMS–ESI TOF (*m*/*z*): [M + H]^+^ calcd for C_14_H_14_N_3_O, 240.1131; found, 240.1121.

#### 3-(Prop-2-yn-1yloxy)-3*H*-[1,2,3]triazolo[4,5,*b*]pyridine (**2j**)

The compound was synthesized from At-OTs (0.159 g, 0.55 mmol), propargyl alcohol (60 μL, 0.5 mmol), and DBU (89 μL, 0.6 mmol) in anhydrous THF (2.5 mL) over 2 h at room temperature. The reaction mixture was partitioned between EtOAc and water. The organic layer was separated, dried over anhydrous Na_2_SO_4_, and evaporated under reduced pressure. The crude product was chromatographed on a silica gel column by sequential elution with 50% and 60% EtOAc in hexanes. Compound **2j** was obtained as colorless solid (63.5 mg, 73% yield). *R*_f_ 0.24 (SiO_2_/30% EtOAc in hexanes); ^1^H NMR (500 MHz, CDCl_3_) δ 8.72 (dd, *J* = 1.5, 4.4 Hz, 1H, Ar-H), 7.37 (dd, *J* = 1.4, 8.4 Hz, 1H, Ar-H), 7.41 (dd, *J* = 4.4, 8.4 Hz, 1H, Ar-H), 5.27 (d, *J* = 2.6 Hz, 2H, OCH_2_), 2.60 (t, *J* = 2.4 Hz, 1H, ≡C-H); ^13^C NMR (125 MHz, CDCl_3_) δ 151.6, 140.2, 135.1, 129.5, 121.0, 79.9, 75.4, 67.9; HRMS–ESI TOF (*m/z*): [M + H]^+^ calcd for C_8_H_7_N_4_O, 175.0614; found, 175.0621.

#### 1-Benzyl-3-cinnamylpiperidin-4-one (**19**)

To a solution of cinnamyloxybenzotriazole (**1g**, 125.6 mg, 0.50 mmol) in DMSO (2 mL), Pd(PPh_3_)_4_ (28.8 mg, 25 μmol, 5 mol %) was added, and the mixture was stirred at room temperature for 5 min. Then *N*-benzylpiperidone (278 µL, 1.50 mmol) and pyrrolidine (12 µL, 0.15 mmol, 30 mol %) were added. The reaction vial was flushed with nitrogen gas and the mixture was stirred at room temperature for 2 h. The mixture was then diluted with EtOAc and was washed with water followed by brine. The organic layer was dried over anhydrous Na_2_SO_4_ and evaporated. The crude material was chromatographed on a silica gel column using 18% EtOAc in hexanes as eluting solvent. Compound **19** was obtained as a pale yellow solid (100.9 mg, 66% yield). *R*_f_ 0.26 (SiO_2_/30% EtOAc in hexanes); ^1^H NMR (500 MHz, CDCl_3_) δ 7.34–7.27 (m, 9H, Ar-H), 7.22–7.18 (m, 1H, Ar-H), 6.37 (d, *J* = 15.8 Hz, 1H, =CH), 6.14 (dt, *J* = 7.5, 15.4 Hz, 1H, =CH), 3.70 and 3.53 (two d, *J*_A,B_ = 12.7 Hz, 2H, CH_2_), 3.09 and 3.00 (two AB m, 2H, CH_2_), 2.69–2.22 (m, 7H, CH_2_, CH_2_, CH_2_, CH); ^13^C NMR (125 MHz, CDCl_3_) δ 210.4, 138.4, 137.5, 132.1, 129.1, 128.7, 127.7, 127.3, 126.3, 62.0, 58.6, 53.5, 49.9, 41.1, 31.2; HRMS–ESI TOF (*m/z*): [M + H]^+^ calcd for C_21_H_24_NO, 306.1852; found, 306.1833.

## Supporting Information

File 1 Experimental.

File 2 NMR spectra.
