# Supplementary material for: Facile synthesis of 1-alkoxy-1H-benzo- and 7-azabenzotriazoles from peptide coupling agents, mechanistic studies, and synthetic applications
Source: Beilstein J Org Chem. 2014 Aug 19;10:1919–32. doi: 10.3762/bjoc.10.200 (PMC4168895; doi:10.3762/bjoc.10.200)
Supplement: File 1 — Experimental. [file Beilstein_J_Org_Chem-10-1919-s001.pdf]

Supporting Information 1

for

Facile synthesis of 1-alkoxy-1*H*-benzo- and 7-azabenzotriazoles from peptide coupling agents, mechanistic studies, and synthetic applications

Mahesh K. Lakshman*, Manish K. Singh, Mukesh Kumar, Raghu Ram Chamala, Vijayender R. Yedulla, Domenick Wagner, Evan Leung, Lijia Yang, Asha Matin, and Sadia Ahmad

Address: Department of Chemistry, The City College and The City University of New York, 160 Convent Avenue, New York, NY 10031, USA

Email: Mahesh K. Lakshman* - lakshman@sci.ccny.cuny.edu

*Corresponding author

Experimental

Table of contents

Information	Page
1-Methoxy-1*H*-benzo[*d*][1,2,3]triazole **(1a)**	S-3
1-Ethoxy-1*H*-benzo[*d*][1,2,3]triazole **(1b)**	S-3
1-(Allyloxy)-1*H*-benzo[*d*][1,2,3]triazole **(1c)**	S-3
1-(Benzyloxy)-1*H*-benzo[*d*][1,2,3]triazole **(1d)**	S-4
1-Phenethoxy-1*H*-benzo[*d*][1,2,3]triazole **(1f)**	S-4
1-(Cinnamyloxy)-1*H*-benzo[*d*][1,2,3]triazole **(1g)**	S-5
1-((3-Methylbut-2-en-1-yl)oxy)-1*H*-benzo[*d*][1,2,3]triazole **(1h)**	S-5
1-(Furan-3-ylmethoxy)-1*H*-benzo[*d*][1,2,3]triazole **(1i)**	S-6
1-(Furan-2-ylmethoxy)-1*H*-benzo[*d*][1,2,3]triazole **(1j)**	S-6
1-((2,3-Dimethoxybenzyl)oxy)-1*H*-benzo[*d*][1,2,3]triazole **(1k)**	S-7
4-((1*H*-Benzo[*d*][1,2,3]triazol-1-yl)oxy)butan-2-ol **(1l)**	S-7
1-(*sec*-Butoxy)-1*H*-benzo[*d*][1,2,3]triazole **(1m)**	S-8
1-(Prop-2-yn-1-yloxy)-1*H*-benzo[*d*][1,2,3]triazole **(1n)**	S-8
1-((4-Nitrobenzyl)oxy)-1*H*-benzo[*d*][1,2,3]triazole **(1o)**	S-9
Phenol tosylate **(1p)**	S-9

3-Methoxy-3*H*-[1,2,3]triazolo[4,5-*b*]pyridine **(2a)**	S-10
3-(Allyloxy)-3*H*-[1,2,3]triazolo[4,5-*b*]pyridine **(2b)**	S-10
3-(Benzyloxy)-3*H*-[1,2,3]triazolo[4,5-*b*]pyridine **(2c)**	S-11
3-(1-Phenylethoxy)-3*H*-[1,2,3]triazolo[4,5-*b*]pyridine **(2d)**	S-11
3-Phenethoxy-3*H*-[1,2,3]triazolo[4,5-*b*]pyridine **(2e)**	S-12
3-(Furan-2-ylmethoxy)-3H-[1,2,3]triazolo[4,5-*b*]pyridine **(2f)**	S-12
3-((2,3-Dimethoxybenzyl)oxy)-3*H*-[1,2,3]triazolo[4,5-*b*]pyridine **(2g)**	S-13
4-((3*H*-[1,2,3]triazolo[4,5-*b*]pyridin-3-yl)oxy)butan-2-ol **(2h)**	S-13
(3-*sec*-Butoxy)-3*H*-[1,2,3]triazolo[4,5-*b*]pyridine **(2i)**	S-14
3-(1*H*-Benzo[*d*][1,2,3]triazol-1-yl)oxy)propan-1-ol **(3a)**	S-14
3-((3*H*-[1,2,3]Triazolo[4,5-*b*]pyridin-3-yl)oxy)propan-1-ol **(3b)**	S-15
3-(But-3-enyloxy)-1*H*-benzo[*d*][1,2,3,]triazole **(4a)**	S-16
3-(But-3-enyloxy)-3*H*-[1,2,3]triazolo[4,5-*b*]pyridine **(4b)**	S-16
(±)-4-(1*H*-Benzo[*d*][1,2,3]triazol-1-yl)oxy)butane-1,2-diol **(5a)**	S-17
(±)-4-((3*H*-[1,2,3]Triazolo[4,5-*b*]pyridin-3-yl)oxy)butane-1,2-diol **(5b)**	S-17
Benzyl nitrile **(6)**	S-18
Benzyl phenyl ether **(7)**	S-18
(1-Azidoethyl)benzene **(8)**	S-19
1-(1-Phenylethyl)-1*H*-benzo[*d*][1,2,3]triazole **(9a)** and 2-(1-phenylethyl)-2*H*-benzo[*d*][1,2,3]-triazole **(9b)**	S-19
(Furan-2-yl)acetonitrile **(10)**	S-20
2-(Azidomethyl)furan **(11)**	S-20
2-(Phenoxymethyl)furan **(12)**	S-21
1-(Furan-2-ylmethyl)-1*H*-benzo[*d*][1,2,3]triazole **(13a)** and 2-(furan-2-ylmethyl)-2*H*-benzo[*d*][1,2,3]triazole **(13b)**	S-21
2-(2,3-Dimethoxyphenyl)acetonitrile **(14)**	S-22
1-(Azidomethyl)-2,3-dimethoxybenzene **(15)**	S-23
1,2-Dimethoxy-3-(phenoxymethyl)benzene **(16)**	S-23
1-(2,3-Dimethoxybenzyl)-1*H*-benzo[*d*][1,2,3]triazole **(17a)** and 2-(2,3-dimethoxybenzyl)-2*H*-benzo[*d*][1,2,3]triazole **(17b)**	S-24
Reaction of 1-phenethoxy-1*H*-benzo[*d*][1,2,3]triazole **(1f)** with NaN_3 leading to (2-azidoethyl)benzene	S-25
2-Cinnamylcyclohexan-1-one **(18)**	S-25
Syn-4-(*t*-butyl)-2-cinnamylcyclohexan-1-one **(20a)** and *anti*-4-(*t*-butyl)-2-cinnamylcyclohexan-1-one **(20b)**	S-26
Conditions for the $^{31}P\{^1H\}$ NMR experiments.	S-27
**Figure 1:** Mass spectrum of the product obtained from the reaction of BOP with $PhCH_2OH$.	S-28
**Figure 2:** Mass spectrum of the product obtained from the reaction of BOP with $PhCH_2{}^{18}OH$.	S-28
**Figure 3.** Mass spectrum of the product obtained from the reaction of Bt–OTs with $PhCH_2{}^{18}OH$.	S-29
References	S-30

1-Methoxy-1*H*-benzo[*d*][1,2,3]triazole (1a) [1]**:** Synthesized from Bt–OTs (0.578 g, 2.0 mmol), MeOH (184 µL, 0.45 mmol), and DBU (0.45 mL, 3.0 mmol) in anhydrous THF (10 mL), over 3 h at room temperature. The volatiles were evaporated and the crude material was chromatographed on a silica gel column by sequential elution with hexanes, 2%, 4%, 8%, and finally 20% EtOAc in hexanes. Compound **1a** was obtained as a white solid (0.198 g, 66% yield). R_f (SiO_2/30% EtOAc in hexanes) = 0.42; ^{1}H NMR (500 MHz, $CDCl_3$) δ 8.00 (d, 1H, J = 8.3 Hz), 7.58 (d, 1H, J = 8.3 Hz), 7.51 (t, 1H, J = 7.8 Hz), 7.38 (t, 1H, J = 7.3 Hz), 4.39 (s, 3H, OCH_3); ^{13}C NMR (125 MHz, $CDCl_3$) δ 143.7, 128.2, 126.8, 124.8, 120.4, 108.7, 67.8; HRMS (ESI/TOF) *m/z* calcd for $C_7H_8N_3O$ $[M + H]^+$ 150.0662, found 150.0679.

1-Ethoxy-1*H*-benzo[*d*][1,2,3]triazole (1b) [2]**:** Synthesized from Bt–OTs (0.954 g, 3.3 mmol), EtOH (0.28 mL, 3.63 mmol), and DBU (0.74 mL. 4.9 mmol) in anhydrous THF (16.5 mL), over 3 h at room temperature. The volatiles were evaporated and the crude material was chromatographed on a silica gel column using 4% EtOAc in hexanes as eluting solvent. Compound **1b** was obtained as clear liquid (0.457 g, 85% yield). R_f (SiO_2/20% EtOAc in hexanes) = 0.33; ^{1}H NMR (500 MHz, $CDCl_3$) δ 8.01 (d, 1H, Ar-H, J = 8.5 Hz), 7.58 (d, 1H, Ar-H, J = 8.4 Hz), 7.51 (t, 1H, Ar-H, J = 7.7 Hz), 7.39 (t, 1H, Ar-H, J = 7.6 Hz), 4.63 (q, 2H, OCH_2, J = 7.1 Hz), 1.49 (t, 3H, CH_3, J = 7.1 Hz); ^{13}C NMR (125 MHz, $CDCl_3$) δ 143.4, 128.0, 127.6, 124.6, 120.0, 108.7, 76.6, 13.7; HRMS (ESI/TOF) *m/z* calcd for $C_8H_{10}N_3O$ $[M + H]^+$ 164.0818, found 164.0797.

1-(Allyloxy)-1*H*-benzo[*d*][1,2,3]triazole (1c) [3]**:** Synthesized from Bt–OTs (0.868 g, 3.00 mmol), allyl alcohol (0.22 mL, 3.24 mmol), and DBU (0.67 mL, 4.48 mmol) in anhydrous THF (15 mL), over 3 h at room temperature. The reaction mixture was partitioned between EtOAc and water. The organic layer was separated, dried over anhydrous Na_2SO_4, and evaporated

under reduced pressure. The crude material was chromatographed on a silica gel column using 4% EtOAc in hexanes as eluting solvent. Compound **1c** was obtained as clear, oily liquid (0.376 g, 73% yield). R_f (SiO_2/20% EtOAc in hexanes) = 0.38; ^{1}H NMR (500 MHz, $CDCl_3$) δ 7.92 (d, 1H, Ar-H, J = 8.4 Hz), 7.50 (d, 1H, Ar-H, J = 8.3 Hz), 7.43 (t, 1H, Ar-H, J = 7.8 Hz), 7.30 (t, 1H, Ar-H, J = 8.2 Hz), 6.05 (m, 1H, =CH), 5.29–5.25 (m, 2H, =CH_2), 4.96 (d, 2H, OCH_2, J = 6.8 Hz); ^{13}C NMR (125 MHz, $CDCl_3$) δ 143.4, 130.1, 125.0, 127.9, 124.6, 123.5, 120.1, 108.9, 81.2; HRMS (ESI/TOF) *m/z* calcd for $C_9H_{10}N_3O$ $[M + H]^+$ 176.0818, found 176.0811.

1-(Benzyloxy)-1*H*-benzo[*d*][1,2,3]triazole (1d) [4]**:** Synthesized from Bt–OTs (0.335 g, 1.16 mmol), benzyl alcohol (0.1 mL, 0.966 mmol), and DBU (0.21 mL, 1.4 mmol) in anhydrous THF (5 mL), over 1.5 h at room temperature. The reaction mixture was partitioned between EtOAc and water. The organic layer was separated, dried over anhydrous Na_2SO_4, and evaporated under reduced pressure. The crude product was chromatographed on a silica gel column using 5% EtOAc in hexanes as eluting solvent. Compound **1d** was obtained as thick, clear liquid (0.198 g, 91% yield). R_f (SiO_2/20% EtOAc in hexanes) = 0.39; ^{1}H NMR (500 MHz, $CDCl_3$) δ 7.95 (d, 1H, Ar-H, J = 8.2 Hz), 7.37–7.28 (m, 7H, Ar-H), 7.17 (d, 1H, Ar-H, J = 8.1 Hz), 5.53 (s, 2H, OCH_2); ^{13}C NMR (125 MHz, $CDCl_3$) δ 143.5, 133.3, 130.2, 130.0, 129.0, 128.1, 127.9, 124.6, 120.2, 108.9, 82.8; HRMS (ESI/TOF) *m/z* calcd for $C_{13}H_{12}N_3O$ $[M + H]^+$ 226.0975, found 226.0970.

1-Phenethoxy-1*H*-benzo[*d*][1,2,3]triazole (1f): Synthesized from Bt–OTs (0.995 g, 3.44 mmol), 2-phenylethanol (0.34 mL, 2.86 mmol), and DBU (0.64 mL, 4.28 mmol) in anhydrous THF (15 mL), over 8 h at room temperature. The reaction mixture was partitioned between EtOAc and water. The organic layer was separated, dried over anhydrous Na_2SO_4, and evaporated under reduced pressure. The crude material was chromatographed on a silica gel

column using 5% EtOAc in hexanes as eluting solvent and re-purified using 3% EtOAc in hexanes. Compound **1f** was obtained as clear liquid (0.611 g, 90% yield). R_f (SiO_2/20% EtOAc in hexanes) = 0.34; ^{1}H NMR (500 MHz, $CDCl_3$) δ 8.0 (d, 1H, Ar-H, J = 8.4 Hz), 7.45 (t, 1H, Ar-H, J = 8.0 Hz), 7.38–7.34 (m, 3H, Ar-H), 7.31–7.27 (m, 4H, Ar-H), 4.77 (t, 2H, OCH_2, J = 6.8 Hz), 3.20 (t, 2H, CH_2, J = 6.8 Hz); ^{13}C NMR (125 MHz, $CDCl_3$) δ 143.5, 136.6, 129.0, 128.8, 128.0, 127.3, 127.0, 124.7, 120.2, 108.7, 81.0, 34.6; HRMS (ESI/TOF) *m/z* calcd for $C_{14}H_{14}N_3O$ $[M + H]^+$: 240.1131, found 240.1137.

1-(Cinnamyloxy)-1*H*-benzo[*d*][1,2,3]triazole (1g) [5]**:** Synthesized from Bt–OTs (0.580 g, 2.0 mmol), cinnamyl alcohol (0.322 g, 2.4 mmol), and DBU (360 μL, 2.4 mmol) in anhydrous THF (10 mL), over 3 h at room temperature. The volatiles were evaporated and the crude material was chromatographed on a silica gel column using 13% EtOAc/hexanes as eluting solvent. Compound **1g** was obtained as a pale yellow solid (0.417 g, 83% yield). R_f (SiO_2/20% EtOAc in hexanes) = 0.30; ^{1}H NMR (500 MHz, $CDCl_3$) δ 7.99 (d, 1H, Ar-H, J = 8.4 Hz), 7.58 (d, 1H, Ar-H, J = 8.3 Hz), 7.48 (t, 1H, Ar-H, J = 7.6 Hz), 7.36 (t, 1H, Ar-H, J = 7.6 Hz), 7.31–7.27 (m, 5H, Ar-H), 6.63 (d, 1H, =CH, J = 15.8 Hz), 6.44 (dt, 1H, =CH, J = 7.1, 15.8 Hz), 5.19 (d, 2H, OCH_2, J = 7.1 Hz); ^{13}C NMR (125 MHz, $CDCl_3$) δ 143.7, 138.9, 135.6, 128.9, 128.8, 128.2, 127.1, 124.7, 120.7, 120.4, 109.1, 81.3; HRMS (ESI/TOF) *m/z* calcd for $C_{15}H_{14}N_3O$ $[M + H]^+$ 252.1131, found 252.1128.

1-((3-Methylbut-2-en-1-yl)oxy)-1*H*-benzo[*d*][1,2,3]triazole (1h): A mixture of Bt–OTs (2.56 g, 8.84 mmol) and the 3-methyl-2-buten-1-ol (0.74 mL, 7.28 mmol) in THF (15 mL) were cooled to 0 ˚C, and DBU (1.32 mL, 8.80 mmol) was added. The mixture was allowed to warm to room temperature and stirred for 2.5 h. The reaction mixture was diluted with CH_2Cl_2 and washed with water (3x). The organic layer was separated, dried over anhydrous Na_2SO_4, and evaporated

under reduced pressure. The crude material was chromatographed on a silica gel column by elution with 5% EtOAc in hexanes. Compound **1h** was obtained as colorless oil (1.33 g, 74% yield). R_f (SiO_2/1% MeOH in CH_2Cl_2) = 0.60; ^{1}H NMR (500 MHz, $CDCl_3$) δ 7.96 (d, 1H, Ar-H, J = 8.4 Hz), 7.51 (d, 1H, Ar-H, J = 8.1 Hz), 7.46 (t, 1H, Ar-H, J = 7.5 Hz), 7.33 (t, 1H, Ar-H, J = 7.7 Hz), 5.51 (t, 1H, J = 7.4 Hz), 4.99 (d, 2H, J = 8.1 Hz), 1.65 (s, 3H, CH_3), 1.45 (s, 3H, CH_3); ^{13}C NMR (125 MHz, $CDCl_3$) δ 145.2, 143.5, 128.3, 127.8, 124.6, 120.1, 116.4, 109.2, 76.9, 17.92, 17.91; HRMS (ESI/TOF) *m/z* calcd for $C_{11}H_{14}N_3O$ 204.1131, found 204.1120.

1-(Furan-3-ylmethoxy)-1*H*-benzo[*d*][1,2,3]triazole (1i): Synthesized from Bt–OTs (0.145 g, 0.50 mmol), 3-furanmethanol (51 μL, 0.60 mmol), and DBU (89 μL, 0.60 mmol) in anhydrous THF (2.5 mL), over 4 h at room temperature. The reaction mixture was partitioned between EtOAc and water. The organic layer was separated, dried over anhydrous Na_2SO_4, and evaporated under reduced pressure. The crude material was chromatographed on a silica gel column by sequential elution with 15% and 20% EtOAc in hexanes. Compound **1i** was obtained as colorless solid (77.4 mg, 72% yield). R_f (SiO_2/20% EtOAc in hexanes) = 0.26; ^{1}H NMR (500 MHz, $CDCl_3$) δ 7.94 (d, 1H, Ar-H, J = 8.4 Hz), 7.39–7.35 (m, 2H, Ar-H), 7.31–7.27 (m, 3H, Ar-H), 6.43 (s, 1H, Ar-H), 5.42 (s, 2H, OCH_2); ^{13}C NMR (125 MHz, $CDCl_3$) δ 144.3, 143.7, 143.4, 128.3, 128.1, 124.8, 120.2, 118.2, 110.9, 109.0, 73.4; HRMS (ESI/TOF) *m/z* calcd for $C_{11}H_{10}N_3O_2$ $[M + H]^+$ 216.0768, found 216.0770.

1-(Furan-2-ylmethoxy)-1*H*-benzo[*d*][1,2,3]triazole (1j): Synthesized from Bt–OTs (0.578 g, 2.0 mmol), 2-furanmethanol (0.26 mL, 3.0 mmol), and DBU (0.36 mL, 2.4 mmol) in anhydrous THF (10 mL), over 24 h at room temperature. The reaction mixture was partitioned between EtOAc and water. The organic layer was separated, dried over anhydrous Na_2SO_4, and evaporated under reduced pressure. The crude material was chromatographed on a silica gel

column by sequential elution with 15% and 20% EtOAc in hexanes. Compound **1j** was obtained as colorless solid (0.220 g, 51% yield). R_f (SiO_2/20% EtOAc in hexanes) = 0.24; ^{1}H NMR (500 MHz, $CDCl_3$) δ 7.91 (d, 1H, Ar-H, J = 8.1 Hz), 7.38 (d, 1H, Ar-H, J = 1.1 Hz), 7.33 (t, 1H, Ar-H, J = 8.1 Hz), 7.26 (t, 1H, Ar-H, J = 7.8 Hz), 7.15 (d, 1H, Ar-H, J = 8.1 Hz), 6.21 (d, 1H, Ar-H, J = 3.3 Hz), 6.20-6.18 (m, 1H, Ar-H), 5.43 (s, 2H, OCH_2); ^{13}C NMR (125 MHz, $CDCl_3$) δ 147.1, 144.6, 143.2, 128.2, 127.9, 124.5, 120.0, 114.1, 111.1, 108.6, 73.0; HRMS (ESI/TOF) *m/z* calcd for $C_{11}H_{10}N_3O_2$ $[M + H]^+$ 216.0768, found 216.0767.

1-((2,3-Dimethoxybenzyl)oxy)-1*H*-benzo[*d*][1,2,3]triazole (1k): Synthesized from Bt–OTs (1.10 g, 3.80 mmol), 2,3-dimethoxybenzyl alcohol (0.58 g, 3.45 mmol), and DBU (0.77 mL, 5.1 mmol) in anhydrous THF (17 mL), over 2.5 h at room temperature. The reaction mixture was partitioned between EtOAc and water. The organic layer was separated, dried over anhydrous Na_2SO_4, and evaporated under reduced pressure. The crude material was chromatographed on a silica gel column using 20% EtOAc in hexanes as eluting solvent. Compound **1k** was obtained as white solid (0.862 g, 87% yield). R_f (SiO_2/20% EtOAc in hexanes) = 0.29; ^{1}H NMR (500 MHz, $CDCl_3$) δ 7.98 (d, 1H, Ar-H, J = 8.4 Hz), 7.41–7.36 (m, 2H, Ar-H), 7.33 (br t, 1H, Ar-H, J = 6.5 Hz), 7.01–6.95 (m, 2H, Ar-H), 6.89 (dd, 1H, Ar-H, J = 1.6, 7.3 Hz), 5.57 (s, 2H, OCH_2), 3.88 (s, 3H, OCH_3), 3.87 (s, 3H, OCH_3); ^{13}C NMR (125 MHz, $CDCl_3$) δ 152.9, 148.9, 143.5, 128.1, 127.9, 127.1, 124.6, 124.3, 123.7, 120.2, 114.6, 109.1, 77.8, 61.6, 56.1; HRMS (ESI/TOF) *m/z* calcd for $C_{15}H_{16}N_3O_3$ $[M + H]^+$ 286.1186, found 286.1194.

4-((1*H*-Benzo[*d*][1,2,3]triazol-1-yl)oxy)butan-2-ol (1l): Synthesized from Bt–OTs (0.159 g, 0.55 mmol), 1,3-butanediol (45 μL, 0.50 mmol), and DBU (0.11 mL, 0.75 mmol) in anhydrous THF (2.5 mL), over 4 h at room temperature. The volatiles were evaporated and the crude material was chromatographed on a silica gel column using 15% EtOAc in hexanes as eluting

solvent. Compound **1l** was obtained as white solid (50.1 mg, 48% yield). R_f (SiO_2/40% EtOAc in hexanes) = 0.10; ^{1}H NMR (500 MHz, $CDCl_3$) δ 8.01 (d, 1H, Ar-H, J = 8.4 Hz), 7.60 (d, 1H, Ar-H, J = 8.4 Hz), 7.52 (t, 1H, Ar-H, J = 7.6 Hz), 7.39 (t, 1H, Ar-H, J = 7.7 Hz), 4.66–4.76 (m, 2H, OCH_2), 4.24 (br m, 1H, OCH), 2.08 (m, 1H, C<u>H</u>H), 1.95 (m, 1H, CH<u>H</u>), 1.80 (d, 1H, OH, J = 4.3 Hz), 1.33 (d, 3H, CH_3, J = 6.2 Hz); ^{13}C NMR (125 MHz, $CDCl_3$) δ 143.5, 128.2, 127.4, 124.9, 120.2, 108.9, 78.4, 64.6, 37.3, 24.1; HRMS (ESI/TOF) *m/z* calcd for $C_{10}H_{14}N_3O_2$ [M + H]$^+$ 208.1081, found 208.1084.

1-(*sec*-Butoxy)-1*H*-benzo[*d*][1,2,3]triazole (1m): Synthesized from Bt–OTs (0.144 g, 0.50 mmol), 2-butanol (91.7 μL, 1.0 mmol), and DBU (149 μL, 1.0 mmol) in anhydrous THF (3 mL), over 24 h at 60 ˚C. The volatiles were evaporated and the crude material was chromatographed on a silica gel column using 5% EtOAc/hexanes as eluting solvent. Compound **1m** was obtained as a pale yellow oil (50.6 mg, 53% yield). R_f (SiO_2/20% EtOAc in hexanes) = 0.41; ^{1}H NMR (500 MHz, $CDCl_3$) δ 8.0 (d, 1H, Ar-H, J = 8.3 Hz), 7.55 (d, 1H, Ar-H, J = 8.3 Hz), 7.49 (t, 1H, Ar-H, J = 7.6 Hz), 7.36 (t, 1H, Ar-H, J = 8.0 Hz), 4.70 (sext, 1H, OCH, J = 6.2 Hz), 1.93–1.84 (m, 1H, C<u>H</u>H), 1.79–1.70 (m, 1H, CH<u>H</u>), 1.37 (d, 3H, CH_3, J = 5.9 Hz), 1.11 (t, 3H, CH_3, J = 7.3 Hz); ^{13}C NMR (125 MHz, $CDCl_3$) δ 143.6, 128.6, 128.0, 124.6, 120.4, 109.1, 89.0, 27.9, 18.6, 9.7; HRMS (ESI/TOF) *m/z* calcd for $C_{10}H_{14}N_3O$ [M + H]$^+$ 192.1131, found 192.1140.

1-(Prop-2-yn-1-yloxy)-1*H*-benzo[*d*][1,2,3]triazole (1n) [6]**:** Synthesized from Bt–OTs (0.640 g, 2.2 mmol), propargyl alcohol (120 μL, 2.0 mmol), and DBU (360 μL, 2.4 mmol) in anhydrous THF (5 mL), over 3.5 h at room temperature. The reaction mixture was diluted with EtOAc and washed with water. The aqueous layer was back extracted (2x) with EtOAc. The combined organic layer were dried over anhydrous Na_2SO_4 and evaporated. The crude material was chromatographed on a silica gel column by sequential elution with 2%, 4%, 8%, and 15%

acetone/hexanes. Compound **1n** was obtained as a yellow oil (0.140 g, 79% yield). R_f (SiO_2/10% acetone in hexanes, developed 4x) = 0.40; ^{1}H NMR (500 MHz, $CDCl_3$) δ 7.94 (d, 1H, Ar-H, J = 8.3 Hz), 7.63 (d, 1H, Ar-H, J = 8.3 Hz), 7.45 (t, 1H, Ar-H, J = 7.6 Hz), 7.32 (t, 1H, Ar-H, J = 7.8 Hz), 5.13 (d, 2H, OCH_2, J = 2.4 Hz), 2.54 (t, 1H, ≡CH, J = 2.4 Hz); ^{13}C NMR (125 MHz, $CDCl_3$) δ 143.6, 128.5, 128.3, 124.9, 120.4, 109.6, 79.5, 76.1, 67.7; HRMS (ESI/TOF) *m/z* calcd for $C_9H_7N_3O$ $[M + H]^+$ 174.0662, found 174.0668.

1-((4-Nitrobenzyl)oxy)-1*H*-benzo[*d*][1,2,3]triazole (1o) [7]**:** In a 100 mL oven-dried, round-bottomed flask equipped with a stirring bar were placed *p*-nitrobenzyl alcohol (0.612 g, 4.0 mmol) and Bt–OTs, (**3**, 1.735 g, 6.0 mmol) in anhydrous THF (40 mL). The reaction mixture was cooled in an ice bath. To the cold, stirred solution was added DBU (0.65 mL, 4.35 mmol) dropwise over the period of 1.5 h. The reaction mixture was allowed to warm to room temperature and stirred for 6 h. The mixture was partitioned between EtOAc and water. The organic layer was separated, dried over anhydrous Na_2SO_4, and evaporated under reduced pressure. The crude material was chromatographed on a silica gel column using 50% CH_2Cl_2 in hexanes as eluting solvent. Compound **1o** was obtained as light yellow liquid (0.734 g, 68% yield). R_f (SiO_2/20% EtOAc in hexanes) = 0.12; ^{1}H NMR (500 MHz, $CDCl_3$) δ 8.22 (d, 2H, Ar-H, J = 8.5 Hz), 8.00 (d, 1H, Ar-H, J = 8.3 Hz), 7.61 (d, 2H, Ar-H, J = 8.5 Hz), 7.43 (t, 1H, Ar-H, J = 7.6 Hz), 7.36 (t, 1H, Ar-H, J = 8.0 Hz), 7.32 (d, 1H, Ar-H, J = 8.2 Hz), 5.65 (s, 2H, OCH_2); ^{13}C NMR (125 MHz, $CDCl_3$) δ 148.8, 143.5, 140.2, 130.5, 128.5, 127.7, 125.0, 124.2, 120.6, 108.5, 80.7; HRMS (ESI/TOF) *m/z* calcd for $C_{13}H_{11}N_4O_3$ $[M + H]^+$ 271.0826, found: 271.0823.

Phenol tosylate (1p) [8]**:** Synthesized from Bt–OTs (34.7 mg, 0.12 mmol), phenol (9.4 mg, 0.10 mmol), and DBU (22 μL, 0.15 mmol) in anhydrous THF (0.5 mL), over 3 h at room temperature.

The reaction mixture was partitioned between EtOAc and water. The organic layer was separated, dried over anhydrous Na_2SO_4, and evaporated under reduced pressure. The crude material was chromatographed on a silica gel column using 5% EtOAc in hexanes as eluting solvent. Compound **1p** was obtained as white crystalline solid (20.9 mg, 84% yield). R_f (SiO_2/10% EtOAc in hexanes) = 0.18; ^{1}H NMR (500 MHz, $CDCl_3$) δ 7.70 (d, 2H, Ar-H, J = 8.0 Hz), 7.31–7.22 (m, 5H, Ar-H), 6.98 (d, 2H, Ar-H, J = 8.0 Hz) 2.45 (s, 3H, CH_3).

3-Methoxy-3*H*-[1,2,3]triazolo[4,5-*b*]pyridine (2a) [9]**:** Synthesized from At–OTs (0.20 g, 0.69 mmol), methanol (33.5 µL, 0.83 mmol), and DBU (0.12 mL, 0.8 mmol) in anhydrous THF (4 mL), over 24 h at room temperature. The mixture was evaporated under reduced pressure and the crude material was chromatographed on a silica gel column using CH_2Cl_2 followed by 7% acetone in CH_2Cl_2 as eluting solvent. Compound **2a** was obtained as white crystalline solid (76.5 mg, 74% yield). R_f (SiO_2/10% acetone in CH_2Cl_2) = 0.50; mp: 94.5–95.5 ˚C; ^{1}H NMR (500 MHz, $CDCl_3$) δ 8.73 (dd, 1H, Ar-H, J = 1.3, 4.3 Hz), 8.38 (dd, 1H, Ar-H, J = 1.3, 8.3 Hz), 7.41 (dd, 1H, Ar-H, J = 4.4, 8.3 Hz), 4.47 (s, 3H, OCH_3); ^{13}C NMR (125 MHz, $CDCl_3$): δ 151.4, 139.5, 135.4, 129.5, 120.8, 68.4.

3-(Allyloxy)-3*H*-[1,2,3]triazolo[4,5-*b*]pyridine (2b): Synthesized from At–OTs (0.147 g, 0.51 mmol), allyl alcohol (40.0 µL, 0.56 mmol), and DBU (0.12 mL, 0.8 mmol) in anhydrous THF (3 mL), over 24 h at room temperature. The reaction mixture was partitioned between EtOAc and water. The organic layer was separated, dried over anhydrous Na_2SO_4, and evaporated under reduced pressure. The crude material was chromatographed on a silica gel column by gradient elution with 0.5–3% acetone in CH_2Cl_2. Compound **2b** was obtained as slightly yellow, powdery solid (61.1 mg, 69% yield). R_f (SiO_2/5% acetone in CH_2Cl_2) = 0.47; ^{1}H NMR (500 MHz, $CDCl_3$) δ 8.69 (dd, 1H, Ar-H, J = 1.4, 4.4 Hz), 8.33 (dd, 1H, Ar-H, J = 1.4, 8.4 Hz), 7.37

(dd, 1H, Ar-H, *J* = 4.5, 8.4 Hz), 6.17 (ddt, 1H, =CH, *J* = 6.8, 10.3, 17.1 Hz), 5.33–5.28 (m, 2H, =CH), 5.10–5.08 (m, 2H, OCH_2); ^{13}C NMR (125 MHz, $CDCl_3$) δ 151.3, 140.0, 135.0, 130.1, 129.3, 123.6, 120.7, 81.7; HRMS (ESI/TOF) *m/z* calcd for $C_8H_9N_4O$ [M + H]$^+$ 177.0771, found 177.0769.

3-(Benzyloxy)-3*H*-[1,2,3]triazolo[4,5-*b*]pyridine (2c) [10]**:** Synthesized from At–OTs (0.145 g, 0.50 mmol), benzyl alcohol (58 μL, 0.56 mmol), and DBU (0.12 mL, 0.8 mmol) in anhydrous THF (3 mL), over 24 h at room temperature. The reaction mixture was partitioned between EtOAc and water. The organic layer was separated, dried over anhydrous Na_2SO_4, and evaporated under reduced pressure. The crude material was chromatographed on a silica gel column by gradient elution with 5–20% EtOAc in hexanes. Compound **2c** was obtained as grayish solid (91.2 mg, 80% yield). R_f (SiO_2/40% EtOAc in hexanes) = 0.25; ^{1}H NMR (500 MHz, $CDCl_3$) δ 8.64 (d, 1H, Ar-H, *J* = 4.2 Hz), 8.30 (dd, 1H, Ar-H, *J* = 1.2, 8.4 Hz), 7.45 (m, 2H, Ar-H), 7.32 (m, 4H, Ar-H), 5.60 (s, 2H, OCH_2); ^{13}C NMR (125 MHz, $CDCl_3$) δ 151.3, 140.0, 135.1, 132.7, 130.1, 129.8, 129.2, 128.8, 120.7, 82.9; HRMS (ESI/TOF) *m/z* calcd for $C_{12}H_{11}N_4O$ [M + H]$^+$ 227.0927, found 227.0950.

3-(1-Phenylethoxy)-3*H*-[1,2,3]triazolo[4,5-*b*]pyridine (2d): Synthesized from At–OTs (0.146 g, 0.50 mmol), 1-phenylethanol (0.12 mL, 1.0 mmol), and DBU (0.15 mL, 1.0 mmol) in anhydrous THF (3 mL), over 24 h at 60 ˚C. The reaction mixture was partitioned between EtOAc and water. The organic layer was separated, dried over anhydrous Na_2SO_4, and evaporated under reduced pressure. The crude material was chromatographed on a silica gel column by gradient elution with 3–12% EtOAc in hexanes. Compound **2d** was obtained as slightly yellow, viscous oil (76.2 mg, 64% yield). R_f (SiO_2/20% EtOAc in hexanes) = 0.26; ^{1}H NMR (500 MHz, $CDCl_3$) δ 8.63 (dd, 1H, Ar-H, *J* = 1.2, 4.5 Hz), 8.26 (dd, 1H, Ar-H, *J* = 1.3, 8.4

Hz), 7.44 (dd, 1H, Ar-H, J = 4.2, 8.7 Hz), 7.31–7.25 (m, 5H, Ar-H), 5.94 (q, 1H, OCH, J = 6.6 Hz), 1.85 (d, 3H, CH_3, J = 6.6 Hz); ^{13}C NMR (125 MHz, $CDCl_3$) δ 151.2, 140.4, 137.9, 134.9, 129.5, 129.2, 128.7, 127.7, 120.5, 89.1, 20.5; HRMS (ESI/TOF) *m/z* calcd for $C_{13}H_{13}N_4O$ [M + H]$^+$ 241.1084, found 241.1094.

3-Phenethoxy-3*H*-[1,2,3]triazolo[4,5-*b*]pyridine (2e): Synthesized from At–OTs (0.145 g, 0.50 mmol), 2-phenylethanol (66 μL, 0.55 mmol), and DBU (0.12 mL, 0.8 mmol) in anhydrous THF (3 mL), over 24 h at room temperature. The reaction mixture was partitioned between EtOAc and water. The organic layer was separated, dried over anhydrous Na_2SO_4, and evaporated under reduced pressure. The crude material was chromatographed on a silica gel column by gradient elution with 3–15% EtOAc in hexanes. Compound **2e** was obtained as slightly yellow, viscous oil (81.9 mg, 68% yield). R_f (SiO_2/20% EtOAc in hexanes): 0.21; ^1H NMR (500 MHz, $CDCl_3$) δ 8.73 (dd, 1H, Ar-H, J = 1.2 Hz, 4.4 Hz), 8.36 (dd, 1H, Ar-H, J = 1.3, 8.3 Hz), 7.40 (dd, 1H, Ar-H, J = 4.5, 8.4 Hz), 7.34–7.30 (m, 4H, Ar-H), 7.27–7.22 (m, 1H, Ar-H), 4.86 (t, 2H, OCH_2, J = 7.4 Hz), 3.27 (t, 2H, CH_2, J = 7.4 Hz); ^{13}C NMR (125 MHz, $CDCl_3$) δ 151.4, 139.9, 136.3, 135.2, 129.5, 129.1, 128.9, 127.1, 120.9, 81.6, 34.7; HRMS (ESI/TOF) *m/z* calcd for $C_{13}H_{13}N_4O$ [M + H]$^+$ 241.1084, found 241.1085.

3-(Furan-2-ylmethoxy)-3H-[1,2,3]triazolo[4,5-*b*]pyridine (2f): Synthesized from At–OTs (0.145 g, 0.50 mmol), 2-furanmethanol (48 μL, 0.55 mmol), and DBU (0.12 mL, 0.8 mmol) in anhydrous THF (3 mL), over 24 h at room temperature. The reaction mixture was partitioned between EtOAc and water. The organic layer was separated, dried over anhydrous Na_2SO_4, and evaporated under reduced pressure. The crude material was chromatographed on a silica gel column using 5–15% EtOAc in hexanes. Compound **2f** was obtained as greenish viscous oil (72.0 mg, 67% yield). R_f (SiO_2/40% EtOAc in hexanes): 0.21; ^1H NMR (500 MHz, $CDCl_3$) δ

8.63 (d, 1H, Ar-H, *J* = 3.6), 8.31 (d, 1H, Ar-H, *J* = 8.3), 7.38 (t, 1H, Ar-H, *J* = 0.8 Hz), 7.34 (dd, 1H, Ar-H, *J* = 4.3, 8.2 Hz), 6.30 (d, 1H, *J* = 3.2 Hz), 6.22 (m, 1H), 5.53 (s, 2H, OCH_2); ^{13}C NMR (125 MHz, $CDCl_3$) δ 151.4, 146.7, 144.9, 140.1, 135.0, 129.2, 120.7, 114.2, 110.9, 73.5; HRMS (ESI/TOF) *m/z* calcd for $C_{10}H_9N_4O_2$ $[M + H]^+$ 217.0720, found 217.0740.

3-((2,3-Dimethoxybenzyl)oxy)-3*H*-[1,2,3]triazolo[4,5-*b*]pyridine (2g): Synthesized from At–OTs (0.148 g, 0.51 mmol), 2,3-dimethoxybenzyl alcohol (95.0 mg, 0.56 mmol), and DBU (0.12 mL, 0.8 mmol) in anhydrous THF (3 mL), over 24 h at room temperature. The reaction mixture was partitioned between EtOAc and water. The organic layer was separated, dried over anhydrous Na_2SO_4, and evaporated under reduced pressure. The crude material was chromatographed on a silica gel column by gradient elution with 1–4% acetone in CH_2Cl_2. Compound **2g** was obtained as a white powdery solid (97.2 mg, 68% yield). R_f (SiO_2/5% acetone in CH_2Cl_2) = 0.52; ^1H NMR (500 MHz, $CDCl_3$) δ 8.67 (dd, 1H, Ar-H, *J* = 1.3, 4.4 Hz), 8.32 (dd, 1H, Ar-H, *J* = 1.3, 8.3 Hz), 7.35 (dd, 1H, Ar-H, *J* = 4.4, 8.4 Hz), 6.92–6.99 (m, 3H, Ar-H), 5.68 (s, 2H, OCH_2), 3.96 (s, 3H, OCH_3), 3.85 (s, 3H, OCH_3); ^{13}C NMR (125 MHz, $CDCl_3$) δ 153.0, 151.3, 148.9, 140.2, 135.1, 129.2, 127.0, 124.2, 123.4, 120.6, 114.4, 78.0, 61.9, 56.0; HRMS (ESI/TOF) *m/z* calcd for $C_{14}H_{15}N_4O_3$ $[M + H]^+$ 287.1139, found 287.1143.

4-((3*H*-[1,2,3]triazolo[4,5-*b*]pyridin-3-yl)oxy)butan-2-ol (2h): Synthesized from At–OTs (0.148 g, 0.51 mmol), 1,3-butanediol (90 μL, 1.0 mmol), and DBU (0.15 mL, 1.0 mmol) in anhydrous THF (3 mL), over 24 h at 60 ˚C. The reaction mixture was partitioned between EtOAc and water. The organic layer was separated, dried over anhydrous Na_2SO_4, and evaporated under reduced pressure. The crude material was chromatographed on a silica gel column by gradient elution with 1–7% acetone in CH_2Cl_2. Compound **2h** was obtained as colorless, viscous oil (61.4 mg, 59% yield). R_f (SiO_2/10% acetone in CH_2Cl_2) = 0.49; ^1H NMR

(500 MHz, $CDCl_3$) δ 8.71 (dd, 1H, Ar-H, J = 1.2, 4.5 Hz), 8.39 (dd, 1H, Ar-H, J = 1.3, 8.4 Hz), 7.42 (dd, 1H, Ar-H, J = 4.5, 8.4 Hz), 4.83–4.76 (m, 2H, OCH_2), 4.43-4.36 (m, 1H, OCH), 3.18 (s, 1H, OH), 2.17–2.10 (m, 1H, C<u>H</u>H), 1.92–1.85 (m, 1H, CH<u>H</u>), 1.33 (d, 3H, CH_3, J = 6.3 Hz); ^{13}C NMR (125 MHz, $CDCl_3$) δ 151.2, 139.6, 135.4, 129.9, 120.9, 79.5, 63.9, 37.4, 24.2; HRMS (ESI/TOF) *m/z* calcd for $C_9H_{13}N_4O_2$ $[M + H]^+$ 209.1033, found 209.1033.

(3-*sec*-Butoxy)-3*H*-[1,2,3]triazolo[4,5-*b*]pyridine (2i): Synthesized from At–OTs (0.145 g, 0.50 mmol), 2-butanol (92 µL, 1.0 mmol), and DBU (0.15 mL, 1.0 mmol) in anhydrous THF (3 mL), over 24 h at 60 ˚C. The reaction mixture was partitioned between EtOAc and water. The organic layer was separated, dried over anhydrous Na_2SO_4, and evaporated under reduced pressure. The crude material was chromatographed on a silica gel column by gradient elution with 10–30% EtOAc in hexanes. Compound **2i** was obtained as clear oil (32.3 mg, 34% yield). R_f (SiO_2/40% EtOAc in hexanes) = 0.34; ^1H NMR (500 MHz, $CDCl_3$) δ 8.71 (d, 1H, Ar-H, J = 4.1 Hz), 8.36 (d, 1H, Ar-H, J = 8.3 Hz), 7.38 (dd, 1H, Ar-H, J = 4.4, 8.3 Hz), 4.81 (sext, 1H, OCH, J = 6.2 Hz), 1.95–1.87 (m, 1H, C<u>H</u>H), 1.81–1.73 (dt, 1H, CH<u>H</u>, J = 7.1, 14.2 Hz), 1.39 (d, 3H, CH_3, J = 6.3 Hz), 1.10 (t, 3H, CH_3, J = 7.5 Hz); ^{13}C NMR (125 MHz, $CDCl_3$) δ 151.3, 140.7, 135.3, 129.3, 120.7, 89.4, 27.8, 18.4, 9.6; HRMS (ESI/TOF) *m/z* calcd for $C_9H_{13}N_4O$ $[M + H]^+$ 193.1084, found 193.1069.

3-(1*H*-Benzo[*d*][1,2,3]triazol-1-yl)oxy)propan-1-ol (3a): Synthesized from Bt–OTs (0.30 g, 1.04 mmol), 1,3-propanediol (750 µL, 10.4 mmol), and DBU (190 µL, 1.25 mmol) in anhydrous THF (5.2 mL), over 24 h at 60 ˚C. The volatiles were evaporated and the crude was purified on a silica gel column. Initial elution with 40% EtOAc in hexanes led to the isolation of the minor 1,3-bis-benzotriazolyl product and subsequent elution with 50% EtOAc/hexanes led to the isolation of compound **3a** as a pale yellow oil (0.158 g, 79% yield). R_f (SiO_2/60% EtOAc in

hexanes) = 0.17; ^{1}H NMR (500 MHz, $CDCl_3$) δ 7.97 (d, 1H, Ar-H, J = 8.8 Hz), 7.58 (d, 1H, Ar-H, J = 8.3 Hz), 7.48 (t, 1H, Ar-H, J = 7.6 Hz), 7.36 (t, 1H, Ar-H, J = 7.6 Hz), 4.69 (t, 2H, OCH_2, J = 6.1 Hz), 3.96 (t, 2H, OCH_2, J = 6.1 Hz), 2.96 (br, 1H, OH), 2.11 (quint, 2H, CH_2, J = 6.1 Hz); ^{13}C NMR (125 MHz, $CDCl_3$) δ 143.5, 128.3, 127.4, 124.9, 120.2, 108.9, 78.2, 58.7, 31.1; HRMS (ESI/TOF) *m/z* calcd for $C_9H_{12}N_3O_2$ $[M + H]^+$ 194.0924, found 194.0936. The minor bis benzotriazolyl product was obtained as a white solid (20.2 mg, 6% yield). R_f (SiO_2/60% EtOAc in hexanes) = 0.35; ^{1}H NMR (500 MHz, $CDCl_3$) δ 8.04 (d, 2H, Ar-H, J = 8.4 Hz), 7.66 (d, 2H, Ar-H, J = 8.3 Hz), 7.55 (t, 2H, Ar-H, J = 7.6 Hz), 7.42 (t, 2H, Ar-H, J = 7.7 Hz), 4.90 (t, 4H, OCH_2, J = 6.0 Hz), 2.48 (quint, 2H, CH_2, J = 6.0 Hz); ^{13}C NMR (125 MHz, $CDCl_3$) δ 143.7, 128.5, 127.4, 125.0, 120.6, 108.7, 76.5, 27.3; HRMS (ESI/TOF) *m/z* calcd for $C_{15}H_{15}N_6O_2$ $[M + H]^+$ 311.1251, found 311.1246.

3-((3*H*-[1,2,3]Triazolo[4,5-*b*]pyridin-3-yl)oxy)propan-1-ol (3b): Synthesized from At–OTs (72.9 mg, 0.25 mmol), 1,3-propanediol (0.18 mL, 2.5 mmol), and DBU (45 µL, 0.30 mmol) in anhydrous THF (1.25 mL), over 48 h at 60 ˚C. The mixture was evaporated under reduced pressure and the crude product was chromatographed on a silica gel column. Elution with 1% and then 2% MeOH in CH_2Cl_2 yielded an impure 1,3-bis-AzaBt product (9.6 mg). Further elution with 2.5% MeOH in CH_2Cl_2 yielded compound **3b** as a clear oil (36.6 mg, 75% yield). R_f (SiO_2/5% MeOH in CH_2Cl_2) = 0.38; ^{1}H NMR (500 MHz, $CDCl_3$) δ 8.69 (d, 1H, J = 4.4 Hz), 8.37 (dd, 1H, J = 1.5, 8.3 Hz), 7.40 (dd, 1H, J = 4.6, 8.6 Hz), 4.78 (t, 2H, OCH_2, J = 5.8 Hz), 4.02 (t, 2H, OCH_2, J = 5.8 Hz), 3.60 (br s, 1H, OH), 2.10 (quint, 2H, J = 5.8 Hz); ^{13}C NMR (125 MHz, $CDCl_3$): δ 151.2, 139.5, 135.3, 129.8, 120.9, 78.8, 58.1, 30.9; HRMS (ESI/TOF) *m/z* calcd for $C_8H_{11}N_4O_2$ $[M + H]^+$ 195.0877, found 195.0878. A small amount of what appeared to be the

bis 7-azabenzotriazolyl product was also isolated but it was not of adequate purity to allow characterization.

3-(But-3-enyloxy)-1*H*-benzo[*d*][1,2,3,]triazole (4a): Synthesized from Bt–OTs (0.289 g, 1.0 mmol), 3-buten-1-ol (103 μL, 1.2 mmol), and DBU (0.18 mL, 1.2 mmol) in anhydrous THF (5 mL), over 24 hours at room temperature. The reaction mixture was partitioned between EtOAc and water. The organic layer was separated, dried over anhydrous Na_2SO_4, and evaporated under reduced pressure. The crude material was chromatographed on a silica gel column using 10% EtOAc in hexanes as eluting solvent. Compound **4a** was obtained as colorless oil (0.106 g, 56% yield). R_f (SiO_2/20% EtOAc in hexanes) = 0.44; ^{1}H NMR (500 MHz, $CDCl_3$) δ 7.98 (d, 1H, Ar-H, J = 8.8 Hz), 7.55 (d, 1H, Ar-H, J = 8.1 Hz), 7.48 (t, 1H, Ar-H, J = 6.8 Hz), 7.35 (t, 1H, Ar-H, J = 7.7 Hz), 5.92–5.84 (m, 1H, =CH), 5.16 (d, 1H, =CH, J = 17.9 Hz), 5.13 (d, 1H, =CH, J = 10.3 Hz), 4.56 (t, 2H, OCH_2, J = 6.6 Hz), 2.59 (app q, 2H, CH_2, J_{app} = 6.6 Hz); ^{13}C NMR (125 MHz, $CDCl_3$) δ 143.5, 132.7, 128.1, 127.5, 124.8, 120.3, 118.5, 108.8, 79.8, 32.4; HRMS (ESI/TOF) *m/z* calcd for $C_{10}H_{12}N_3O$ $[M + H]^+$ 190.0975, found 190.0984.

3-(But-3-enyloxy)-3*H*-[1,2,3]triazolo[4,5-*b*]pyridine (4b): Synthesized from At–OTs (0.878 g, 3.0 mmol), 3-buten-1-ol (312 μL, 3.6 mmol), and DBU (0.54 mL, 3.6 mmol) in anhydrous THF (15 mL), over 24 h at 60 ˚C. The reaction mixture was partitioned between EtOAc and water. The organic layer was separated, dried over anhydrous Na_2SO_4, and evaporated under reduced pressure. The crude material was chromatographed on a silica gel column by gradient elution with 5–15% EtOAc in hexanes. Compound **4b** was obtained as a clear oil (0.49 g, 86% yield). R_f (SiO_2/20% acetone in hexanes) = 0.27; ^{1}H NMR (500 MHz, $CDCl_3$) δ 8.76 (dd, 1H, Ar-H, J = 1.3, 4.4 Hz), 8.40 (dd, 1H, Ar-H, J = 1.3, 8.4 Hz), 7.44 (dd, 1H, Ar-H, J = 4.5, 8.4 Hz), 5.95 (ddt, 1H, =CH, J = 6.7, 10.3, 17.1 Hz.), 5.26 (dd, 1H, =CH, J = 1.5, 17.2 Hz), 5.19 (dd, 1H, =CH, J =

1.2, 10.3 Hz), 4.72 (t, 2H, OCH_2, J = 6.8 Hz), 2.71 (app q, 2H, CH_2, J_{app} = 6.8 Hz); ^{13}C NMR (125 MHz, $CDCl_3$): δ 151.5, 139.9, 135.3, 132.6, 129.5, 120.9, 118.5, 80.4, 32.6; HRMS (ESI/TOF) *m/z* calcd for $C_9H_{11}N_4O$ $[M + H]^+$ 191.0927, found 191.0919.

(±)-4-(1*H*-Benzo[*d*][1,2,3]triazol-1-yl)oxy)butane-1,2-diol (5a): To a solution of compound **4a** (94.5 mg, 0.5 mmol) in 9:1 THF–H_2O (8.5 mL) was added potassium osmate (8.1 mg, 22 μmol), and NMO (58.5 mg, 0.5 mmol). The mixture was stirred at room temperature for 24 h and then diluted with EtOAc. The mixture was extracted with saturated aqueous $NaHSO_3$, the organic layer was dried over anhydrous Na_2SO_4, and evaporated under reduced pressure. The crude product was chromatographed on a silica gel column using 15% MeOH in CH_2Cl_2 as eluting solvent. Compound **5a** was obtained as brown gummy material (76.3 mg, 68% yield). R_f (SiO_2/50% EtOAc in hexanes) = 0.16; ^{1}H NMR (500 MHz, $CDCl_3$) δ 8.02 (d, 1H, Ar-H, J = 8.3 Hz), 7.63 (d, 1H, Ar-H, J = 8.3 Hz), 7.53 (t, 1H, Ar-H, J = 7.6 Hz), 7.41 (t, 1H, Ar-H, J = 7.6 Hz), 4.80–4.72 (m, 2H), 4.20–4.17 (m, 1H), 3.82 (dd, 1H, CH, J = 2.9, 10.7 Hz), 3.63 (dd, 1H, J = 6.8, 11.2 Hz), 3.01 (br s, 2H, OH), 2.11–2.06 (m, 1H), 2.03–1.97 (m, 1H); ^{13}C NMR (125 MHz, $CDCl_3$) δ 143.6, 128.4, 127.5, 125.0, 120.4, 108.9, 78.0, 68.8, 66.8, 31.7; HRMS (ESI/TOF) *m/z* calcd for $C_{10}H_{14}N_3O_3$ $[M + H]^+$ 224.1030, found 224.1045.

(±)-4-((3*H*-[1,2,3]Triazolo[4,5-*b*]pyridin-3-yl)oxy)butane-1,2-diol (5b): To a solution of compound **4b** (0.238 g, 1.25 mmol) in 9:1 THF/H_2O (8.5 mL) was added potassium osmate (23.0 mg, 62.4 μmol), and NMO (0.177 g, 1.5 mmol). The mixture was stirred at room temperature for 24 h and then diluted with EtOAc. The mixture was extracted with saturated aqueous $NaHSO_3$, the organic layer was dried over anhydrous Na_2SO_4, and evaporated under reduced pressure. The crude material was chromatographed on a silica gel column using 15% MeOH in CH_2Cl_2 as eluting solvent. Compound **5b** was obtained as white powdery solid (161.8 mg, 58%

yield). R_f (SiO_2/10% MeOH in CH_2Cl_2) = 0.37; ^{1}H NMR (500 MHz, $CDCl_3$) δ 8.72 (dd, 1H, Ar-H, J = 1.2, 4.5 Hz), 8.43 (dd, 1H, Ar-H, J = 1.2, 8.4 Hz), 7.45 (dd, 1H, Ar-H, J = 4.5, 8.4 Hz), 4.92–4.87 (m, 1H), 4.79 (td, 1H, J = 3.7, 9.8 Hz), 4.33 (ddt, 1H, J = 3.3, 6.5, 9.7 Hz), 3.81 (dd, 1H, J = 3.4, 11.2 Hz), 3.67 (dd, 1H, J = 6.8, 11.2 Hz), 3.07–2.86 (br s, 2H, OH), 2.13–2.07 (m, 1H), 1.91 (ddt, 1H, J = 4.7, 9.7, 14.7 Hz); ^{13}C NMR (125 MHz, $CDCl_3$): δ 151.3, 139.6, 135.4, 130.1, 120.1, 78.9, 68.4, 66.6 31.6; HRMS (ESI/TOF) *m/z* calcd for $C_9H_{13}N_4O_3$ $[M + H]^+$ 225.0982, found 225.0985.

Products From Displacement Reactions with Nucleophiles

Benzyl nitrile (6) [11]**:** A mixture of benzyloxy benzotriazole **1d** (112.6 mg, 0.50 mmol) and NaCN (49 mg, 1.0 mmol) in anhydrous DMSO (1.25 mL) was stirred at 100 ˚C for 1 h. The mixture was diluted with Et_2O and washed with a water/brine mixture (1:1), followed by water (3–4x). The organic layer was dried over anhydrous Na_2SO_4, filtered, and concentrated under reduced pressure. Compound **6** was obtained as a yellow oil (40.2 mg, 69% yield) without need for additional purification. R_f (SiO_2/20% EtOAc in hexanes) = 0.33; ^{1}H NMR (500 MHz, $CDCl_3$): δ 7.40–7.37 (m, 2H, Ar-H), 7.34–7.32 (m, 3H, Ar-H), 3.76 (s, 2H, CH_2); ^{13}C NMR (125 MHz, $CDCl_3$): δ 130.1, 129.4, 128.3, 128.1, 118.0, 23.8. This compound is commercially available.

Benzyl phenyl ether (7) [12]**:** To a solution of benzyloxy benzotriazole **1d** (112.6 mg, 0.50 mmol) in anhydrous DMSO (1.25 mL), were added phenol (94.2 mg, 1.0 mmol) and Cs_2CO_3 (325.8 mg, 1.0 mmol). The mixture was stirred at 100 ˚C for 3 h. The mixture was then diluted with Et_2O and washed with a water/brine mixture (1:1), followed by water (3–4x). The organic layer was dried over anhydrous Na_2SO_4, filtered, and concentrated under reduced pressure. The crude material was purified on a silica gel column using 3% EtOAc/hexanes as eluting solvent.

Compound **7** was obtained as a white solid (42.6 mg, 46% yield). R_f (SiO_2/30% EtOAc in hexanes) = 0.56; ^{1}H NMR (500 MHz, $CDCl_3$): δ 7.49–7.47 (m, 2H, Ar-H), 7.44–7.41 (m, 2H, Ar-H), 7.38–7.32 (m, 3H, Ar-H), 7.03–6.98 (m, 3H, Ar-H), 5.10 (s, 2H, OCH_2); ^{13}C NMR (125 MHz, $CDCl_3$): δ 159.0, 137.3, 129.7, 128.8, 128.1, 127.7, 121.1, 115.1, 70.1. This compound is commercially available.

(1-Azidoethyl)benzene (8) [13]**:** A mixture of 1-phenylethoxy benzotriazole **1e** (119.6 mg, 0.50 mmol) and NaN_3 (65 mg, 1.0 mmol) in anhydrous DMSO (1.25 mL) was stirred at 100 ˚C for 30 h. The mixture was diluted with Et_2O and washed with a water/brine mixture (1:1), followed by water (3–4x). The organic layer was dried over anhydrous Na_2SO_4, filtered, and concentrated under reduced pressure. Compound **8** was obtained as a yellow oil (43.5 mg, 59% yield) without need for additional purification. R_f (SiO_2/30% EtOAc in hexanes) = 0.38; ^{1}H NMR (500 MHz, $CDCl_3$): δ 7.38 (m, 2H, Ar-H), 7.32 (m, 3H, Ar-H), 4.62 (q, 1H, CH, J = 6.8 Hz), 1.54 (d, 3H, CH_3, J = 6.8 Hz); ^{13}C NMR (125 MHz, $CDCl_3$): δ 141.1, 128.9, 128.3, 126.6, 61.3, 21.8.

1-(1-Phenylethyl)-1*H*-benzo[*d*][1,2,3]triazole (9a) [14,15] **and 2-(1-phenylethyl)-2*H*-benzo[*d*][1,2,3]triazole (9b)** [15]**:** To a solution of 1-phenylethoxy benzotriazole **1e** (119.6 mg, 0.50 mmol) in anhydrous DMSO (1.25 mL), were added benzotriazole (119 mg, 1.0 mmol) and Cs_2CO_3 (325.8 mg, 1.0 mmol). The mixture was stirred at 100 ˚C for 30 h. The mixture was then diluted with Et_2O and washed with a water/brine mixture (1:1), followed by water (3–4x). The organic layer was dried over anhydrous Na_2SO_4, filtered, and concentrated under reduced pressure. The crude material was purified on a silica gel column. Initial elution with 3% EtOAc in hexanes led to the isolation of a less polar, minor component, and further elution with 6% EtOAc in hexanes led to the isolation of the major component. The major component, isomer **9a** [14,15], was obtained as a pale yellow liquid (53.2 mg, 48% yield). R_f (SiO_2/30% EtOAc in

hexanes) = 0.35; ^{1}H NMR (500 MHz, $CDCl_3$): δ 8.06–8.05 (m, 1H, Ar-H), 7.35–7.24 (m, 8H, Ar-H), 6.04 (q, 1H, CH, J = 7.1 Hz), 2.17 (d, 3H, CH_3, J = 7.1 Hz); ^{13}C NMR (125 MHz, $CDCl_3$): δ 146.6, 140.3, 132.6, 129.0, 128.4, 127.2, 126.4, 123.9, 120.1, 110.3, 59.2, 21.3. The minor component, isomer **9b** [15], was obtained as a pale yellow solid (37.3 mg, 33% yield). R_f (SiO_2/30% EtOAc in hexanes) = 0.50; ^{1}H NMR (500 MHz, $CDCl_3$): δ 7.90–7.87 (m, 2H, Ar-H), 7.43–7.28 (m, 7H, Ar-H), 6.16 (q, 1H, CH, J = 6.8 Hz), 2.15 (d, 3H, CH_3, J = 6.8 Hz); ^{13}C NMR (125 MHz, $CDCl_3$) δ 144.3, 140.4, 128.9, 128.5, 126.8, 126.4, 118.4, 66.3, 21.6.

(Furan-2-yl)acetonitrile (10) [16]**:** A mixture of 2-furanyloxy benzotriazole **1j** (107.5 mg, 0.50 mmol) and NaCN (49.0 mg, 1.0 mmol) in anhydrous DMSO (1.25 mL) was stirred at 100 ˚C for 1 h. The reaction mixture was diluted with Et_2O and washed with a water/brine mixture (1:1), followed by water. The organic layer was dried over anhydrous Na_2SO_4, filtered, and concentrated under reduced pressure. The crude material was purified on a silica gel column using 5% EtOAc/hexanes as eluting solvent. Compound **10** was obtained as yellow oil (26.4 mg, 49% yield); R_f (SiO_2/20% EtOAc in hexanes) = 0.73; ^{1}H NMR (500 MHz, $CDCl_3$) δ 7.40 (s, 1H, Ar-H), 6.37 (t, 1H, Ar-H, J = 2.4 Hz), 6.34–6.33 (m, 1H, Ar-H), 3.77 (s, 2H, CH_2); ^{13}C NMR (125 MHz, $CDCl_3$) δ 143.3, 143.1, 115.7, 111.1, 108.7, 17.7.

2-(Azidomethyl)furan (11) [17]**:** A mixture of 2-furanyloxy benzotriazole **1j** (107.5 mg, 0.50 mmol) and NaN_3 (49.0 mg, 1.0 mmol) in DMSO-d_6 (1.25 mL) was stirred at 100 ˚C for 1 h. ^{1}HNMR indicated this reaction to be complete and the resonances of the product were comparable to those of the pure product obtained using a reported literature procedure [17]. R_f (SiO_2/20% EtOAc in hexanes) = 0.73; ^{1}H NMR (500 MHz, DMSO-d_6) for a pure reference sample: δ 7.68 (s, 1H, Ar-H), 6.49 (d, 1H, Ar-H, J = 2.9 Hz), 6.46 (s, 1H, Ar-H), 4.44 (s, 2H, CH_2); ^{1}H NMR (500 MHz, DMSO-d_6) for the reaction mixture: δ 7.68 (s, 1H, Ar-H), 7.56 (d,

1H, Ar-$H_{benzotriazolyl}$, J = 8.3 Hz), 7.51 (d, 1H, Ar-$H_{benzotriazolyl}$, J = 7.8 Hz), 7.04 (d, 2H, Ar-$H_{benzotriazolyl}$), 6.48 (s, 1H, Ar-H), 6.44 (s, 1H, Ar-H), 4.43 (s, 2H, CH_2). This compound is commercially available.

2-(Phenoxymethyl)furan (12) [18]**:** To a solution of 2-furanyloxy benzotriazole **1j** (107.5 mg, 0.50 mmol) in anhydrous DMSO (1.25 mL), were added phenol (94.2 mg, 1.0 mmol) and Cs_2CO_3 (325.8 mg, 1.0 mmol). The mixture was stirred at 100 ˚C for 3 h. The mixture was then diluted with Et_2O and washed with a water/brine mixture (1:1), followed by water. The organic layer was dried over anhydrous Na_2SO_4, filtered, and concentrated under reduced pressure. The crude material was purified on a silica gel column using 10% EtOAc/hexanes as eluting solvent. Compound **12** was obtained as colorless oil (58.4 mg, 67% yield). R_f (SiO_2/20% EtOAc in hexanes) = 0.52; ^{1}H NMR (500 MHz, $CDCl_3$) δ 7.45 (d, 1H, Ar-H, J = 1.8 Hz), 7.30 (t, 2H, Ar-H, J = 7.7 Hz), 7.00–6.96 (m, 3H, Ar-H), 6.43 (d, 1H, Ar-H, J = 2.9 Hz), 6.39-6.38 (m, 1H, Ar-H), 5.01 (s, 2H, OCH_2); ^{13}C NMR (125 MHz, $CDCl_3$) δ 158.5, 150.5, 143.3, 129.7, 121.4, 115.1, 110.7, 110.1, 62.6. HRMS (ESI/TOF) *m/z* calcd for $C_{11}H_{11}O_2$ $[M + H]^+$ 175.0754, found 175.0758.

1-(Furan-2-ylmethyl)-1*H*-benzo[*d*][1,2,3]triazole (13a) and 2-(furan-2-ylmethyl)-2*H*-benzo[*d*][1,2,3]triazole (13b) [19]**:** To a solution of 3-furanyloxy benzotriazole **1j** (53.8 mg, 0.25 mmol) in anhydrous DMSO (0.62 mL), were added benzotriazole (59.5 mg, 0.5 mmol) and Cs_2CO_3 (162.9 mg, 0.5 mmol). The mixture was stirred at 100 ˚C for 3h. The reaction mixture was then diluted with Et_2O and washed with a water/brine mixture (1:1), followed by water. The organic layer was dried over anhydrous Na_2SO_4, filtered, and concentrated under reduced pressure. The crude material was purified on a silica gel column using EtOAc/hexanes as eluting solvent. Elution with 2%, 4%, 6%, and 8% EtOAc in hexanes led to the isolation of a less polar,

minor component. Further elution with 10% and 12% EtOAc in hexanes led to the isolation of the major component. The major component, isomer **13a**, was obtained as colorless solid (35.2 mg, 71% yield). R_f (SiO_2/20% EtOAc in hexanes) = 0.19; ^{1}H NMR (500 MHz, $CDCl_3$) δ 8.05 (d, 1H, Ar-H, J = 8.4 Hz), 7.55 (d, 1H, Ar-H, J = 8.5 Hz), 7.47 (t, 1H, Ar-H, J = 7.3 Hz), 7.38 (s, 1H), 7.36 (t, 1H, Ar-H, J = 7.6 Hz), 6.44 (d, 1H, Ar-H, J = 2.6 Hz), 6.35 (d, 1H, Ar-H, J = 1.1 Hz), 5.82 (s, 2H, CH_2); ^{13}C NMR (125 MHz, $CDCl_3$) δ 147.9, 146.3, 143.4, 132.9, 127.7, 124.1, 120.1, 110.9, 110.0, 109.9, 45.2; HRMS (ESI/TOF) m/z calcd for $C_{11}H_{10}N_3O$ $[M + H]^+$ 200.0818, found 200.0817. The minor component, isomer **13b**, was obtained as colorless solid (4.2 mg, 8% yield). R_f (SiO_2/20% EtOAc in hexanes) = 0.28; ^{1}H NMR (500 MHz, $CDCl_3$) δ 7.87 (dd, 2H, Ar-H, J = 2.9, 6.6 Hz), 7.43 (d, 1H, Ar-H J = 0.7 Hz), 7.37 (dd, 2H, Ar-H, J = 2.9, 6.6 Hz), 6.56 (d, 1H, Ar-H, J = 3.3 Hz), 6.38 (m, 1H, Ar-H), 5.88 (s, 2H, CH_2). Adequate material was not available for a ^{13}C NMR.

2-(2,3-Dimethoxyphenyl)acetonitrile (14) [20]**:** A mixture of (2,3-dimethoxybenzyl)oxy benzotriazole **1k** (0.057 g, 0.2 mmol) and NaCN (19.6 mg, 0.4 mmol) in anhydrous DMSO (0.5 mL) was stirred at 100 ˚C for for 1 h. The reaction mixture was diluted with Et_2O and washed with a water/brine mixture (1:1), followed by water (3x). The organic layer was dried over anhydrous Na_2SO_4, filtered, and concentrated under reduced pressure. The crude material was chromatographed on a silica gel column using 10% EtOAc in hexanes as eluting solvent. Compound **14** was obtained as clear liquid (0.025 g, 70% yield). R_f (SiO_2/20% EtOAc in hexanes) = 0.27; ^{1}H NMR (500 MHz, $CDCl_3$) δ 7.05 (t, 1H, Ar-H, J = 7.9 Hz), 6.95 (d, 1H, Ar-H, J = 7.7 Hz), 6.91 (d, 1H, Ar-H, J = 8.2 Hz), 3.91 (s, 3H, CH_3), 3.87 (s, 3H, CH_3), 3.71 (s, 2H, CH_2); ^{13}C NMR (125 MHz, $CDCl_3$) δ 152.9, 146.9, 124.5, 124.3, 121.1, 118.3, 112.9, 60.7, 56.0,

18.7; HRMS (ESI/TOF) *m/z* calcd for $C_{10}H_{11}NO_2Na$ $[M + Na]^+$ 200.0682, found 200.0684. This compound is commercially available.

1-(Azidomethyl)-2,3-dimethoxybenzene (15): A mixture of (2,3-dimethoxybenzyl)oxy benzotriazole **1k** (0.143 g, 0.5 mmol) and NaN_3 (65.0 mg, 1.0 mmol) was stirred in anhydrous DMSO (1.25 mL) at 100 ˚C for 2.5 h. The reaction mixture was diluted with Et_2O and washed with a water/brine mixture (1:1), followed by water (3x). The organic layer was dried over anhydrous Na_2SO_4, filtered, and concentrated under reduced pressure. The crude material was chromatographed on a silica gel column using 10% EtOAc in hexanes as eluting solvent. Compound **15** was obtained as clear liquid (86.0 g, 89% yield). R_f (SiO_2/20% EtOAc in hexanes) = 0.5; ^1H NMR (500 MHz, $CDCl_3$) δ 7.06 (t, 1H, Ar-H, J = 7.9 Hz), 6.92 (d, 1H, Ar-H, J = 8.1 Hz), 6.90 (d, 1H, Ar-H, J = 7.8 Hz), 4.37 (s, 2H, CH_2), 3.90 (s, 3H, CH_3), 3.87 (s, 3H, CH_3); ^{13}C NMR (125 MHz, $CDCl_3$) δ 152.8, 147.5, 129.3, 124.2, 121.8, 113.0, 61.1, 55.8, 49.9; HRMS (EI^+/TOF) calcd for $C_9H_{11}N_3O_2$ $[M]^+$ 193.0846, found 193.0849.

1,2-Dimethoxy-3-(phenoxymethyl)benzene (16): To a solution of (2,3-dimethoxybenzyl)oxy benzotriazole **1k** (0.143 g, 0.5 mmol) in anhydrous DMSO (1.25 mL), were added phenol (94.0 mg, 1.0 mmol) and Cs_2CO_3 (32.6 mg, 1.0 mmol). The mixture was stirred at 100 ˚C for 2.5 h. The reaction mixture was diluted with Et_2O and washed with a water/brine mixture (1:1), followed by water (3x). The organic layer was dried over anhydrous Na_2SO_4, filtered, and concentrated under reduced pressure. The crude material was chromatographed on a silica gel column using 10% EtOAc in hexanes as eluting solvent. Compound **16** was obtained as clear liquid (66.0 g, 54% yield). R_f (SiO_2/20% EtOAc in hexanes) = 0.47; ^1H NMR (500 MHz, $CDCl_3$) δ 7.34 (t, 2H, Ar-H, J = 7.6 Hz), 7.14 (m, 2H, Ar-H), 7.07 (d, 2H, Ar-H, J = 8.6 Hz), 7.02 (t, 1H, Ar-H, J = 7.3 Hz), 6.95 (dd, 1H, Ar-H, J = 3.5, 5.9 Hz), 5.19 (s, 2H, OCH_2), 3.95 (s,

3H, CH_3). 3.93 (s, 3H, CH_3); ^{13}C NMR (125 MHz, $CDCl_3$): δ 152.9, 147.0, 128.6, 127.5, 124.6, 124.0, 121.3, 120.0, 112.9, 110.2, 61.1, 56.0, 46.8; HRMS (ESI/TOF) *m/z* calcd for $C_{15}H_{16}O_3Na$ $[M + Na]^+$ 267.0992, found 267.1003.

1-(2,3-Dimethoxybenzyl)-1*H*-benzo[*d*][1,2,3]triazole (17a) and 2-(2,3-dimethoxybenzyl)-2*H*-benzo[*d*][1,2,3]triazole (17b): To a solution of (2,3-dimethoxybenzyl)oxy benzotriazole **1k** (57.0 mg, 0.2 mmol), in anhydrous DMSO (0.5 mL), were added benzotriazole (47.6 mg, 0.4 mmol) and Cs_2CO_3 (0.130 g, 0.4 mmol). The mixture was stirred at 100 ˚C 4 h. The reaction mixture was then diluted with Et_2O and washed with a water/brine mixture (1:1), followed by water (3x). The organic layer was dried over anhydrous Na_2SO_4, filtered, and concentrated under reduced pressure. The crude material was chromatographed on a silica gel column using 10% EtOAc in hexanes as eluting solvent. The minor component eluted first followed by the major component. The major component, isomer **17a**, was obtained as a obtained as clear, gummy material (39.0 mg, 69% yield). R_f (20% EtOAc in hexanes) = 0.14; ^{1}H NMR (500 MHz, $CDCl_3$) δ 8.04 (d, 1H, Ar-H, J = 8.3 Hz), 7.52 (d, 1H, Ar-H, J = 8.3 Hz), 7.41 (t, 1H, Ar-H, J = 7.6 Hz), 7.33 (t, 1H, Ar-H, J = 7.6 Hz), 6.97 (t, 1H, Ar-H, J = 8.0 Hz), 6.87 (d, 1H, Ar-H, J = 8.1 Hz), 6.73 (d, 1H, Ar-H, J = 7.7 Hz), 5.87 (s, 2H, CH_2), 3.86 (s, 3H, CH_3), 3.82 (s, 3H, CH_3); ^{13}C NMR (125 MHz, $CDCl_3$) δ 152.9, 147.0, 146.3, 133.1, 128.6, 127.4, 124.5, 124.0, 121.2, 120.0, 112.9, 110.2, 61.1, 56.0, 46.8; HRMS (ESI/TOF) calcd for $C_{15}H_{16}N_3O_2$ $[M + H]^+$ 270.1237, found 270.1238. The minor component, isomer **17b**, was also obtained as a clear, gummy material (12.0 mg, 20%). R_f (SiO_2/20% EtOAc in hexanes) = 0.27; ^{1}H NMR (500 MHz, $CDCl_3$) δ 7.86 (dd, 2H, Ar-H, J = 3.1, 6.5 Hz), 7.36 (dd, 2H, Ar-H, J = 3.1, 6.5 Hz), 7.02 (t, 1H, Ar-H, J = 8.0 Hz), 6.90 (d, 1H, Ar-H, J = 8.0 Hz), 6.83 (d, 1H, Ar-H, J = 7.7 Hz), 5.95 (s, 2H, CH_2), 3.87 (s, 3H, CH_3), 3.84 (s, 3H, CH_3); ^{13}C NMR (125 MHz, $CDCl_3$) δ 152.9, 147.3, 144.7, 128.8,

126.4, 124.4, 121.7, 118.3, 113.1, 61.1, 56.0, 55.1; HRMS (ESI/TOF) calcd for $C_{15}H_{16}N_3O_2$ [M + H]$^+$ 270.1237, found 270.1236.

Reaction of 1-phenethoxy-1*H*-benzo[*d*][1,2,3]triazole (1f) with NaN_3 leading to (2-azidoethyl)benzene: To a stirred solution of 1-phenethoxy benzotriazole **1f** (0.036g, 0.15 mmol) in DMSO-d_6 (0.4 mL) in a dry vial, was added NaN_3 (20 mg, 0.3 mmol). The mixture was stirred at 100 ˚C for 28 h and an aliquot was assessed by ^{1}H NMR. Because a significant amount of product was observed, the reaction mixture was diluted with Et_2O and washed with a water/brine solution (1:1). The organic layer was dried over anhydrous Na_2SO_4 and evaporated under a stream of nitrogen gas. Due to volatility of the product (68 ˚C at 0.5 mm [21]) the product mixture was briefly dried with a water aspirator. The mass balance was 29.4 mg and ^{1}H NMR analysis of this material indicated it to be a 7.3:1 mixture of (2-azidoethyl)benzene [22] and precursor **1f**.

Synthesis of γ,δ-Unsaturated Cycloalkanones

2-Cinnamylcyclohexan-1-one (18) [23]**:** To a solution of cinnamyloxy benzotriazole **1g** (125.6 mg, 0.50 mmol) in DMSO (2 mL), $Pd(PPh_3)_4$ (28.8 mg, 25 µmol, 5 mol%) was added, and the mixture was stirred at room temperature for 5 min. Then cyclohexanone (155 µL, 1.50 mmol) and pyrrolidine (12 µL, 0.15 mmol, 30 mol%) were added. The reaction vial was flushed with nitrogen gas and the mixture was stirred at room temperature for 2 h. The mixture was then diluted with EtOAc and washed with water followed by brine. The organic layer was dried with anhydrous Na_2SO_4 and evaporated. The crude material was chromatographed on a silica gel column by sequential elution with hexanes followed by 1%, 2%, and 2.5% EtOAc in hexanes. Compound **18** was obtained as a pale yellow oil (62.9 mg, 59% yield). R_f (SiO_2/5% EtOAc in hexanes) = 0.21; ^{1}H NMR (500 MHz, $CDCl_3$) δ 7.34–7.27 (m, 4H, Ar-H), 7.21–7.18 (m, 1H, Ar-

H), 6.39 (d, 1H, =CH, *J* = 15.8 Hz), 6.23–6.17 (ddd, 1H, =CH, J = 6.8, 8.3, 15.1 Hz), 2.70–2.65 (m, 1H, CH), 2.46–2.39 (m, 2H, CH_2), 2.36–2.30 (m, 1H, CH_2), 2.21–2.13 (m, 2H, CH_2), 2.10–2.05 (m, 1H, CH_2), 1.92–1.84 (m, 1H, CH_2), 1.73–1.62 (m, 2H, CH_2), 1.46–1.39 (m, 1H, CH_2); ^{13}C NMR (125 MHz, $CDCl_3$): δ 212.7, 137.7, 131.8, 128.7, 128.6, 127.2, 126.2, 50.9, 42.3, 33.8, 33.2, 28.1, 25.3.

***Syn*-4-(*t*-butyl)-2-cinnamylcyclohexan-1-one (20a)** [24] **and *anti*-4-(*t*-butyl)-2-cinnamylcyclohexan-1-one (20b):** To a solution of cinnamyloxy benzotriazole **1g** (125.6 mg, 0.50 mmol) in DMSO (2 mL), $Pd(PPh_3)_4$ (28.8 mg, 25 μmol, 5 mol%) was added, and the mixture was stirred at room temperature for 5 min. Then *t*-butylcyclohexanone (231 mg, 1.50 mmol) and pyrrolidine (12 μL, 0.15 mmol, 30 mol%) were added. The reaction vial was flushed with nitrogen gas and the mixture was stirred at room temperature for 2 h. The reaction mixture was diluted with EtOAc and was washed with water followed by brine. The organic layer was dried over anhydrous Na_2SO_4 and evaporated. The crude material was chromatographed on a silica gel column by sequential elution with hexanes, 1% (2 x 100 mL), and 2% EtOAc in hexanes. *Syn*-**20a** and *anti*-**20b** were obtained as a clear oil (113.4 mg, 84% yield) and were an inseparable mixture of diastereomers. In this mixture *syn*-**20a** was the major isomer. However, during the chromatographic purification a few fractions of pure *syn*-**20a** (early eluting) and a few fractions containing *anti*-**20b** (late eluting) were obtained. These fractions were used to characterize the two diastereomers. *Syn*-**20a** (major isomer): R_f (SiO_2/5% EtOAc in hexanes, developed twice) = 0.46; ^{1}H NMR (500 MHz, $CDCl_3$) δ 7.34 (m, 2H, Ar-H), 7.29 (m, 2H, Ar-H), 7.20 (m, 1H, Ar-H), 6.43 (d, 1H, =CH, *J* = 15.8 Hz), 6.24 (dt, 1H, =CH, *J* = 7.5, 15.4 Hz), 2.69 (m, 1H, H1'a), 2.43 (m, 2H, $H2_{ax}$, $H6_{eq}$), 2.32 (dt, 1H, $H6_{ax}$, *J* = 6.0, 13.8 Hz), 2.19 (m, 1H, $H3_{eq}$), 2.12 (m, 2H, $H5_{eq}$, H1'b), 1.59 (tt, 1H, $H4_{ax,}$ *J* = 2.8, 12.4 Hz), 1.47 (app dq, 1H, $H5_{ax}$, *J* =

4.7, 12.6 Hz), 1.21 (app q, 1H, $H3_{ax}$, J = 12.8 Hz), 0.91 (s, 9H, *t*-Bu); ^{13}C NMR (125 MHz, $CDCl_3$) δ 212.9, 137.8, 131.7, 128.7, 128.6, 127.1, 126.2, 50.1, 47.3, 41.8, 35.0, 33.3, 32.7, 28.9, 27.9; HRMS (ESI/TOF, for the mixture of *syn*-**20a** and *anti*-**20b**) *m/z* calcd for $C_{19}H_{27}O$ $[M + H]^+$ 271.2056, found 271.2057. *Anti*-**20b** (minor isomer): R_f (SiO_2/5% EtOAc in hexanes, developed twice) = 0.40; ^{1}H NMR (500 MHz, $CDCl_3$): δ 7.30 (m, 4H, Ar-H), 7.20 (t, 1H, Ar-H, J = 7.3 Hz), 6.42 (d, 1H, =CH, J = 16.1 Hz), 6.09 (m, 1H, =CH), 2.56 (m, 2H), 2.40 (m, 3H), 2.02 (app quint d, 1H, J = 2.9, 15.1 Hz), 1.90 (m, 1H), 1.70–1.59 (m, 2H), 1.50 (app dq, 1H, J = 4.9, 12.7 Hz), 0.91 (s, 9H, *t*-Bu); ^{13}C NMR (125 MHz, $CDCl_3$): δ 212.9, 137.8, 131.7, 128.7, 128.6, 127.1, 126.2, 50.2, 47.3, 41.8, 35.0, 33.3, 32.7, 30.4, 27.8; HRMS (ESI/TOF) *m/z* calcd for $C_{19}H_{27}O$ $[M + H]^+$ 271.2056, found 271.2068.

Conditions for the $^{31}P\{^1H\}$ NMR experiments.

In an oven-dried NMR tube were placed BOP (13.2 mg, 30.0 μmol, 1 molar equiv) in distilled THF (0.5 mL) at –78 °C. The tube was transferred to the NMR probe maintained at –30 °C and a spectrum was acquired. The tube was removed and placed at –78 °C, 2-phenylethanol (3.6 μL, 30.0 μmol, 1 molar equiv) was added, and another spectrum was obtained at –30 °C. The tube was again removed, placed at –78 °C, and DBU (4.5 μL, 30.0 μmol, 1 molar equiv) was added. A spectrum was obtained every five minutes for 50 min at –30 °C (total acquisition time for each spectrum was about 4 min). The NMR tube was then left at room temperature and a spectrum was obtained after 24 h.

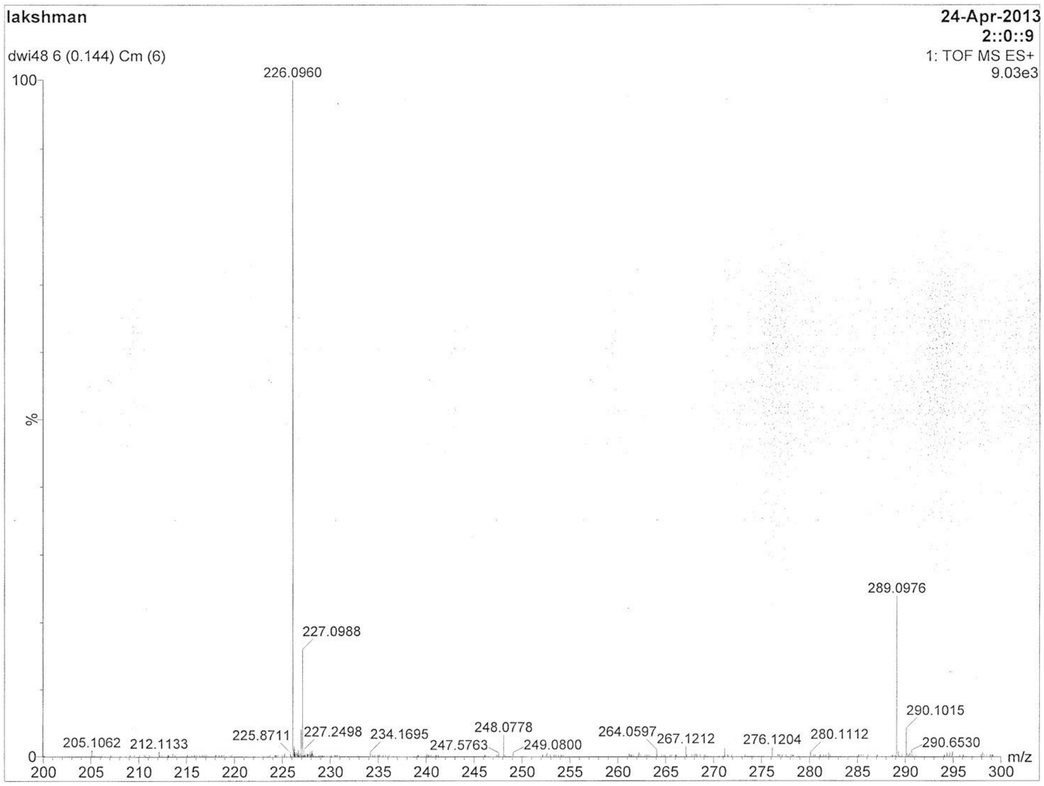

Figure 1: Mass spectrum of the product obtained from the reaction of BOP with $PhCH_2OH$.

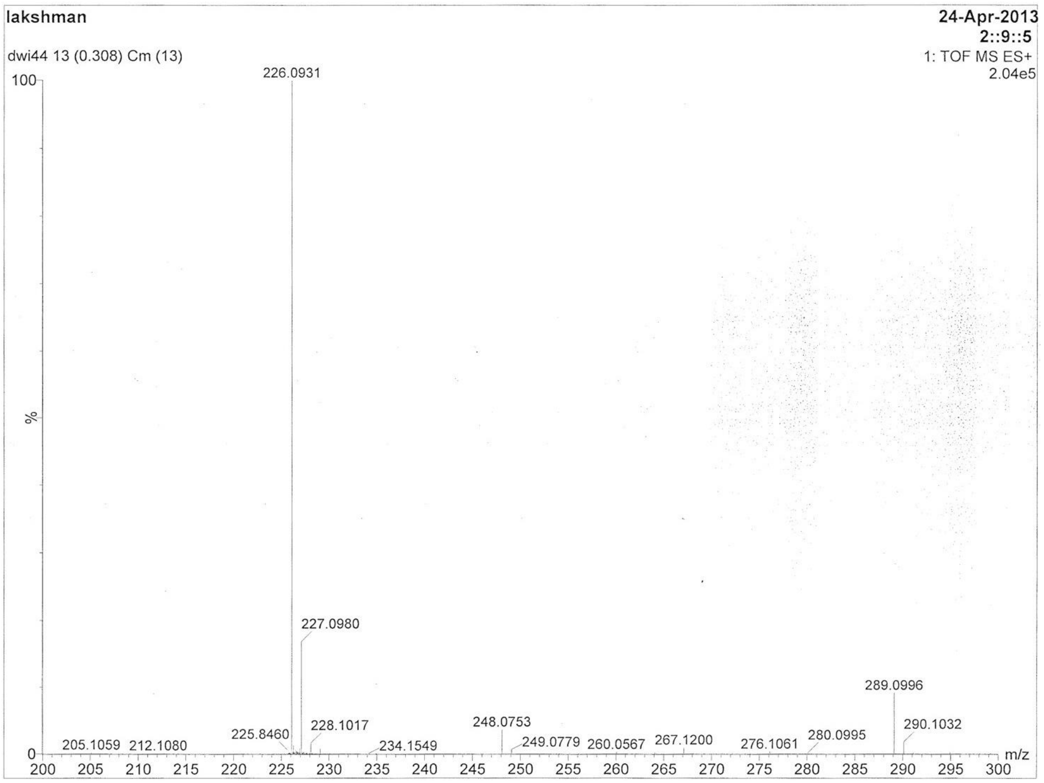

Figure 2: Mass spectrum of the product obtained from the reaction of BOP with $PhCH_2[^{18}O]H$.

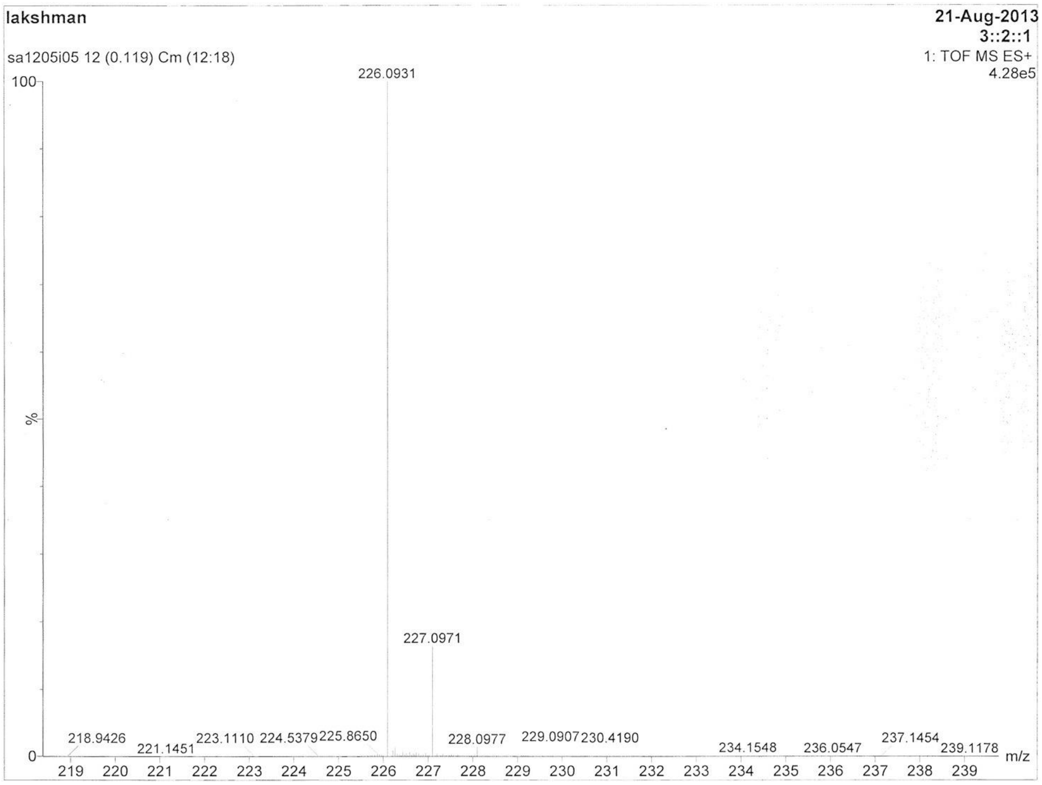

Figure 3: Mass spectrum of the product obtained from the reaction of Bt–OTs with $PhCH_2[^{18}O]H$.

References

1. 1-Methoxy-1*H*-benzo[*d*][1,2,3]triazole **(1a):** Feld, W. A.; Paessun, R. J.; Servé, M. P. *J. Macromol. Sci.-Chem.* **1981**, *A15*, 891–896; Servé, M. P.; Seybold, P. G.; Feld, W. A.; Chao, M. A. *J. Heterocycl. Chem.* **1976**, *13*, 509–512; Brady, O. L.; Reynolds, C. V. *J. Chem. Soc.* **1928**, 193–202.
2. 1-Ethoxy-1*H*-benzo[*d*][1,2,3]triazole **(1b):** Calvino, R.; Mortarini, V.; Serafino, A. *Farmco* **1980**, *35*, 240–247.
3. 1-(Allyloxy)-1*H*-benzo[*d*][1,2,3]triazole **(1c):** Goreshnik, E. A.; Mys'kiv, M. G. *Russ. J. Coord. Chem.* **2005**, *31*, 341–346.
4. 1-(Benzyloxy)-1*H*-benzo[*d*][1,2,3]triazole **(1d):** (a) Dehli, J. R.; Bolm, C. *Synthesis* **2005**, 1058–1060.
5. 1-(Cinnamyloxy)-1*H*-benzo[*d*][1,2,3]triazole **(1g):** Sharma, K. N.; Anteunis, M. J. O. *B. Soc. Chim. Belg.* **1987**, *96*, 467–472.
6. 1-(Prop-2-yn-1-yloxy)-1*H*-benzo[*d*][1,2,3]triazole **(1n):** Bai, S.-Q.; Leelasubcharoen, S.; Chen, X.; Koh, L. L.; Zuo, J.-L.; Hor, T. S. A. *Cryst. Growth Des.* **2010**, *10*, 1715–1720.
7. 1-((4-Nitrobenzyl)oxy)-1*H*-benzo[*d*][1,2,3]triazole **(1o):** Górska, A.; Chomicz, L.; Żebrowska, J.; Myjak, P.; Augustynowicz-Kopeć, E.; Zwolska, Z.; Piekarczyk, J.; Rebandel, H.; Kazimierczuk, Z. *Z. Naturforsch.* **2006**, *61b*, 101–107.
8. Phenol tosylate **(1p):** Tang, Z.-Y.; Hu, Q.-S. *J. Am. Chem. Soc.* **2004**, *126*, 3058–3059.
9. 3-Methoxy-3*H*-[1,2,3]triazolo[4,5-*b*]pyridine **(2a):** Carpino, L. A.; Imazumi, H.; Foxman, B. M.; Vela, M. J.; Henklein, P.; El-Faham, A.; Klose, J.; Bienert, M. *Org. Lett.* **2000**, *2*, 2253–2256.

10. 3-(Benzyloxy)-3*H*-[1,2,3]triazolo[4,5-*b*]pyridine **(2c):** Tran, T. P.; Mullins, P. B.; am Ende, C. W.; Pettersson, M. *Org. Lett.* **2013**, *15*, 642–645.

11. Benzyl nitrile **(6):** Shimojo, H.; Moriyama, K.; Togo, H. *Synthesis* **2013**, *45*, 2155–2164.

12. Benzyl phenyl ether **(7):** Kobayashi, T.; Komatsu, T.; Kamiya, M.; Campos, C.; González-Gaitán, M.; Terai, T.; Hanaoka, K.; Nagano, T.; Urano, Y. *J. Am. Chem. Soc.* **2012**, *134*, 11153–11160.

13. (1-Azidoethyl)benzene **(8):** Kitamura, M.; Yano, M.; Tashiro, N.; Miyagawa, S.; Sando, M.; Okauchi, T. *Eur. J. Org. Chem.* **2011**, 458–462; Hassner, A.; Fibiger, R.; Andisik, D. *J. Org. Chem.* **1984**, *49*, 4237–4244.

14. 1-(1-Phenylethyl)-1*H*-benzo[*d*][1,2,3]triazole **(9a):** Xue, Q.; Xie, J.; Li, H.; Cheng, Y.; Zhu, C. *Chem. Commun.* **2013**, *49*, 3700–3702.

15. 1-(1-Phenylethyl)-1*H*-benzo[*d*][1,2,3]triazole **(9a)** and 2-(1-Phenylethyl)-2*H*-benzo[*d*][1,2,3]triazole **(9b):** Yan, W.; Liao, T.; Tuguldur, O.; Zhong, C.; Petersen, J. L.; Shi, X. *Chem. Asian J.* **2011**, *6*, 2720–2724.

16. (Furan-2-yl)acetonitrile **(10):** Divald, S.; Chun, M. C.; Joullie, M. M. *J. Org. Chem.* **1976**, *41*, 2835–2846.

17. 2-(Azidomethyl)furan **(11):** Rogers, S. A.; Melander, C. *Angew. Chem., Int. Ed.* **2008**, *47*, 5229–5231.

18. 2-(Phenoxymethyl)furan **(12):** Quach, T. D.; Batey, R. A. *Org. Lett.*, **2003**, *5*, 1381–1384.

19. 1-(Furan-2-ylmethyl)-1*H*-benzo[*d*][1,2,3]triazole **(13a)** and 2-(Furan-2-ylmethyl)-2*H*-benzo[*d*][1,2,3]triazole **(13b):** Katritzky, A. R.; Zhang, G.-F.; Pernak, J.; Fan, W.-Q. *Heterocycles* **1993**, *36*, 1253–1262.

20. 2-(2,3-Dimethoxyphenyl)acetonitrile **(14):** Detterbeck, R.; Hesse, M. *Helv. Chim. Acta* **2003**, *86*, 343–360.

21. Boyer, J. H.; Hamer, J. *J. Am. Chem. Soc.* **1955**, *77*, 951–954.

22. Benati, L.; Bencivenni, G.; Leardini, R.; Nanni, D.; Minozzi, M.; Spagnolo, P.; Scialpi, R.; Zanardi, G. *Org. Lett.* **2006**, *8*, 2499–2502.

23. 2-Cinnamylcyclohexan-1-one **(18):** Chen, J.-P.; Peng, Q.; Lei, B.-L.; Hou, X.-L.; Wu, Y.-D. *J. Am. Chem. Soc.* **2011**, *133*, 14180–14183.

24. *Syn*-4-(*t*-butyl)-2-cinnamylcyclohexan-1-one **(20a):** Usui, I.; Schmidt, S.; Breit, B. *Org. Lett.* **2009**, *11*, 1453–1456.
