# Supplementary material for: Facile synthesis of 1-alkoxy-1H-benzo- and 7-azabenzotriazoles from peptide coupling agents, mechanistic studies, and synthetic applications
Source: Beilstein J Org Chem. 2014 Aug 19;10:1919–32. doi: 10.3762/bjoc.10.200 (PMC4168895; doi:10.3762/bjoc.10.200)

Supporting Information 2

for

Facile synthesis of 1-alkoxy-1*H*-benzo- and 7-azabenzotriazoles from peptide coupling agents, mechanistic studies, and synthetic applications

Mahesh K. Lakshman*, Manish K. Singh, Mukesh Kumar, Raghu Ram Chamala, Vijayender R. Yedulla, Domenick Wagner, Evan Leung, Lijia Yang, Asha Matin, and Sadia Ahmad

Address: Department of Chemistry, The City College and The City University of New York, 160 Convent Avenue, New York, NY 10031, USA

Email: Mahesh K. Lakshman* - lakshman@sci.ccny.cuny.edu

*Corresponding author

NMR Spectra

Table of Contents

Information	Page
500 MHz ^{1}H NMR spectrum of **1a** in $CDCl_3$	S5
125 MHz ^{13}C NMR spectrum of **1a** in $CDCl_3$	S6
500 MHz ^{1}H NMR spectrum of **1b** in $CDCl_3$	S7
125 MHz ^{13}C NMR spectrum of **1b** in $CDCl_3$	S8
500 MHz ^{1}H NMR spectrum of **1c** in $CDCl_3$	S9
125 MHz ^{13}C NMR spectrum of **1c** in $CDCl_3$	S10
500 MHz ^{1}H NMR spectrum of **1d** in $CDCl_3$	S11
125 MHz ^{13}C NMR spectrum of **1d** in $CDCl_3$	S12
500 MHz ^{1}H NMR spectrum of **1e** in $CDCl_3$	S13
125 MHz ^{13}C NMR spectrum of **1e** in $CDCl_3$	S14
500 MHz ^{1}H NMR spectrum of **1f** in $CDCl_3$	S15
125 MHz ^{13}C NMR spectrum of **1f** in $CDCl_3$	S16
500 MHz ^{1}H NMR spectrum of **1g** in $CDCl_3$	S17
125 MHz ^{13}C NMR spectrum of **1g** in $CDCl_3$	S18
500 MHz ^{1}H NMR spectrum of **1h** in $CDCl_3$	S19

125 MHz ^{13}C NMR spectrum of **1h** in $CDCl_3$	S20
500 MHz ^{1}H NMR spectrum of **1i** in $CDCl_3$	S21
125 MHz ^{13}C NMR spectrum of **1i** in $CDCl_3$	S22
500 MHz ^{1}H NMR spectrum of **1j** in $CDCl_3$	S23
125 MHz ^{13}C NMR spectrum of **1j** in $CDCl_3$	S24
500 MHz ^{1}H NMR spectrum of **1k** in $CDCl_3$	S25
125 MHz ^{13}C NMR spectrum of **1k** in $CDCl_3$	S26
500 MHz ^{1}H NMR spectrum of **1l** in $CDCl_3$	S27
125 MHz ^{13}C NMR spectrum of **1l** in $CDCl_3$	S28
500 MHz ^{1}H NMR spectrum of **1m** in $CDCl_3$	S29
Expanded 500 MHz ^{1}H NMR spectrum of **1m** in $CDCl_3$	S30
125 MHz ^{13}C NMR spectrum of **1m** in $CDCl_3$	S31
500 MHz ^{1}H NMR spectrum of **1n** in $CDCl_3$	S32
125 MHz ^{13}C NMR spectrum of **1n** in $CDCl_3$	S33
500 MHz ^{1}H NMR spectrum of **1o** in $CDCl_3$	S34
125 MHz ^{13}C NMR spectrum of **1o** in $CDCl_3$	S35
500 MHz ^{1}H NMR spectrum of **1p** in $CDCl_3$	S36
500 MHz ^{1}H NMR spectrum of **2a** in $CDCl_3$	S37
125 MHz ^{13}C NMR spectrum of **2a** in $CDCl_3$	S38
500 MHz ^{1}H NMR spectrum of **2b** in $CDCl_3$	S39
125 MHz ^{13}C NMR spectrum of **2b** in $CDCl_3$	S40
500 MHz ^{1}H NMR spectrum of **2c** in $CDCl_3$	S41
125 MHz ^{13}C NMR spectrum of **2c** in $CDCl_3$	S42
500 MHz ^{1}H NMR spectrum of **2d** in $CDCl_3$	S43
125 MHz ^{13}C NMR spectrum of **2d** in $CDCl_3$	S44
500 MHz ^{1}H NMR spectrum of **2e** in $CDCl_3$	S45
125 MHz ^{13}C NMR spectrum of **2e** in $CDCl_3$	S46
500 MHz ^{1}H NMR spectrum of **2f** in $CDCl_3$	S47
125 MHz ^{13}C NMR spectrum of **2f** in $CDCl_3$	S48
500 MHz ^{1}H NMR spectrum of **2g** in $CDCl_3$	S49
125 MHz ^{13}C NMR spectrum of **2g** in $CDCl_3$	S50
500 MHz ^{1}H NMR spectrum of **2h** in $CDCl_3$	S51
Expanded 500 MHz ^{1}H NMR spectrum of **2h** in $CDCl_3$	S52
125 MHz ^{13}C NMR spectrum of **2h** in $CDCl_3$	S53
500 MHz ^{1}H NMR spectrum of **2i** in $CDCl_3$	S54
Expanded 500 MHz ^{1}H NMR spectrum of **2i** in $CDCl_3$	S55
125 MHz ^{13}C NMR spectrum of **2i** in $CDCl_3$	S56
500 MHz ^{1}H NMR spectrum of **2j** in $CDCl_3$	S57

125 MHz ^{13}C NMR spectrum of **2j** in $CDCl_3$	S58
500 MHz ^{1}H NMR spectrum of **3a** in $CDCl_3$	S59
Expanded 500 MHz ^{1}H NMR spectrum of **3a** in $CDCl_3$	S60
125 MHz ^{13}C NMR spectrum of **3a** in $CDCl_3$	S61
500 MHz ^{1}H NMR spectrum of **3a’** in $CDCl_3$	S62
125 MHz ^{13}C NMR spectrum of **3a’** in $CDCl_3$	S63
500 MHz ^{1}H NMR spectrum of **3b** in $CDCl_3$	S64
125 MHz ^{13}C NMR spectrum of **3b** in $CDCl_3$	S65
500 MHz ^{1}H NMR spectrum of **4a** in $CDCl_3$	S66
125 MHz ^{13}C NMR spectrum of **4a** in $CDCl_3$	S67
500 MHz ^{1}H NMR spectrum of **4b** in $CDCl_3$	S68
Expanded 500 MHz ^{1}H NMR spectrum of **4b** in $CDCl_3$	S69
125 MHz ^{13}C NMR spectrum of **4b** in $CDCl_3$	S70
500 MHz ^{1}H NMR spectrum of **5a** in $CDCl_3$	S71
125 MHz ^{13}C NMR spectrum of **5a** in $CDCl_3$	S72
500 MHz ^{1}H NMR spectrum of **5b** in $CDCl_3$	S73
Expanded 500 MHz ^{1}H NMR spectrum of **5b** in $CDCl_3$	S74
125 MHz ^{13}C NMR spectrum of **5b** in $CDCl_3$	S75
500 MHz ^{1}H NMR spectrum of **6** in $CDCl_3$	S76
125 MHz ^{13}C NMR spectrum of **6** in $CDCl_3$	S77
500 MHz ^{1}H NMR spectrum of **7** in $CDCl_3$	S78
125 MHz ^{13}C NMR spectrum of **7** in $CDCl_3$	S79
500 MHz ^{1}H NMR spectrum of **8** in $CDCl_3$	S80
125 MHz ^{13}C NMR spectrum of **8** in $CDCl_3$	S81
500 MHz ^{1}H NMR spectrum of **9a** in $CDCl_3$	S82
125 MHz ^{13}C NMR spectrum of **9a** in $CDCl_3$	S83
500 MHz ^{1}H NMR spectrum of **9b** in $CDCl_3$	S84
125 MHz ^{13}C NMR spectrum of **9b** in $CDCl_3$	S85
500 MHz ^{1}H NMR spectrum of **10** in $CDCl_3$	S86
125 MHz ^{13}C NMR spectrum of **10** in $CDCl_3$	S87
500 MHz ^{1}H NMR spectrum of a pure sample of 2-(azidomethyl)furan in DMSO-d_6	S88
500 MHz ^{1}H NMR spectrum in DMSO-d_6 of the reaction mixture containing **11**	S89
500 MHz ^{1}H NMR spectrum of **12** in $CDCl_3$	S90
125 MHz ^{13}C NMR spectrum of **12** in $CDCl_3$	S91
500 MHz ^{1}H NMR spectrum of **13a** in $CDCl_3$	S92
125 MHz ^{13}C NMR spectrum of **13a** in $CDCl_3$	S93
500 MHz ^{1}H NMR spectrum of **13b** in $CDCl_3$	S94

500 MHz ^{1}H NMR spectrum of **14** in $CDCl_3$	S95
125 MHz ^{13}C NMR spectrum of **14** in $CDCl_3$	S96
500 MHz ^{1}H NMR spectrum of **15** in $CDCl_3$	S97
125 MHz ^{13}C NMR spectrum of **15** in $CDCl_3$	S98
500 MHz ^{1}H NMR spectrum of **16** in $CDCl_3$	S99
125 MHz ^{13}C NMR spectrum of **16** in $CDCl_3$	S100
500 MHz ^{1}H NMR spectrum of **17a** in $CDCl_3$	S101
125 MHz ^{13}C NMR spectrum of **17a** in $CDCl_3$	S102
500 MHz ^{1}H NMR spectrum of **17b** in $CDCl_3$	S103
125 MHz ^{13}C NMR spectrum of **17b** in $CDCl_3$	S104
500 MHz ^{1}H NMR spectrum in $CDCl_3$ of the mixture containing a 7.3:1 ratio of 2-(azidoethyl)benzene/benzotriazolyl ether **1f**	S105
500 MHz ^{1}H NMR spectrum of **18** in $CDCl_3$	S106
Expanded 500 MHz ^{1}H NMR spectrum of **18** in $CDCl_3$	S107
125 MHz ^{13}C NMR spectrum of **18** in $CDCl_3$	S108
500 MHz ^{1}H NMR spectrum of **19** in $CDCl_3$	S109
Expanded 500 MHz ^{1}H NMR spectrum of **19** in $CDCl_3$	S110
125 MHz ^{13}C NMR spectrum of **19** in $CDCl_3$	S111
500 MHz ^{1}H NMR spectrum of **20a,b** in $CDCl_3$	S112
Expanded 500 MHz ^{1}H NMR spectrum of **20a,b** in $CDCl_3$	S113
500 MHz ^{1}H NMR spectrum of **20a** in $CDCl_3$	S114
Expanded 500 MHz ^{1}H NMR spectrum of **20a** in $CDCl_3$	S115
125 MHz ^{13}C NMR spectrum of **20a** in $CDCl_3$	S116
500 MHz ^{1}H NMR spectrum of **20b** in $CDCl_3$	S117
Expanded 500 MHz ^{1}H NMR spectrum of **20b** in $CDCl_3$	S118
125 MHz ^{13}C NMR spectrum of **20b** in $CDCl_3$	S119
500 MHz gCOSY spectrum of **20a** in $CDCl_3$	S120
500 MHz NOESY spectrum of **20a** in $CDCl_3$	S121
500 MHz gHMQC spectrum of **20a** in $CDCl_3$	S122

DW-1205-I-HNMR-CDCl3-10-methoxy

Pulse Sequence: s2pul

Solvent: CDCl3
Temp. 25.0 C / 298.1 K
Operator: mkl
File: 1205-DW-01-CDCl3-HNMR-0010-methoxy
INOVA-500 "riga"

Pulse 38.6 degrees
Acq. time 1.892 sec
Width 8000.0 Hz
16 repetitions
OBSERVE H1, 499.7707095 MHz
DATA PROCESSING
FT size 32768
Total time 0 min, 30 sec

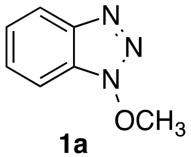

1a

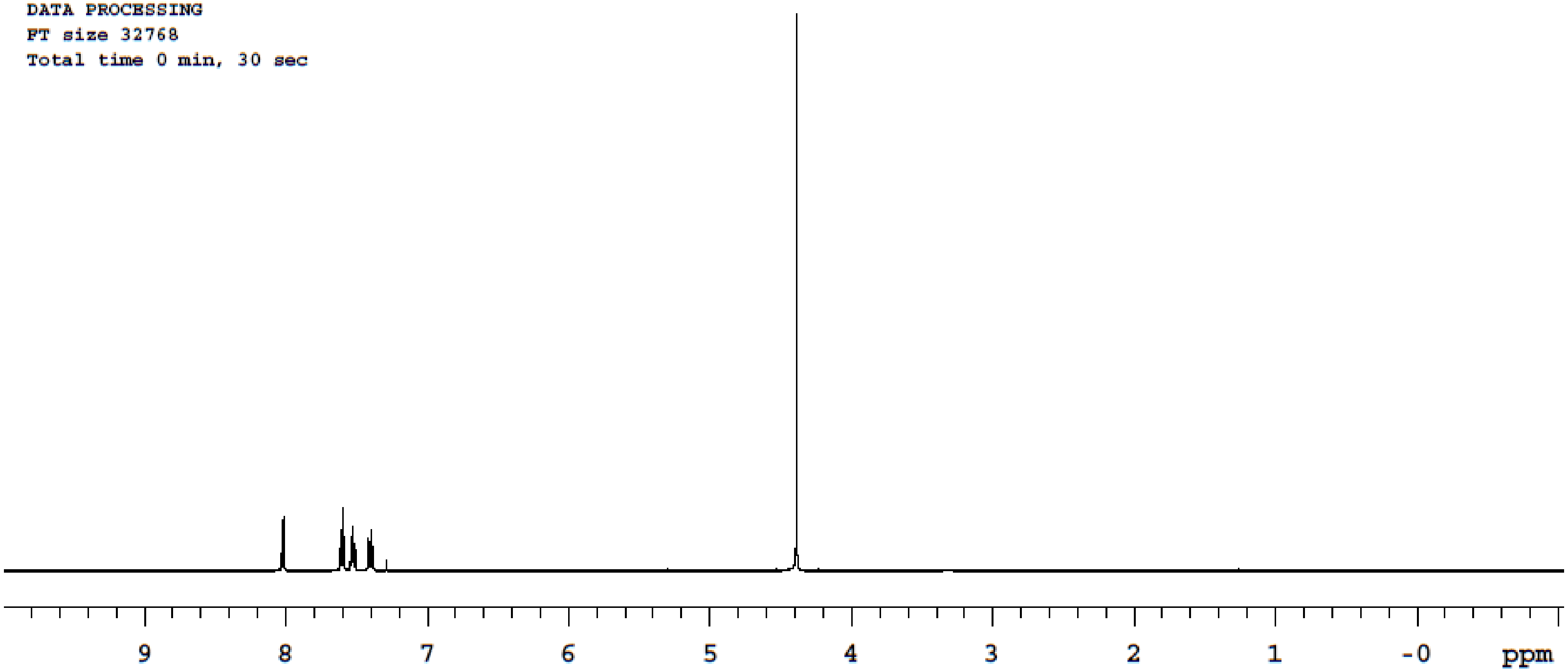

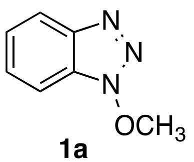

1a

DW-1205-I-CNMR-CDCl3-10-methoxybt

Pulse Sequence: s2pul

Solvent: CDCl3
Ambient temperature
Operator: mkl
File: 1205-DW-01-CDCl3-CNMR-0001-methoxybt
INOVA-500 "riga"

Pulse 45.0 degrees
Acq. time 1.300 sec
Width 25000.0 Hz
384 repetitions
OBSERVE C13, 125.6674149 MHz
DECOUPLE H1, 499.7730084 MHz
Power 39 dB
continuously on
WALTZ-16 modulated
DATA PROCESSING
Line broadening 2.0 Hz
Gauss apodization 0.600 sec
FT size 65536
Total time 21 min, 49 sec

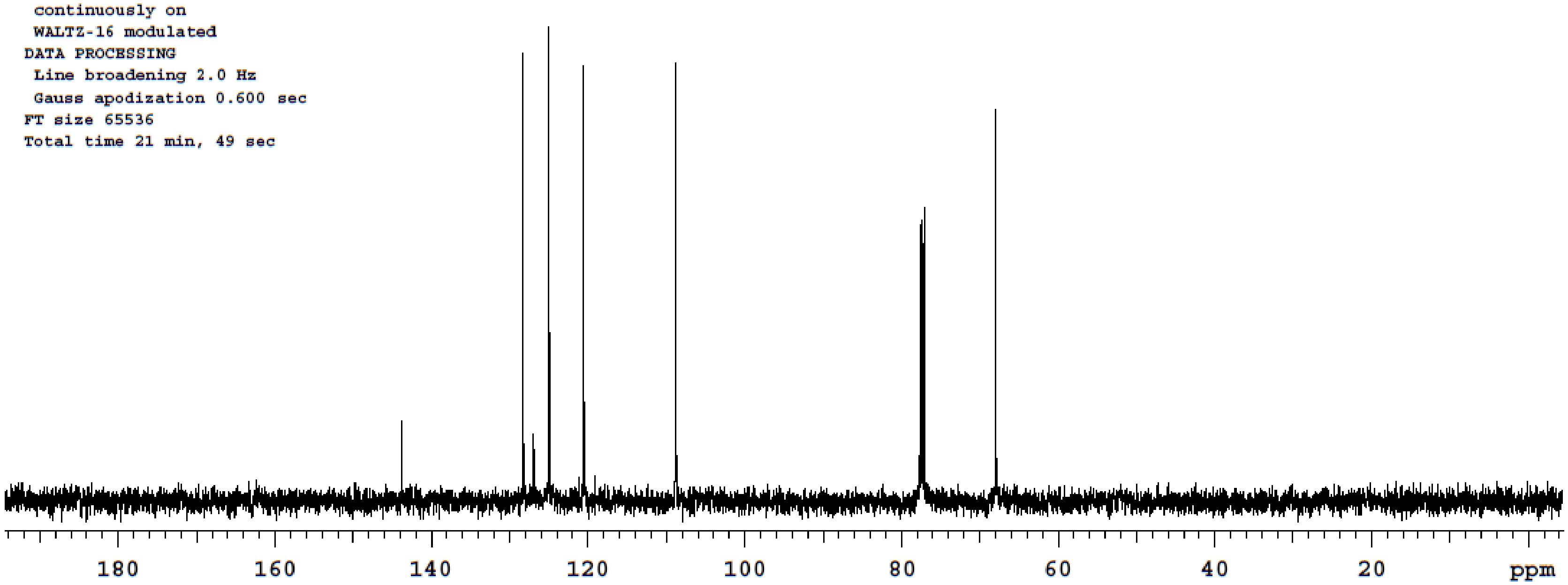

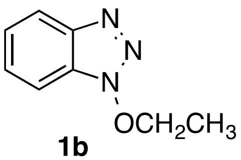

1b

MKS-1205-08-53-CDCl3-CC

Pulse Sequence: s2pul

Solvent: cdcl3
Temp. 25.0 C / 298.1 K
Operator: mkl
File: MKS-1205-08-53-CDCl3-CC
INOVA-500 "riga"

Pulse 45.0 degrees
Acq. time 1.892 sec
Width 8000.0 Hz
52 repetitions
OBSERVE H1, 499.7707212 MHz
DATA PROCESSING
FT size 32768
Total time 1 hr, 4 min, 52 sec

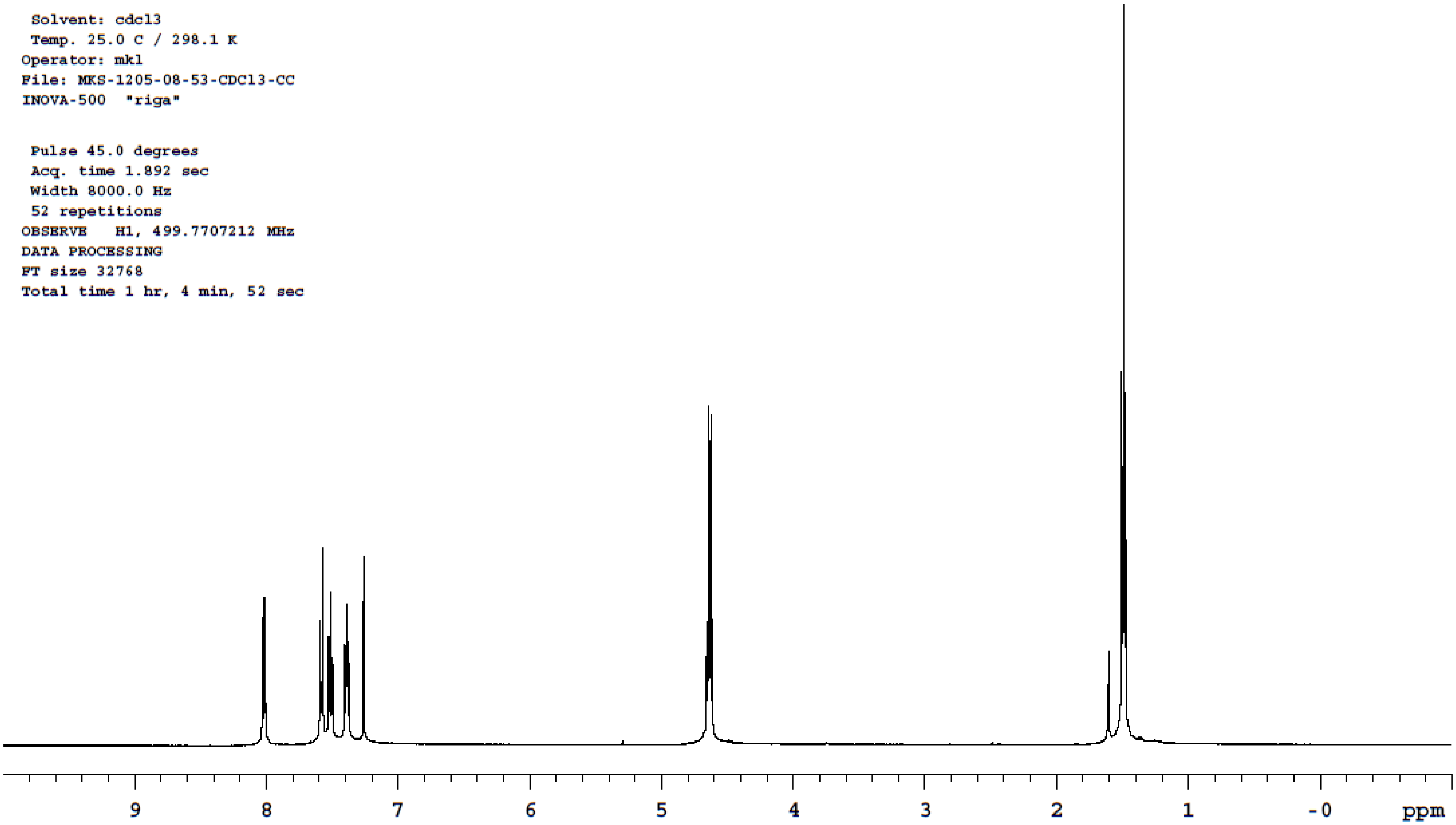

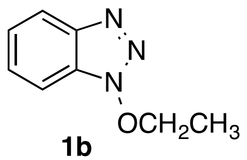

1b

MKS-1205-08-53-CDCl3-13C-CC

Pulse Sequence: s2pul

Solvent: CDCl3
Temp. 25.0 C / 298.1 K
Operator: mkl
File: MKS-1205-08-53-CDCl3-13C-CC
INOVA-500 "riga"

Relax. delay 3.000 sec
Pulse 45.0 degrees
Acq. time 1.300 sec
Width 25000.0 Hz
52 repetitions
OBSERVE C13, 125.6674382 MHz
DECOUPLE H1, 499.7730084 MHz
Power 39 dB
continuously on
WALTZ-16 modulated
DATA PROCESSING
Line broadening 0.2 Hz
FT size 65536
Total time 2 hr, 27 min, 5 sec

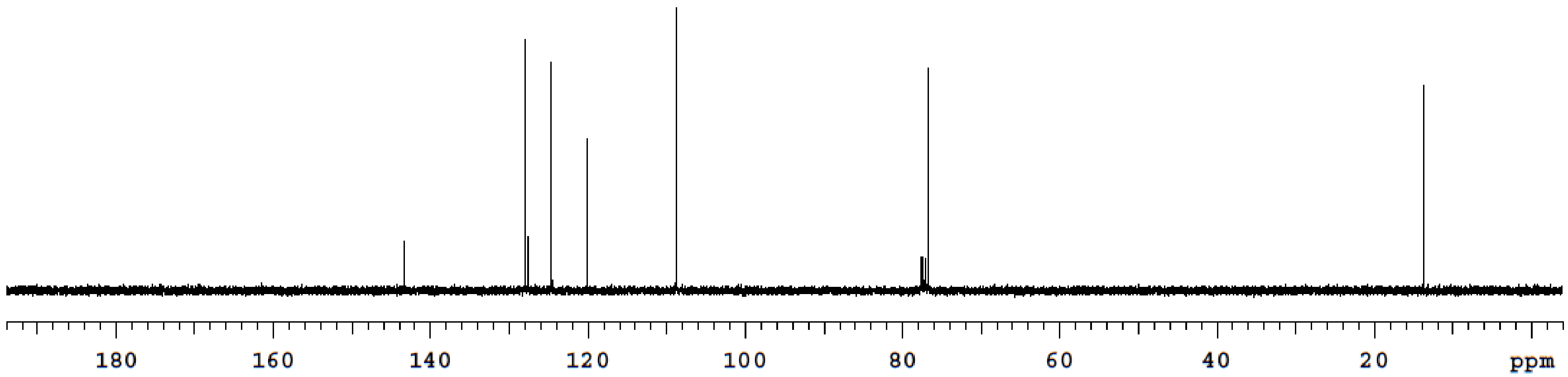

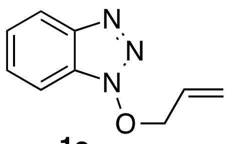

1c

MKS-1205-13-AllylBtEther-CDCl3-CC

Pulse Sequence: s2pul

Solvent: cdcl3
Temp. 25.0 C / 298.1 K
Operator: mkl
File: MKS-1205-13-AllylBtEther-CDCl3-CC
INOVA-500 "riga"

Pulse 45.0 degrees
Acq. time 1.892 sec
Width 8000.0 Hz
64 repetitions
OBSERVE H1, 499.7707217 MHz
DATA PROCESSING
Line broadening 0.2 Hz
FT size 32768
Total time 1 hr, 3 min, 21 sec

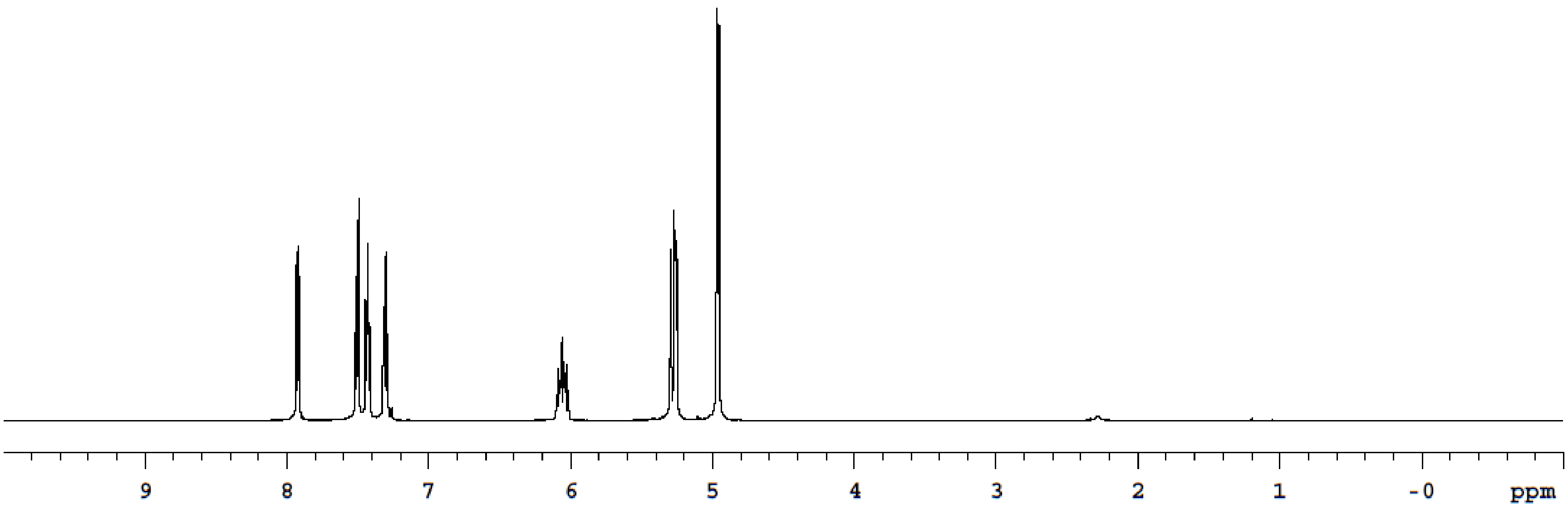

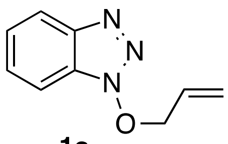

1c

MKS-1205-13-AllylBtEther-13C-CDCl3-CC

Pulse Sequence: s2pul
Solvent: CDCl3
Temp. 25.0 C / 298.1 K
Operator: mkl
File: MKS-1205-13-AllylBtEther-13C-CDCl3-CC
INOVA-500 "riga"

Relax. delay 3.000 sec
Pulse 45.0 degrees
Acq. time 1.300 sec
Width 25000.0 Hz
64 repetitions
OBSERVE C13, 125.6674385 MHz
DECOUPLE H1, 499.7730084 MHz
Power 39 dB
continuously on
WALTZ-16 modulated
DATA PROCESSING
Line broadening 0.2 Hz
FT size 65536
Total time 2 hr, 27 min, 5 sec

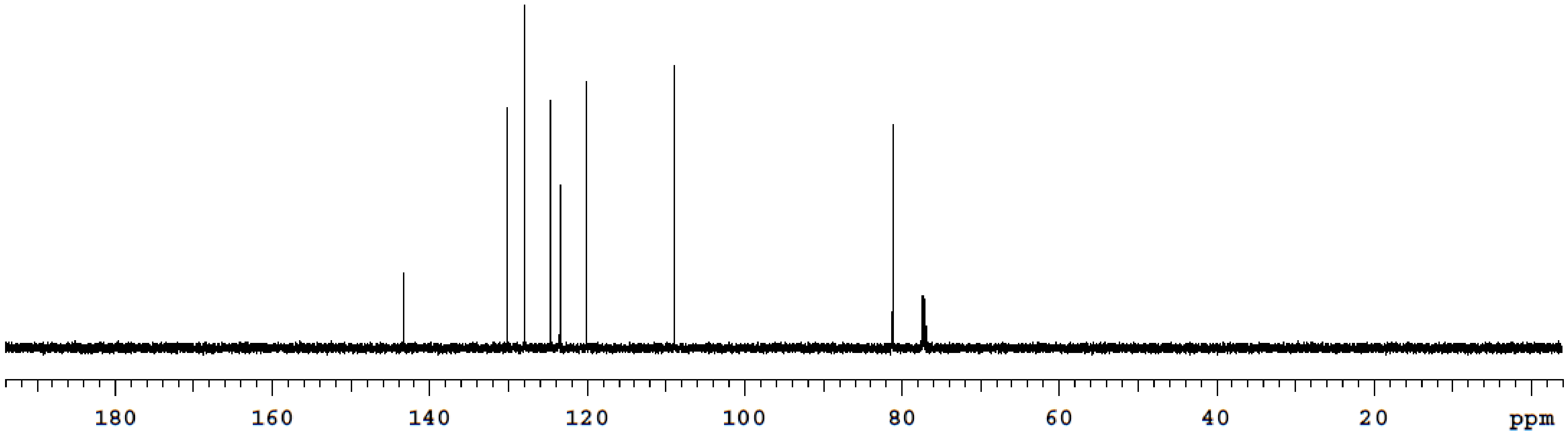

MKS-1205-12-73-CDCl3-CC

Pulse Sequence: s2pul

Solvent: cdcl3
Ambient temperature
Operator: mkl
File: MKS-1205-12-73-CDCl3-CC
INOVA-500 "riga"

Pulse 45.0 degrees
Acq. time 1.892 sec
Width 8000.0 Hz
112 repetitions
OBSERVE H1, 499.7707217 MHz
DATA PROCESSING
Line broadening 0.2 Hz
FT size 32768
Total time 1 hr, 3 min, 21 sec

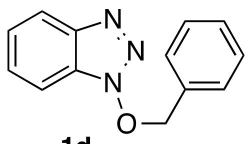

1d

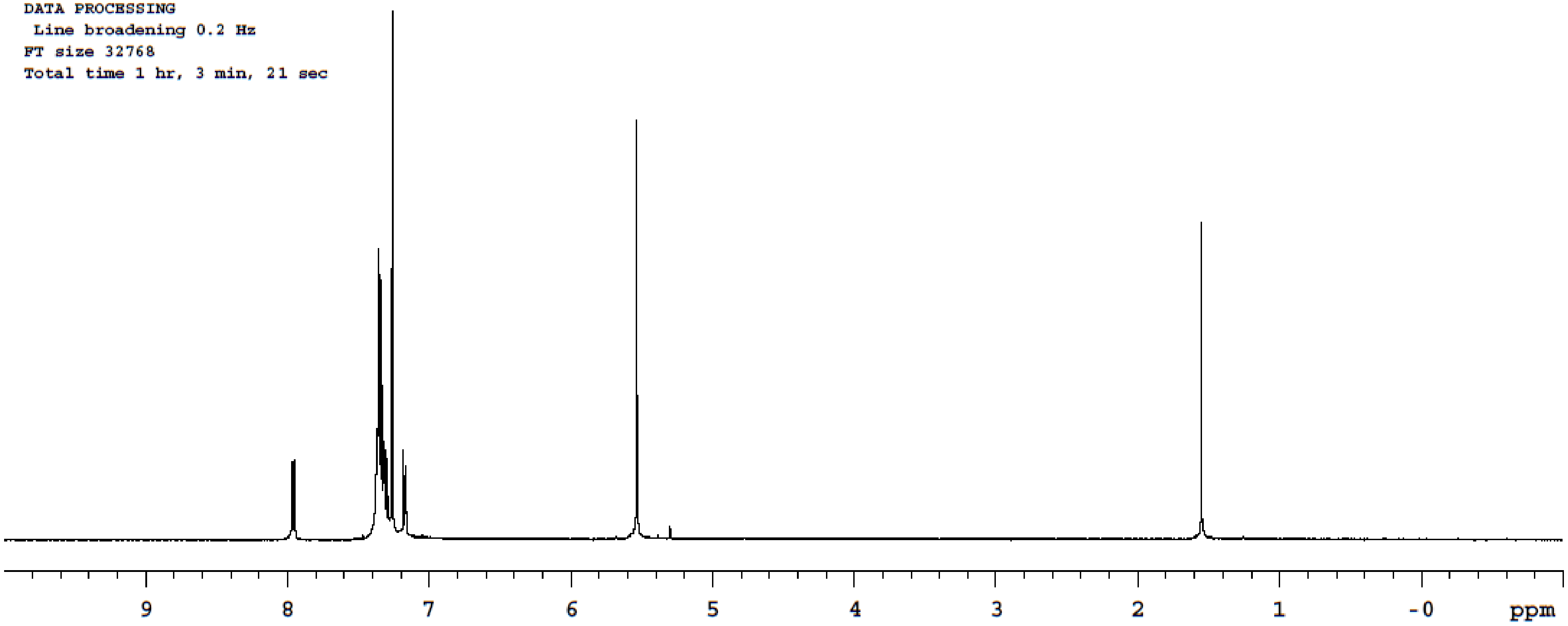

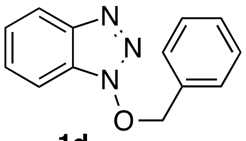

1d

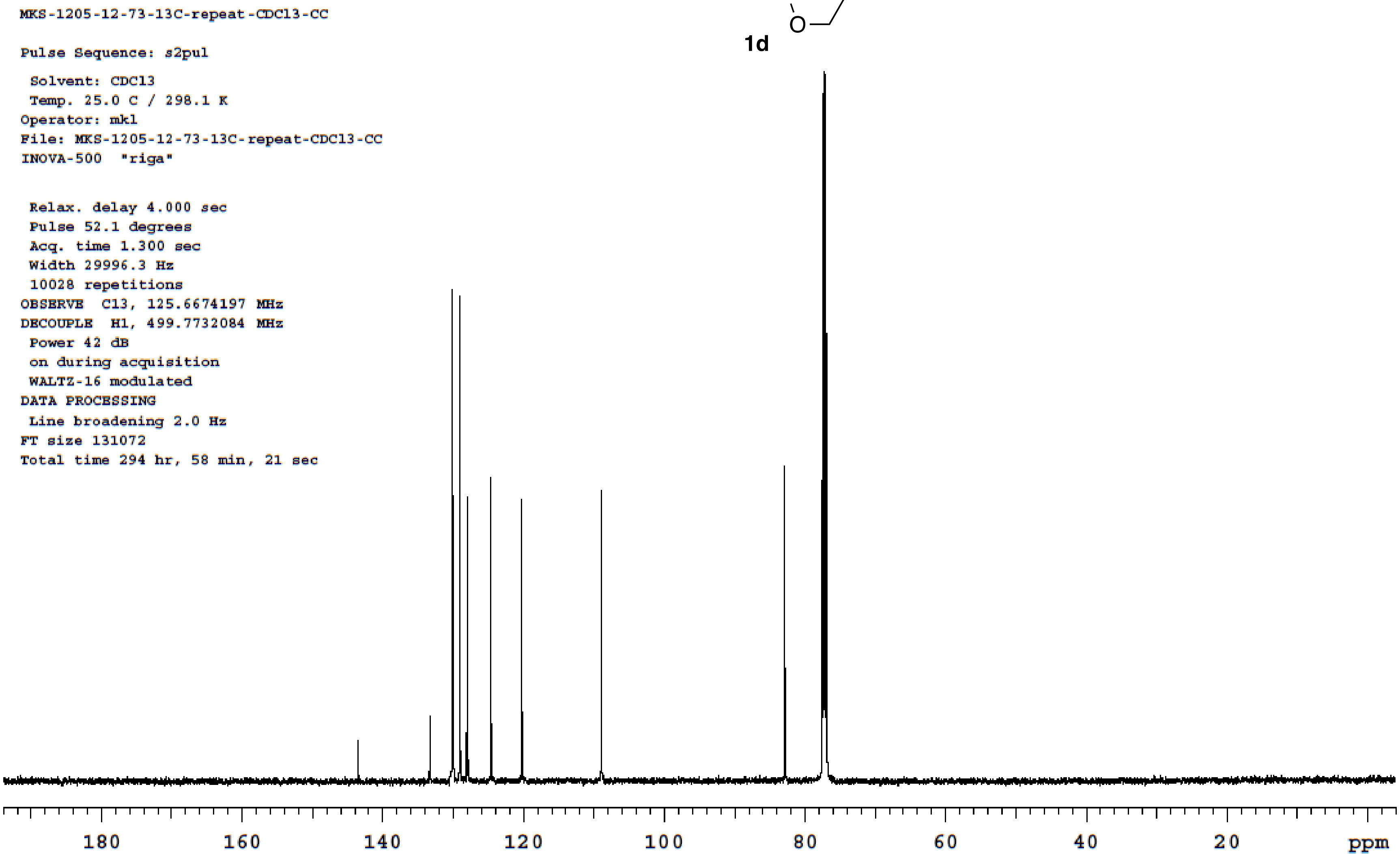
MKS-1205-12-73-13C-repeat-CDCl3-CC
Pulse Sequence: s2pul
Solvent: CDCl3
Temp. 25.0 C / 298.1 K
Operator: mkl
File: MKS-1205-12-73-13C-repeat-CDCl3-CC
INOVA-500 "riga"
Relax. delay 4.000 sec
Pulse 52.1 degrees
Acq. time 1.300 sec
Width 29996.3 Hz
10028 repetitions
OBSERVE C13, 125.6674197 MHz
DECOUPLE H1, 499.7732084 MHz
Power 42 dB
on during acquisition
WALTZ-16 modulated
DATA PROCESSING
Line broadening 2.0 Hz
FT size 131072
Total time 294 hr, 58 min, 21 sec
180
160
140
120
100
80
60
40
20
ppm

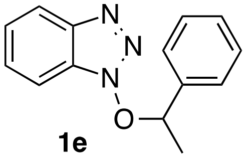

1e

RRC-1205-III-243-HNMR-CDCl3

Pulse Sequence: s2pul

Solvent: CDCl3
Temp. 25.0 C / 298.1 K
Operator: Raghu
File: RRC-1205-III-243-HNMR-CDCl3
INOVA-500 "riga"

Relax. delay 5.000 sec
Pulse 45.0 degrees
Acq. time 1.892 sec
Width 8000.0 Hz
24 repetitions
OBSERVE H1, 499.7707212 MHz
DATA PROCESSING
Resol. enhancement 0.5 Hz
Gauss apodization 0.500 sec
FT size 32768
Total time 2 min, 45 sec

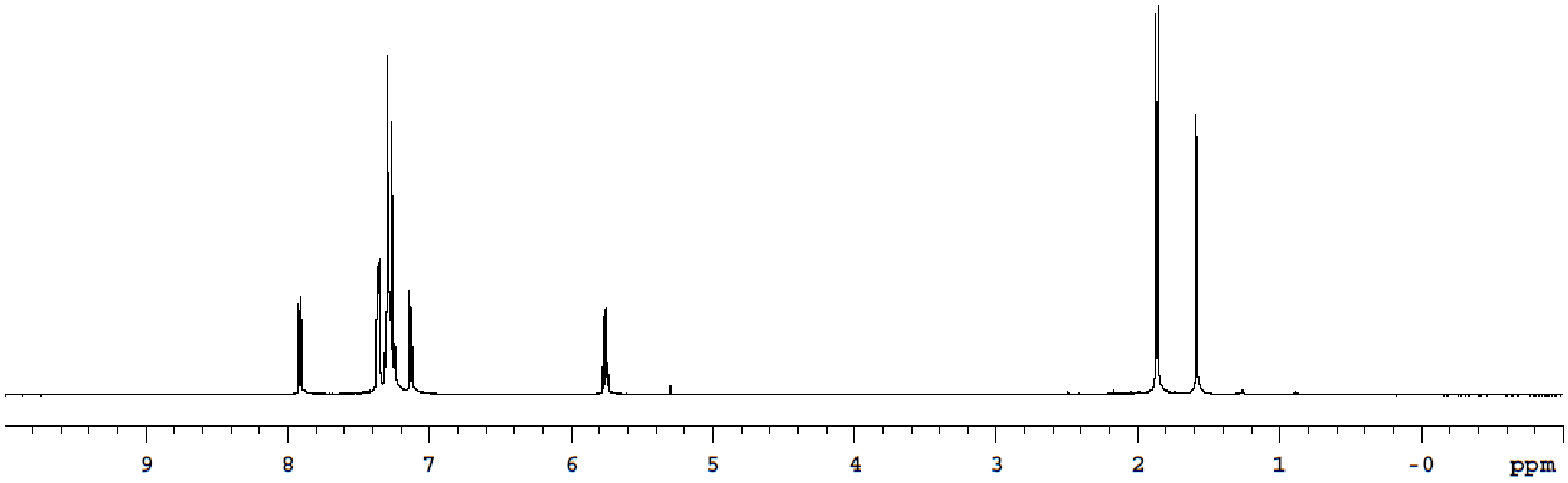

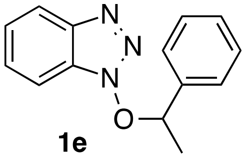

1e

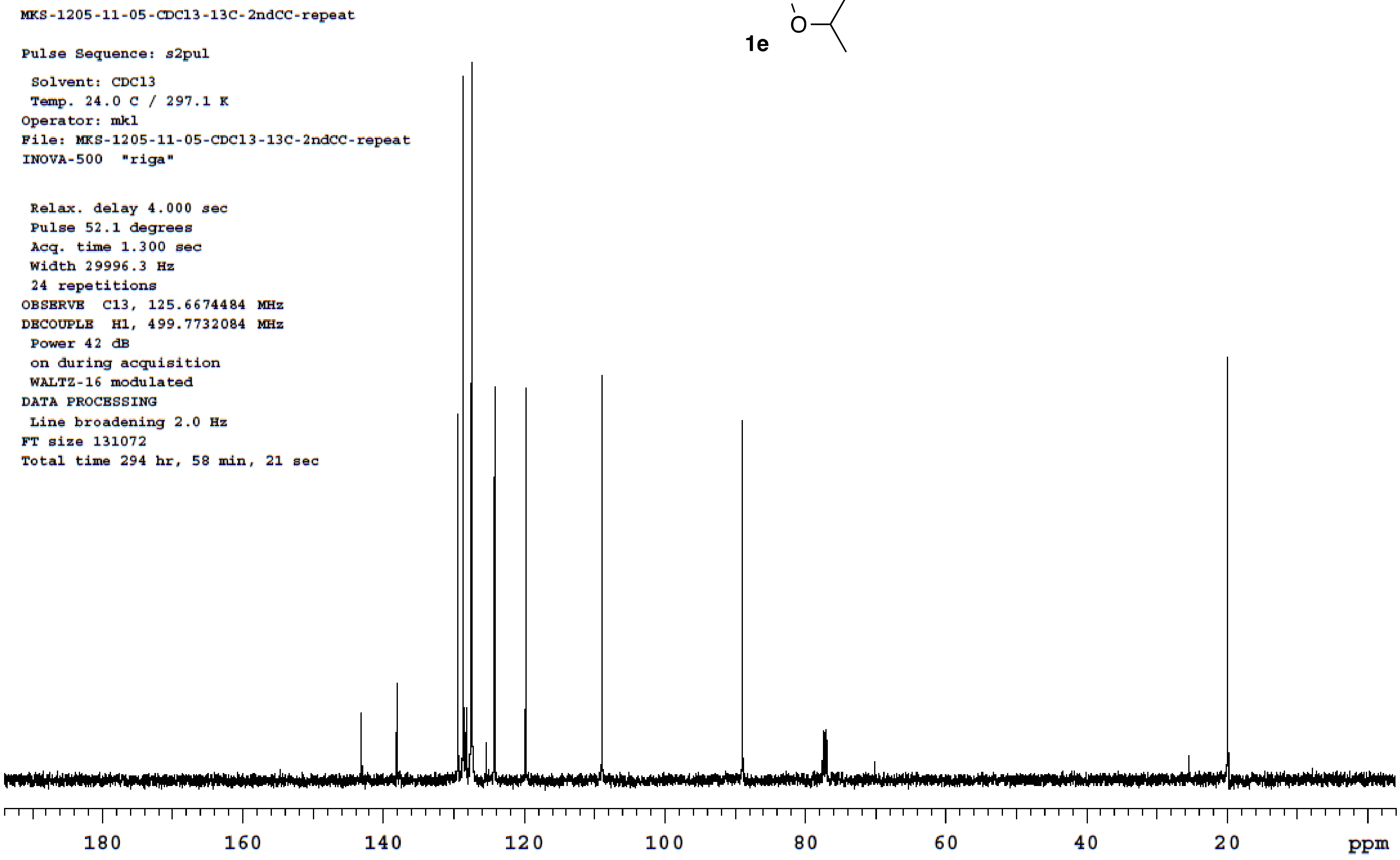
MKS-1205-11-05-CDCl3-13C-2ndCC-repeat
Pulse Sequence: s2pul
Solvent: CDCl3
Temp. 24.0 C / 297.1 K
Operator: mkl
File: MKS-1205-11-05-CDCl3-13C-2ndCC-repeat
INOVA-500 "riga"
Relax. delay 4.000 sec
Pulse 52.1 degrees
Acq. time 1.300 sec
Width 29996.3 Hz
24 repetitions
OBSERVE C13, 125.6674484 MHz
DECOUPLE H1, 499.7732084 MHz
Power 42 dB
on during acquisition
WALTZ-16 modulated
DATA PROCESSING
Line broadening 2.0 Hz
FT size 131072
Total time 294 hr, 58 min, 21 sec
180
160
140
120
100
80
60
40
20
ppm

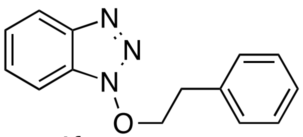

1f

MKS-1205-08-51-CDCl3-CC

Pulse Sequence: s2pul

Solvent: cdcl3
Temp. 25.0 C / 298.1 K
Operator: mkl
File: MKS-1205-08-51-CDCl3-CC
INOVA-500 "riga"

Pulse 45.0 degrees
Acq. time 1.892 sec
Width 8000.0 Hz
48 repetitions
OBSERVE H1, 499.7707217 MHz
DATA PROCESSING
FT size 32768
Total time 1 hr, 4 min, 52 sec

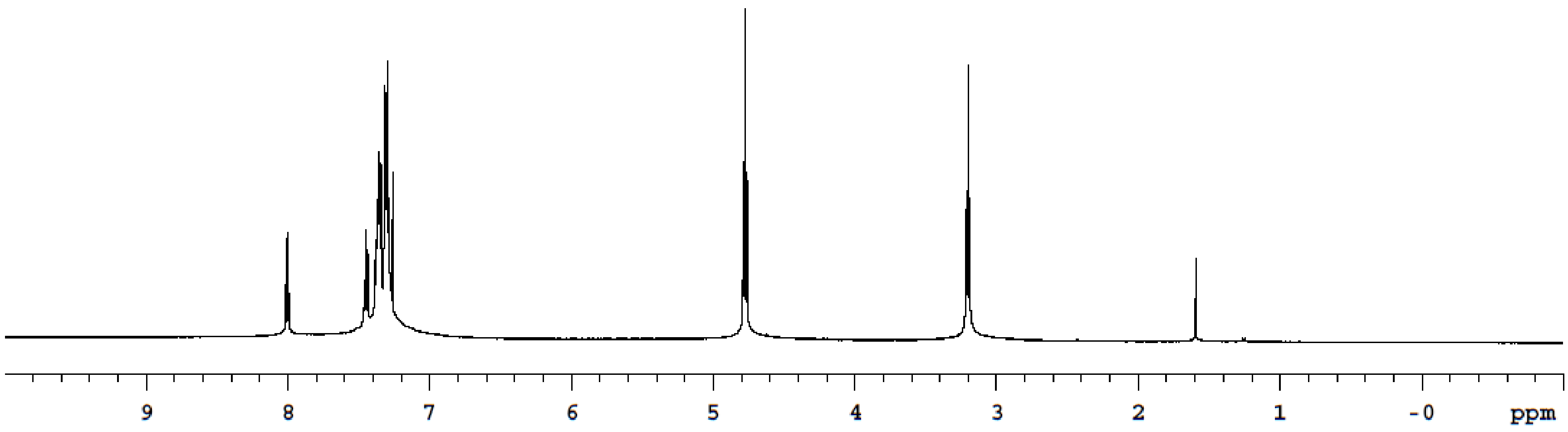

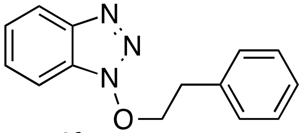

1f

MKS-1205-08-51-CDCl3-13C-CC

Pulse Sequence: s2pul

Solvent: CDCl3
Temp. 25.0 C / 298.1 K
Operator: mkl
File: MKS-1205-08-51-CDCl3-13C-CC
INOVA-500 "riga"

Relax. delay 3.000 sec
Pulse 45.0 degrees
Acq. time 1.300 sec
Width 25000.0 Hz
96 repetitions
OBSERVE C13, 125.6674382 MHz
DECOUPLE H1, 499.7730084 MHz
Power 39 dB
continuously on
WALTZ-16 modulated
DATA PROCESSING
Line broadening 0.2 Hz
FT size 65536
Total time 2 hr, 27 min, 5 sec

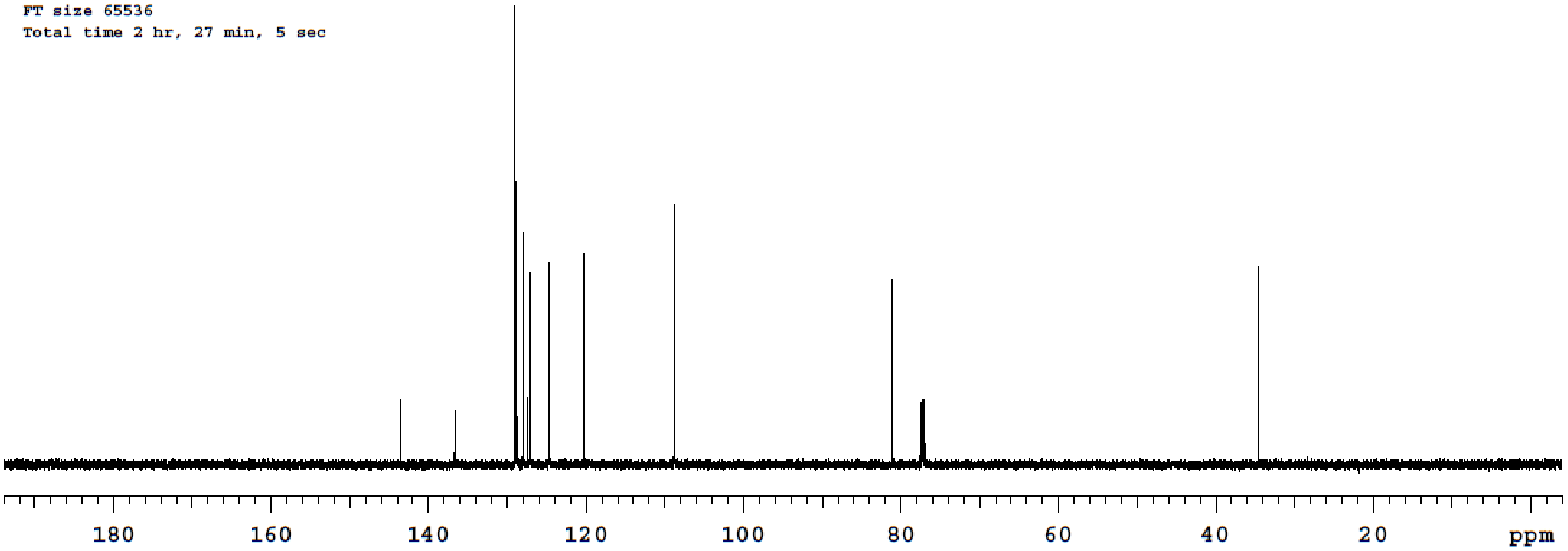

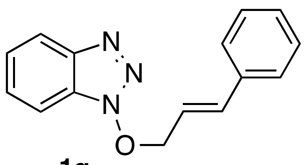

1g

RRC-1205-IV-276-HNMR-CDCl3

Pulse Sequence: s2pul

Solvent: CDCl3
Temp. 25.0 C / 298.1 K
Operator: Raghu
File: RRC-1205-IV-276-HNMR-CDCl3
INOVA-500 "riga"

Relax. delay 5.000 sec
Pulse 45.0 degrees
Acq. time 1.892 sec
Width 8000.0 Hz
32 repetitions
OBSERVE H1, 499.7707212 MHz
DATA PROCESSING
Resol. enhancement 0.5 Hz
Gauss apodization 0.500 sec
FT size 32768
Total time 7 min, 21 sec

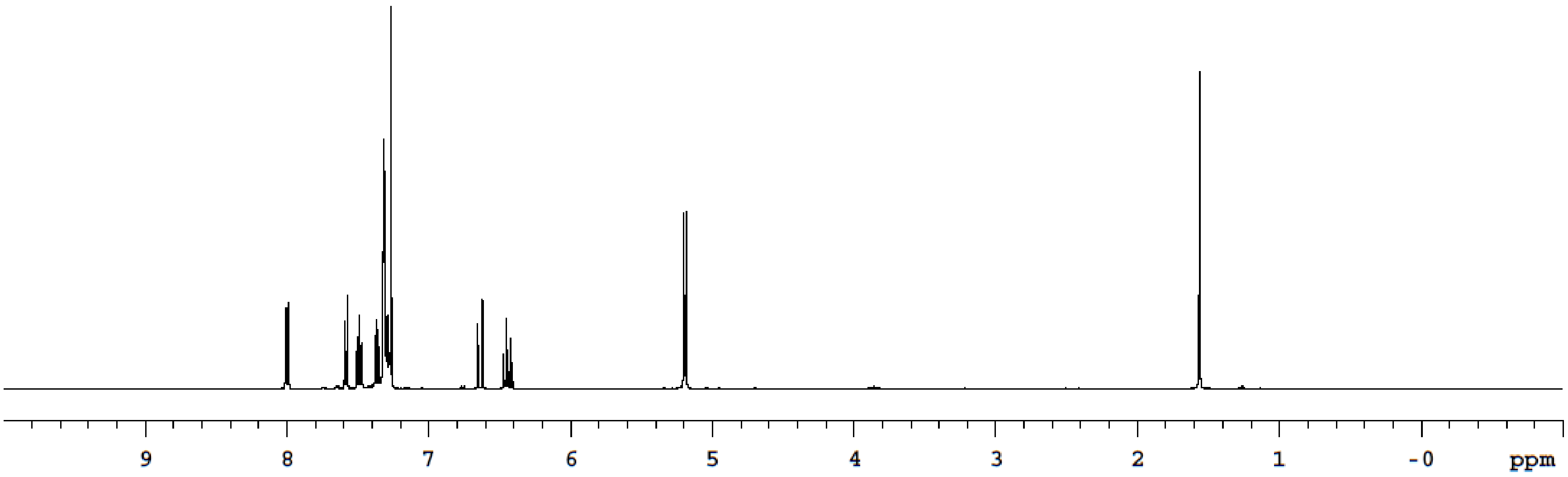

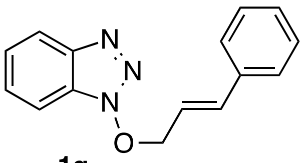

1g

RRC-1205-IV-276-CNMR-CDCl3

Pulse Sequence: *s2pul*

Solvent: CDCl3
Temp. 25.0 C / 298.1 K
Operator: Raghu
File: RRC-1205-IV-276-CNMR-CDCl3
INOVA-500 "riga"

Relax. delay 3.000 sec
Pulse 45.0 degrees
Acq. time 1.300 sec
Width 30499.4 Hz
12910 repetitions
OBSERVE C13, 125.6674203 MHz
DECOUPLE H1, 499.7730084 MHz
Power 40 dB
continuously on
WALTZ-16 modulated
DATA PROCESSING
Line broadening 2.0 Hz
FT size 131072
Total time 23 hr, 56 min, 27 sec

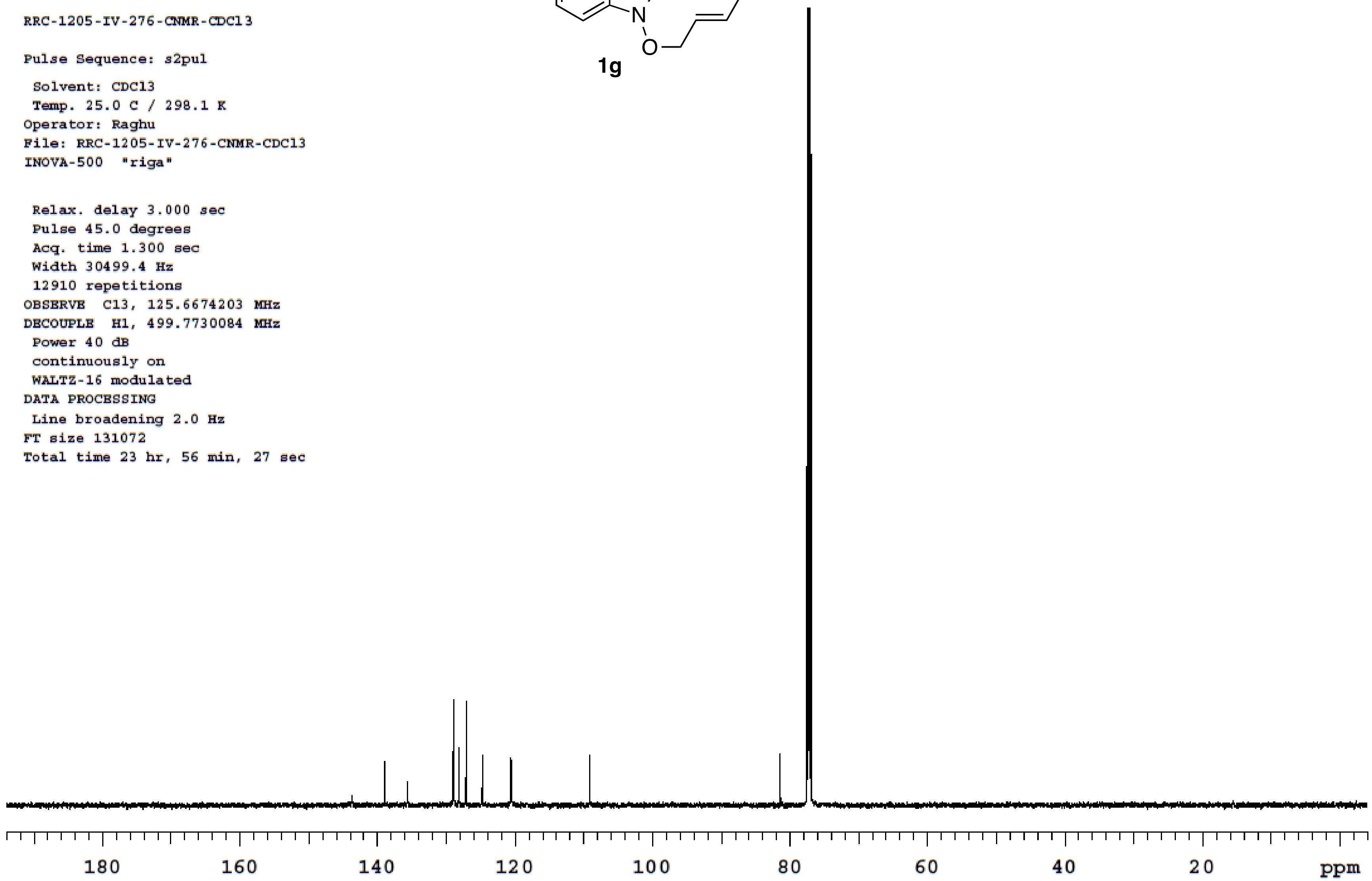

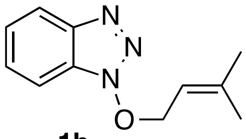
1h

1205-Evan-isoprenyBtether-1H-CDCl3

Pulse Sequence: *s2pul*

Solvent: CDCl3
Temp. 25.0 C / 298.1 K
Operator: mkl
File: 1205-Evan-isoprenyBtether-1H-CDCl3
INOVA-500 "riga"

Relax. delay 5.000 sec
Pulse 38.6 degrees
Acq. time 1.892 sec
Width 6002.4 Hz
8 repetitions
OBSERVE H1, 499.7707095 MHz
DATA PROCESSING
FT size 32768
Total time 3 min, 40 sec

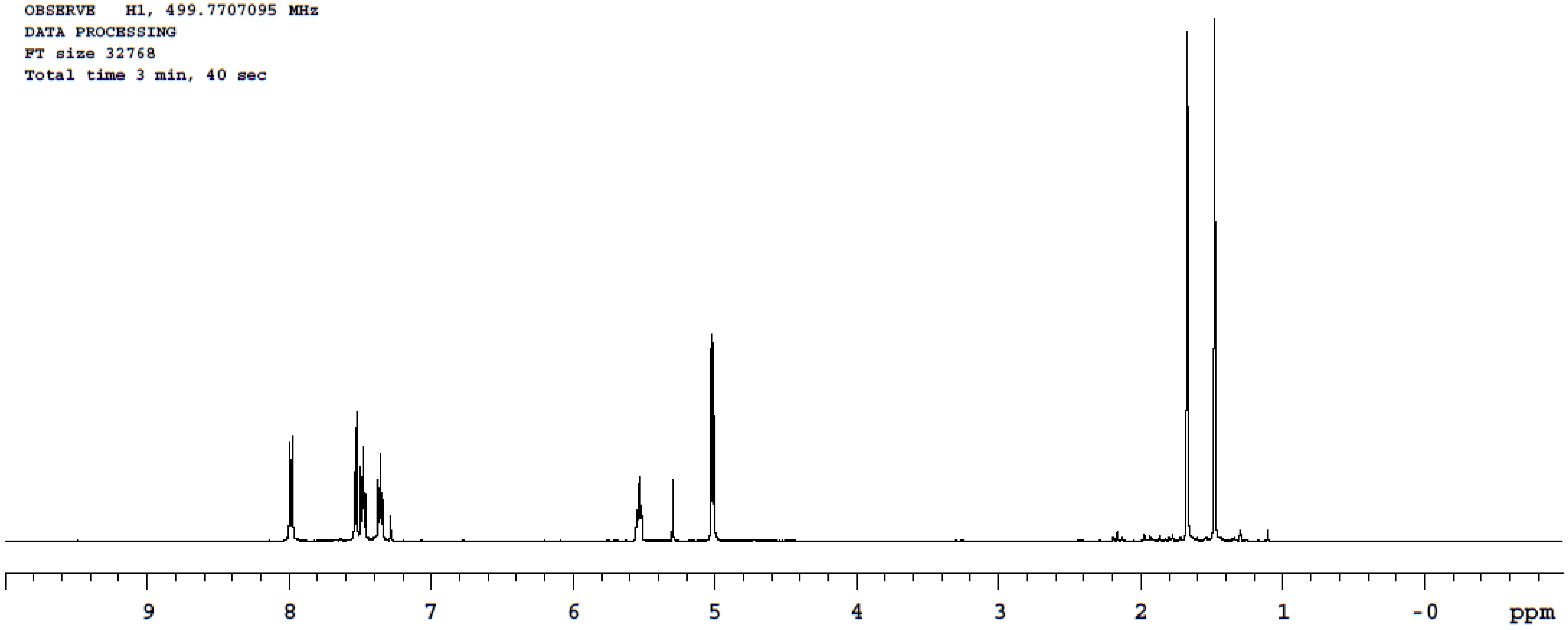

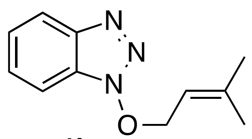

1h

1205-Evans-isoprenylBtether-13C-CDCl3

Pulse Sequence: s2pul

Solvent: CDCl3
Ambient temperature
Operator: mkl
File: 1205-Evans-isoprenylBtether-13C-CDCl3
INOVA-500 "riga"

Relax. delay 2.000 sec
Pulse 45.0 degrees
Acq. time 1.300 sec
Width 29996.3 Hz
1752 repetitions
OBSERVE C13, 125.6674264 MHz
DECOUPLE H1, 499.7730084 MHz
Power 39 dB
continuously on
WALTZ-16 modulated
DATA PROCESSING
Line broadening 0.5 Hz
FT size 131072
Total time 18 hr, 23 min, 8 sec

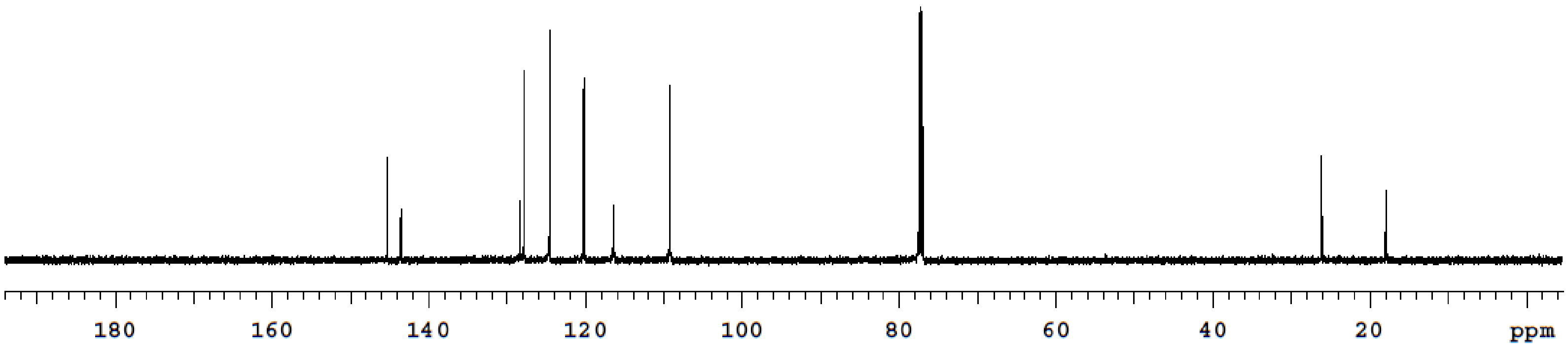

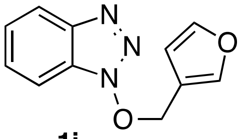

1i

MKS-1205-12-13-CDCl3-CC

Pulse Sequence: s2pul

Solvent: cdcl3
Temp. 25.0 C / 298.1 K
Operator: mkl
File: MKS-1205-12-13-CDCl3-CC
INOVA-500 "riga"

Pulse 45.0 degrees
Acq. time 1.892 sec
Width 8000.0 Hz
48 repetitions
OBSERVE H1, 499.7707202 MHz
DATA PROCESSING
FT size 32768
Total time 6 min, 20 sec

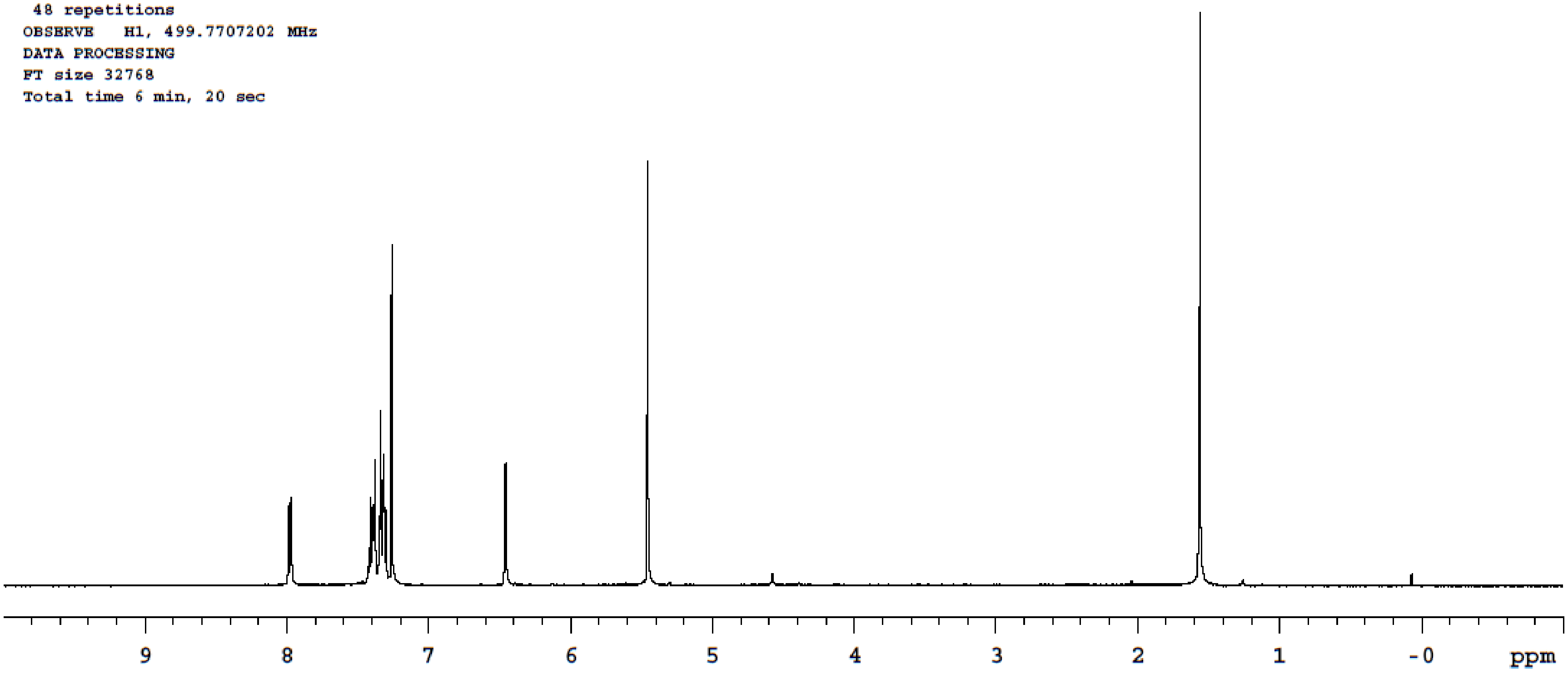

MKS-1205-09-47-CDCl3-13C-CC

Pulse Sequence: s2pul

Solvent: CDCl3
Temp. 25.0 C / 298.1 K
Operator: mkl
File: MKS-1205-09-47-CDCl3-13C-CC
INOVA-500 "riga"

Relax. delay 3.000 sec
Pulse 45.0 degrees
Acq. time 1.300 sec
Width 25000.0 Hz
368 repetitions
OBSERVE C13, 125.6674240 MHz
DECOUPLE H1, 499.7730084 MHz
Power 39 dB
continuously on
WALTZ-16 modulated
DATA PROCESSING
Line broadening 0.2 Hz
FT size 65536
Total time 2 hr, 27 min, 5 sec

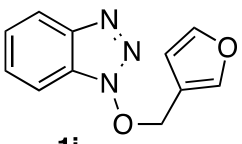

1i

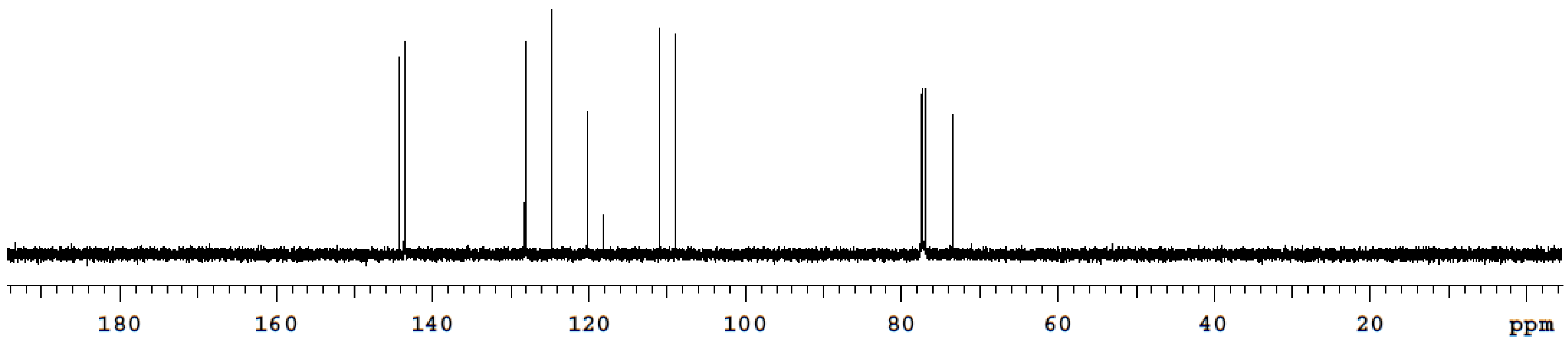

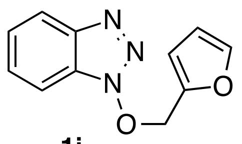

1j

1205-Vijay-03-34-1H-CDCl3

Pulse Sequence: s2pul

Solvent: CDCl3
Temp. 25.0 C / 298.1 K
Operator: mkl
File:
INOVA-500 "riga"

Relax. delay 5.000 sec
Pulse 38.6 degrees
Acq. time 1.892 sec
Width 6002.4 Hz
32 repetitions
OBSERVE H1, 499.7707202 MHz
DATA PROCESSING
FT size 32768
Total time 3 min, 40 sec

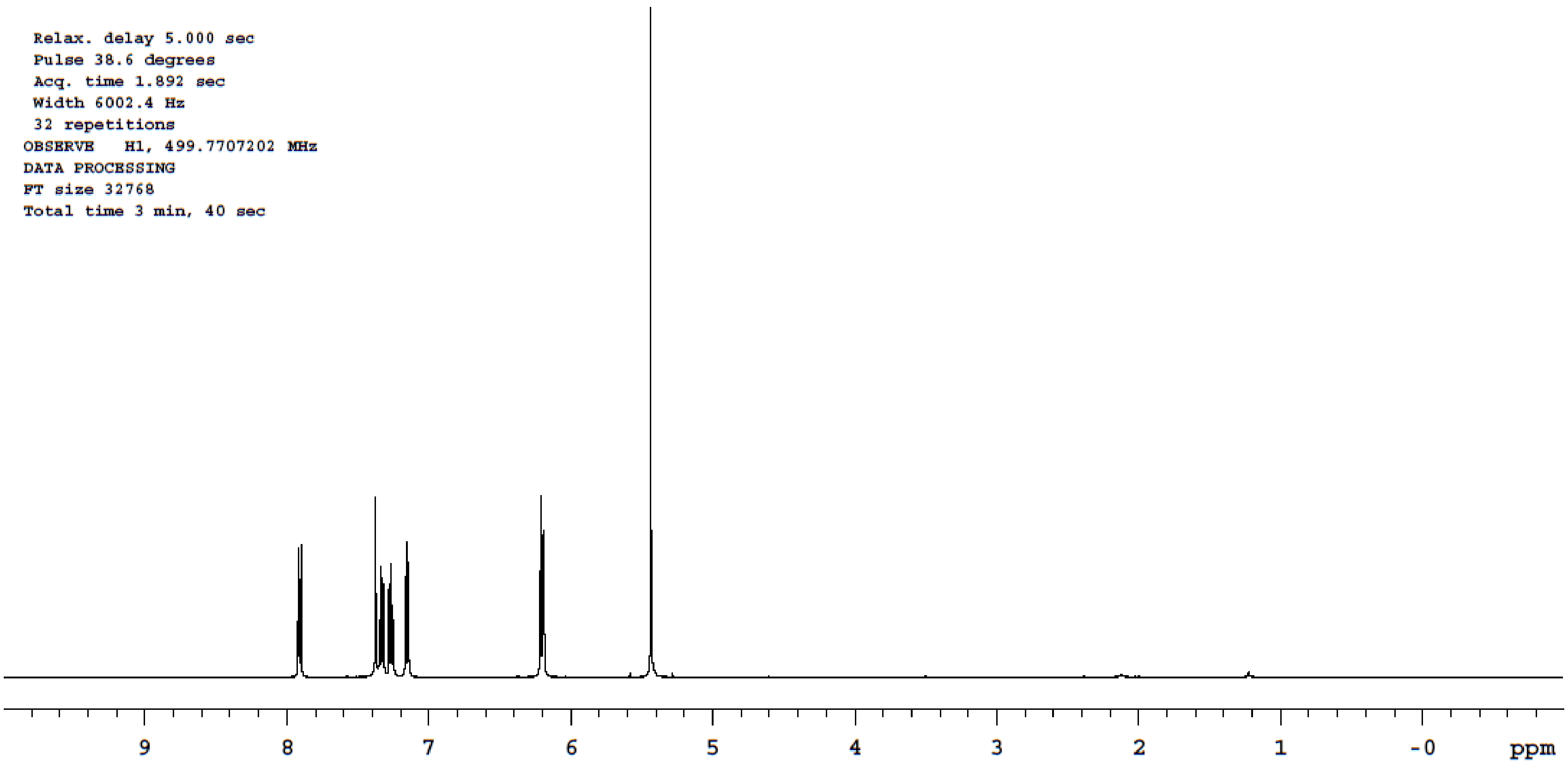

1205-Vijay-03-34-13C-CDCl3

Pulse Sequence: s2pul

Solvent: CDCl3
Ambient temperature
Operator: mkl
File:
INOVA-500 "riga"

Pulse 45.0 degrees
Acq. time 1.300 sec
Width 25000.0 Hz
204 repetitions
OBSERVE C13, 125.6674263 MHz
DECOUPLE H1, 499.7730084 MHz
Power 39 dB
continuously on
WALTZ-16 modulated
DATA PROCESSING
Line broadening 0.5 Hz
FT size 65536
Total time 21 min, 49 sec

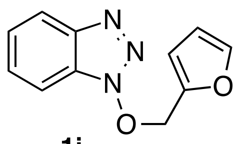

1j

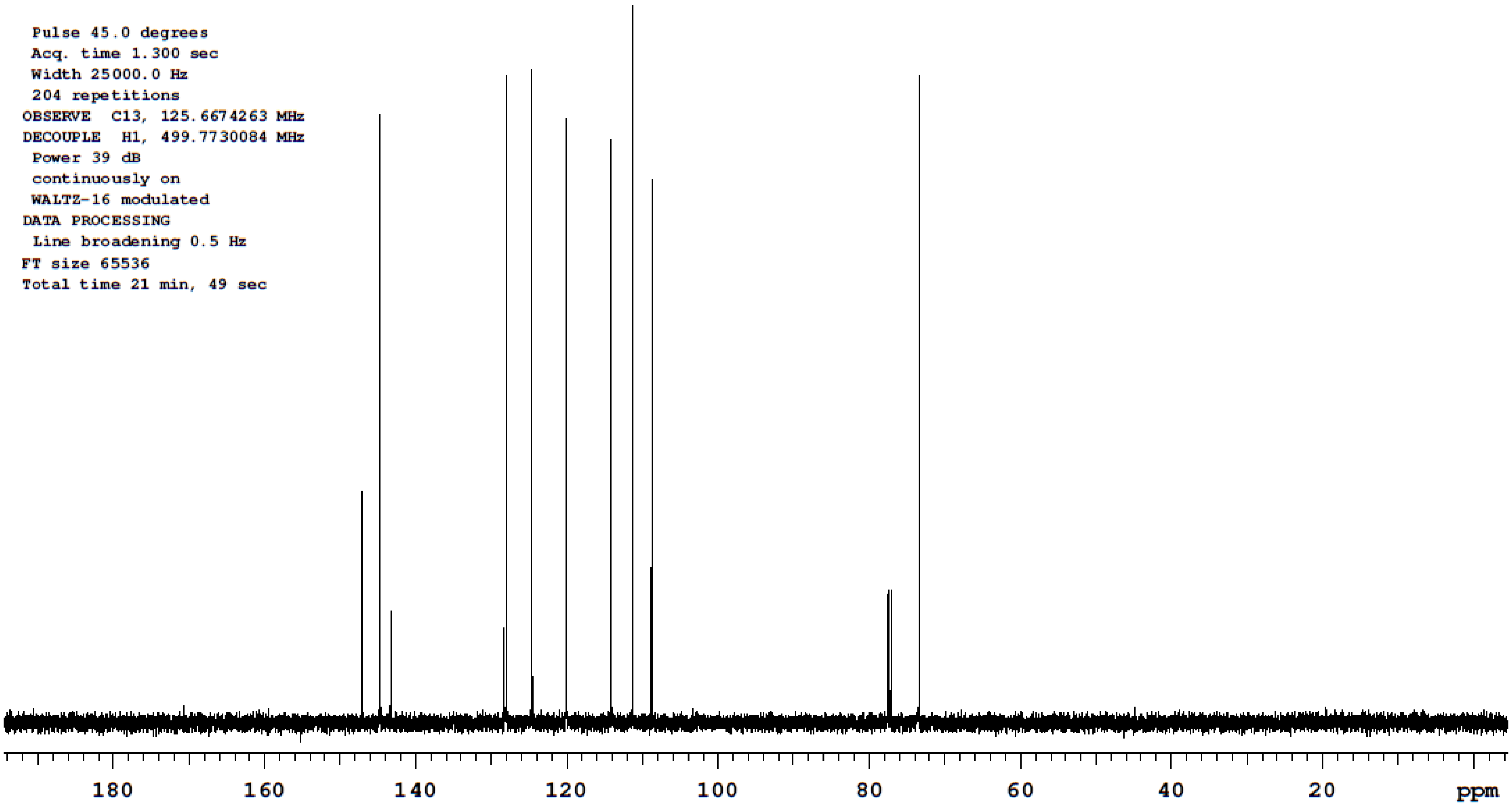

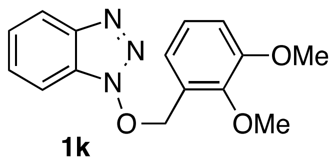

1k

MKS-1205-08-02-CDCl3-CC

Archive directory: /export/home/mkl/vnmrsys/data
Sample directory:

Pulse Sequence: s2pul

Solvent: cdcl3
Temp. 25.0 C / 298.1 K
Operator: mkl
File: MKS-1205-08-02-CDCl3-CC
INOVA-500 "riga"

Pulse 45.0 degrees
Acq. time 1.892 sec
Width 8000.0 Hz
88 repetitions
OBSERVE H1, 499.7707222 MHz
DATA PROCESSING
FT size 32768
Total time 1 hr, 4 min, 52 sec

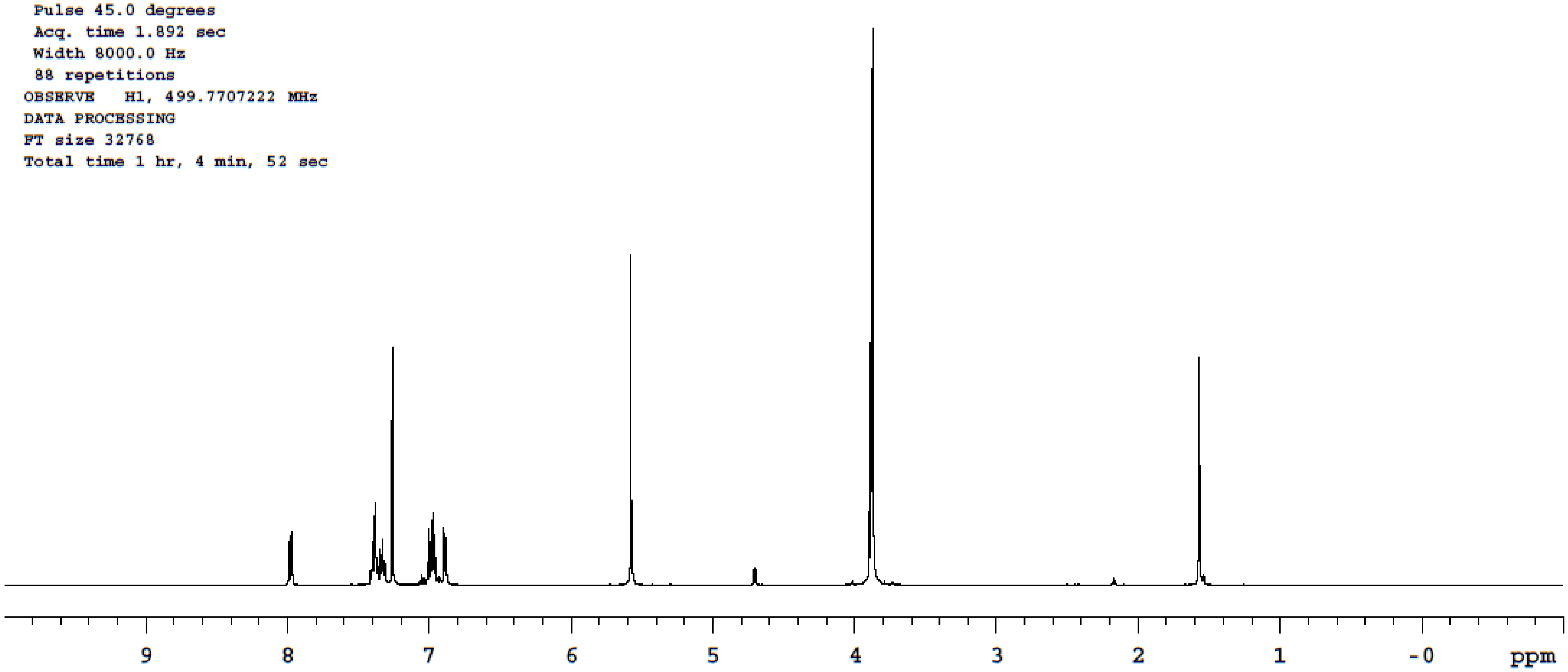

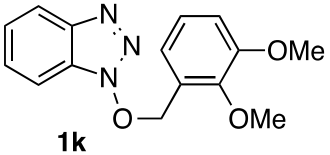

1k

MKS-1205-08-02-CDCl3-13C-1st

Pulse Sequence: s2pul

Solvent: CDCl3
Temp. 25.0 C / 298.1 K
Operator: mkl
File: MKS-1205-08-02-CDCl3-13C-1st
INOVA-500 "riga"

Relax. delay 3.000 sec
Pulse 45.0 degrees
Acq. time 1.300 sec
Width 25000.0 Hz
640 repetitions
OBSERVE C13, 125.6674232 MHz
DECOUPLE H1, 499.7730084 MHz
Power 39 dB
continuously on
WALTZ-16 modulated
DATA PROCESSING
Line broadening 0.2 Hz
FT size 65536
Total time 2 hr, 27 min, 5 sec

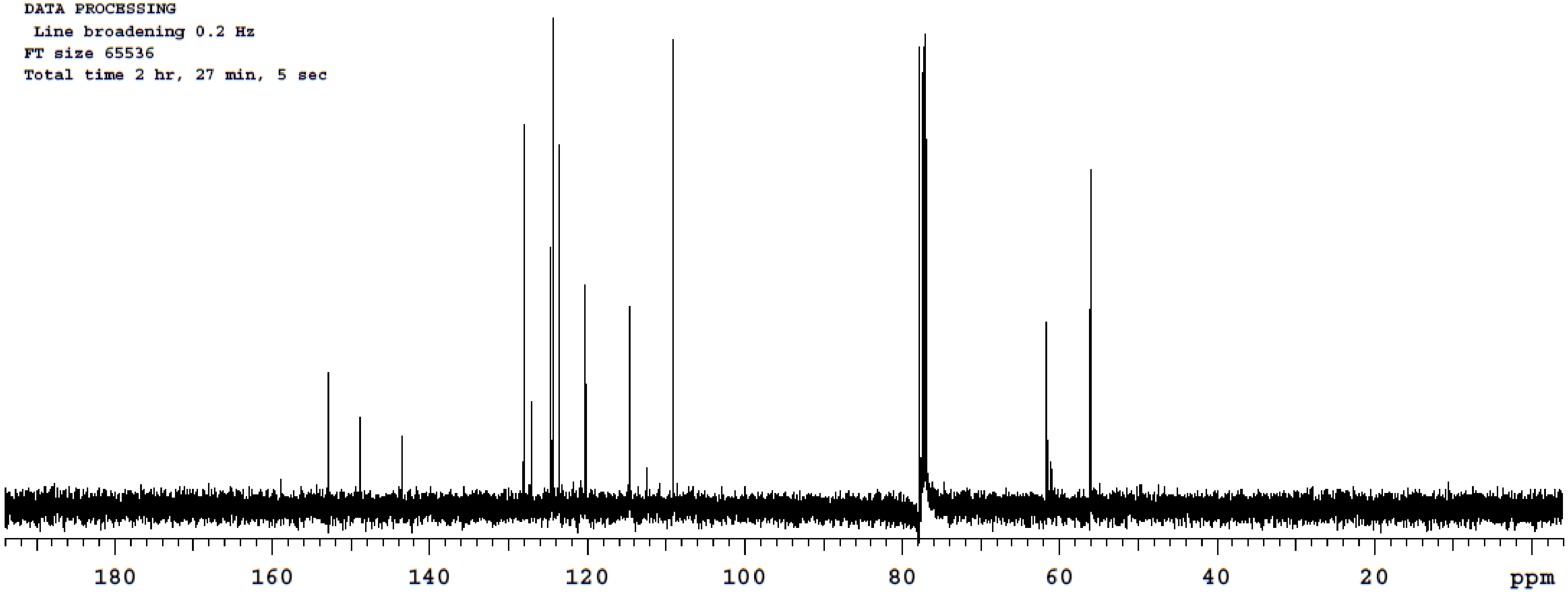

MKS-1205-11-61-CDCl3-3rdFrac-CC

Pulse Sequence: s2pul

Solvent: cdcl3
Temp. 25.0 C / 298.1 K
Operator: mkl
File: MKS-1205-11-61-CDCl3-3rdFrac-CC
INOVA-500 "riga"

Pulse 45.0 degrees
Acq. time 1.892 sec
Width 8000.0 Hz
68 repetitions
OBSERVE H1, 499.7707217 MHz
DATA PROCESSING
FT size 32768
Total time 6 min, 20 sec

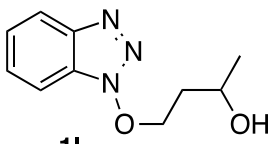

1l

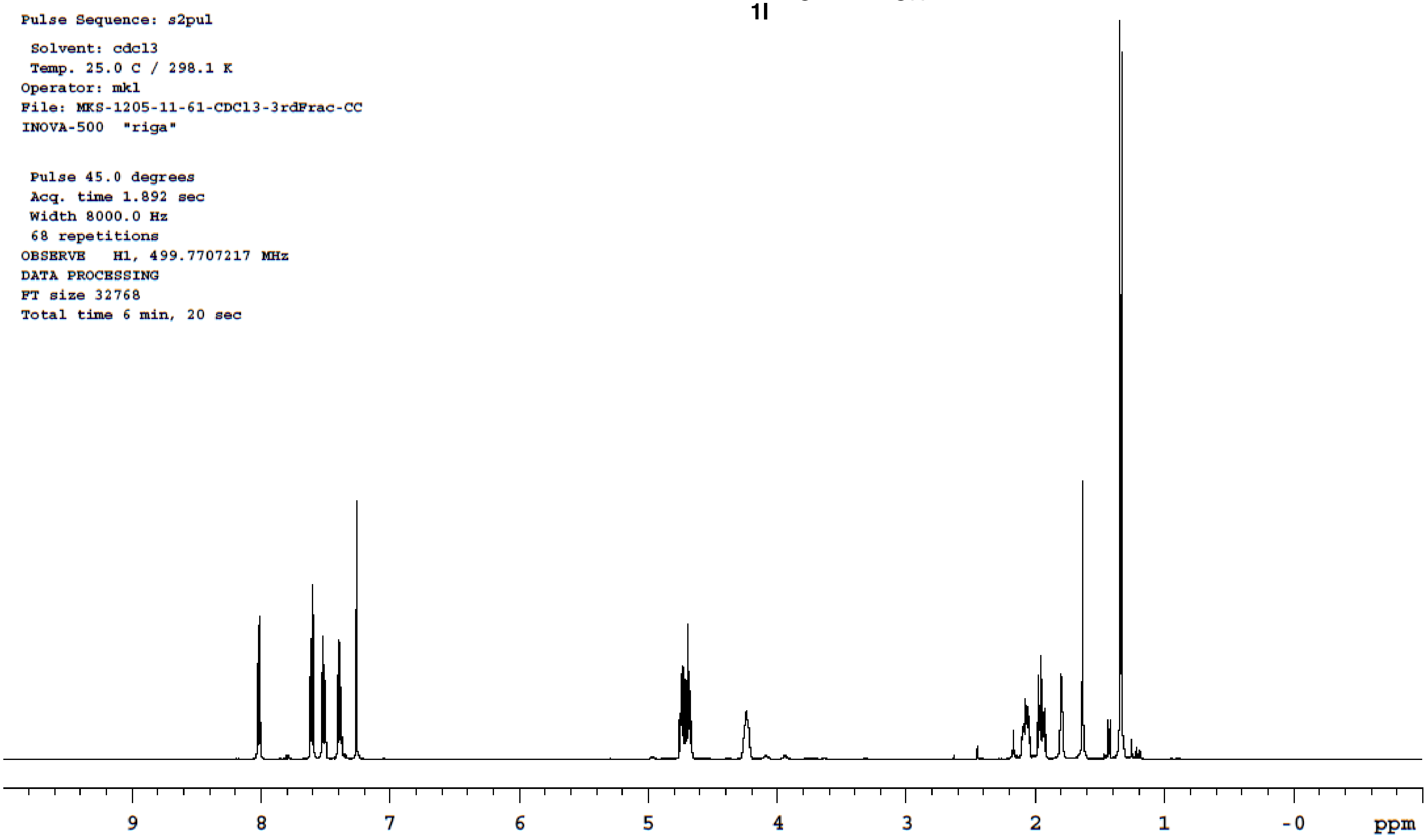

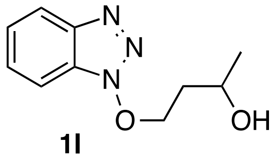

1l

MKS-1205-11-61-CDCl3-13C-2ndFrac-CC

Pulse Sequence: s2pul

Solvent: CDCl3
Temp. 24.0 C / 297.1 K
Operator: mkl
File: MKS-1205-11-61-CDCl3-13C-2ndFrac-CC
INOVA-500 "riga"

Relax. delay 3.000 sec
Pulse 45.0 degrees
Acq. time 1.300 sec
Width 25000.0 Hz
34224 repetitions
OBSERVE C13, 125.6674271 MHz
DECOUPLE H1, 499.7730084 MHz
Power 39 dB
continuously on
WALTZ-16 modulated
DATA PROCESSING
Line broadening 0.2 Hz
FT size 65536
Total time 47 hr, 52 min, 55 sec

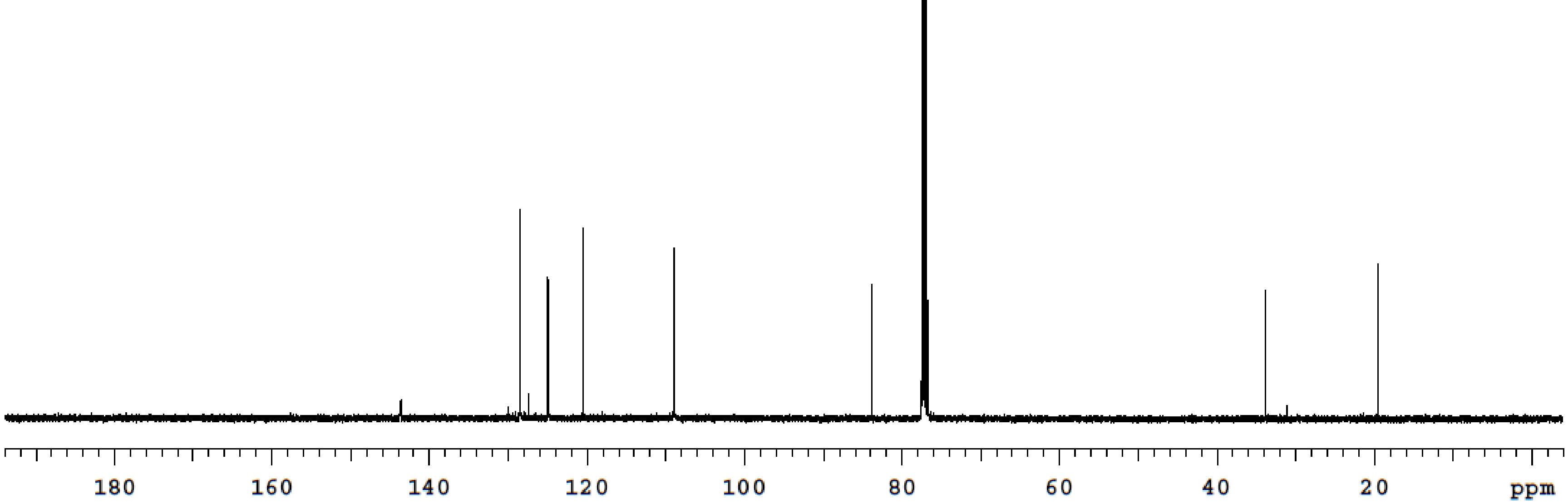

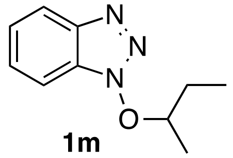

1m

RRC-1205-III-249-HNMR-CDCl3

Pulse Sequence: s2pul

Solvent: CDCl3
Temp. 25.0 C / 298.1 K
Operator: Raghu
File: RRC-1205-III-249-HNMR-CDCl3
INOVA-500 "riga"

Relax. delay 5.000 sec
Pulse 45.0 degrees
Acq. time 1.892 sec
Width 8000.0 Hz
64 repetitions
OBSERVE H1, 499.7707212 MHz
DATA PROCESSING
Resol. enhancement 0.5 Hz
Gauss apodization 0.500 sec
FT size 32768
Total time 7 min, 21 sec

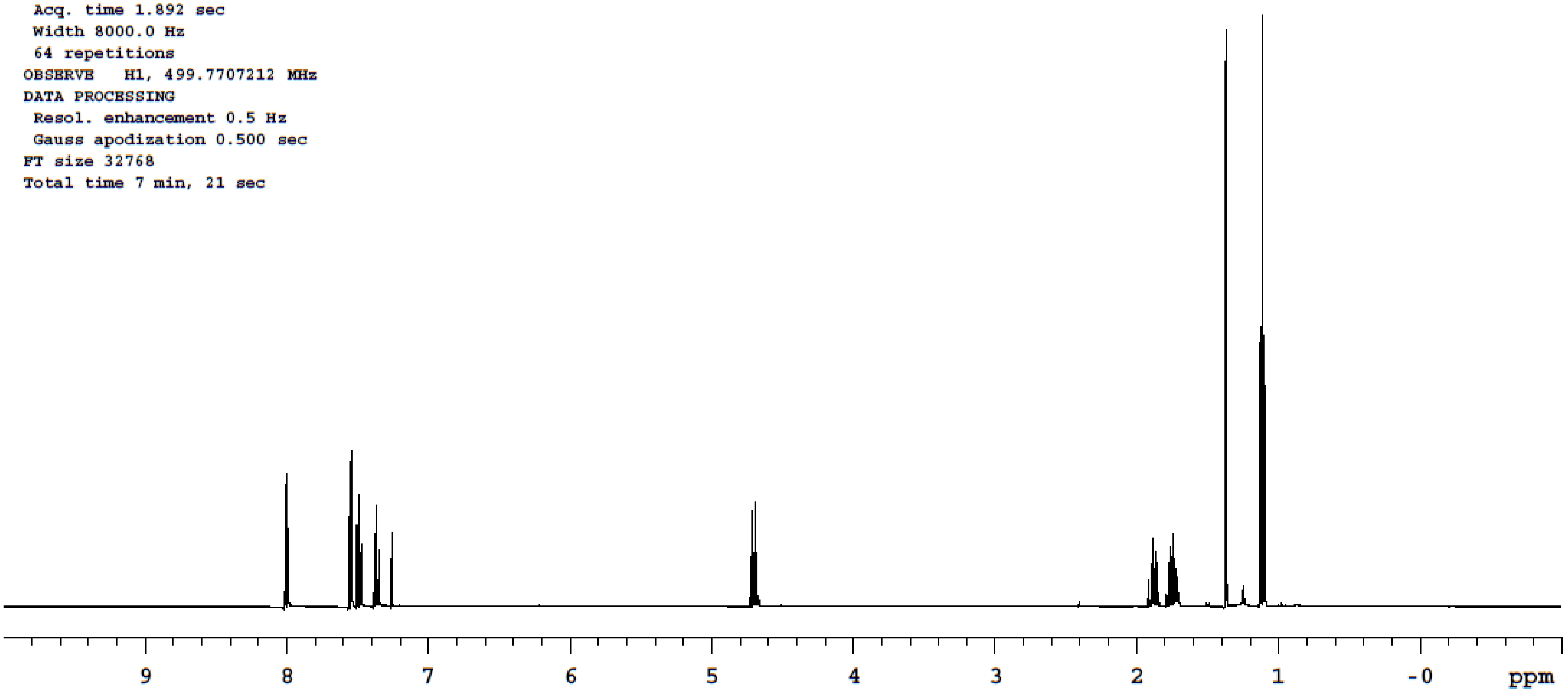

RRC-1205-III-249-HNMR-CDCl3

Pulse Sequence: s2pul

Solvent: CDCl3
Temp. 25.0 C / 298.1 K
Operator: Raghu
File: RRC-1205-III-249-HNMR-CDCl3
INOVA-500 "riga"

Relax. delay 5.000 sec
Pulse 45.0 degrees
Acq. time 1.892 sec
Width 8000.0 Hz
64 repetitions
OBSERVE H1, 499.7707212 MHz
DATA PROCESSING
Resol. enhancement 0.5 Hz
Gauss apodization 0.500 sec
FT size 32768
Total time 7 min, 21 sec

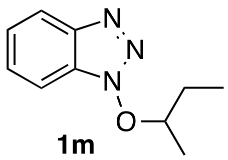

1m
Expanded spectrum

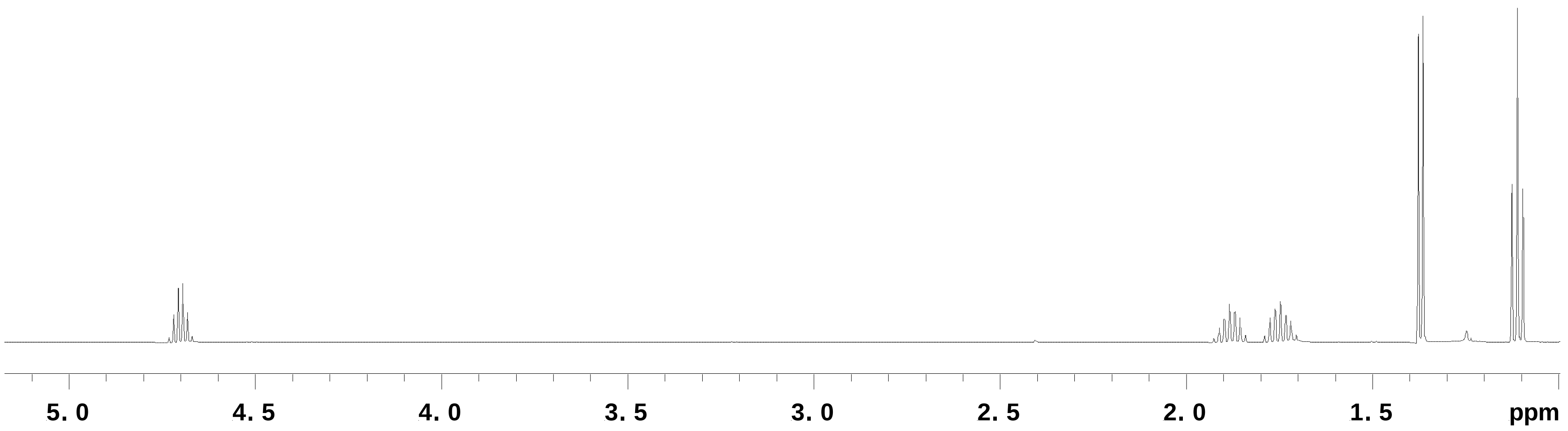

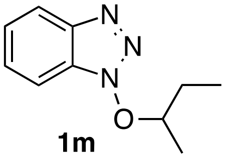

1m

```
RRC-1205-249-CNMR-CDCl3

Pulse Sequence: s2pul

 Solvent: CDCl3
 Temp. 25.0 C / 298.1 K
Operator: Raghu
File: RRC-1205-249-CNMR-CDCl3
INOVA-500  "riga"

 Relax. delay 3.000 sec
 Pulse 45.0 degrees
 Acq. time 1.300 sec
 Width 29996.3 Hz
 13310 repetitions
OBSERVE  C13, 125.6674209 MHz
DECOUPLE  H1, 499.7730084 MHz
 Power 40 dB
 continuously on
 WALTZ-16 modulated
DATA PROCESSING
 Line broadening 2.0 Hz
FT size 131072
Total time 29 hr, 55 min, 35 sec
```

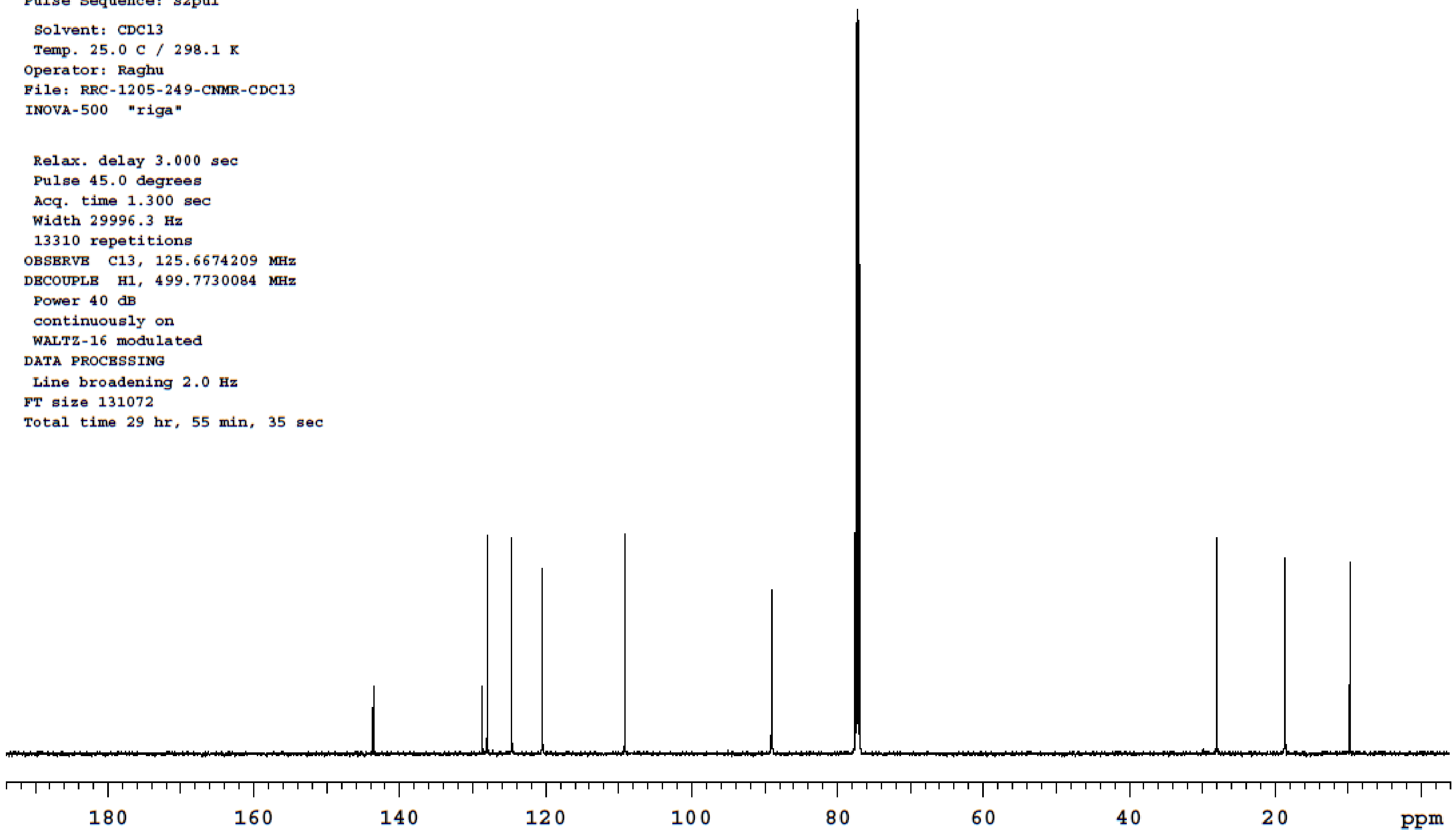

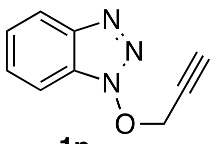

1n

RRC-1205-I-59-HNMR-CDCl3-pure

Pulse Sequence: s2pul
Solvent: CDCl3
Ambient temperature
Operator: Raghu
File: RRC-1205-I-59-HNMR-CDCl3-pure
INOVA-500 "riga"

Relax. delay 3.000 sec
Pulse 45.0 degrees
Acq. time 1.892 sec
Width 8000.0 Hz
64 repetitions
OBSERVE H1, 499.7707317 MHz
DATA PROCESSING
Resol. enhancement 0.5 Hz
Gauss apodization 0.500 sec
FT size 32768
Total time 5 min, 13 sec

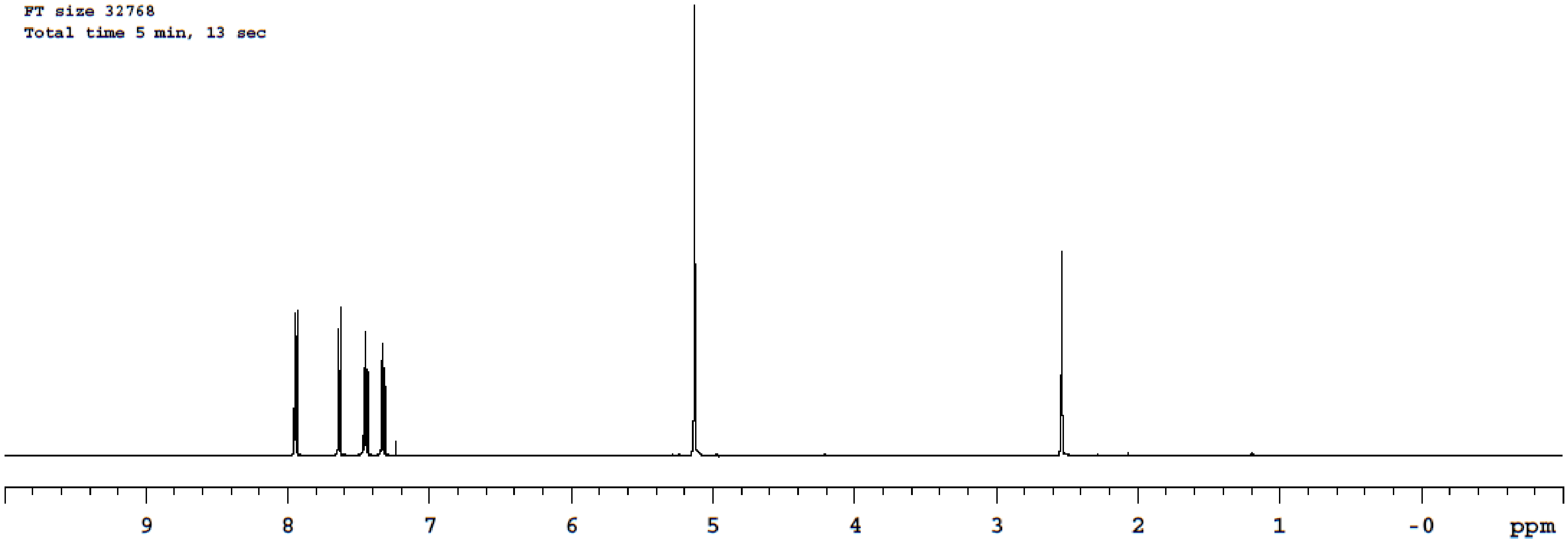

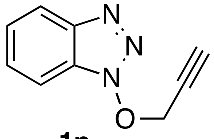

1n

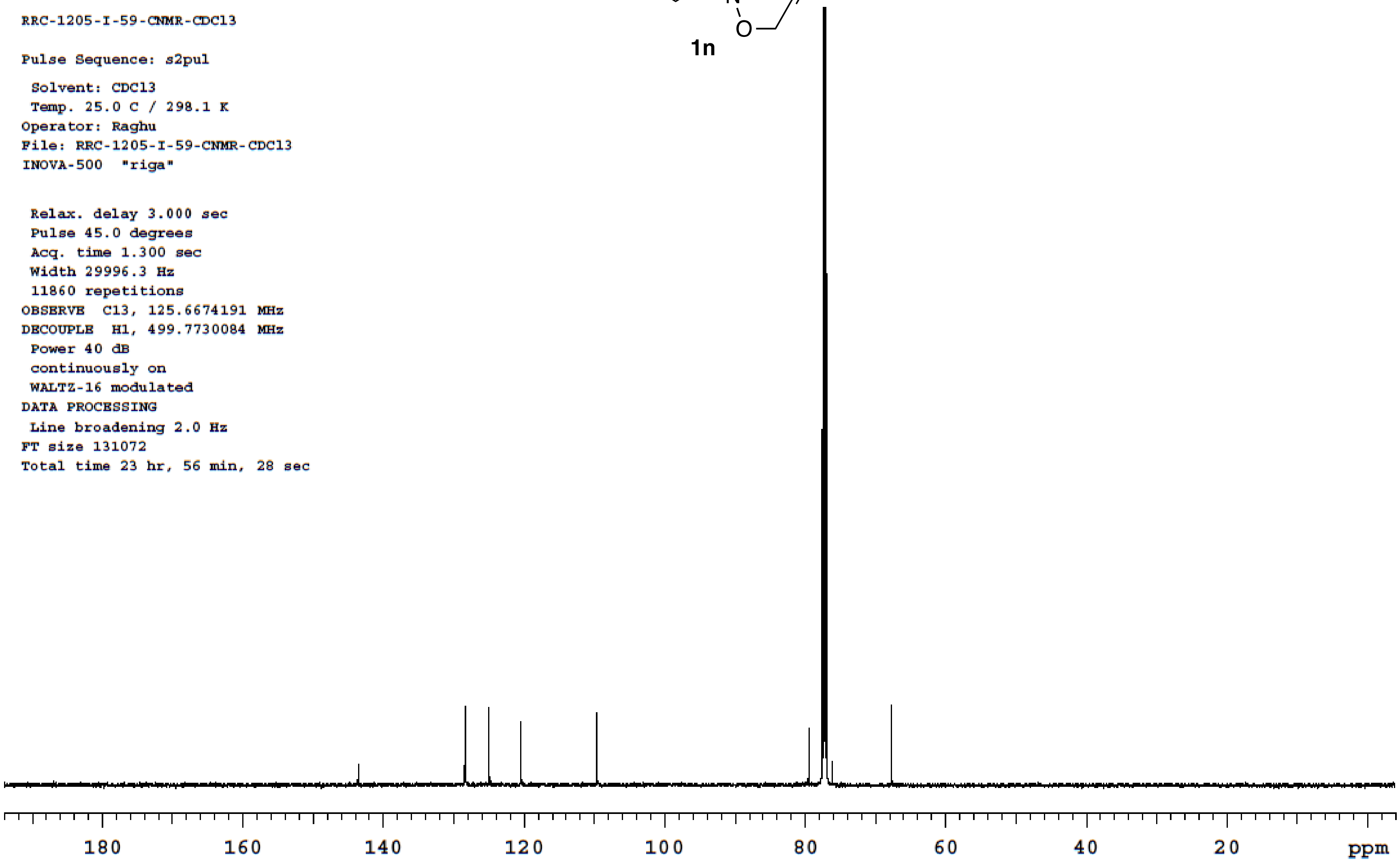

RRC-1205-I-59-CNMR-CDCl3

Pulse Sequence: s2pul

Solvent: CDCl3
Temp. 25.0 C / 298.1 K
Operator: Raghu
File: RRC-1205-I-59-CNMR-CDCl3
INOVA-500 "riga"

Relax. delay 3.000 sec
Pulse 45.0 degrees
Acq. time 1.300 sec
Width 29996.3 Hz
11860 repetitions
OBSERVE C13, 125.6674191 MHz
DECOUPLE H1, 499.7730084 MHz
Power 40 dB
continuously on
WALTZ-16 modulated
DATA PROCESSING
Line broadening 2.0 Hz
FT size 131072
Total time 23 hr, 56 min, 28 sec

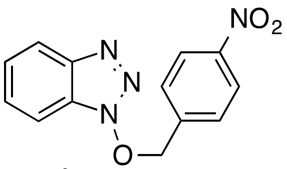

1o

MKS-1205-12-17-CDCl3-pureFrac-2ndCC

Pulse Sequence: s2pul

Solvent: cdcl3
Temp. 25.0 C / 298.1 K
Operator: mkl
File: MKS-1205-12-17-CDCl3-pureFrac-2ndCC
INOVA-500 "riga"

Pulse 45.0 degrees
Acq. time 1.892 sec
Width 8000.0 Hz
60 repetitions
OBSERVE H1, 499.7707217 MHz
DATA PROCESSING
FT size 32768
Total time 6 min, 20 sec

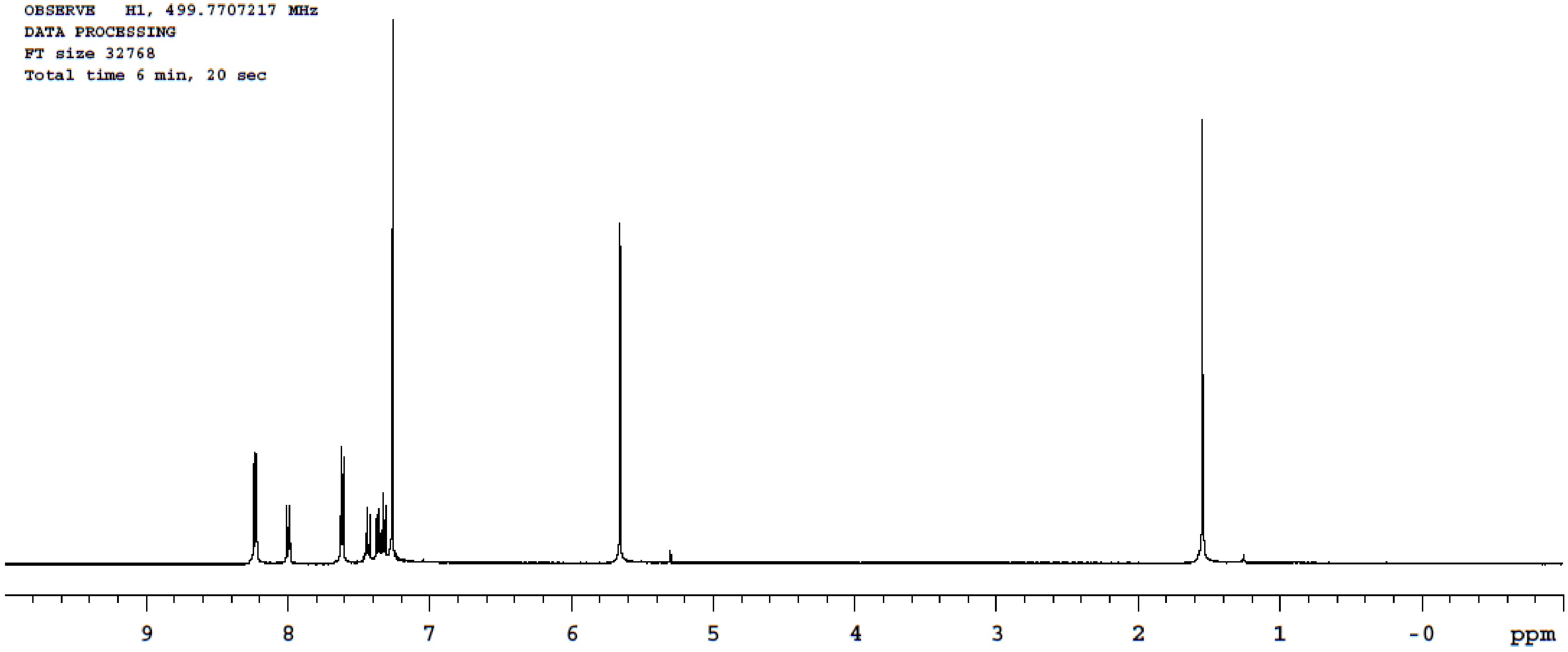

MKS-1205-12-17-CDCl3-13C-CC-pure

Pulse Sequence: s2pul

Solvent: CDCl3
Temp. 25.0 C / 298.1 K
Operator: mkl
File: MKS-1205-12-17-CDCl3-13C-CC-pure
INOVA-500 "riga"

Relax. delay 4.000 sec
Pulse 52.1 degrees
Acq. time 1.300 sec
Width 29996.3 Hz
10632 repetitions
OBSERVE C13, 125.6674200 MHz
DECOUPLE H1, 499.7732084 MHz
Power 42 dB
on during acquisition
WALTZ-16 modulated
DATA PROCESSING
Line broadening 2.0 Hz
FT size 131072
Total time 294 hr, 58 min, 21 sec

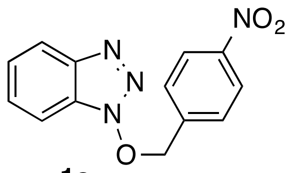

1o

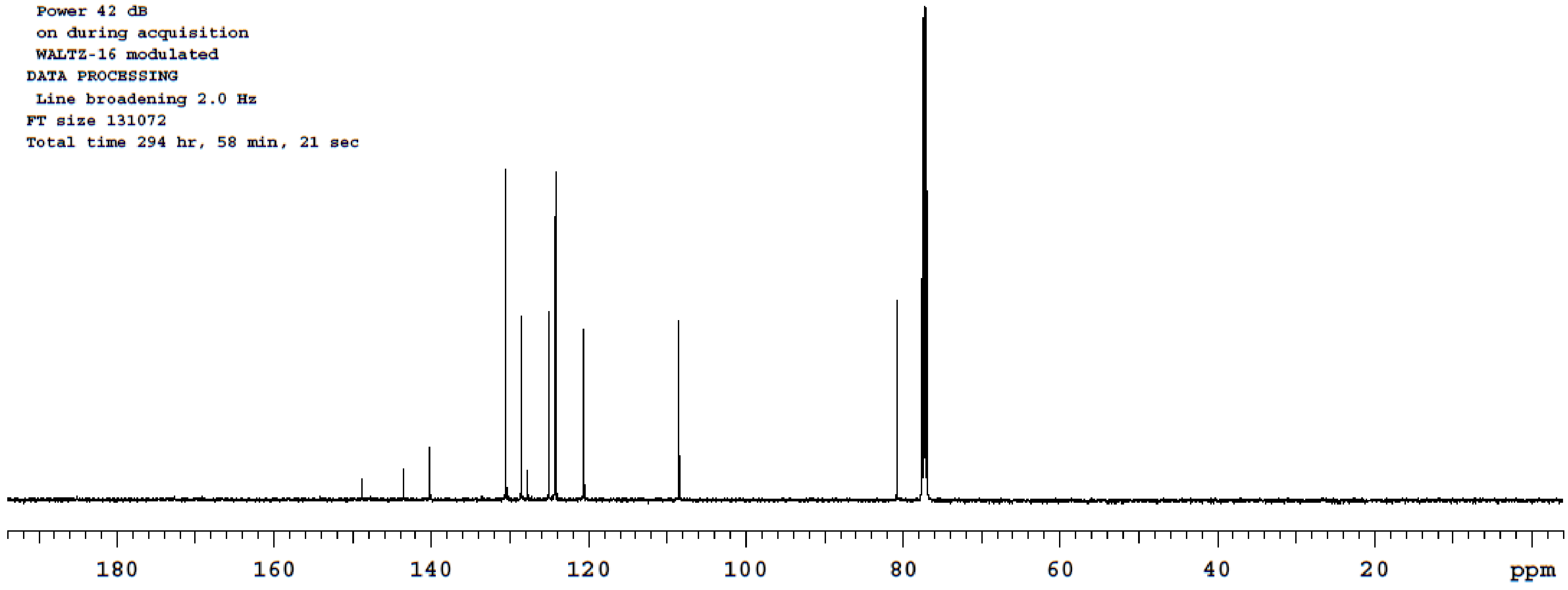

MKS-1205-13-54-CDCl3-CC

Pulse Sequence: s2pul

Solvent: cdcl3
Temp. 24.0 C / 297.1 K
Operator: mkl
File: MKS-1205-13-54-CDCl3-CC
INOVA-500 "riga"

Pulse 45.0 degrees
Acq. time 1.892 sec
Width 8000.0 Hz
56 repetitions
OBSERVE H1, 499.7707212 MHz
DATA PROCESSING
Line broadening 0.2 Hz
FT size 32768
Total time 1 hr, 3 min, 21 sec

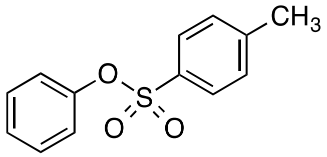

1p

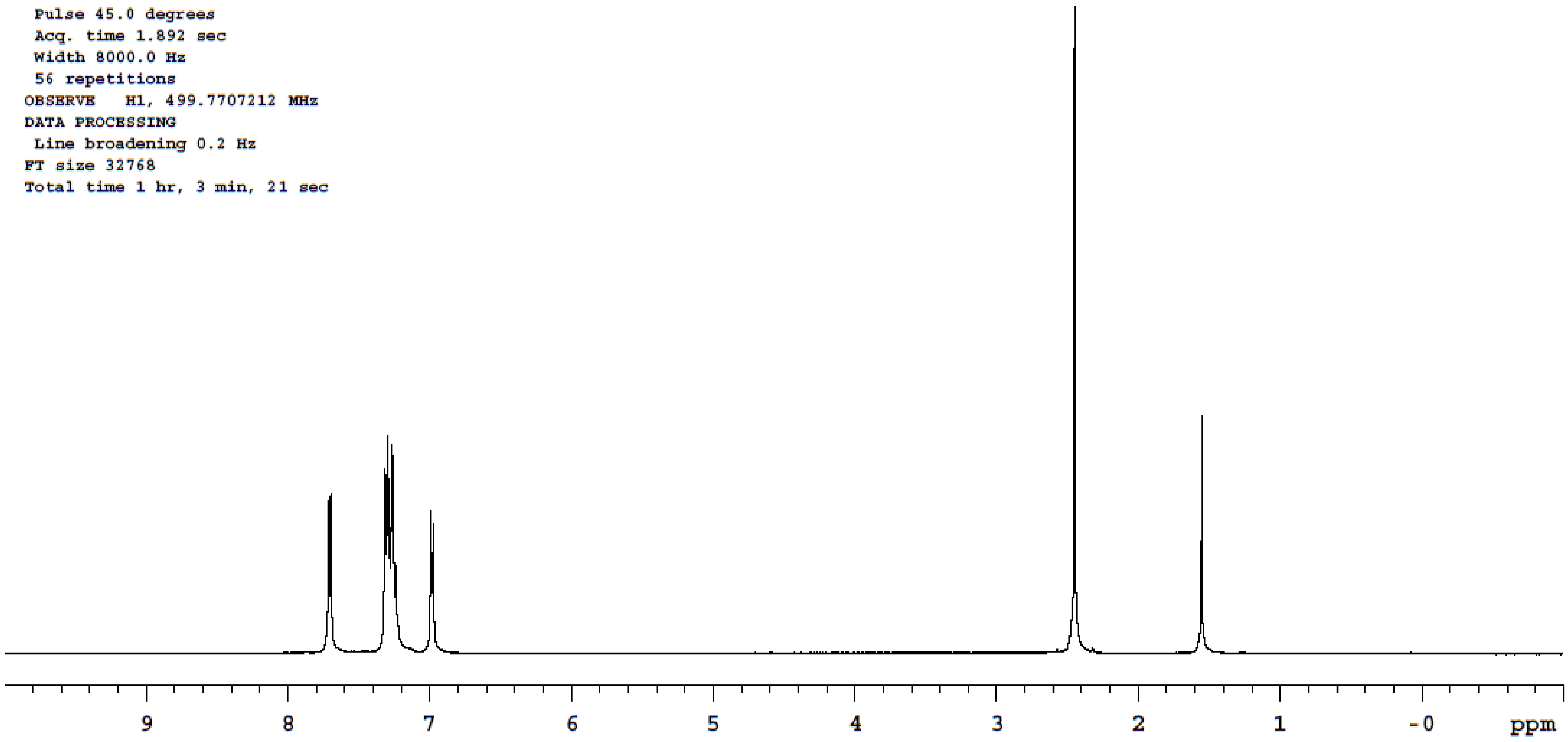

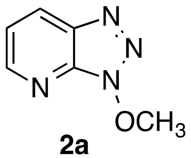

2a

MKS-1205-13-58-CDCl3-CC

Pulse Sequence: s2pul

Solvent: cdcl3
Temp. 24.0 C / 297.1 K
Operator: mkl
File: MKS-1205-13-58-CDCl3-CC
INOVA-500 "riga"

Pulse 45.0 degrees
Acq. time 1.892 sec
Width 8000.0 Hz
76 repetitions
OBSERVE H1, 499.7707212 MHz
DATA PROCESSING
Line broadening 0.2 Hz
FT size 32768
Total time 1 hr, 3 min, 21 sec

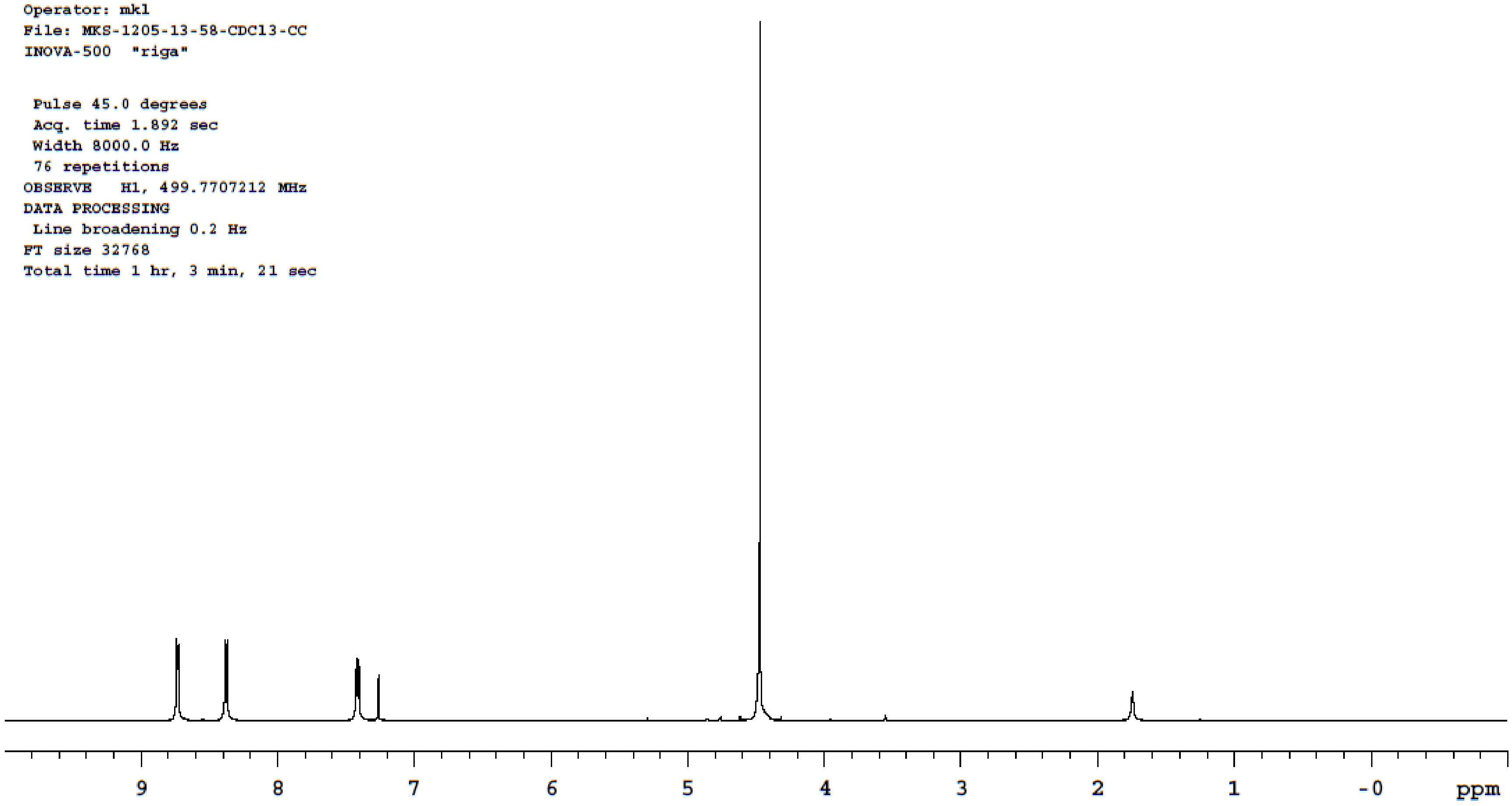

MKS-1205-13-58-CDCl3-13C-CC-rep

Pulse Sequence: s2pul

Solvent: CDCl3
Temp. 24.0 C / 297.1 K
Operator: mkl
File: MKS-1205-13-58-CDCl3-13C-CC-rep
INOVA-500 "riga"

Relax. delay 3.000 sec
Pulse 45.0 degrees
Acq. time 1.300 sec
Width 25000.0 Hz
2048 repetitions
OBSERVE C13, 125.6674202 MHz
DECOUPLE H1, 499.7730084 MHz
Power 39 dB
continuously on
WALTZ-16 modulated
DATA PROCESSING
Line broadening 0.2 Hz
FT size 65536
Total time 2 hr, 27 min, 5 sec

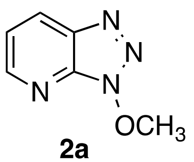

2a

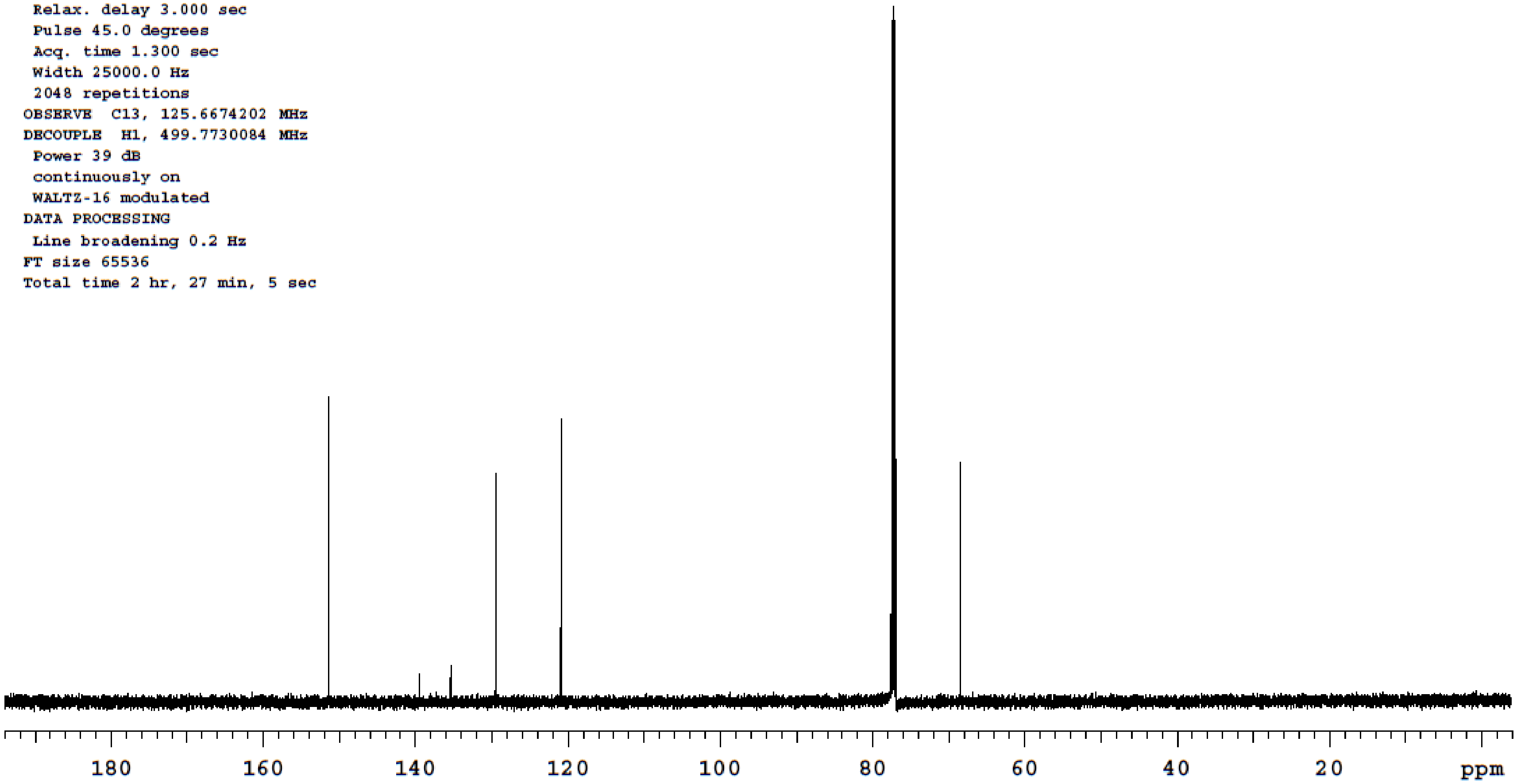

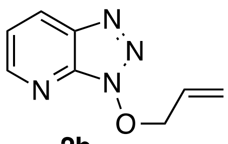

2b

mk-1205-I-HNMR-CDCL3-28-allyl

Pulse Sequence: s2pul

Solvent: CDCl3
Ambient temperature
Operator: mkl
File: mk-1205-I-HNMR-CDCL3-28-allyl
INOVA-500 "riga"

Pulse 38.6 degrees
Acq. time 1.892 sec
Width 8000.0 Hz
16 repetitions
OBSERVE H1, 499.7707212 MHz
DATA PROCESSING
FT size 32768
Total time 0 min, 30 sec

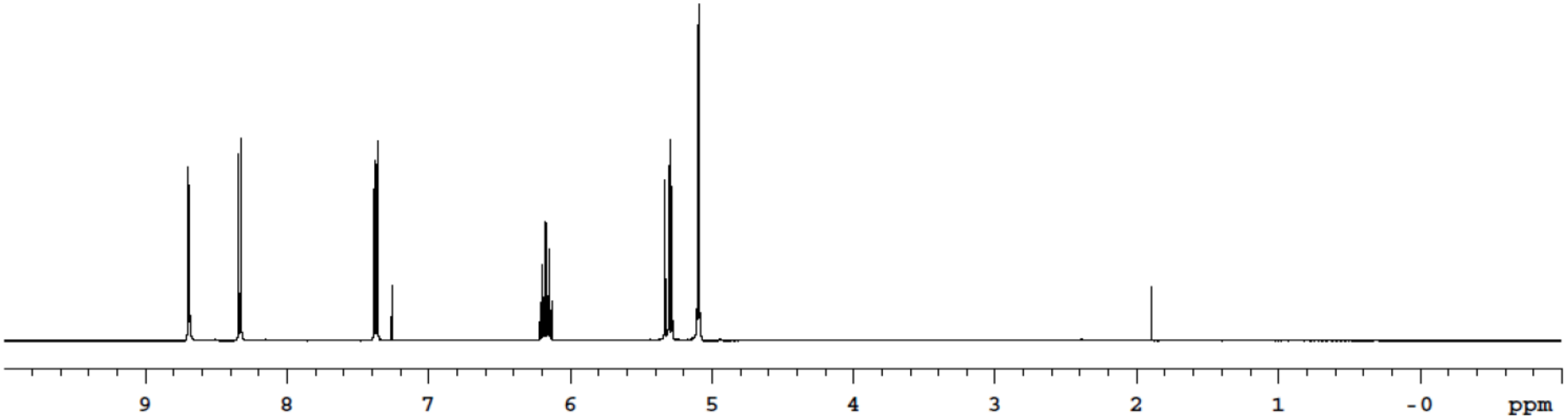

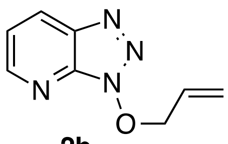

2b

mk-1205-I-C13-CDCl3-28-allyl

Pulse Sequence: s2pul

Solvent: CDCl3
Ambient temperature
Operator: mkl
File: mk-1205-I-C13-CDCl3-28-allyl
INOVA-500 "riga"

Pulse 45.0 degrees
Acq. time 1.300 sec
Width 25000.0 Hz
500 repetitions
OBSERVE C13, 125.6674355 MHz
DECOUPLE H1, 499.7730084 MHz
Power 39 dB
continuously on
WALTZ-16 modulated
DATA PROCESSING
Line broadening 0.5 Hz
FT size 65536
Total time 7 hr, 16 min, 27 sec

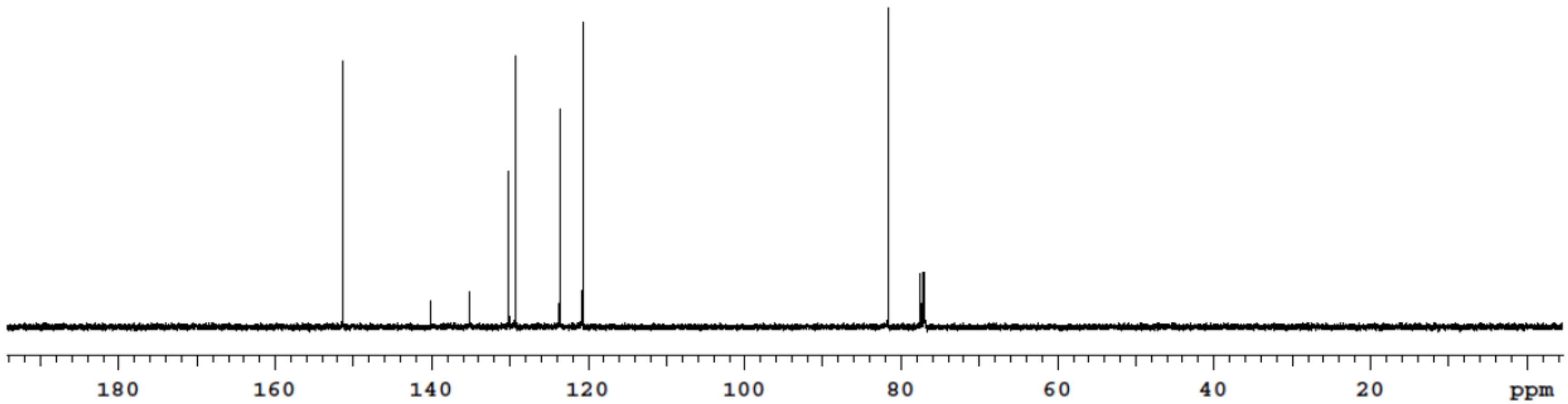

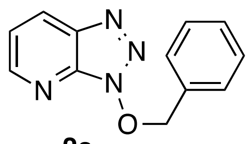

2c

```
mk-1205-I-HNMR-CDCL3-120-benzylazabt

Pulse Sequence: s2pul

 Solvent: CDCl3
 Ambient temperature
Operator: mkl
File: mk-1205-I-HNMR-CDCL3-120-benzylazabt
INOVA-500  "riga"

 Pulse 34.7 degrees
 Acq. time 1.892 sec
 Width 8000.0 Hz
 64 repetitions
OBSERVE   H1, 499.7707207 MHz
DATA PROCESSING
FT size 32768
Total time 2 min, 1 sec
```

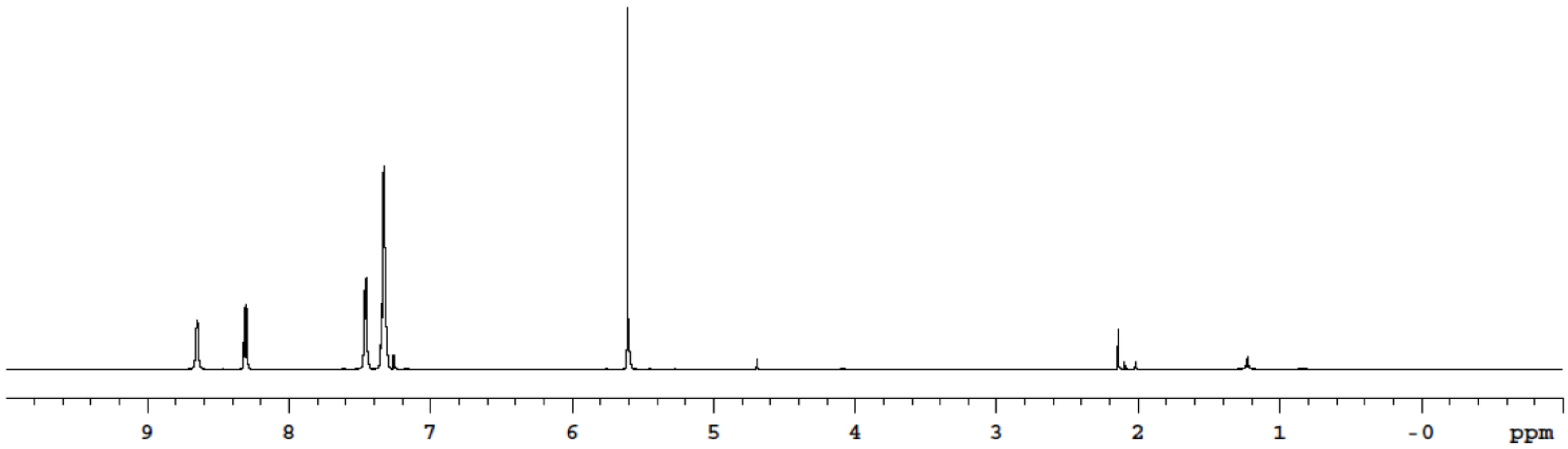

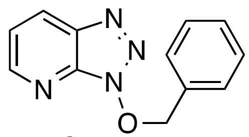

2c

```
mk-1205-I-C13-CDCL3-120-benzylaza

Pulse Sequence: s2pul

 Solvent: CDCl3
 Ambient temperature
Operator: mkl
File: mk-1205-I-C13-CDCL3-120-benzylaza
INOVA-500  "riga"

 Pulse 45.0 degrees
 Acq. time 1.300 sec
 Width 25000.0 Hz
 2064 repetitions
OBSERVE  C13, 125.6674332 MHz
DECOUPLE  H1, 499.7730084 MHz
 Power 39 dB
 continuously on
 WALTZ-16 modulated
DATA PROCESSING
 Line broadening 0.5 Hz
FT size 65536
Total time 1 hr, 49 min, 6 sec
```

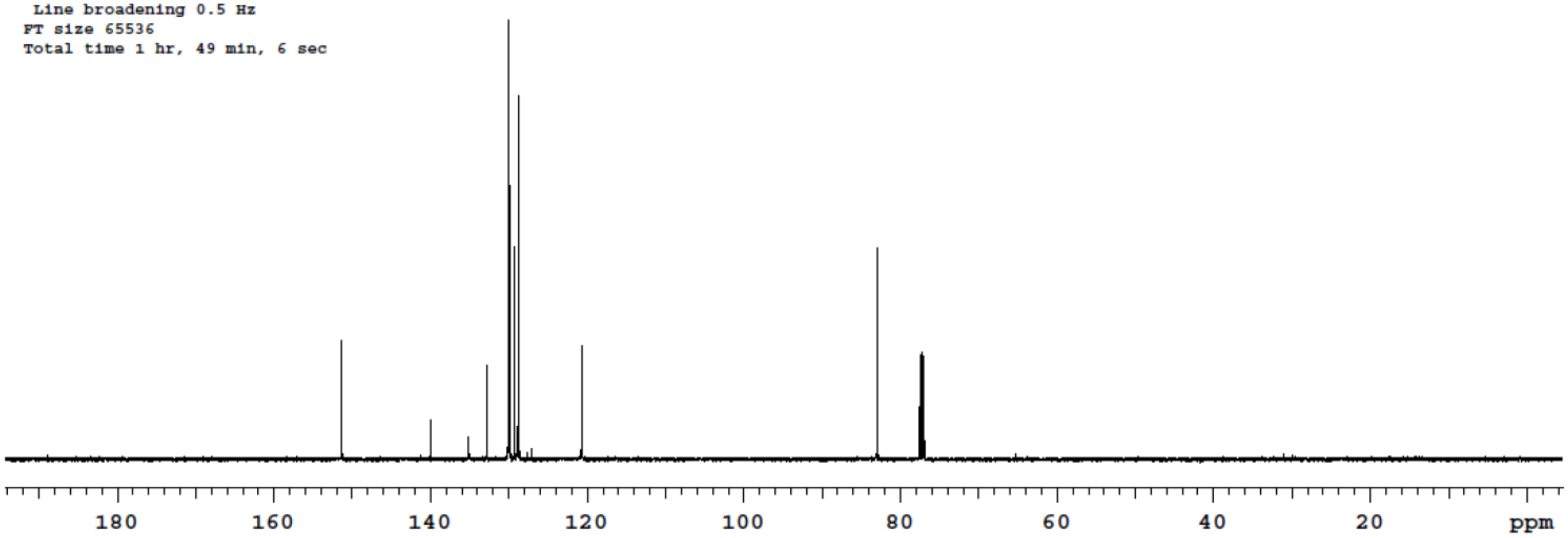

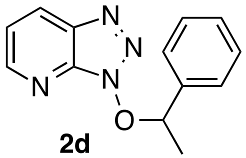

2d

mk-1205-I-HNMR-CDCL3-26-secphenyl

Pulse Sequence: s2pul

Solvent: CDCl3
Ambient temperature
Operator: mkl
File: mk-1205-I-HNMR-CDCL3-26-secphenyl
INOVA-500 "riga"

Pulse 34.7 degrees
Acq. time 1.892 sec
Width 8000.0 Hz
64 repetitions
OBSERVE H1, 499.7707246 MHz
DATA PROCESSING
FT size 32768
Total time 2 min, 1 sec

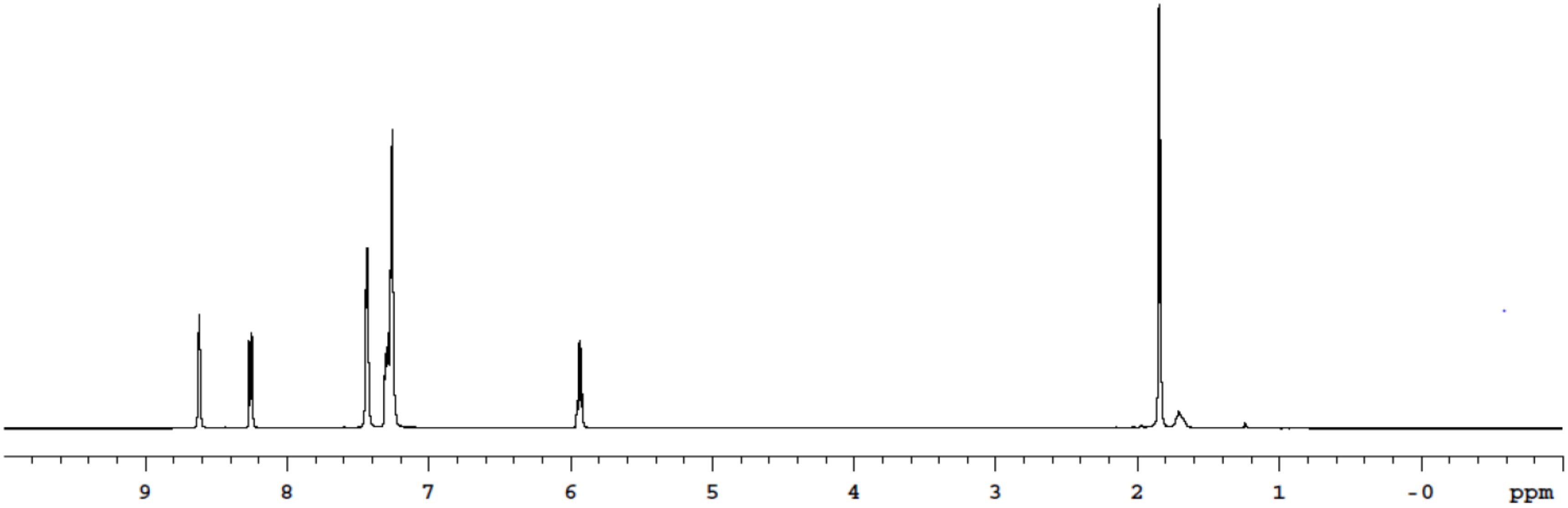

mk-1205-I-C13-CDCl3-26-secphenyl

Pulse Sequence: s2pul

Solvent: CDCl3
Ambient temperature
Operator: mkl
File: mk-1205-I-C13-CDCl3-26-secphenyl
INOVA-500 "riga"

Pulse 45.0 degrees
Acq. time 1.300 sec
Width 25000.0 Hz
2020 repetitions
OBSERVE C13, 125.6674263 MHz
DECOUPLE H1, 499.7730084 MHz
Power 39 dB
continuously on
WALTZ-16 modulated
DATA PROCESSING
Line broadening 0.5 Hz
FT size 65536
Total time 7 hr, 16 min, 27 sec

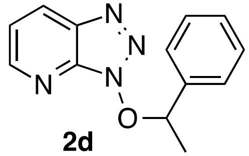

2d

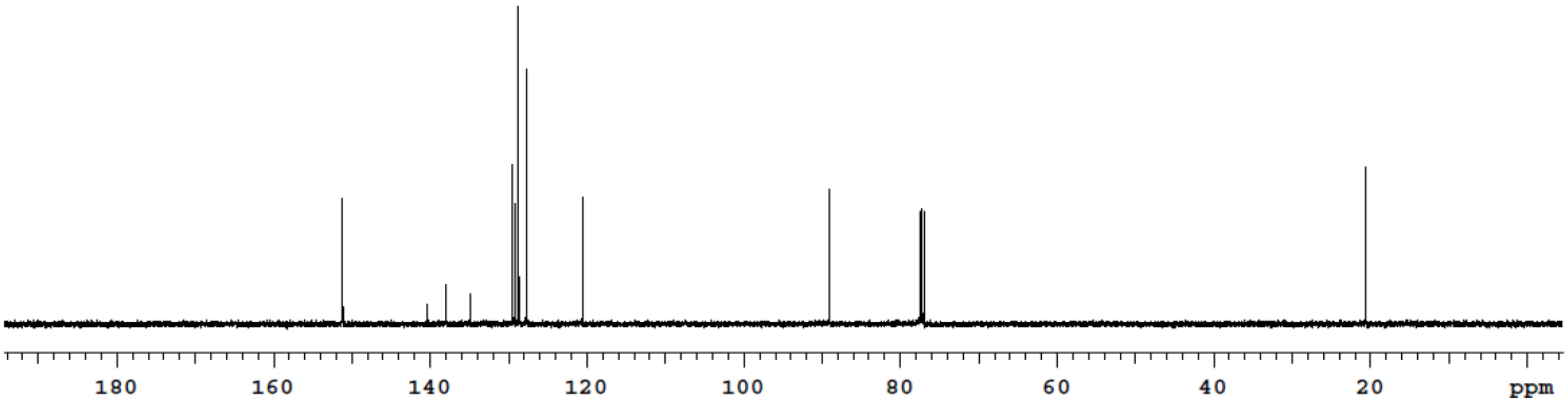

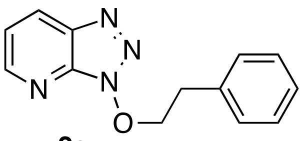

2e

mk-1205-I-HNMR-CDCL3-29-nphenyl

Pulse Sequence: s2pul

Solvent: CDCl3
Ambient temperature
Operator: mkl
File: mk-1205-I-HNMR-CDCL3-29-nphenyl
INOVA-500 "riga"

Pulse 38.6 degrees
Acq. time 1.892 sec
Width 8000.0 Hz
64 repetitions
OBSERVE H1, 499.7707212 MHz
DATA PROCESSING
FT size 32768
Total time 2 min, 1 sec

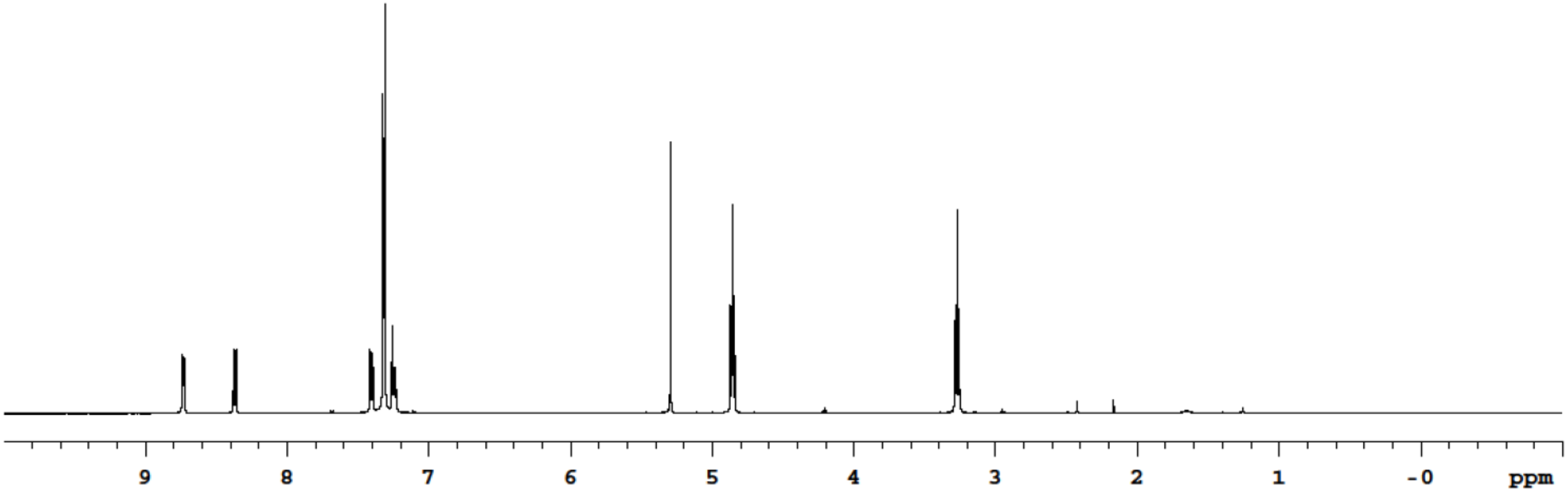

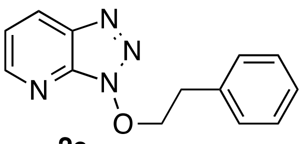

2e

mk-1205-I-C13-CDCl3-29-nphenyl

Pulse Sequence: s2pul

Solvent: CDCl3
Ambient temperature
Operator: mkl
File: mk-1205-I-C13-CDCl3-29-nphenyl
INOVA-500 "riga"

Pulse 45.0 degrees
Acq. time 1.300 sec
Width 25000.0 Hz
2050 repetitions
OBSERVE C13, 125.6674271 MHz
DECOUPLE H1, 499.7730084 MHz
Power 39 dB
continuously on
WALTZ-16 modulated
DATA PROCESSING
Line broadening 0.5 Hz
FT size 65536
Total time 7 hr, 16 min, 27 sec

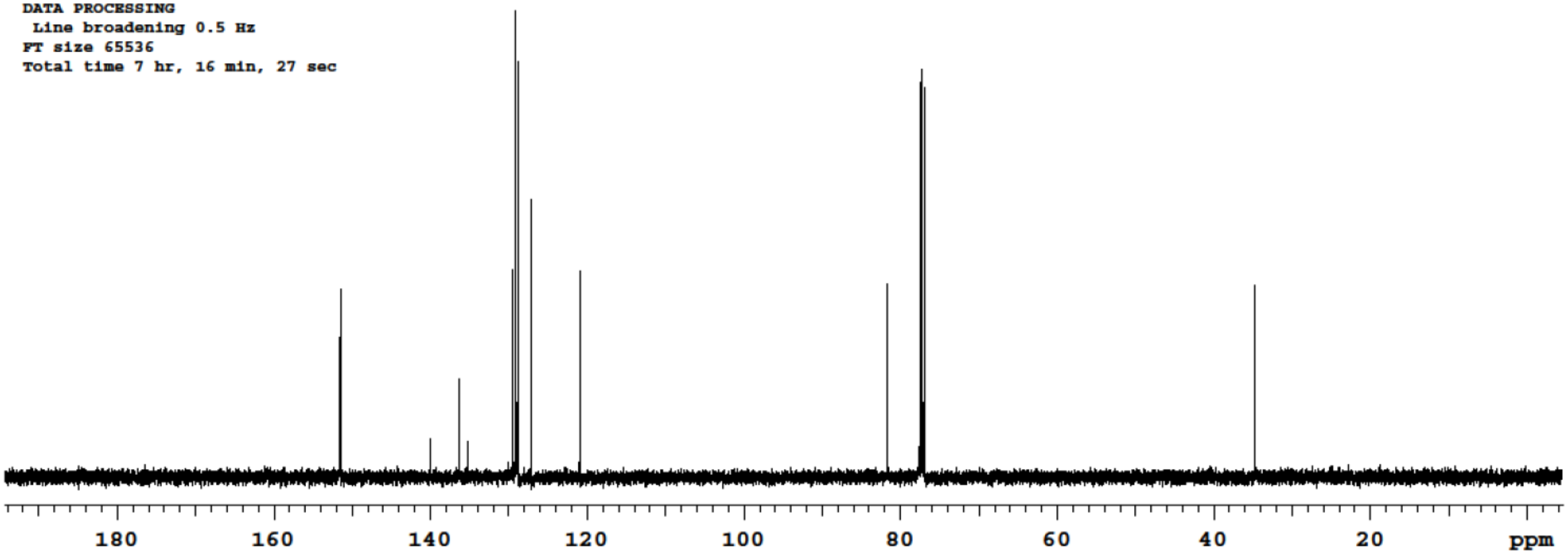

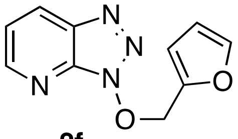

2f

mk-1205-I-HNMR-CDCL3-121-furfurylaza

Pulse Sequence: s2pul

Solvent: CDCl3
Ambient temperature
Operator: mkl
File: mk-1205-I-HNMR-CDCL3-121-furfurylaza
INOVA-500 "riga"

Pulse 34.7 degrees
Acq. time 1.892 sec
Width 8000.0 Hz
64 repetitions
OBSERVE H1, 499.7707202 MHz
DATA PROCESSING
FT size 32768
Total time 2 min, 1 sec

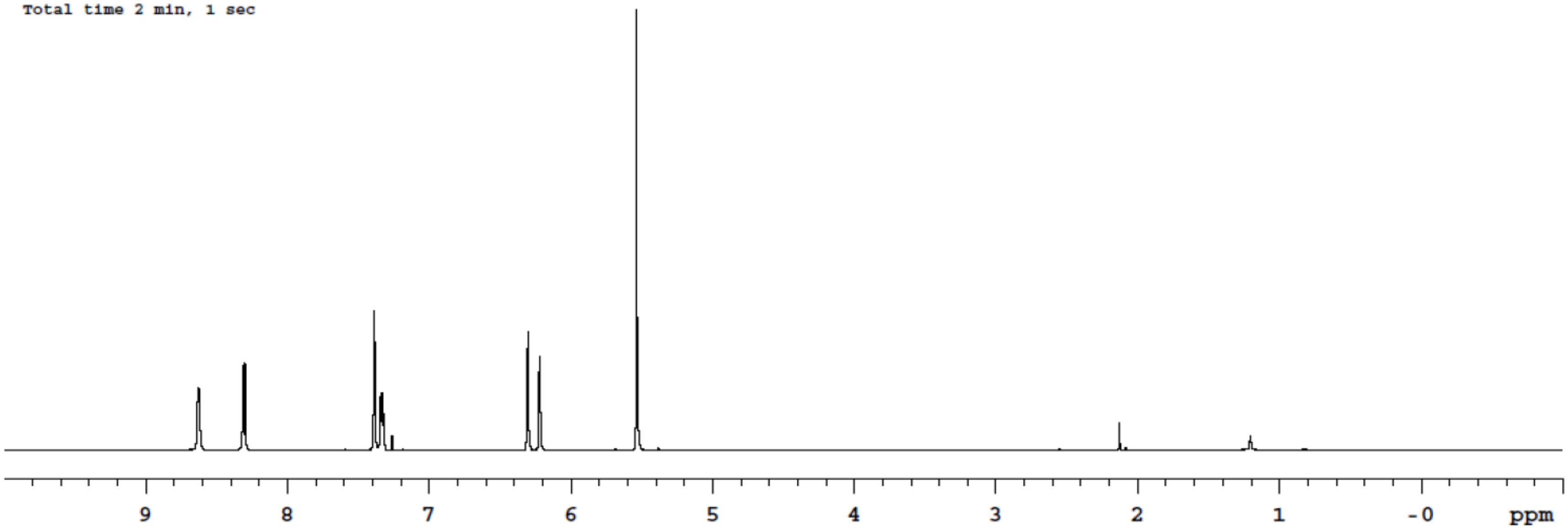

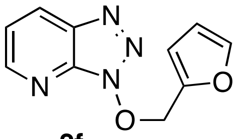

2f

mk-1205-I-C13-CDCL3-121-furfurylaza

Pulse Sequence: s2pul

Solvent: CDCl3
Ambient temperature
Operator: mkl
File: mk-1205-I-C13-CDCL3-121-furfurylaza
INOVA-500 "riga"

Pulse 45.0 degrees
Acq. time 1.300 sec
Width 25000.0 Hz
355 repetitions
OBSERVE C13, 125.6674339 MHz
DECOUPLE H1, 499.7730084 MHz
Power 39 dB
continuously on
WALTZ-16 modulated
DATA PROCESSING
Line broadening 0.5 Hz
FT size 65536
Total time 1 hr, 49 min, 6 sec

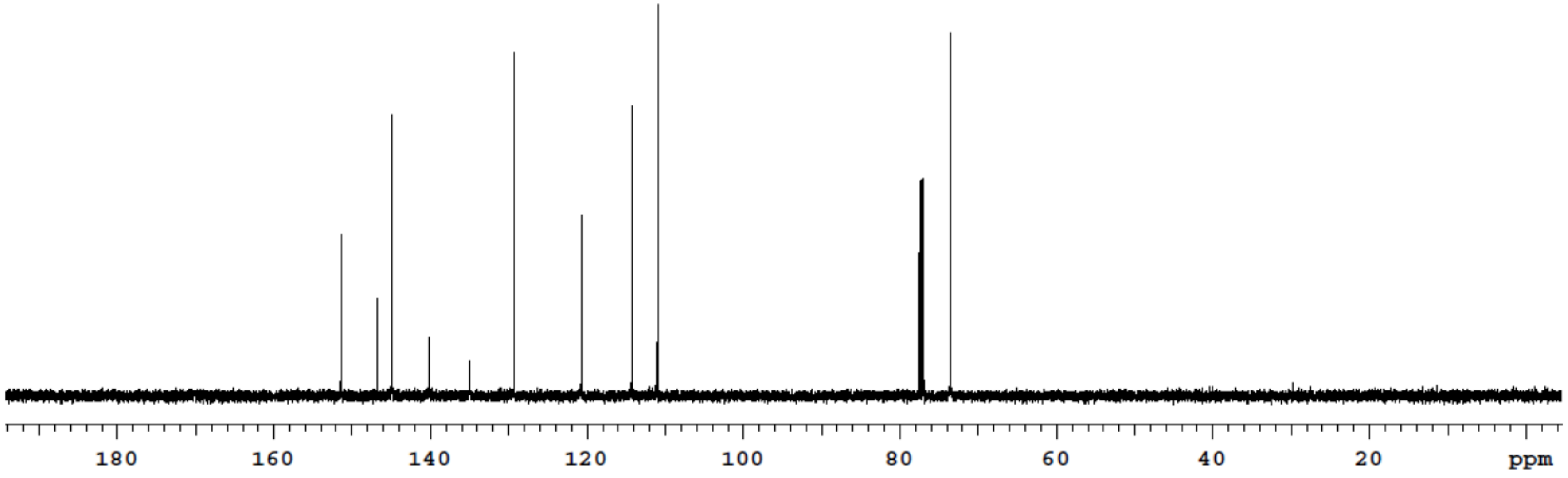

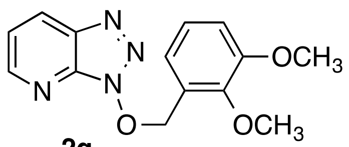

2g

mk-1205-I-HNMR-CDCL3-27-diether

Pulse Sequence: s2pul

Solvent: CDCl3
Ambient temperature
Operator: mkl
File: mk-1205-I-HNMR-CDCL3-27-diether
INOVA-500 "riga"

Pulse 38.6 degrees
Acq. time 1.892 sec
Width 8000.0 Hz
4 repetitions
OBSERVE H1, 499.7707207 MHz
DATA PROCESSING
FT size 32768
Total time 0 min, 7 sec

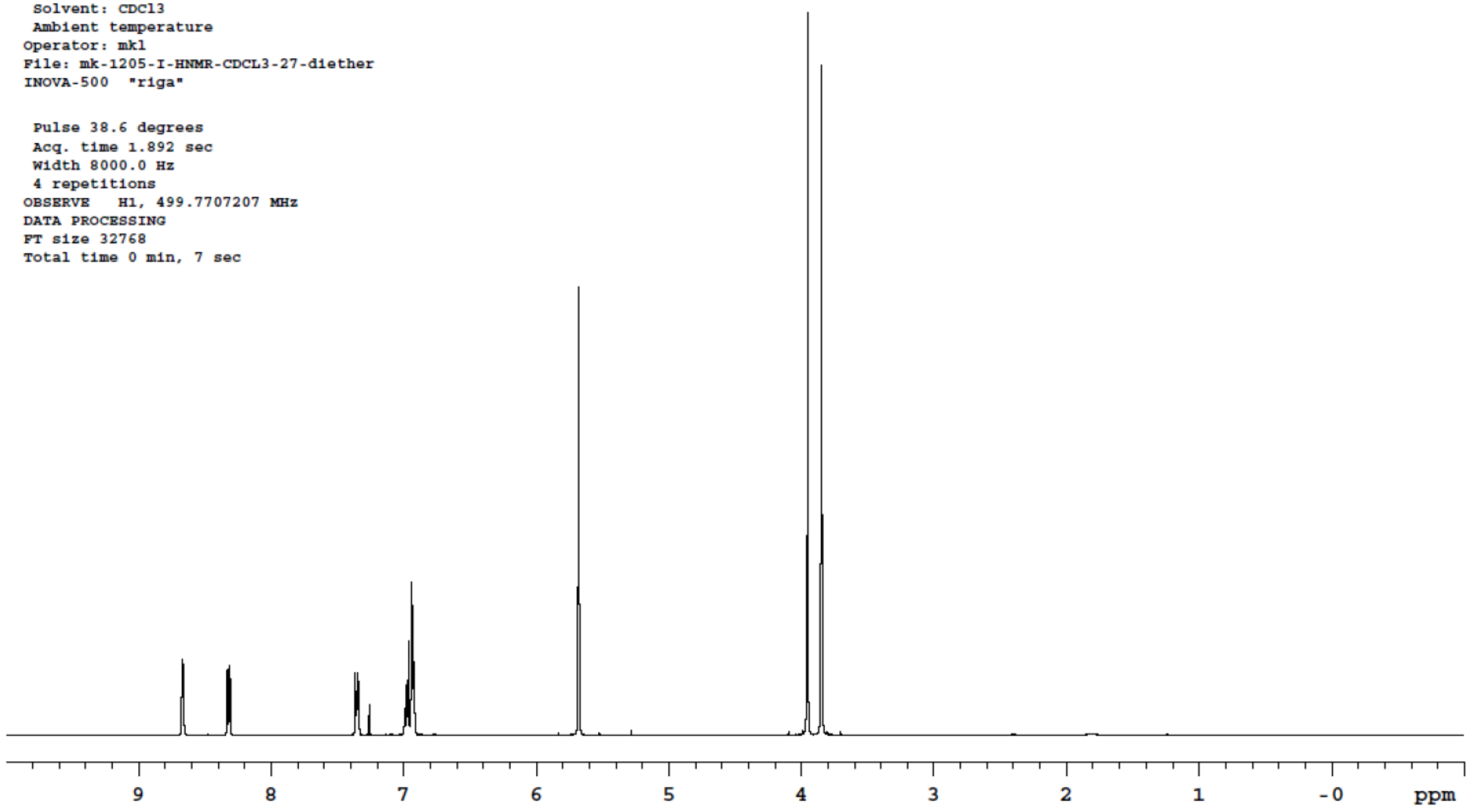

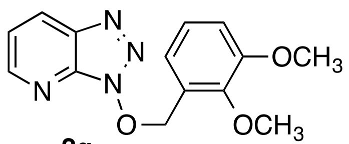

2g

```
mk-1205-I-C13-CDCL3-39-diether

Pulse Sequence: s2pul
 Solvent: CDCl3
 Ambient temperature
Operator: mkl
File: mk-1205-I-C13-CDCL3-39-diether
INOVA-500  "riga"

 Pulse 45.0 degrees
 Acq. time 1.300 sec
 Width 25000.0 Hz
 1280 repetitions
OBSERVE  C13, 125.6674255 MHz
DECOUPLE  H1, 499.7730084 MHz
 Power 39 dB
 continuously on
 WALTZ-16 modulated
DATA PROCESSING
 Line broadening 0.5 Hz
FT size 65536
Total time 7 hr, 16 min, 27 sec
```

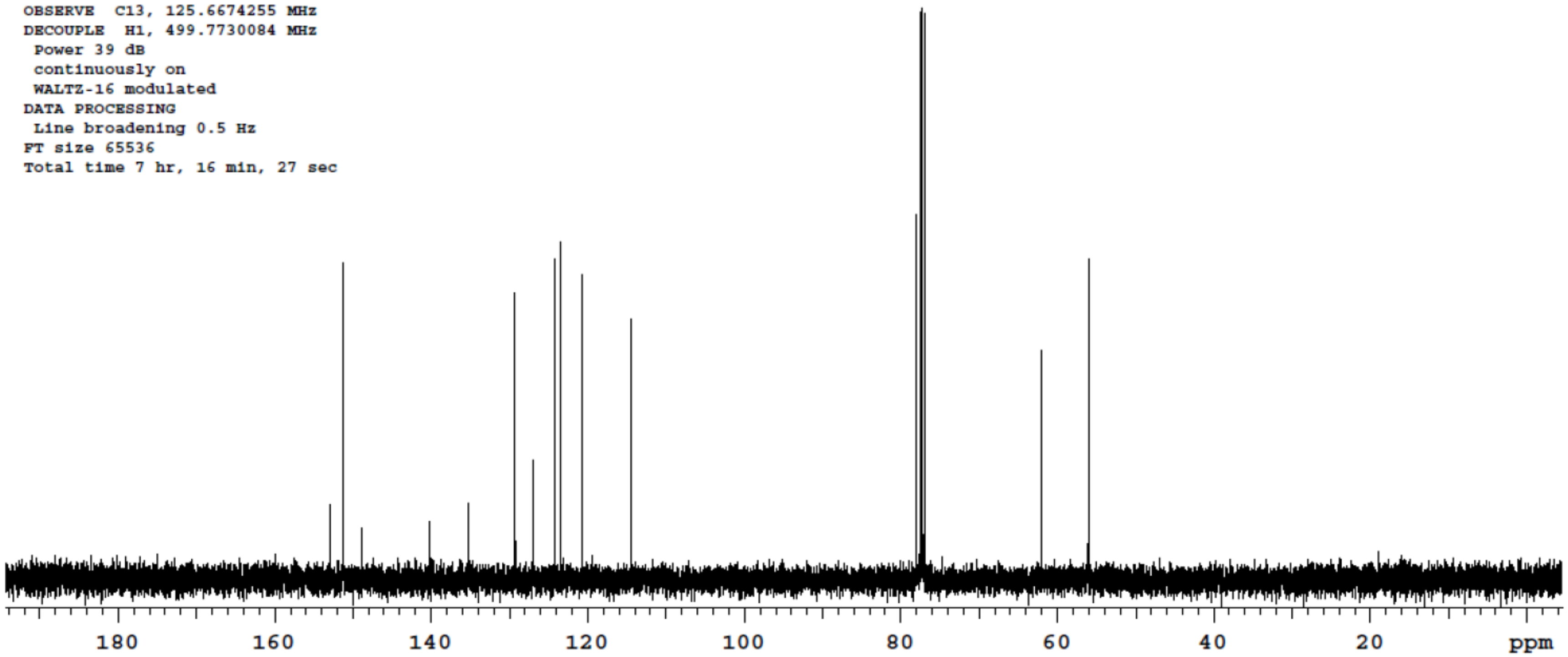

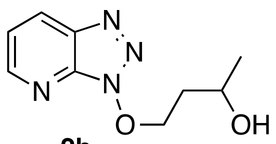

2h

mk-1205-I-HNMR-CDCL3-36-1.3butanediol

Pulse Sequence: s2pul

Solvent: CDCl3
Ambient temperature
Operator: mkl
File: mk-1205-I-HNMR-CDCL3-36-1.3butanediol
INOVA-500 "riga"

Pulse 34.7 degrees
Acq. time 1.892 sec
Width 8000.0 Hz
64 repetitions
OBSERVE H1, 499.7707217 MHz
DATA PROCESSING
FT size 32768
Total time 2 min, 1 sec

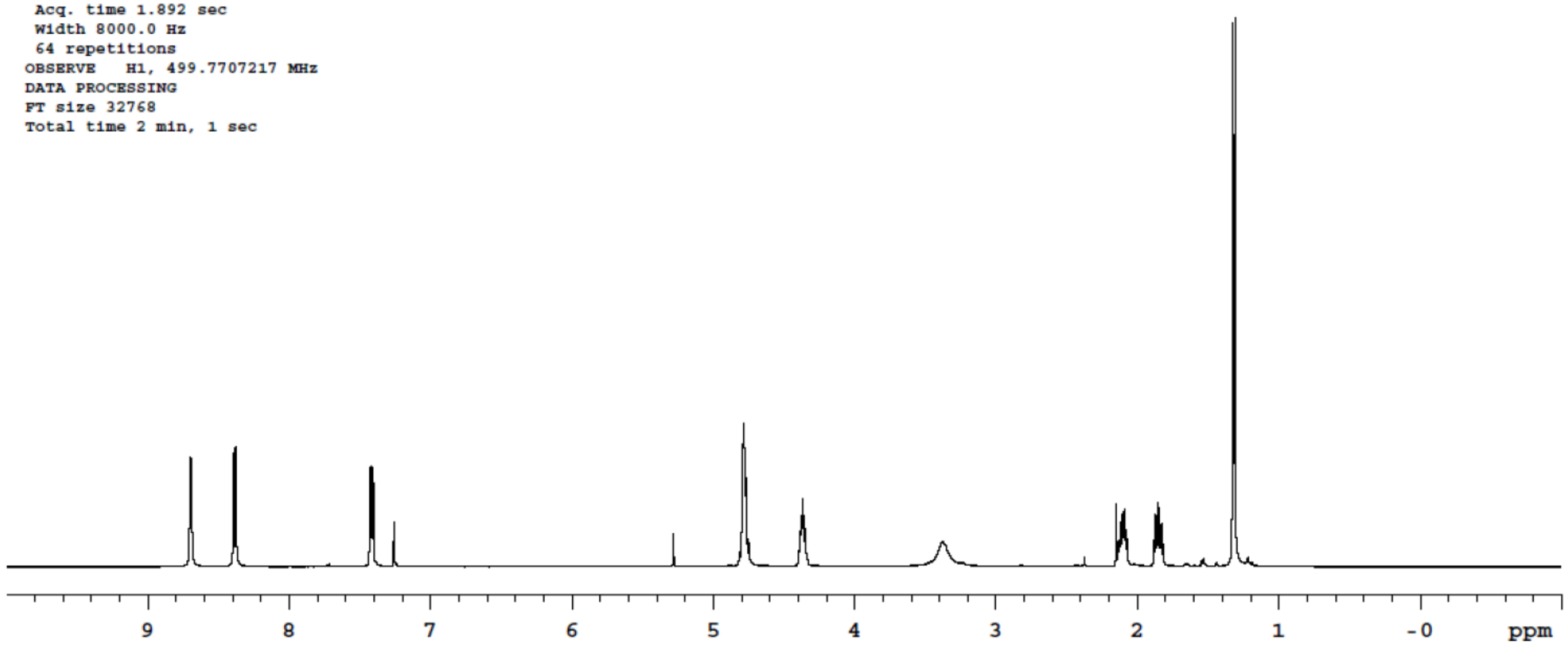

STANDARD PROTON PARAMETERS

Pulse Sequence: s2pul

Solvent: CDCl3
Ambient temperature
Operator: mkl
File: mk-1205-I-HNMR-CDCL3-36-1.3butanediol-retaken08152012
INOVA-500 "riga"

Pulse 34.7 degrees
Acq. time 1.892 sec
Width 8000.0 Hz
64 repetitions
OBSERVE H1, 499.7707095 MHz
DATA PROCESSING
FT size 32768
Total time 2 min, 1 sec

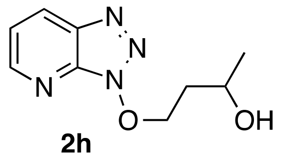

2h

Expanded spectrum

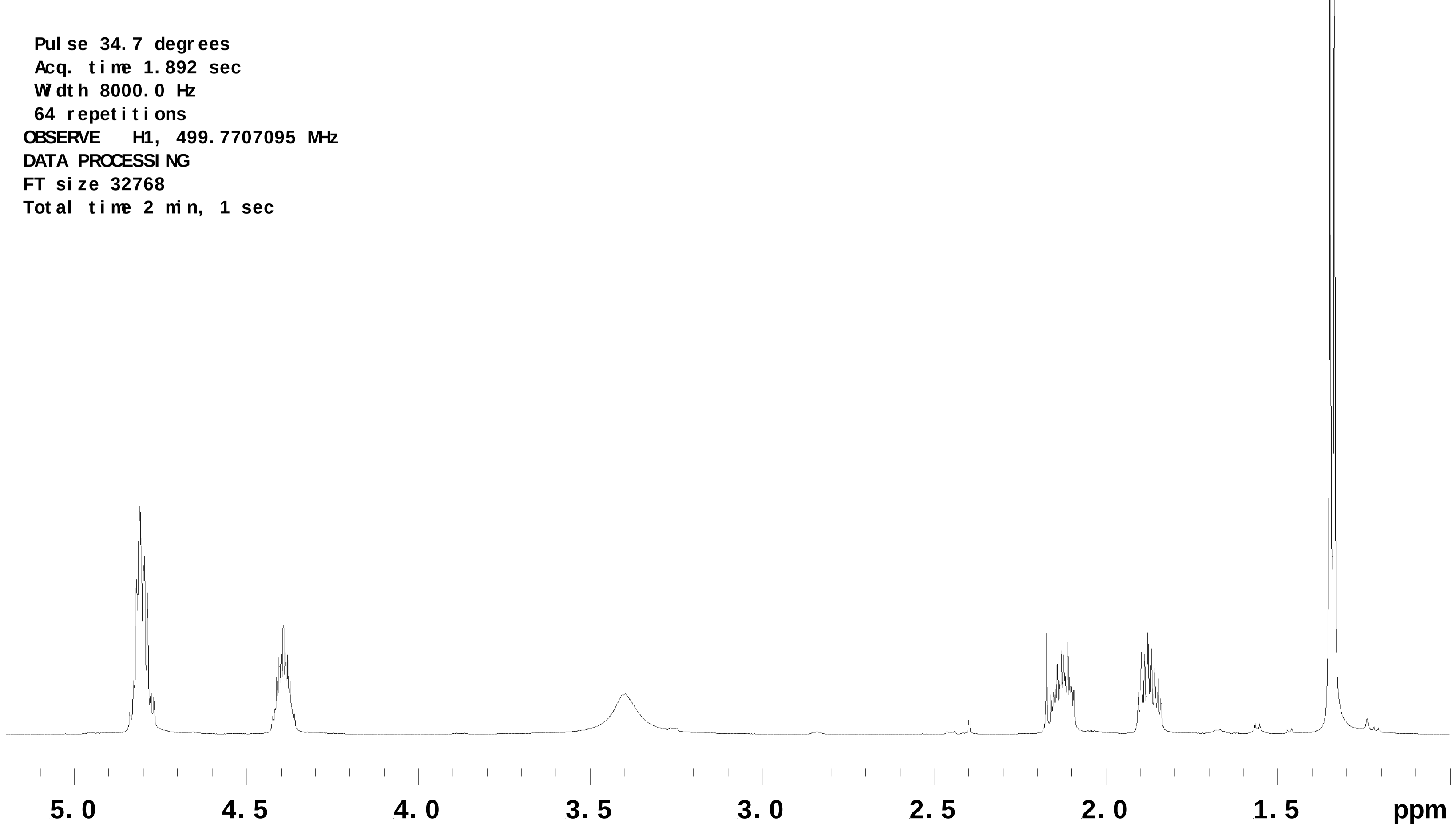

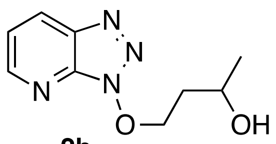

2h

mk-1205-I-C13-CDCL3-36-1.3butanediol

Pulse Sequence: s2pul

Solvent: CDCl3
Ambient temperature
Operator: mkl
File: mk-1205-I-C13-CDCL3-36-1.3butanediol
INOVA-500 "riga"

Pulse 45.0 degrees
Acq. time 1.300 sec
Width 25000.0 Hz
1390 repetitions
OBSERVE C13, 125.6674286 MHz
DECOUPLE H1, 499.7730084 MHz
Power 39 dB
continuously on
WALTZ-16 modulated
DATA PROCESSING
Line broadening 0.5 Hz
FT size 65536
Total time 7 hr, 16 min, 27 sec

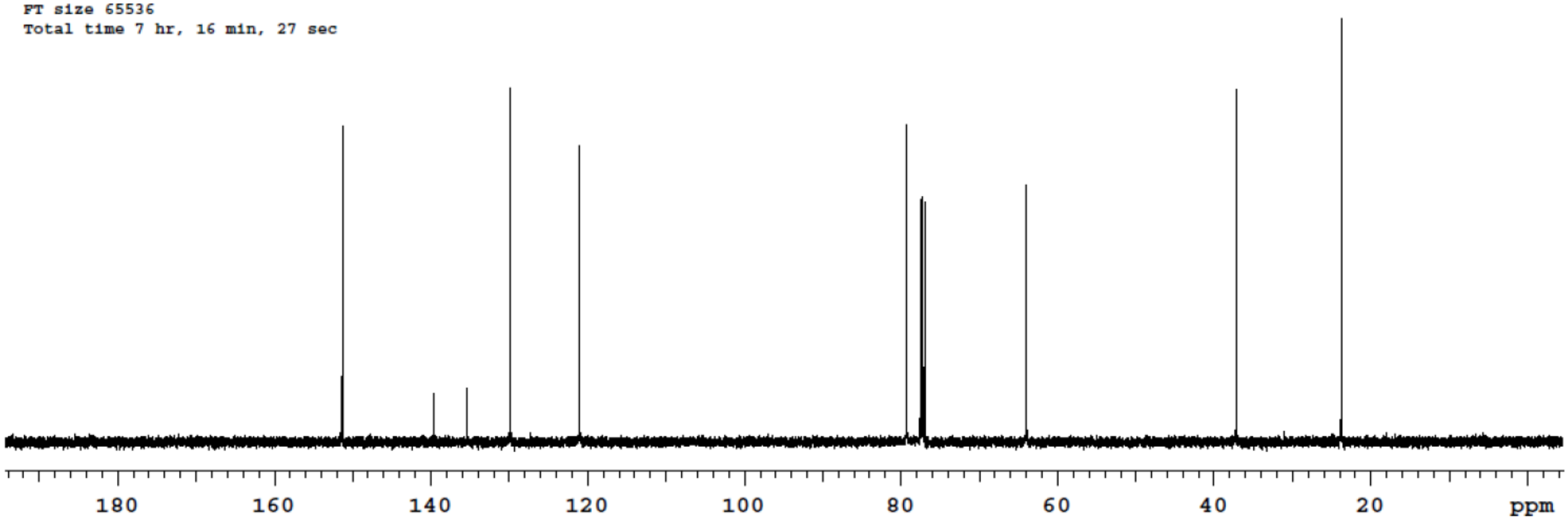

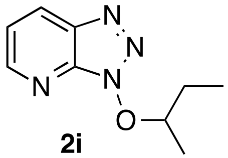

2i

```
mk-1205-I-HNMR-CDCL3-122-aza2butanol

Pulse Sequence: s2pul
 Solvent: CDCl3
 Ambient temperature
Operator: mkl
File: mk-1205-I-HNMR-CDCL3-122-aza2butanol
INOVA-500  "riga"

 Pulse 34.7 degrees
 Acq. time 1.892 sec
 Width 8000.0 Hz
 64 repetitions
OBSERVE   H1, 499.7707212 MHz
DATA PROCESSING
FT size 32768
Total time 2 min, 1 sec
```

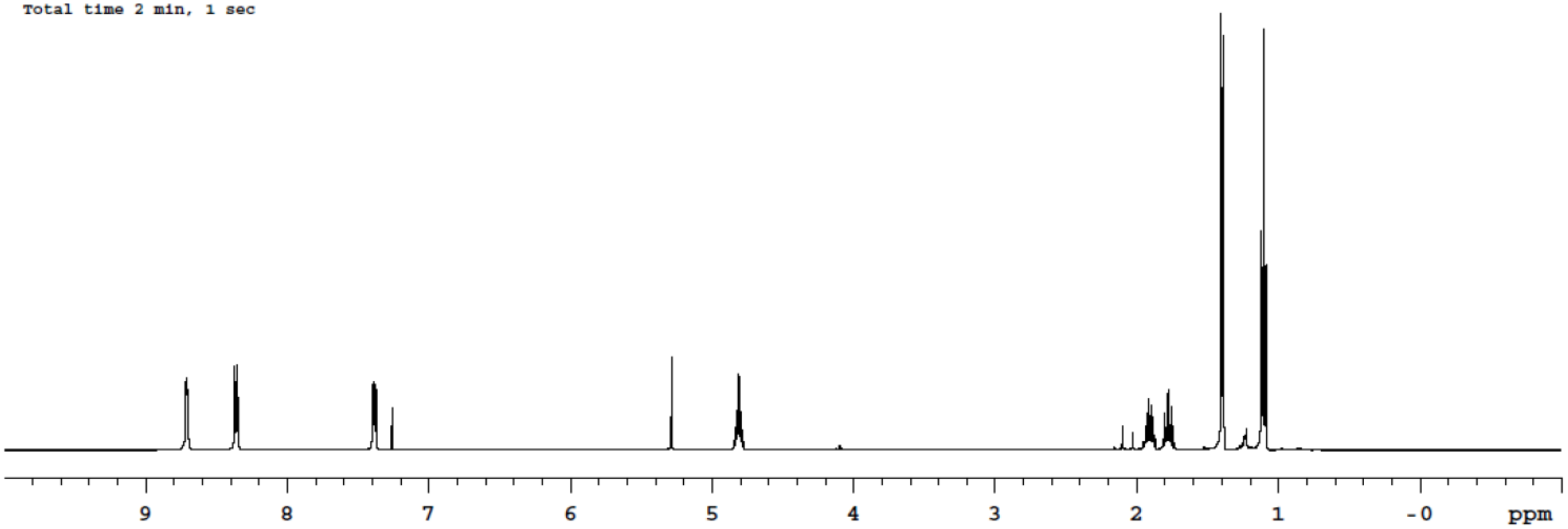

mk-1205-I-HNMR-CDCL3-122-aza2butanol

Pulse Sequence: s2pul

Solvent: CDCl3
Ambient temperature
Operator: mkl
File: mk-1205-I-HNMR-CDCL3-122-aza2butanol
INOVA-500 "riga"

Pulse 34.7 degrees
Acq. time 1.892 sec
Width 8000.0 Hz
64 repetitions
OBSERVE H1, 499.7707212 MHz
DATA PROCESSING
FT size 32768
Total time 2 min, 1 sec

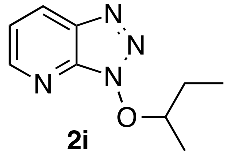

2i

Expanded spectrum

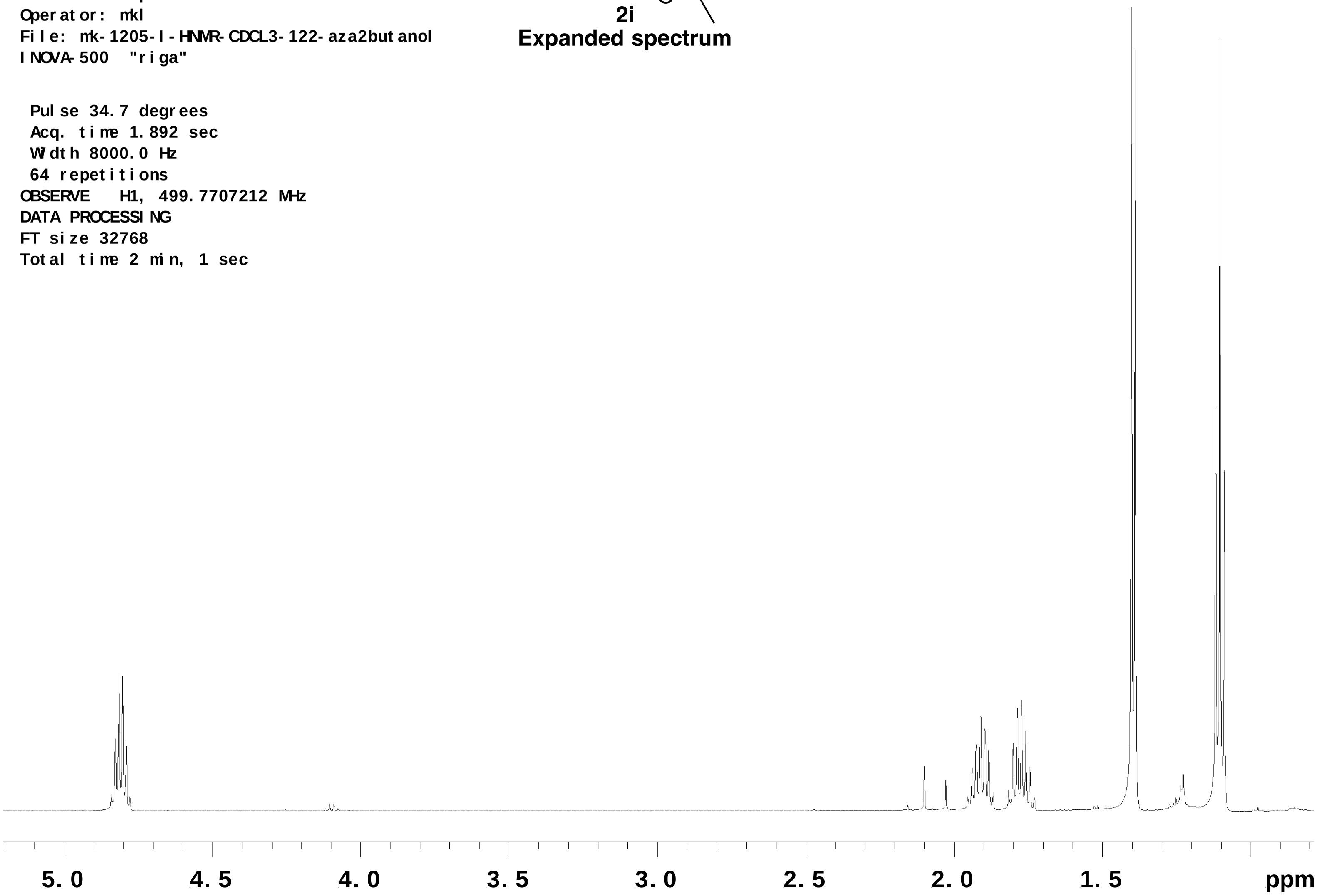

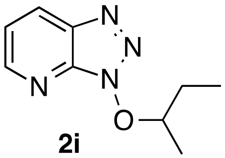

2i

mk-1205-I-C13-CDCL3-122-aza2butanol

Pulse Sequence: s2pul

Solvent: CDCl3
Ambient temperature
Operator: mkl
File: mk-1205-I-C13-CDCL3-122-aza2butanol
INOVA-500 "riga"

Pulse 45.0 degrees
Acq. time 1.300 sec
Width 25000.0 Hz
3455 repetitions
OBSERVE C13, 125.6674255 MHz
DECOUPLE H1, 499.7730084 MHz
Power 39 dB
continuously on
WALTZ-16 modulated
DATA PROCESSING
Line broadening 0.5 Hz
FT size 65536
Total time 1 hr, 49 min, 6 sec

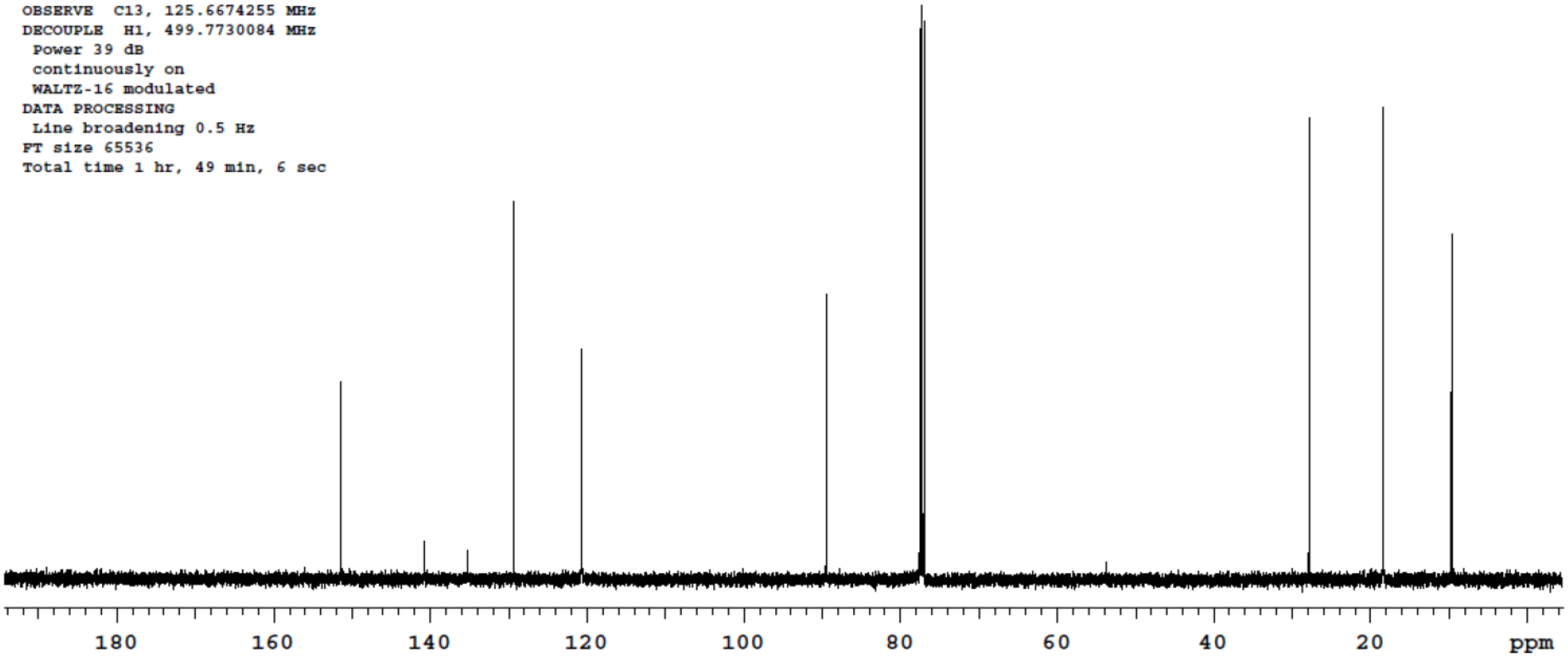

1205-Vijay-03-87-1H-CDCl3

Pulse Sequence: s2pul

Solvent: CDCl3
Temp. 25.0 C / 298.1 K
Operator: mkl
File:
INOVA-500 "riga"

Relax. delay 5.000 sec
Pulse 38.6 degrees
Acq. time 1.892 sec
Width 6002.4 Hz
32 repetitions
OBSERVE H1, 499.7707095 MHz
DATA PROCESSING
FT size 32768
Total time 3 min, 40 sec

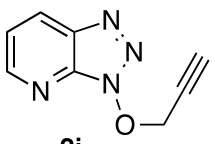

2j

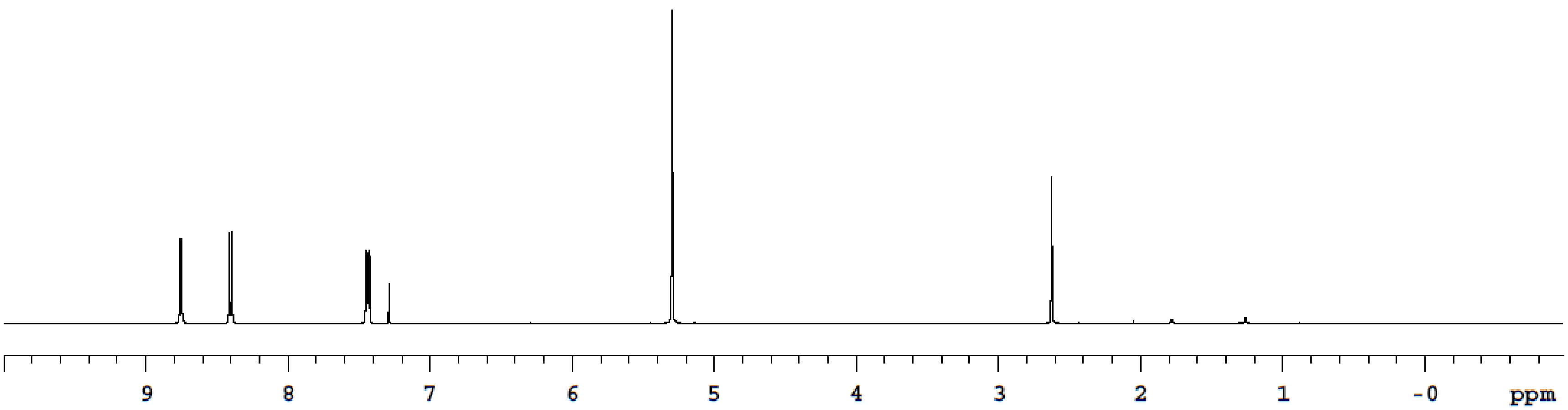

1205-Vijay-03-87-13C-CDCl3

Pulse Sequence: s2pul

Solvent: CDCl3
Ambient temperature
Operator: mkl
File:
INOVA-500 "riga"

Relax. delay 2.000 sec
Pulse 45.0 degrees
Acq. time 1.300 sec
Width 25000.0 Hz
588 repetitions
OBSERVE C13, 125.6674286 MHz
DECOUPLE H1, 499.7730084 MHz
Power 39 dB
continuously on
WALTZ-16 modulated
DATA PROCESSING
Line broadening 0.5 Hz
FT size 65536
Total time 55 min, 9 sec

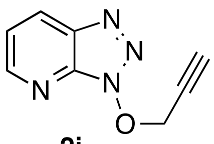

2j

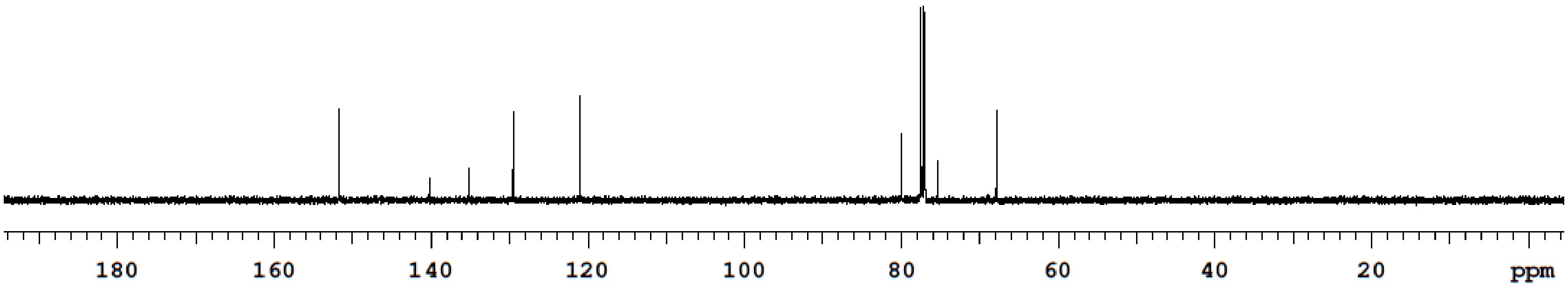

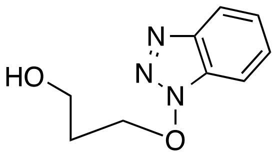

3a

RRC-1205-II-124-HNMR-CDCl3-monoether

Pulse Sequence: s2pul

Solvent: CDCl3
Temp. 25.0 C / 298.1 K
Operator: Raghu
File: RRC-1205-II-124-HNMR-CDCl3-monoether
INOVA-500 "riga"

Relax. delay 3.000 sec
Pulse 45.0 degrees
Acq. time 1.892 sec
Width 8000.0 Hz
64 repetitions
OBSERVE H1, 499.7707217 MHz
DATA PROCESSING
Resol. enhancement 0.5 Hz
Gauss apodization 0.500 sec
FT size 32768
Total time 5 min, 13 sec

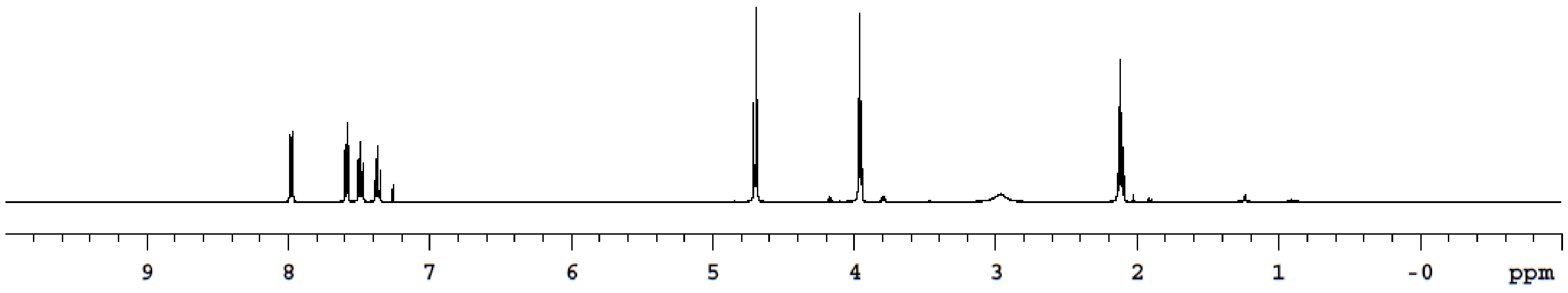

RRC-1205-II-124-HNMR-CDCl3-monoether

Pulse Sequence: s2pul

Solvent: CDCl3
Temp. 25.0 C / 298.1 K
Operator: Raghu
File: RRC-1205-II-124-HNMR-CDCl3-monoether
INOVA-500 "riga"

Relax. delay 3.000 sec
Pulse 45.0 degrees
Acq. time 1.892 sec
Width 8000.0 Hz
64 repetitions
OBSERVE H1, 499.7707217 MHz
DATA PROCESSING
Resol. enhancement 0.5 Hz
Gauss apodization 0.500 sec
FT size 32768
Total time 5 min, 13 sec

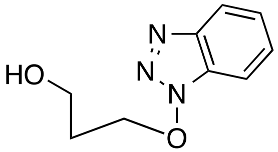

3a
Expanded spectrum

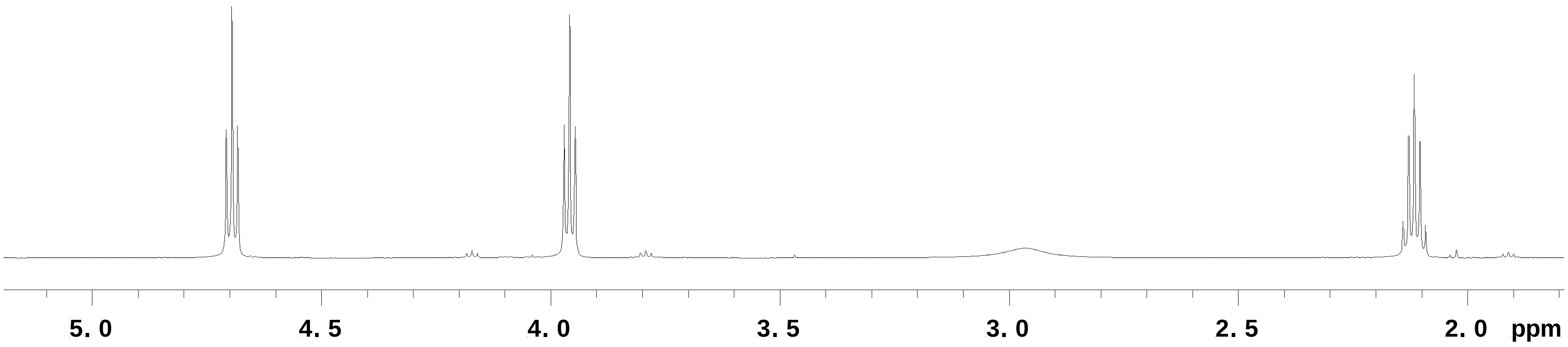

RRC-1205-II-124-CNMR-CDCl3-monoether

Pulse Sequence: s2pul

Solvent: CDCl3
Temp. 25.0 C / 298.1 K
Operator: Raghu
File: RRC-1205-II-124-CNMR-CDCl3-monoether
INOVA-500 "riga"

Relax. delay 3.000 sec
Pulse 45.0 degrees
Acq. time 1.300 sec
Width 29996.3 Hz
12210 repetitions
OBSERVE C13, 125.6674273 MHz
DECOUPLE H1, 499.7730084 MHz
Power 40 dB
continuously on
WALTZ-16 modulated
DATA PROCESSING
Line broadening 2.0 Hz
FT size 131072
Total time 29 hr, 55 min, 35 sec

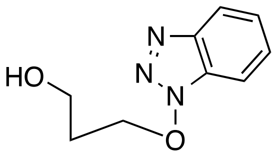

3a

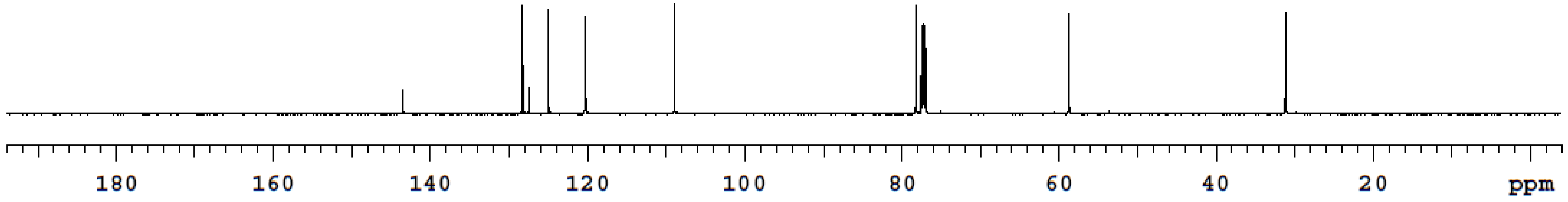

RRC-1205-II-124-HNMR-CDCl3-bisether

Pulse Sequence: s2pul

Solvent: CDCl3
Temp. 25.0 C / 298.1 K
Operator: Raghu
File: RRC-1205-II-124-HNMR-CDCl3-bisether
INOVA-500 "riga"

Relax. delay 3.000 sec
Pulse 45.0 degrees
Acq. time 1.892 sec
Width 8000.0 Hz
44 repetitions
OBSERVE H1, 499.7707217 MHz
DATA PROCESSING
Resol. enhancement 0.5 Hz
Gauss apodization 0.500 sec
FT size 32768
Total time 5 min, 13 sec

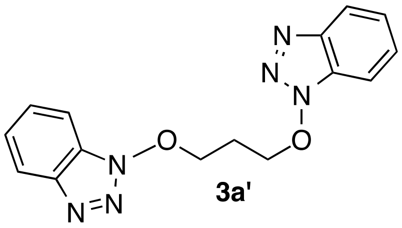

3a'

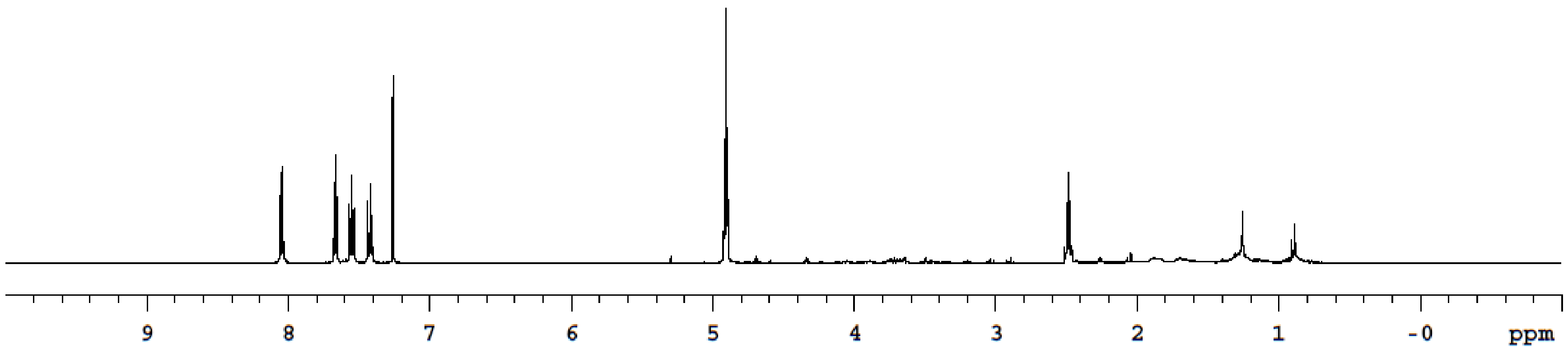

RRC-1205-II-124-CNMR-CDCl3-bisether

Pulse Sequence: s2pul

Solvent: CDCl3
Temp. 25.0 C / 298.1 K
Operator: Raghu
File: RRC-1205-II-124-CNMR-CDCl3-bisether
INOVA-500 "riga"

Relax. delay 3.000 sec
Pulse 45.0 degrees
Acq. time 1.300 sec
Width 29996.3 Hz
14490 repetitions
OBSERVE C13, 125.6674182 MHz
DECOUPLE H1, 499.7730084 MHz
Power 40 dB
continuously on
WALTZ-16 modulated
DATA PROCESSING
Line broadening 2.0 Hz
FT size 131072
Total time 29 hr, 55 min, 35 sec

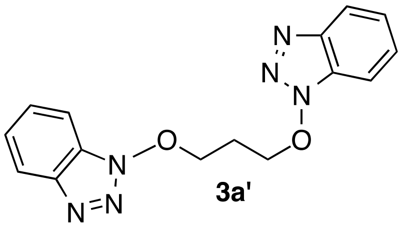

3a'

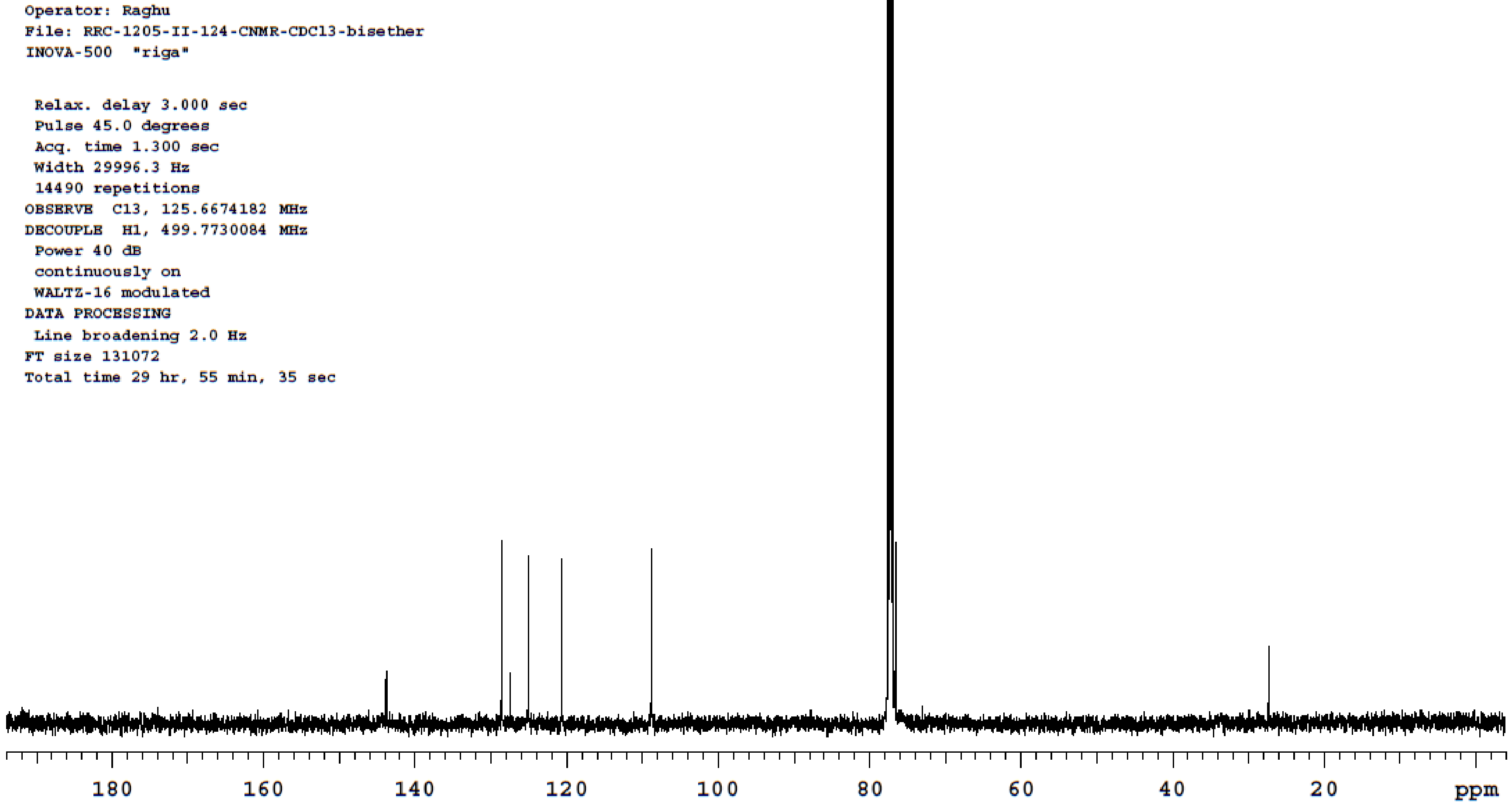

```
mk-1205-I-HNMR-CDCL3-propanediol-12122012

Pulse Sequence: s2pul
 Solvent: CDCl3
 Temp. 25.0 C / 298.1 K
Operator: mkl
File: mk-1205-I-HNMR-CDCL3-propanediol-12122012
INOVA-500  "riga"

 Pulse 34.7 degrees
 Acq. time 1.892 sec
 Width 8000.0 Hz
 64 repetitions
OBSERVE   H1, 499.7707095 MHz
DATA PROCESSING
FT size 32768
Total time 2 min, 1 sec
```

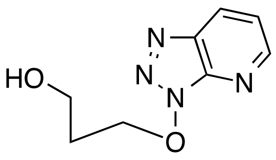

3b

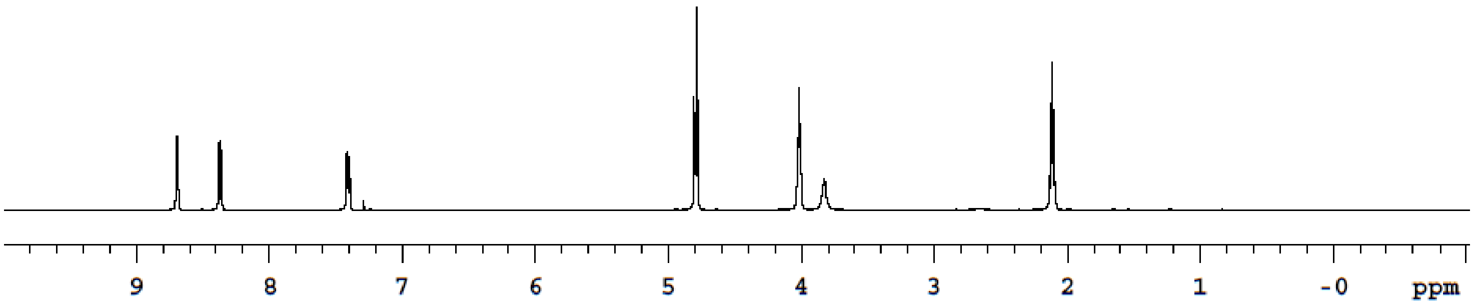

STANDARD CARBON PARAMETERS

Pulse Sequence: s2pul

Solvent: CDCl3
Ambient temperature
Operator: mkl
File: mk-1205-I-CDCL3-propanediolazanucleosidemimic-c13-8042012
INOVA-500 "riga"

Pulse 45.0 degrees
Acq. time 1.300 sec
Width 25000.0 Hz
2290 repetitions
OBSERVE C13, 125.6674149 MHz
DECOUPLE H1, 499.7730084 MHz
Power 39 dB
continuously on
WALTZ-16 modulated
DATA PROCESSING
Line broadening 0.5 Hz
FT size 65536
Total time 7 hr, 16 min, 27 sec

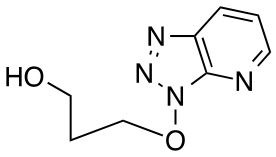

3b

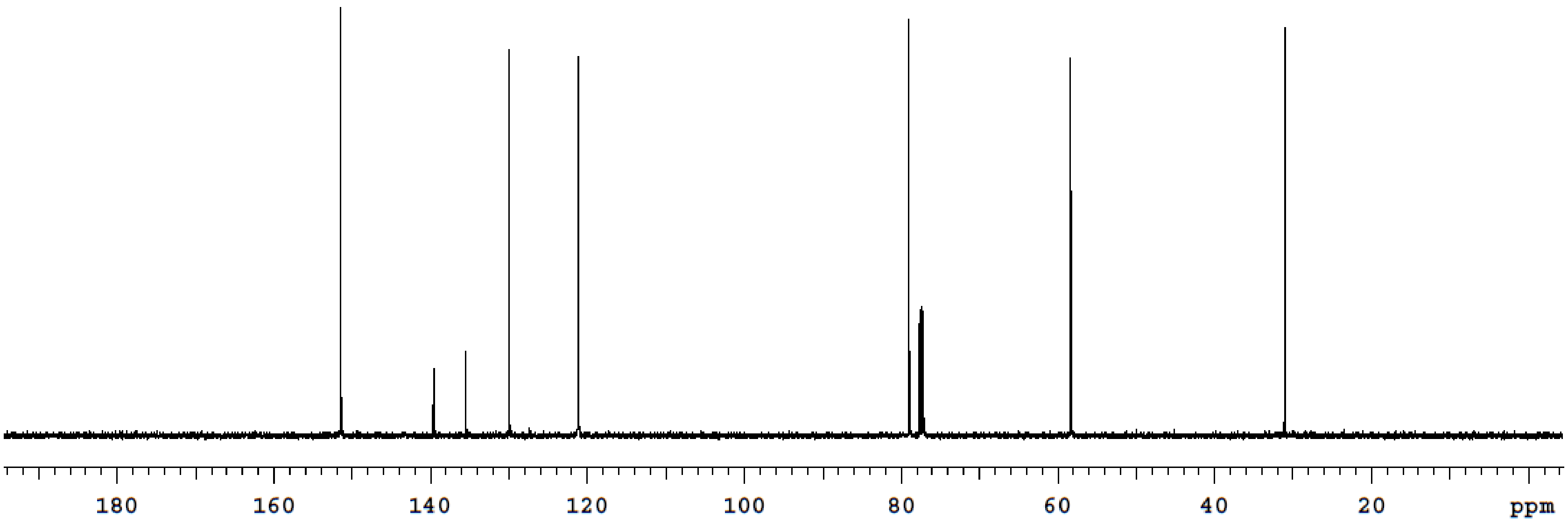

1205-Vijay-03-89-1H-CDCl3

Pulse Sequence: s2pul

Solvent: CDCl3
Temp. 25.0 C / 298.1 K
Operator: mkl
File:
INOVA-500 "riga"

Relax. delay 5.000 sec
Pulse 38.6 degrees
Acq. time 1.892 sec
Width 6002.4 Hz
32 repetitions
OBSERVE H1, 499.7707095 MHz
DATA PROCESSING
FT size 32768
Total time 3 min, 40 sec

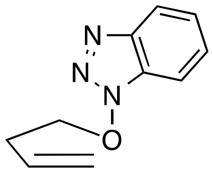

4a

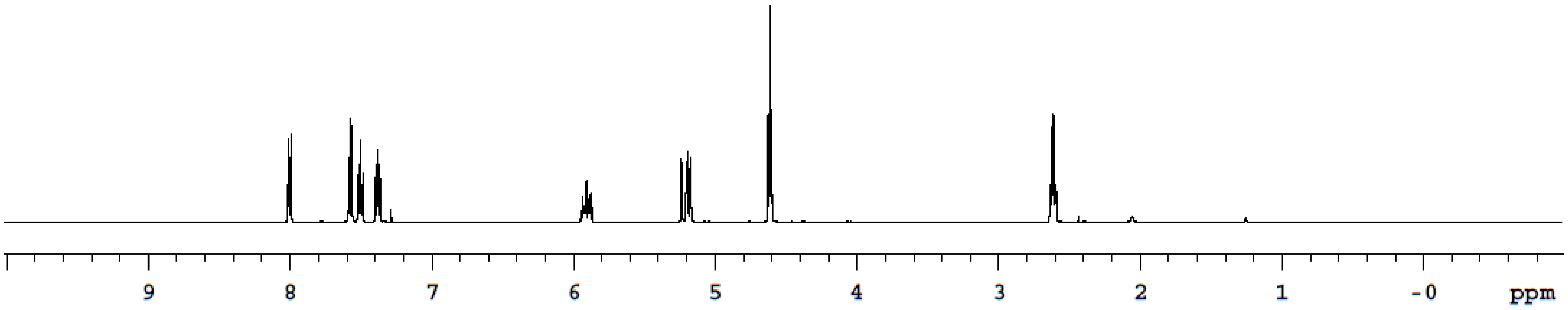

```
1205-Vijay-03-89-13C-CDCl3

Pulse Sequence: s2pul
 Solvent: CDCl3
 Ambient temperature
Operator: mkl
File:
INOVA-500  "riga"

 Relax. delay 2.000 sec
 Pulse 45.0 degrees
 Acq. time 1.300 sec
 Width 29996.3 Hz
 104 repetitions
OBSERVE  C13, 125.6674149 MHz
DECOUPLE  H1, 499.7730084 MHz
 Power 39 dB
 continuously on
 WALTZ-16 modulated
DATA PROCESSING
 Line broadening 0.5 Hz
FT size 131072
Total time 55 min, 9 sec
```

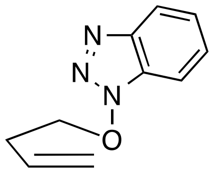

4a

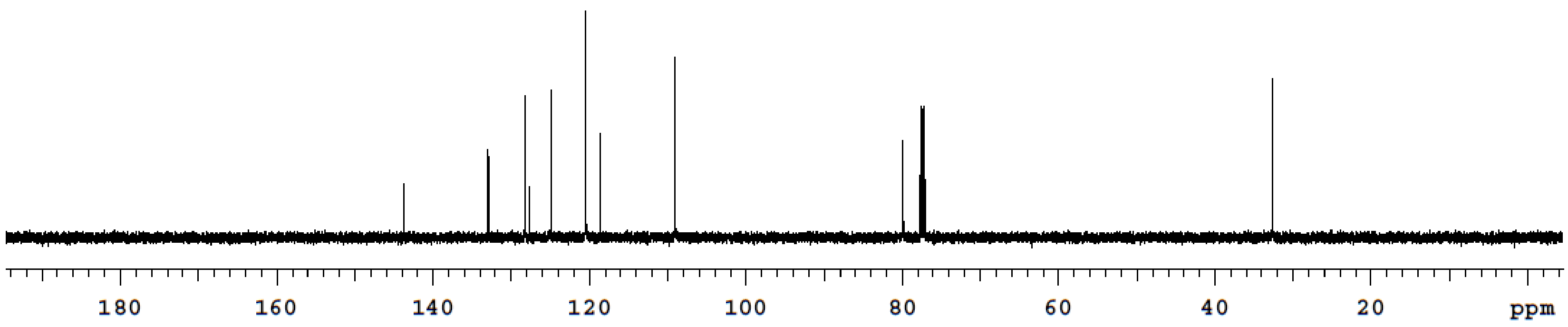

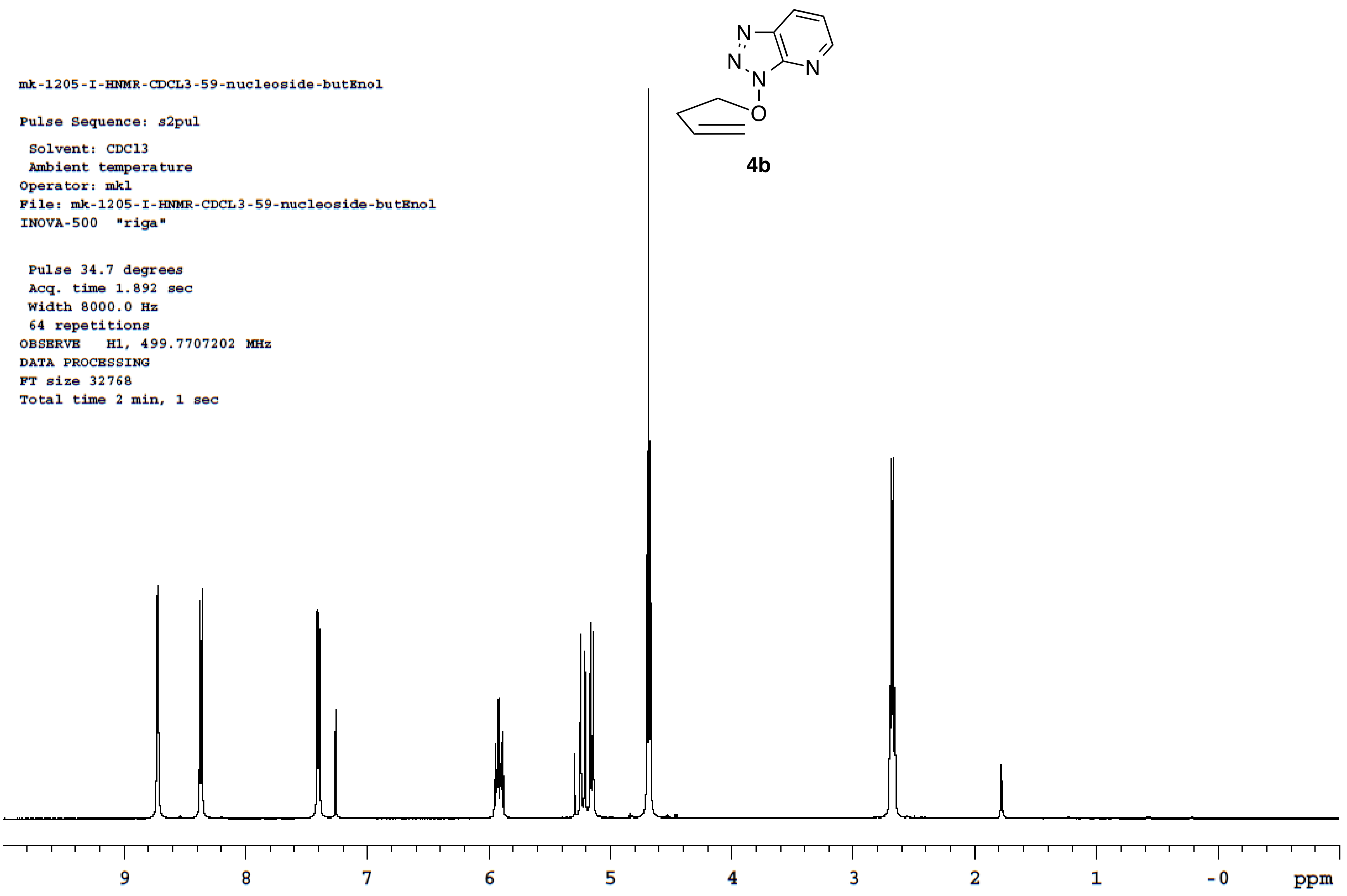
4b
mk-1205-I-HNMR-CDCL3-59-nucleoside-butEnol
Pulse Sequence: s2pul
Solvent: CDCl3
Ambient temperature
Operator: mkl
File: mk-1205-I-HNMR-CDCL3-59-nucleoside-butEnol
INOVA-500 "riga"
Pulse 34.7 degrees
Acq. time 1.892 sec
Width 8000.0 Hz
64 repetitions
OBSERVE H1, 499.7707202 MHz
DATA PROCESSING
FT size 32768
Total time 2 min, 1 sec
9
8
7
6
5
4
3
2
1
-0
ppm

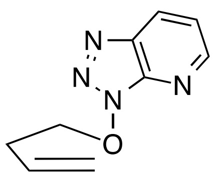

4b
Expanded spectrum

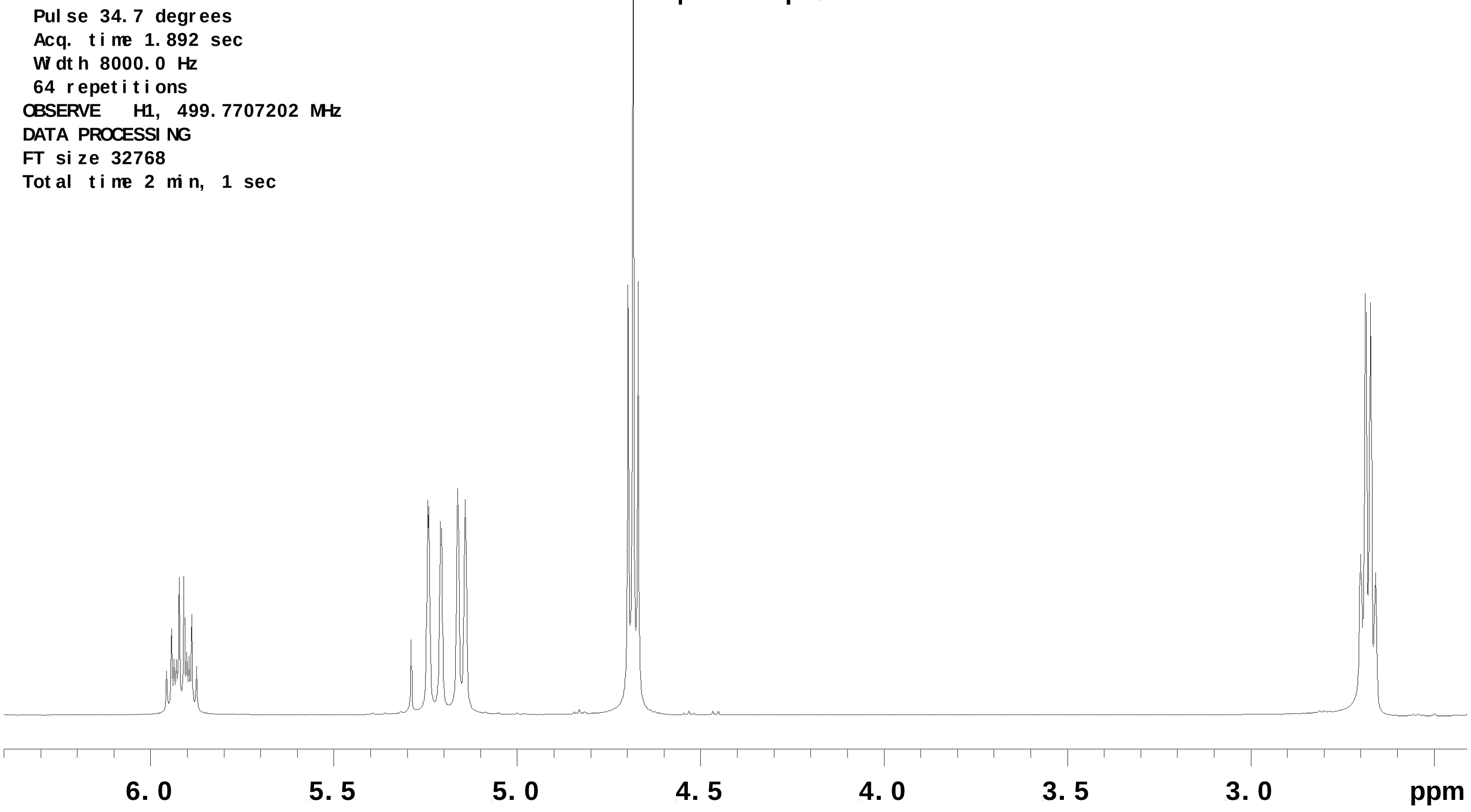

mk-1205-I-C13-CDCL3-59-nucleoside-butEnol

Pulse Sequence: s2pul

Solvent: CDCl3
Ambient temperature
Operator: mkl
File: mk-1205-I-C13-CDCL3-60-nucleoside-butEnol
INOVA-500 "riga"

Pulse 45.0 degrees
Acq. time 1.300 sec
Width 25000.0 Hz
3110 repetitions
OBSERVE C13, 125.6674248 MHz
DECOUPLE H1, 499.7730084 MHz
Power 39 dB
continuously on
WALTZ-16 modulated
DATA PROCESSING
Line broadening 0.5 Hz
FT size 65536
Total time 7 hr, 16 min, 27 sec

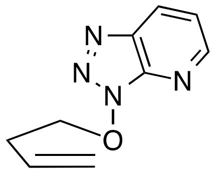

4b

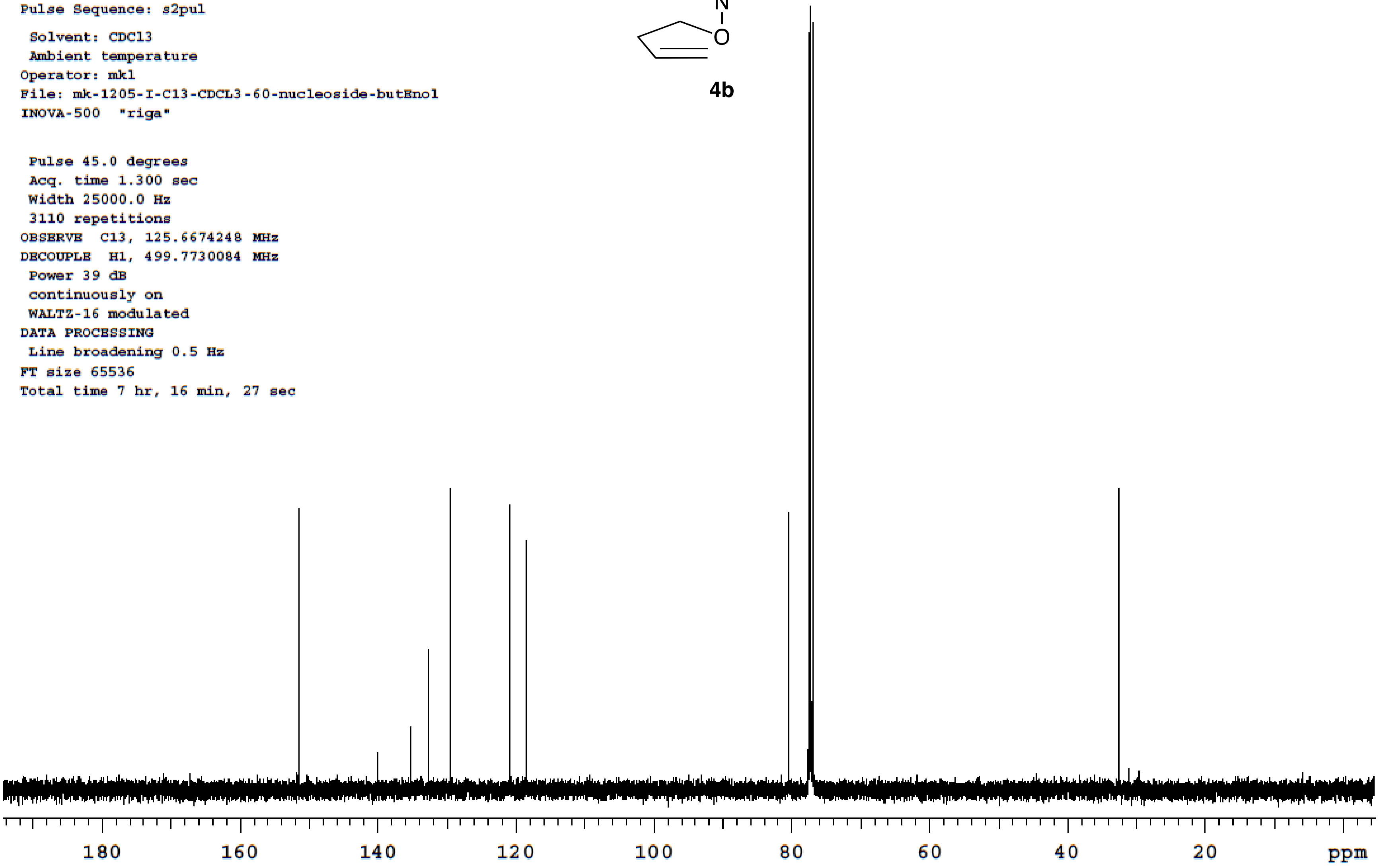

```
1205-Vijay-01-42-pure-1H-CDCl3

Pulse Sequence: s2pul
 Solvent: CDCl3
 Temp. 25.0 C / 298.1 K
Operator: mkl
File:
INOVA-500  "riga"

 Pulse 38.6 degrees
 Acq. time 1.892 sec
 Width 8000.0 Hz
 42 repetitions
OBSERVE   H1, 499.7707095 MHz
DATA PROCESSING
FT size 32768
Total time 6 min, 20 sec
```

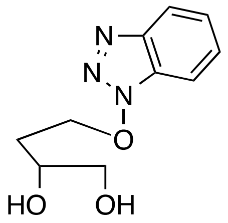

(±)-5a

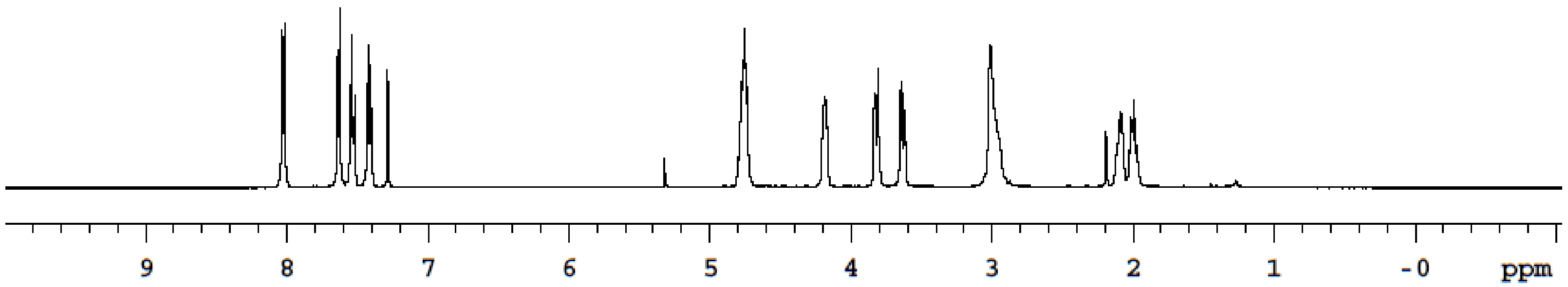

```
1205-vijay-01-42-13C-cdcl3

Pulse Sequence: s2pul
 Solvent: CDCl3
 Temp. 25.0 C / 298.1 K
Operator: mkl
File:
INOVA-500  "riga"

 Pulse 45.0 degrees
 Acq. time 1.300 sec
 Width 25000.0 Hz
 2831 repetitions
OBSERVE  C13, 125.6674210 MHz
DECOUPLE  H1, 499.7730084 MHz
 Power 39 dB
 continuously on
 WALTZ-16 modulated
DATA PROCESSING
 Line broadening 0.5 Hz
FT size 65536
Total time 3 hr, 38 min, 13 sec
```

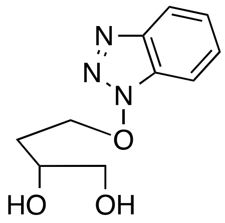

(±)-5a

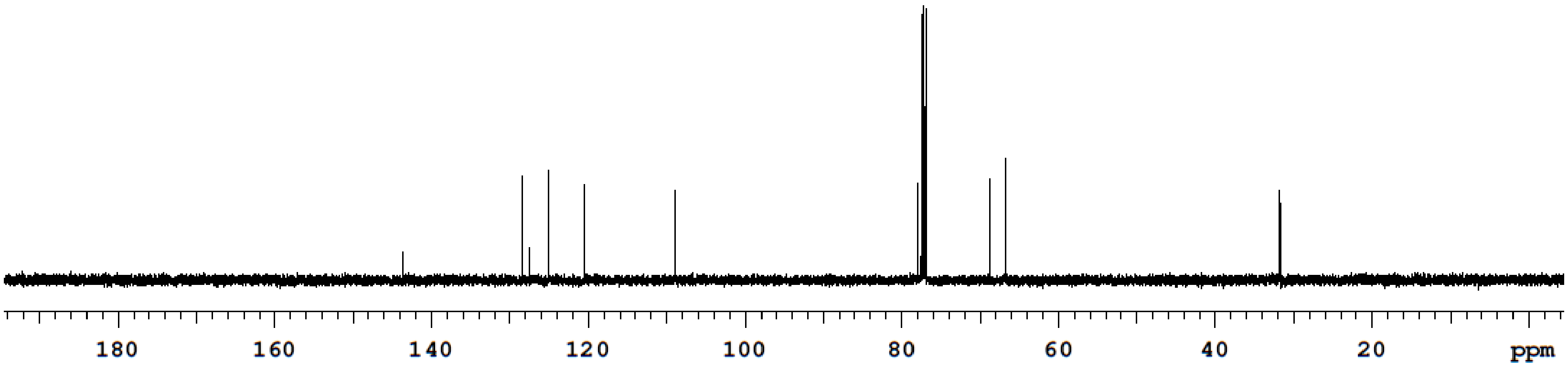

mk-1205-I-HNMR-CDCL3-62-dihydroxybutenol

Pulse Sequence: s2pul

Solvent: CDCl3
Ambient temperature
Operator: mkl
File: mk-1205-I-HNMR-CDCL3-62-dihydroxybutenol
INOVA-500 "riga"

Pulse 34.7 degrees
Acq. time 1.892 sec
Width 8000.0 Hz
64 repetitions
OBSERVE H1, 499.7707217 MHz
DATA PROCESSING
FT size 32768
Total time 2 min, 1 sec

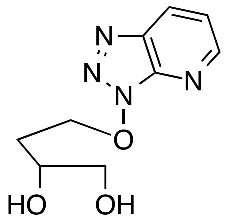

(±)-5b

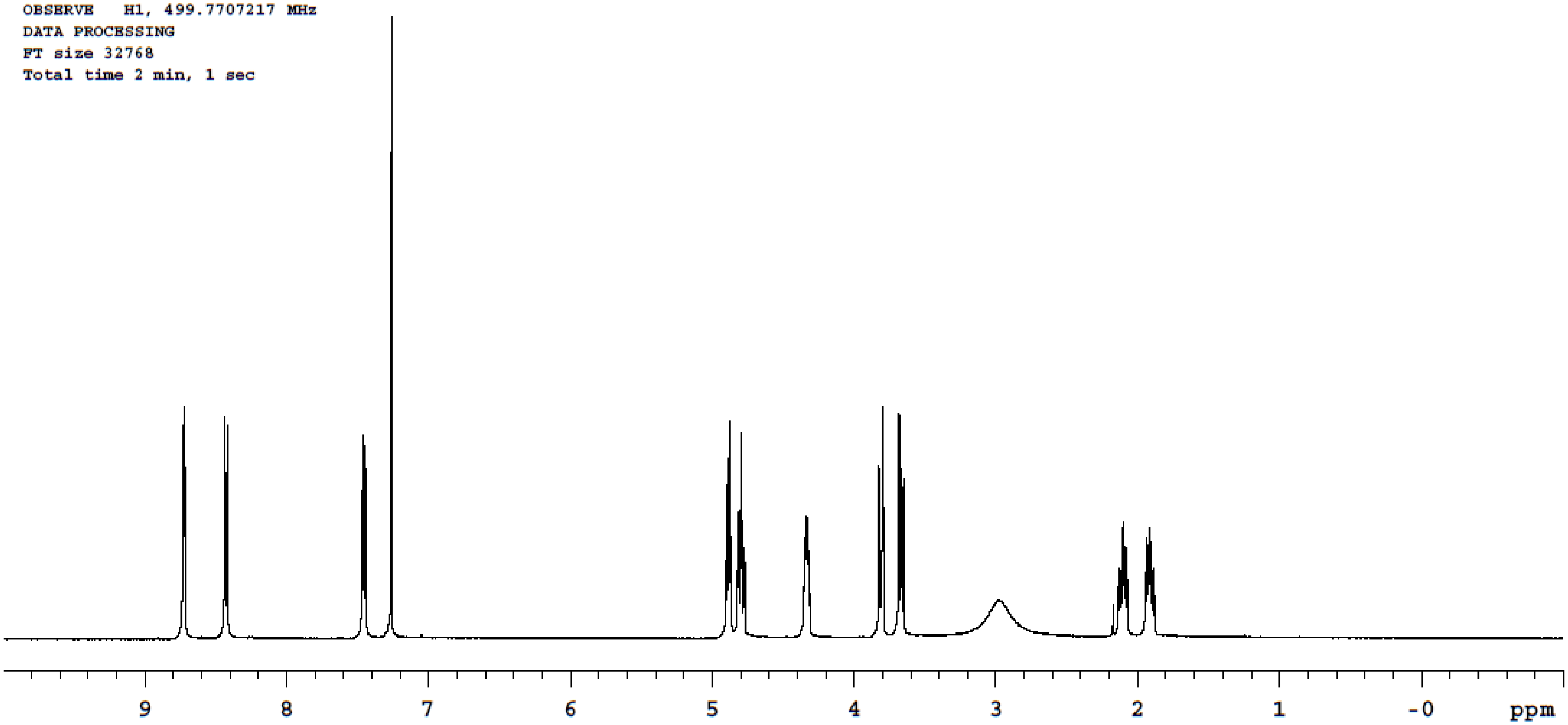

mk-1205-I-HNMR-CDCL3-62-dihydroxybutenol

Pulse Sequence: s2pul

Solvent: CDCl3
Ambient temperature
Operator: mkl
File: mk-1205-I-HNMR-CDCL3-62-dihydroxybutenol
INOVA-500 "riga"

Pulse 34.7 degrees
Acq. time 1.892 sec
Width 8000.0 Hz
64 repetitions
OBSERVE H1, 499.7707217 MHz
DATA PROCESSING
FT size 32768
Total time 2 min, 1 sec

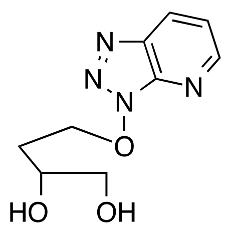

(±)-5b
Expanded spectrum

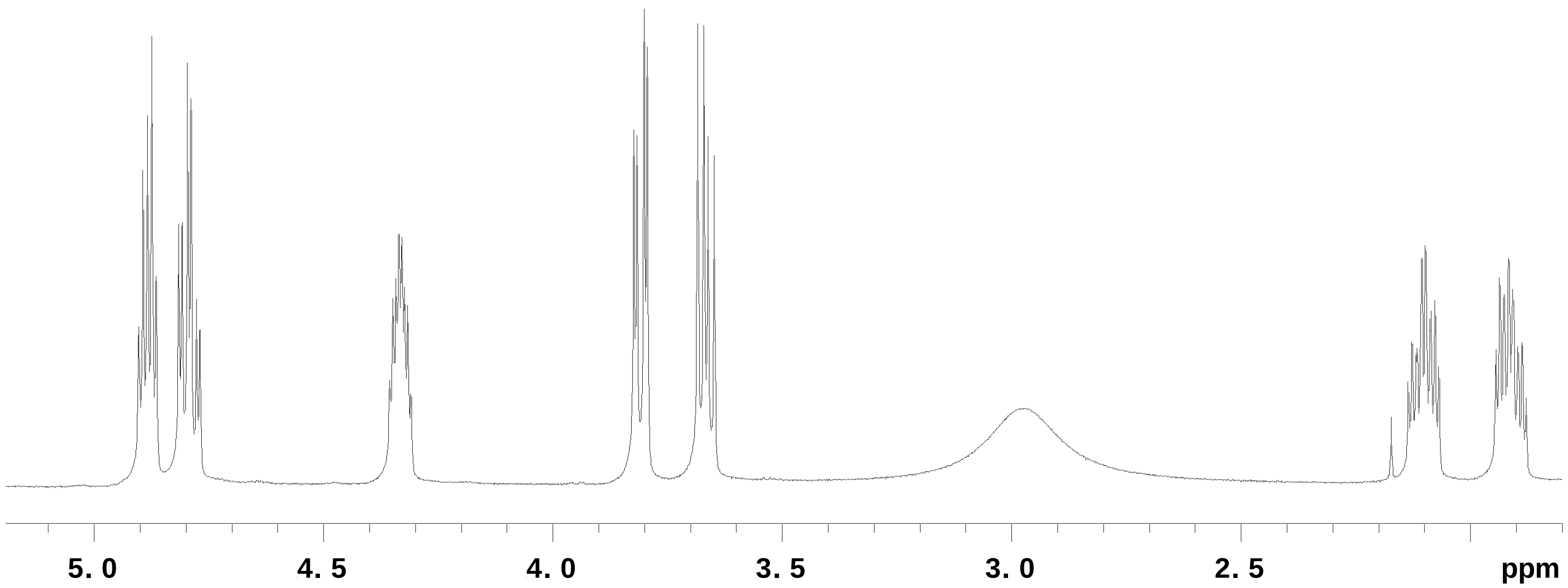

dihydroxybutenol-c13

Pulse Sequence: s2pul

Solvent: CDCl3
Ambient temperature
Operator: mkl
File: mk-1205-I-C13-CDCL3-62-dihydroxybutenol
INOVA-500 "riga"

Pulse 45.0 degrees
Acq. time 1.300 sec
Width 25000.0 Hz
2240 repetitions
OBSERVE C13, 125.6674263 MHz
DECOUPLE H1, 499.7730084 MHz
Power 39 dB
continuously on
WALTZ-16 modulated
DATA PROCESSING
Line broadening 0.5 Hz
FT size 65536
Total time 7 hr, 16 min, 27 sec

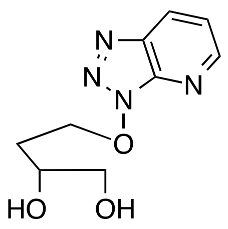

(±)-5b

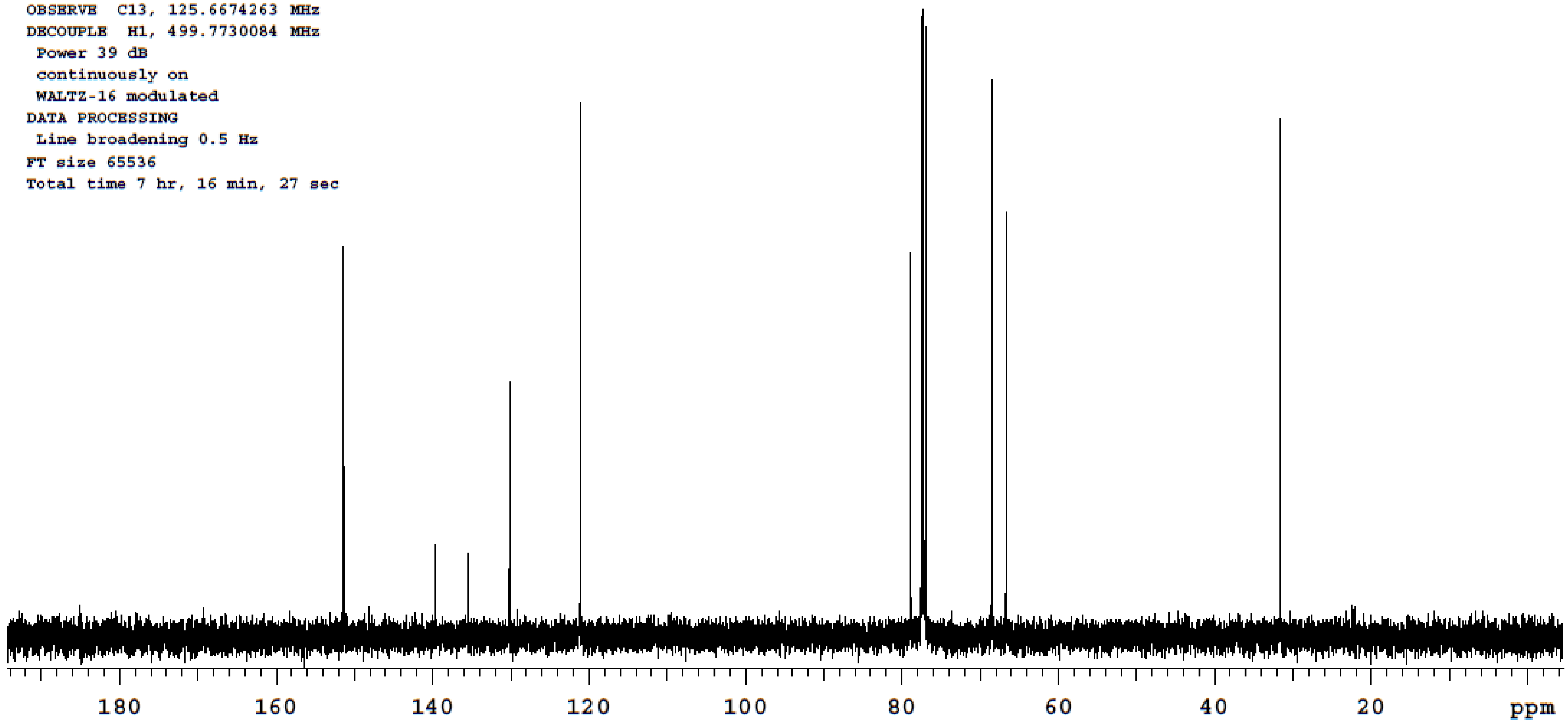

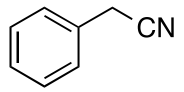

6

RRC-1205-III-273-HNMR-CDCl3

Pulse Sequence: *s2pul*

Solvent: CDCl3
Temp. 25.0 C / 298.1 K
Operator: Raghu
File: RRC-1205-III-273-HNMR-CDCl3
INOVA-500 "riga"

Relax. delay 5.000 *sec*
Pulse 45.0 degrees
Acq. time 1.892 sec
Width 8000.0 Hz
64 repetitions
OBSERVE H1, 499.7707212 MHz
DATA PROCESSING
Resol. enhancement 0.5 Hz
Gauss apodization 0.500 sec
FT size 32768
Total time 7 min, 21 sec

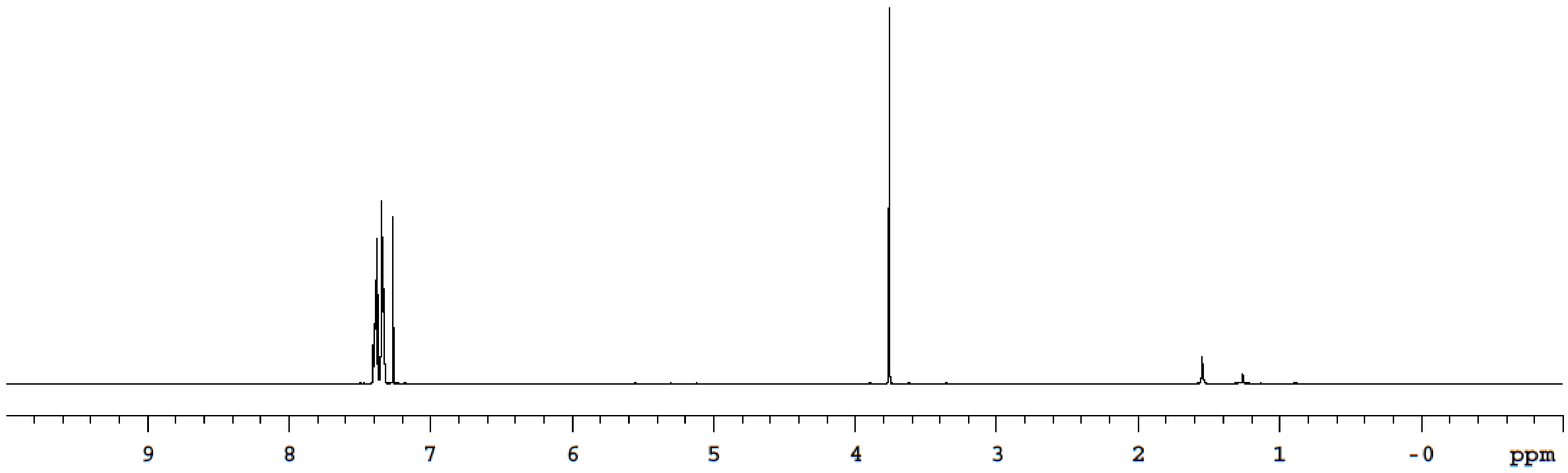

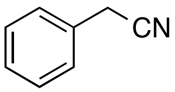
CN

6

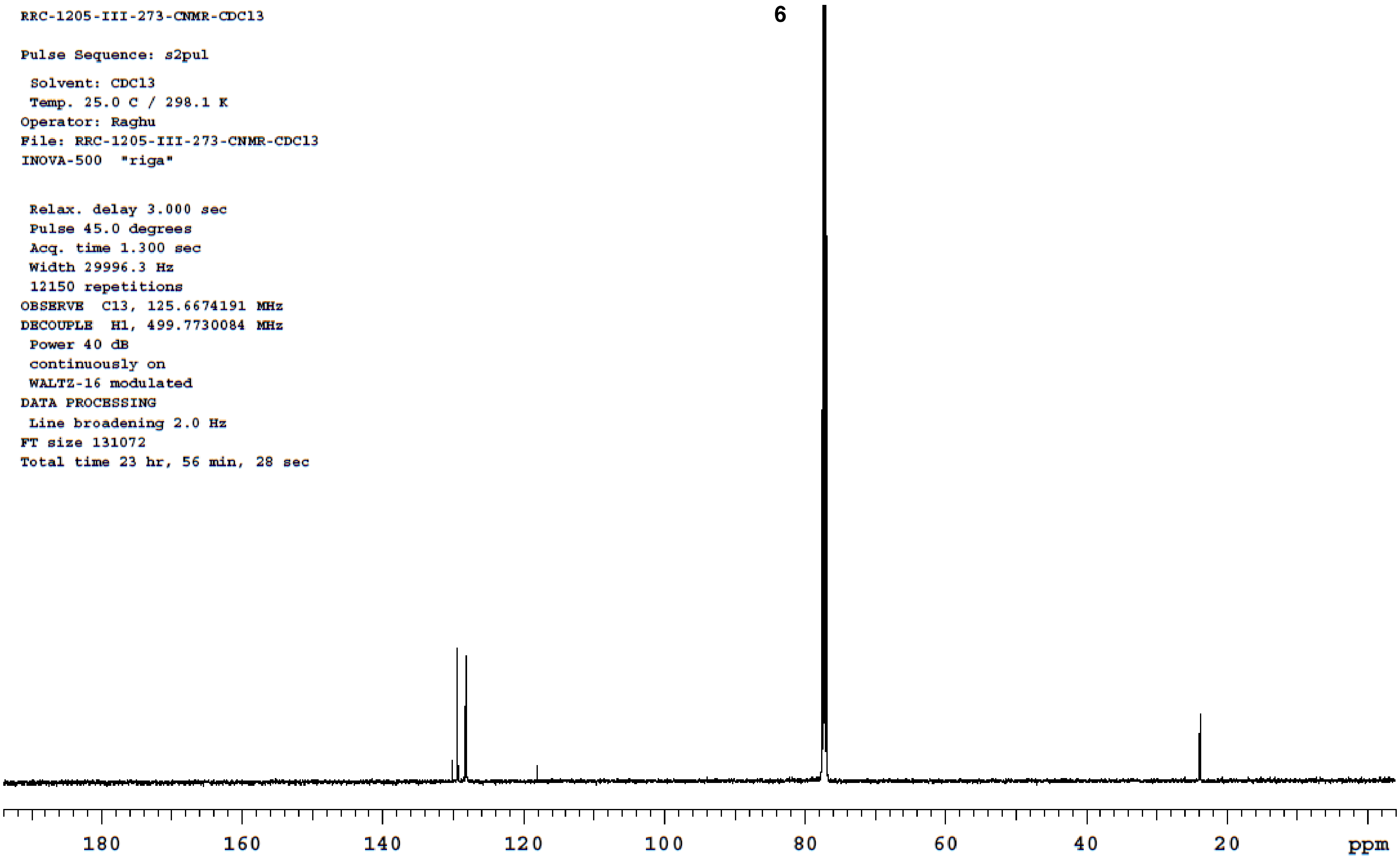
RRC-1205-III-273-CNMR-CDCl3
Pulse Sequence: s2pul
Solvent: CDCl3
Temp. 25.0 C / 298.1 K
Operator: Raghu
File: RRC-1205-III-273-CNMR-CDCl3
INOVA-500 "riga"
Relax. delay 3.000 sec
Pulse 45.0 degrees
Acq. time 1.300 sec
Width 29996.3 Hz
12150 repetitions
OBSERVE C13, 125.6674191 MHz
DECOUPLE H1, 499.7730084 MHz
Power 40 dB
continuously on
WALTZ-16 modulated
DATA PROCESSING
Line broadening 2.0 Hz
FT size 131072
Total time 23 hr, 56 min, 28 sec
180
160
140
120
100
80
60
40
20
ppm

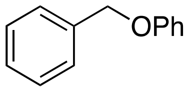

7

RRC-1205-III-272-HNMR-CDCl3-topspot

Pulse Sequence: s2pul

Solvent: CDCl3
Temp. 25.0 C / 298.1 K
Operator: Raghu
File: RRC-1205-III-272-HNMR-CDCl3-topspot
INOVA-500 "riga"

Relax. delay 5.000 sec
Pulse 45.0 degrees
Acq. time 1.892 sec
Width 8000.0 Hz
32 repetitions
OBSERVE H1, 499.7707212 MHz
DATA PROCESSING
Resol. enhancement 0.5 Hz
Gauss apodization 0.500 sec
FT size 32768
Total time 7 min, 21 sec

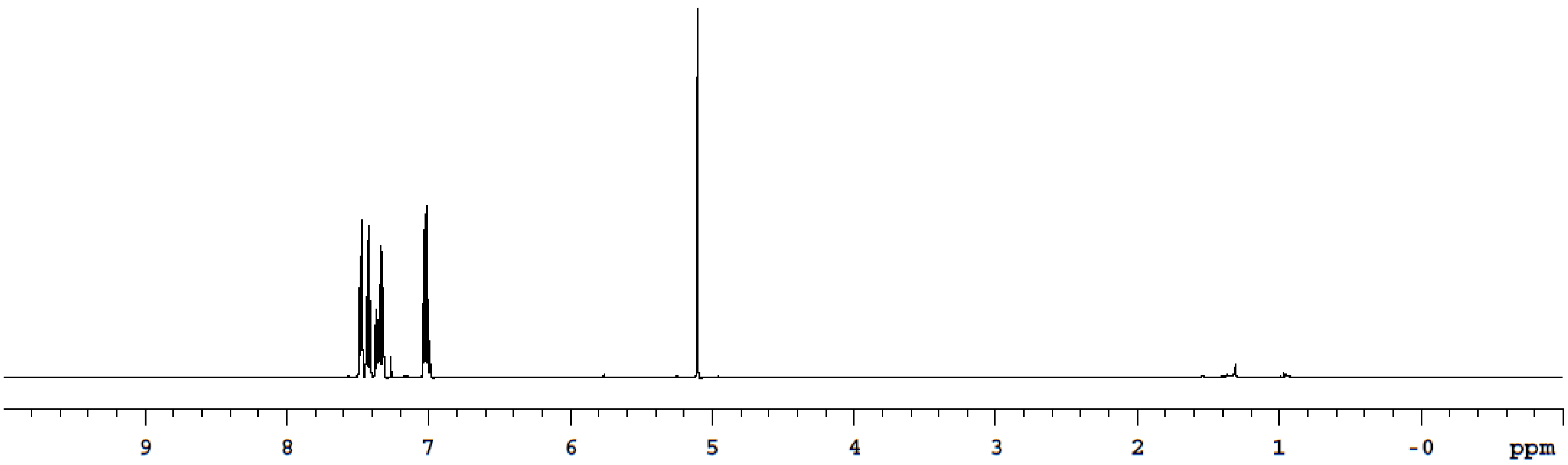

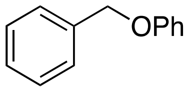

7

RRC-1205-III-272-CNMR-CDCl3

Pulse Sequence: s2pul

Solvent: CDCl3
Temp. 25.0 C / 298.1 K
Operator: Raghu
File: RRC-1205-III-272-CNMR-CDCl3
INOVA-500 "riga"

Relax. delay 3.000 sec
Pulse 45.0 degrees
Acq. time 1.300 sec
Width 30499.4 Hz
12570 repetitions
OBSERVE C13, 125.6674212 MHz
DECOUPLE H1, 499.7730084 MHz
Power 40 dB
continuously on
WALTZ-16 modulated
DATA PROCESSING
Line broadening 2.0 Hz
FT size 131072
Total time 23 hr, 56 min, 27 sec

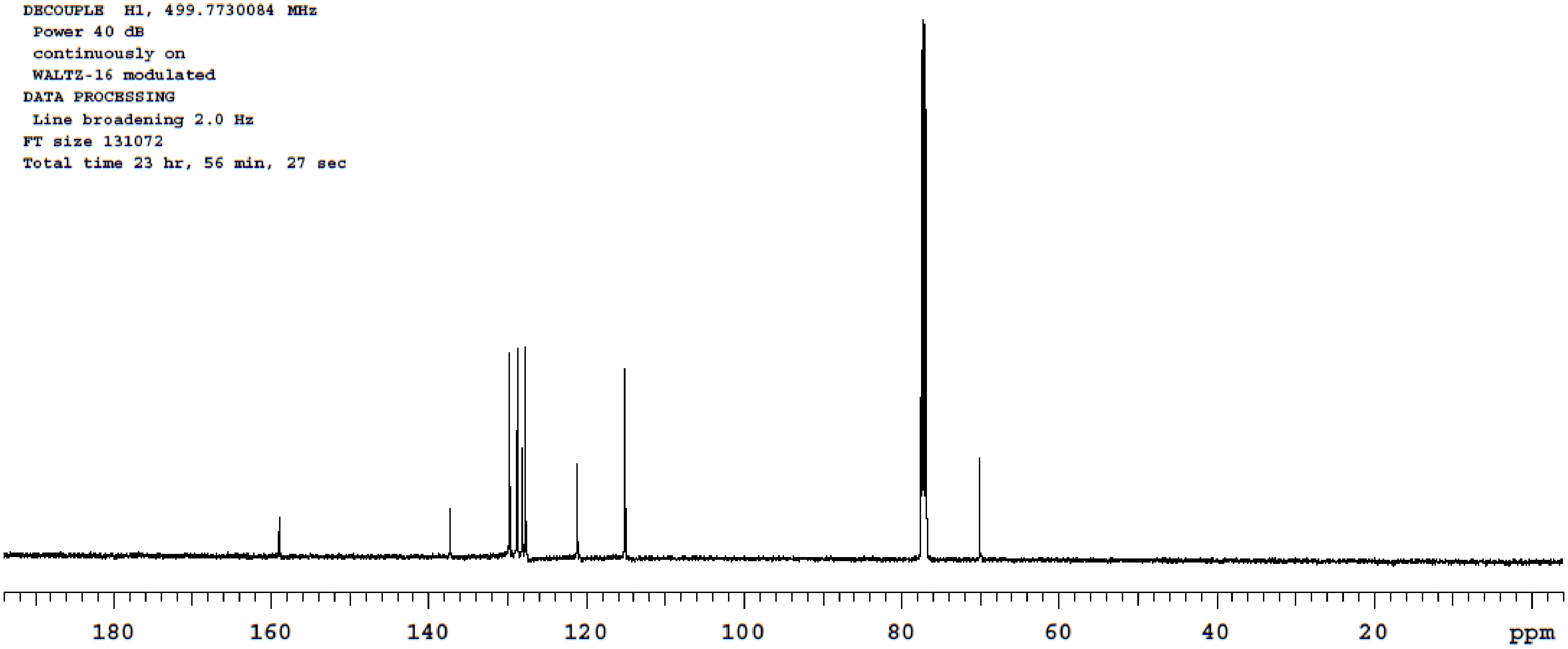

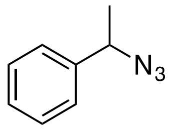

8

RRC-1205-III-252-HNMR-CDCl3

Pulse Sequence: s2pul

Solvent: CDCl3
Temp. 25.0 C / 298.1 K
Operator: Raghu
File: RRC-1205-III-252-HNMR-CDCl3
INOVA-500 "riga"

Relax. delay 5.000 sec
Pulse 45.0 degrees
Acq. time 1.892 sec
Width 8000.0 Hz
64 repetitions
OBSERVE H1, 499.7707212 MHz
DATA PROCESSING
Resol. enhancement 0.5 Hz
Gauss apodization 0.500 sec
FT size 32768
Total time 7 min, 21 sec

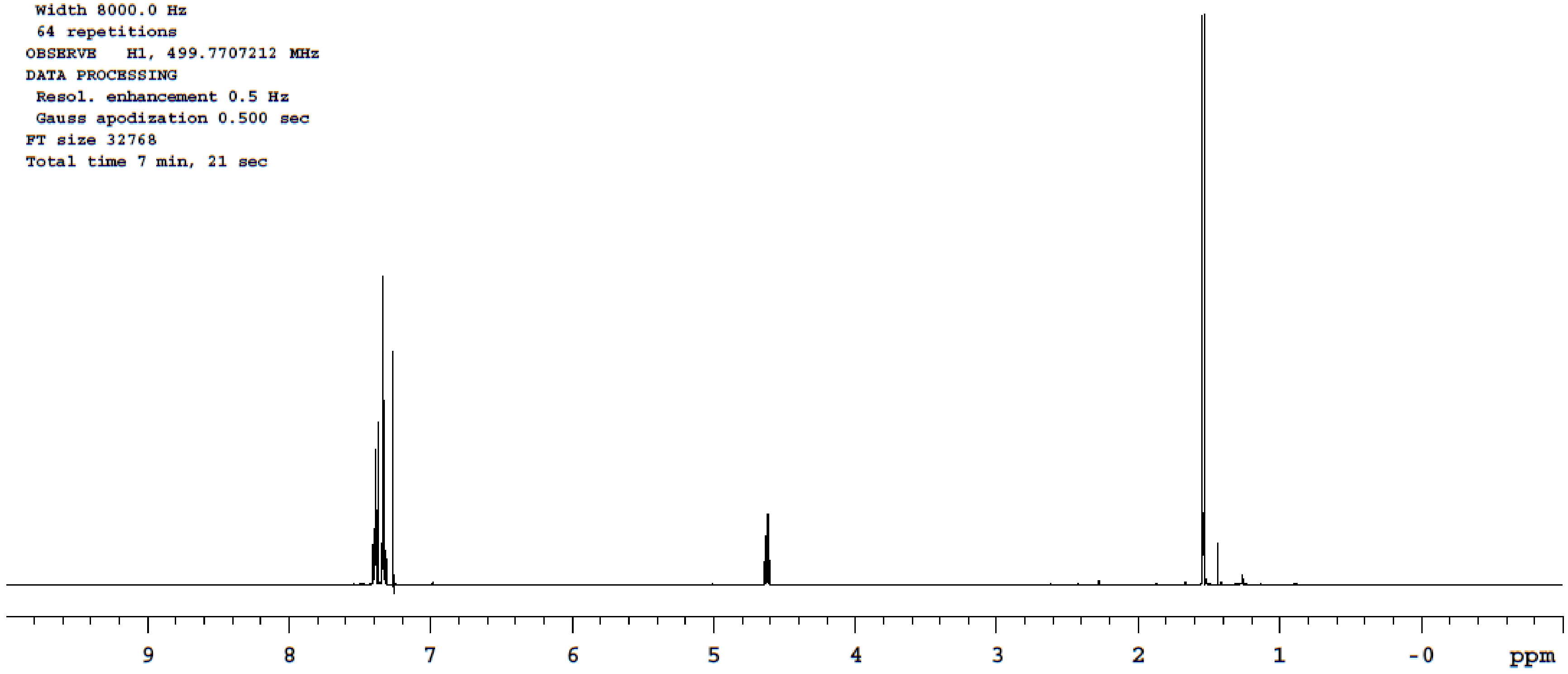

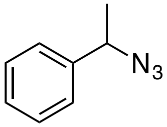

8

RRC-1205-III-252-CNMR-CDCl3

Pulse Sequence: s2pul

Solvent: CDCl3
Temp. 25.0 C / 298.1 K
Operator: Raghu
File: RRC-1205-III-252-CNMR-CDCl3
INOVA-500 "riga"

Relax. delay 3.000 sec
Pulse 45.0 degrees
Acq. time 1.300 sec
Width 29996.3 Hz
12220 repetitions
OBSERVE C13, 125.6674186 MHz
DECOUPLE H1, 499.7730084 MHz
Power 40 dB
continuously on
WALTZ-16 modulated
DATA PROCESSING
Line broadening 2.0 Hz
FT size 131072
Total time 29 hr, 55 min, 35 sec

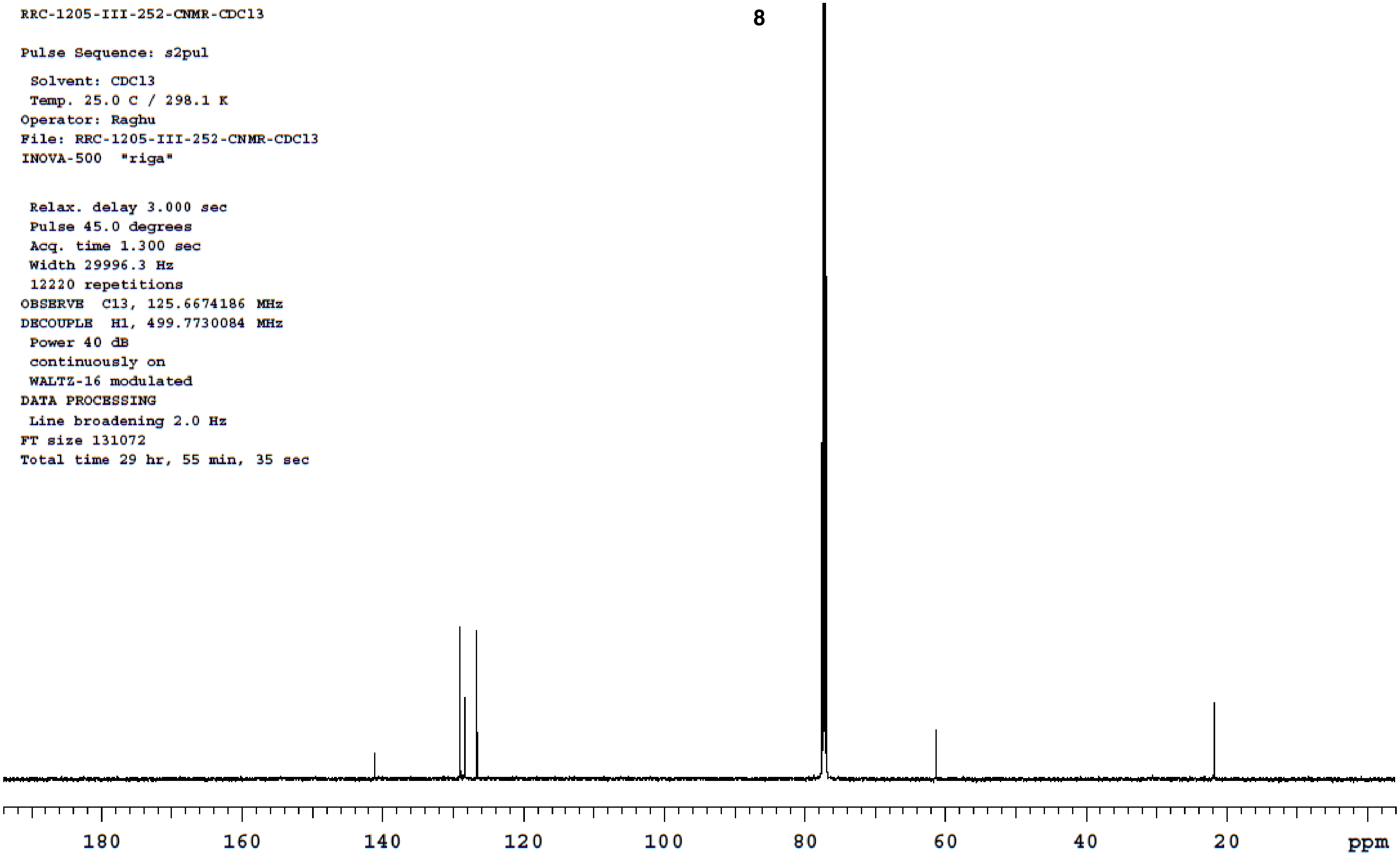

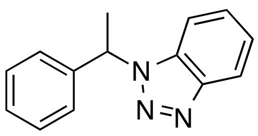

9a

RRC-1205-III-thirdspot-HNMR-CDCl3-Bt

Pulse Sequence: s2pul

Solvent: CDCl3
Temp. 25.0 C / 298.1 K
Operator: Raghu
File: RRC-1205-III-256-HNMR-CDCl3-thirdspot
INOVA-500 "riga"

Relax. delay 5.000 sec
Pulse 45.0 degrees
Acq. time 1.892 sec
Width 8000.0 Hz
32 repetitions
OBSERVE H1, 499.7707212 MHz
DATA PROCESSING
Resol. enhancement 0.5 Hz
Gauss apodization 0.500 sec
FT size 32768
Total time 3 min, 40 sec

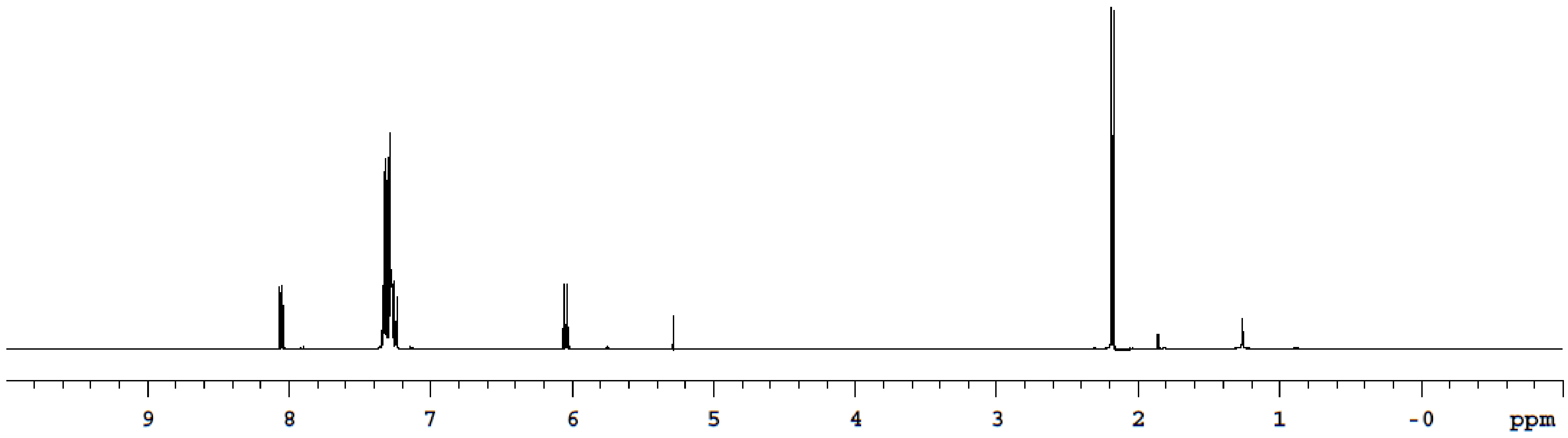

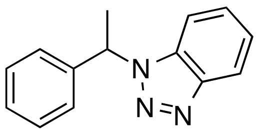

9a

RRC-1205-III-256-CNMR-CDCl3-thirdspot

Pulse Sequence: s2pul

Solvent: CDCl3
Temp. 25.0 C / 298.1 K
Operator: Raghu
File: RRC-1205-III-256-CNMR-CDCl3-thirdspot
INOVA-500 "riga"

Relax. delay 3.000 sec
Pulse 45.0 degrees
Acq. time 1.300 sec
Width 29996.3 Hz
13760 repetitions
OBSERVE C13, 125.6674283 MHz
DECOUPLE H1, 499.7730084 MHz
Power 40 dB
continuously on
WALTZ-16 modulated
DATA PROCESSING
Line broadening 2.0 Hz
FT size 131072
Total time 29 hr, 55 min, 35 sec

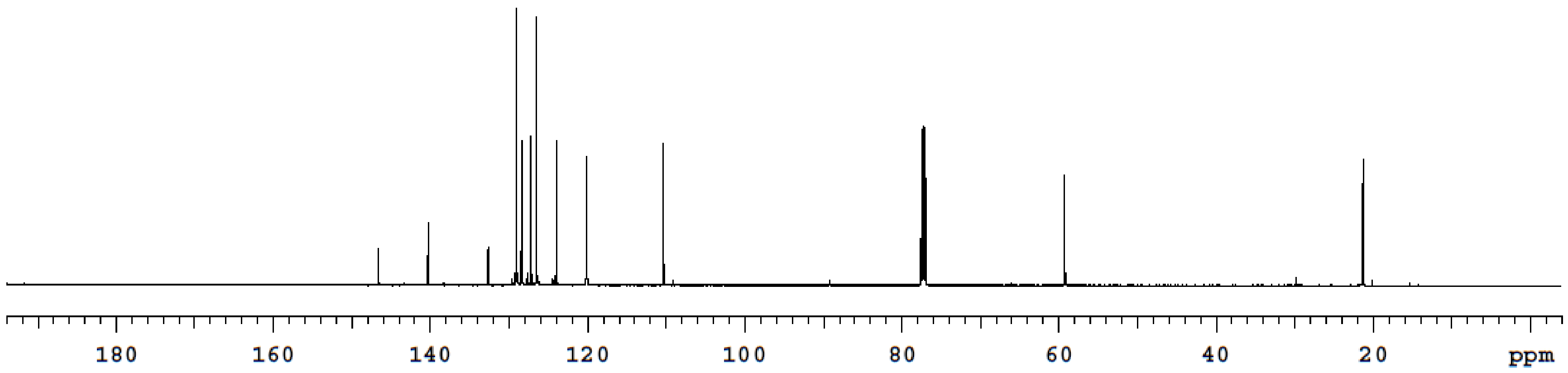

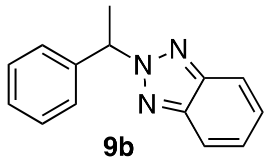

9b

RRC-1205-III-firstspot-HNMR-CDCl3-Bt

Pulse Sequence: s2pul

Solvent: CDCl3
Temp. 25.0 C / 298.1 K
Operator: Raghu
File: RRC-1205-III-256-HNMR-CDCl3-firstspot
INOVA-500 "riga"

Relax. delay 5.000 sec
Pulse 45.0 degrees
Acq. time 1.892 sec
Width 8000.0 Hz
32 repetitions
OBSERVE H1, 499.7707212 MHz
DATA PROCESSING
Resol. enhancement 0.5 Hz
Gauss apodization 0.500 sec
FT size 32768
Total time 3 min, 40 sec

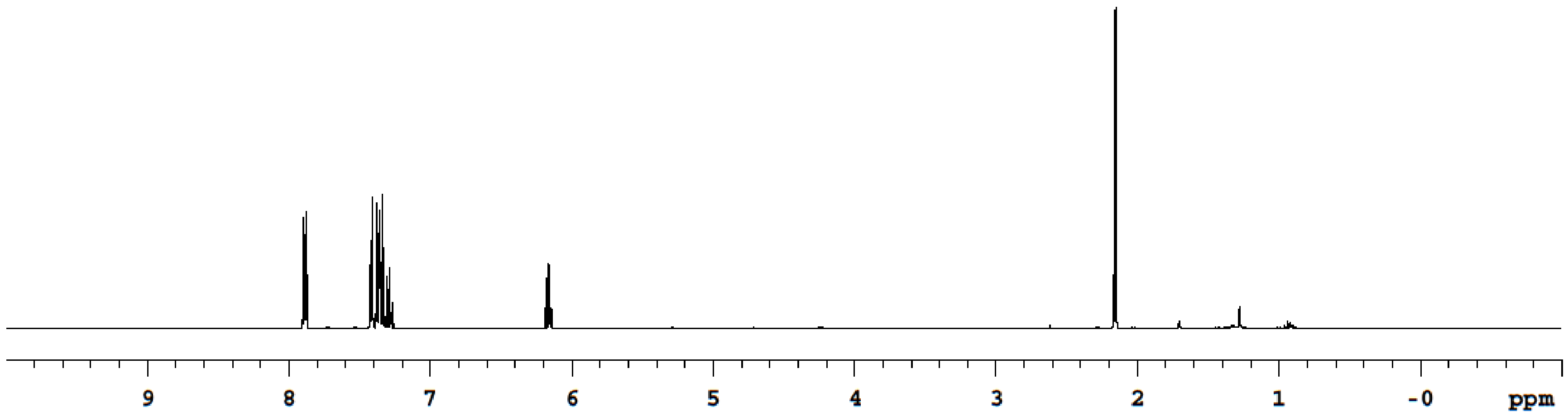

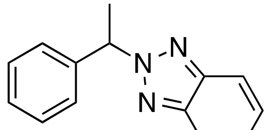

9b

RRC-1205-III-256-CNMR-CDCl3-firstspot

Pulse Sequence: s2pul

Solvent: CDCl3
Temp. 25.0 C / 298.1 K
Operator: Raghu
File: RRC-1205-III-256-CNMR-CDCl3-firstspot
INOVA-500 "riga"

Relax. delay 3.000 sec
Pulse 45.0 degrees
Acq. time 1.300 sec
Width 29996.3 Hz
12700 repetitions
OBSERVE C13, 125.6674246 MHz
DECOUPLE H1, 499.7730084 MHz
Power 40 dB
continuously on
WALTZ-16 modulated
DATA PROCESSING
Line broadening 2.0 Hz
FT size 131072
Total time 29 hr, 55 min, 35 sec

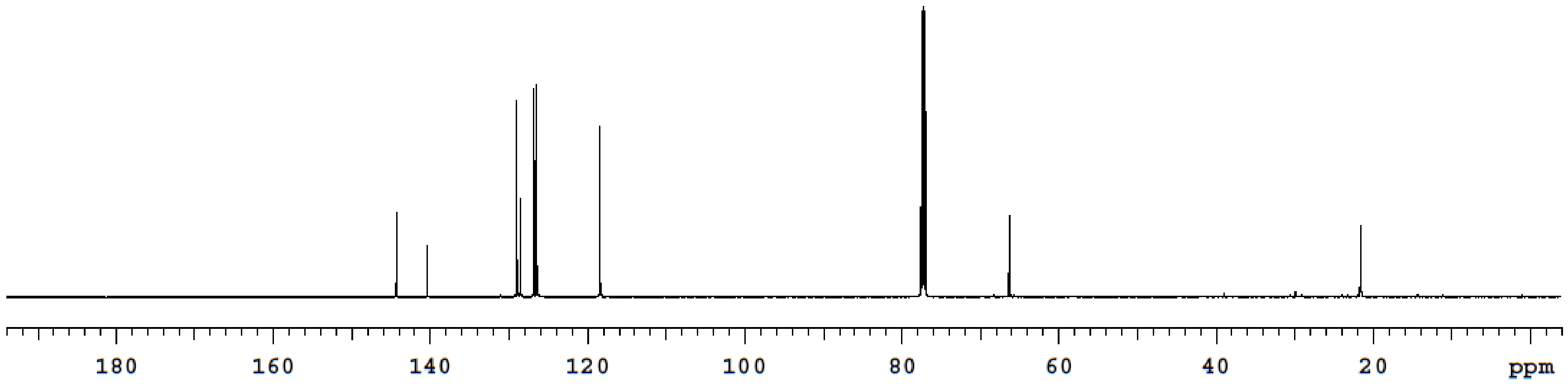

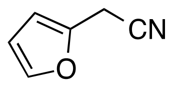

10

1205-Vijay-03-54-1H-Pure-CDCl3

Pulse Sequence: s2pul

Solvent: CDCl3
Temp. 25.0 C / 298.1 K
Operator: mkl
File:
INOVA-500 "riga"

Relax. delay 5.000 sec
Pulse 38.6 degrees
Acq. time 1.892 sec
Width 6002.4 Hz
8 repetitions
OBSERVE H1, 499.7707207 MHz
DATA PROCESSING
FT size 32768
Total time 3 min, 40 sec

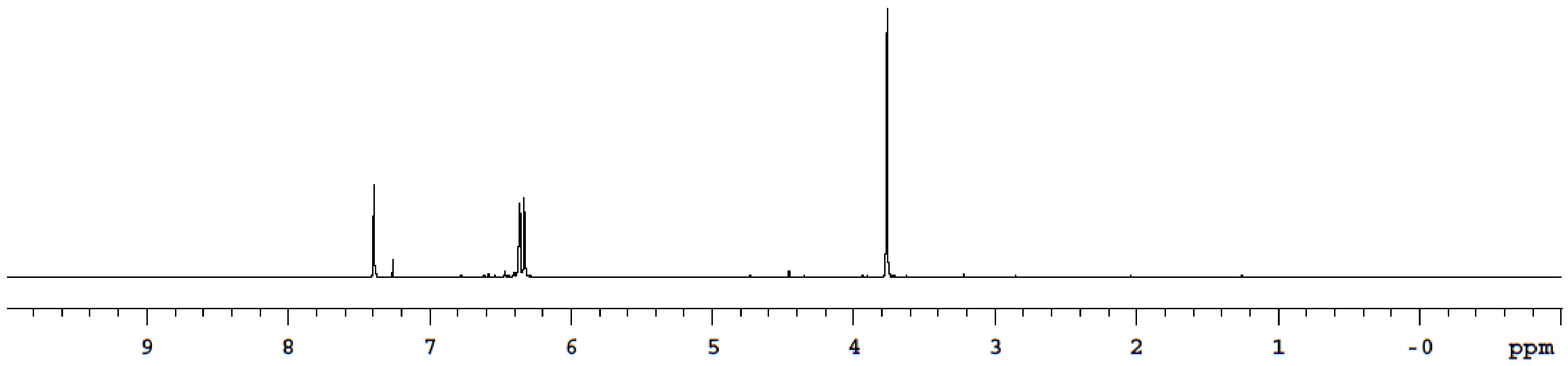

1205-Vijay-03-44-13C-CDCl3

Pulse Sequence: s2pul
Solvent: CDCl3
Ambient temperature
Operator: mkl
File:
INOVA-500 "riga"

Relax. delay 2.000 sec
Pulse 45.0 degrees
Acq. time 1.300 sec
Width 29996.3 Hz
200 repetitions
OBSERVE C13, 125.6674149 MHz
DECOUPLE H1, 499.7730084 MHz
Power 39 dB
continuously on
WALTZ-16 modulated
DATA PROCESSING
Line broadening 0.5 Hz
FT size 131072
Total time 55 min, 9 sec

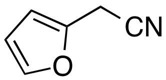

10

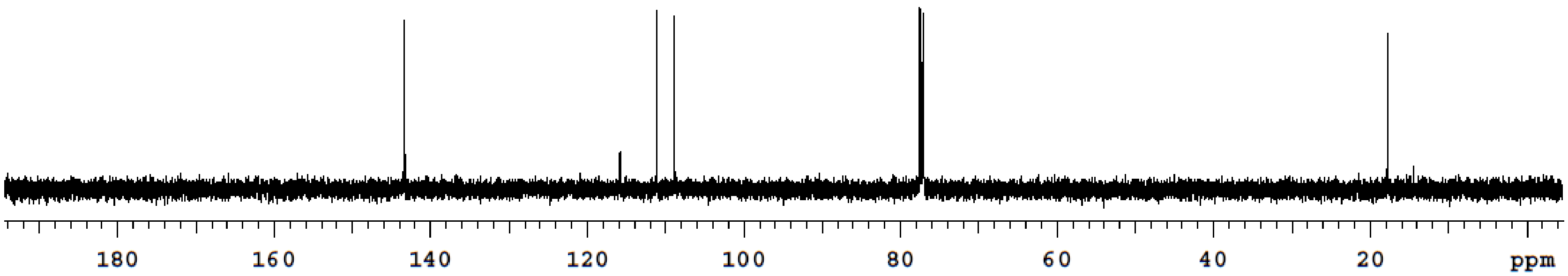

1205-Vijay-furfurylazide-1H-DMSO-D6

Pulse Sequence: s2pul

Solvent: dmso
Temp. 25.0 C / 298.1 K
Operator: mkl
File: 1205-Vijay-2furfurylazide-1H-DMSO-D6
INOVA-500 "riga"

Pulse 34.7 degrees
Acq. time 1.892 sec
Width 8000.0 Hz
32 repetitions
OBSERVE H1, 499.7730834 MHz
DATA PROCESSING
FT size 32768
Total time 1 min, 0 sec

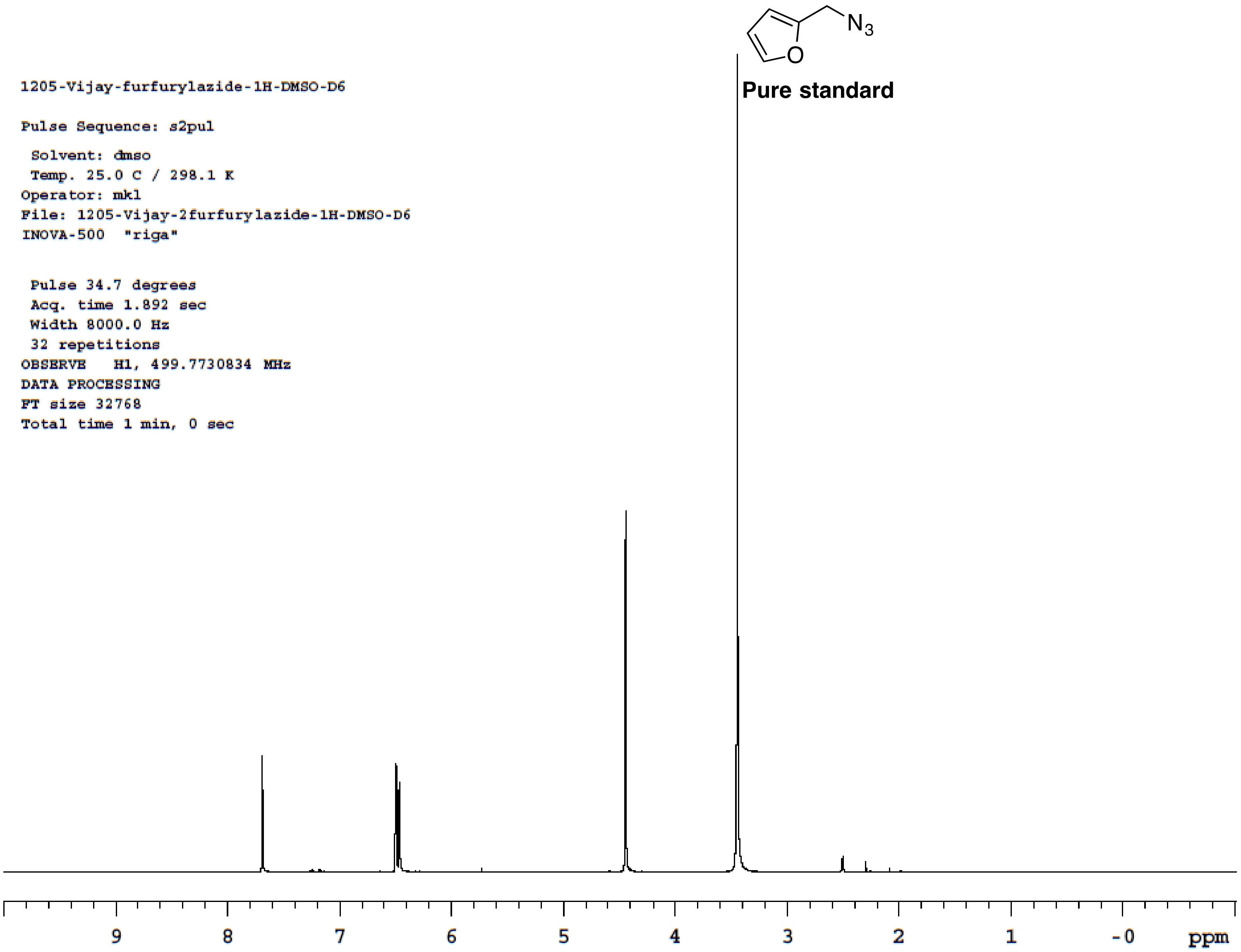

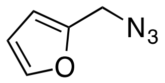

Reaction mixture containing 11

1205-Vijay-03-72-1H-DMSO-D6

Pulse Sequence: s2pul

Solvent: DMSO
Ambient temperature
Operator: mkl
File: 1205-Vijay-03-72-1H-DMSO-D6
INOVA-500 "riga"

Relax. delay 2.000 sec
Pulse 34.7 degrees
Acq. time 1.892 sec
Width 8000.0 Hz
32 repetitions
OBSERVE H1, 499.7730834 MHz
DATA PROCESSING
FT size 32768
Total time 2 min, 4 sec

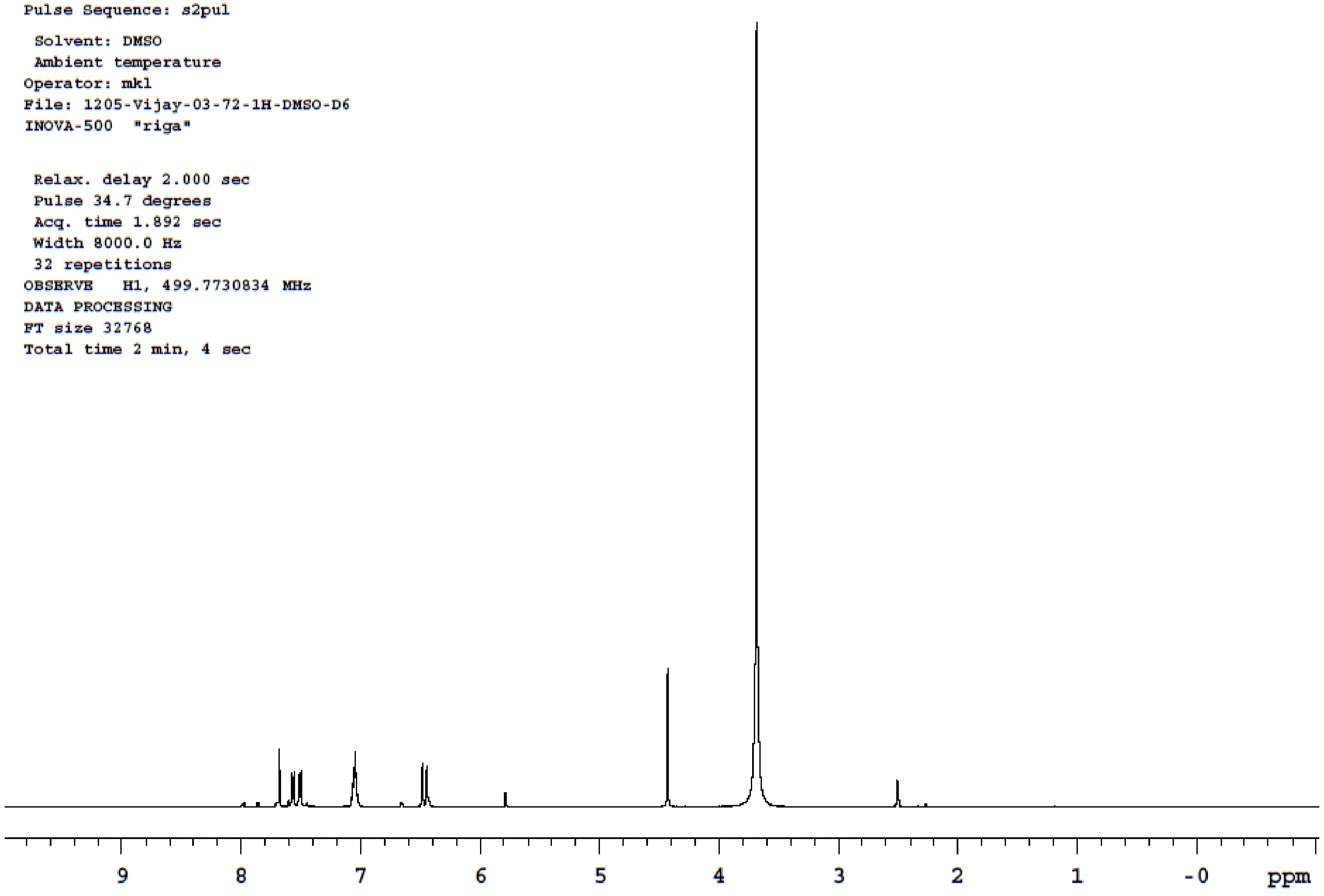

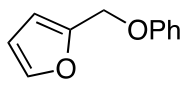

12

1205-Vijay-03-40-1H-CDCl3

Pulse Sequence: s2pul

Solvent: CDCl3
Temp. 25.0 C / 298.1 K
Operator: mkl
File:
INOVA-500 "riga"

Relax. delay 5.000 sec
Pulse 38.6 degrees
Acq. time 1.892 sec
Width 6002.4 Hz
32 repetitions
OBSERVE H1, 499.7707095 MHz
DATA PROCESSING
FT size 32768
Total time 3 min, 40 sec

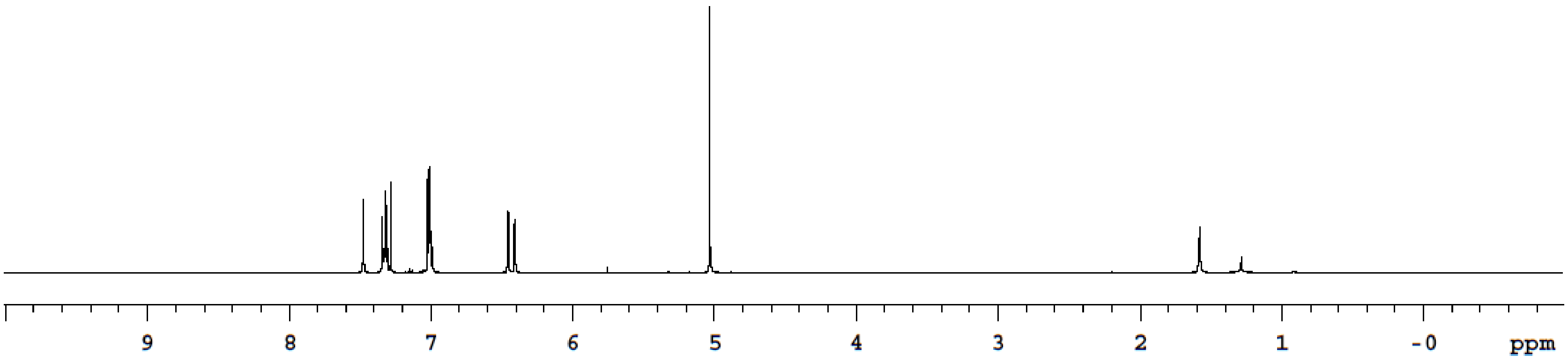

1205-Vijay-03-40-13C-CDCl3

Pulse Sequence: s2pul

Solvent: CDCl3
Ambient temperature
Operator: mkl
File:
INOVA-500 "riga"

Relax. delay 2.000 sec
Pulse 45.0 degrees
Acq. time 1.300 sec
Width 29996.3 Hz
1200 repetitions
OBSERVE C13, 125.6674149 MHz
DECOUPLE H1, 499.7730084 MHz
Power 39 dB
continuously on
WALTZ-16 modulated
DATA PROCESSING
Line broadening 0.5 Hz
FT size 131072
Total time 1 hr, 6 min, 11 sec

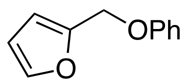

12

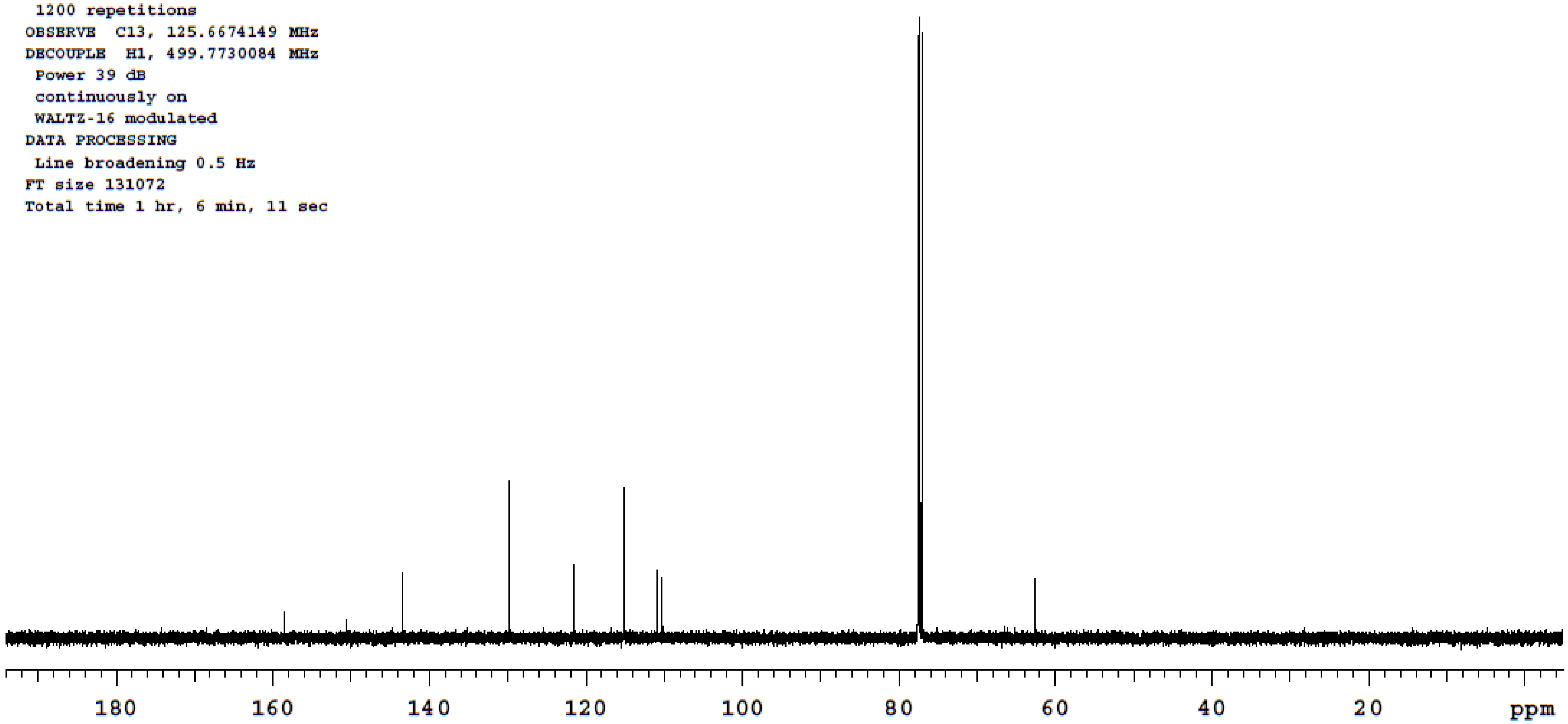

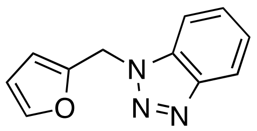

13a

1205-Vijay-03-42-21H-CDCl3

Pulse Sequence: s2pul

Solvent: CDCl3
Temp. 25.0 C / 298.1 K
Operator: mkl
File:
INOVA-500 "riga"

Relax. delay 5.000 sec
Pulse 38.6 degrees
Acq. time 1.892 sec
Width 6002.4 Hz
32 repetitions
OBSERVE H1, 499.7707095 MHz
DATA PROCESSING
FT size 32768
Total time 3 min, 40 sec

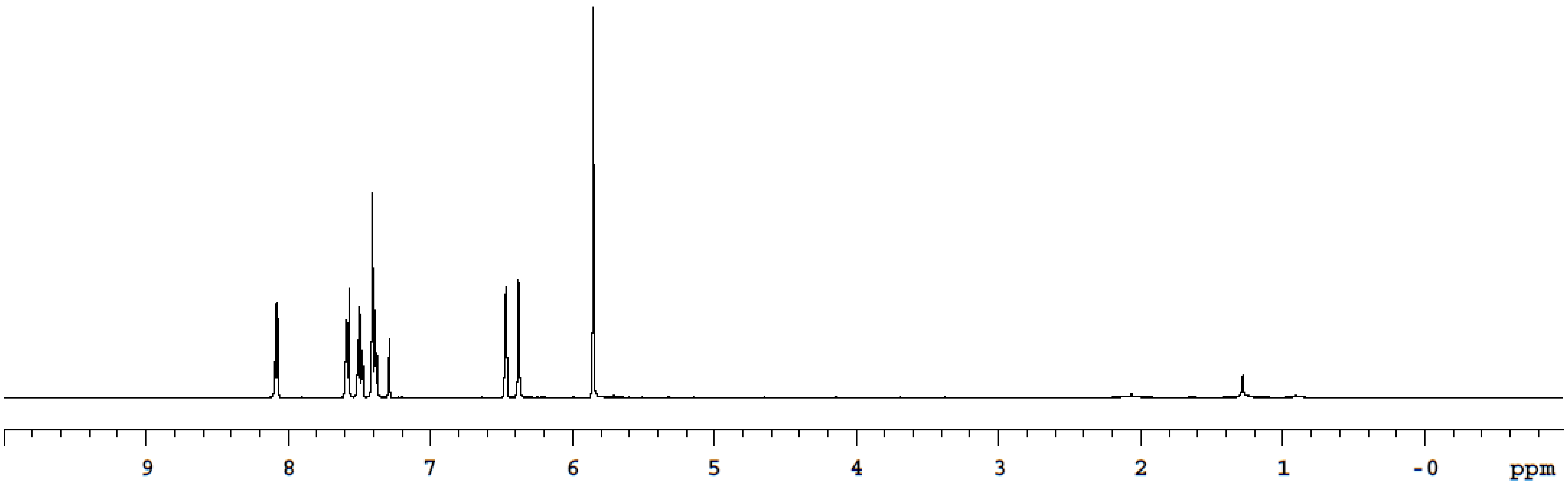

1205-Vijay-03-68-2-13C-CDCl3

Pulse Sequence: s2pul

Solvent: CDCl3
Ambient temperature
Operator: mkl
File:
INOVA-500 "riga"

Relax. delay 2.000 sec
Pulse 45.0 degrees
Acq. time 1.300 sec
Width 25000.0 Hz
316 repetitions
OBSERVE C13, 125.6674309 MHz
DECOUPLE H1, 499.7730084 MHz
Power 39 dB
continuously on
WALTZ-16 modulated
DATA PROCESSING
Line broadening 0.5 Hz
FT size 65536
Total time 2 hr, 45 min, 28 sec

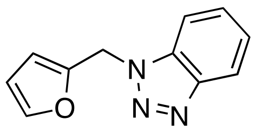

13a

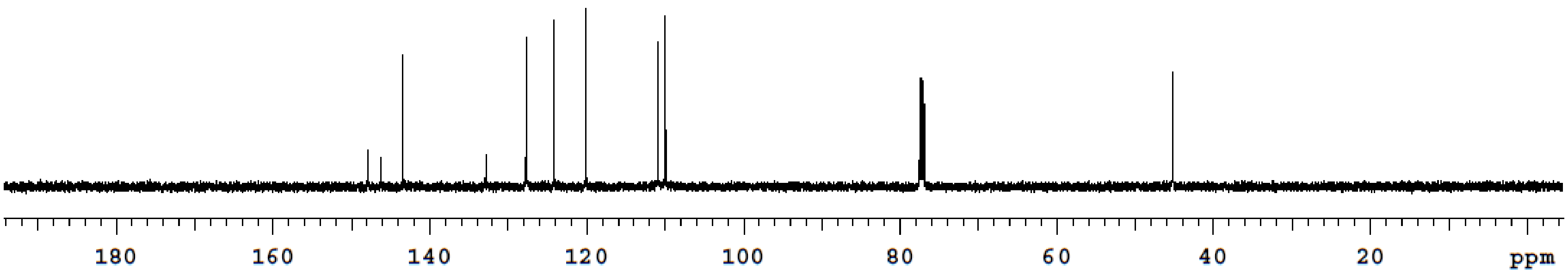

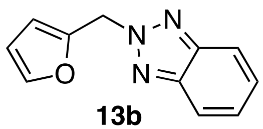

13b

1205-Vijay-03-68-1-1H-CDCl3

Pulse Sequence: s2pul

Solvent: CDCl3
Temp. 25.0 C / 298.1 K
Operator: mkl
File: 1205-Vijay-03-68-1-1H-CDCl3
INOVA-500 "riga"

Relax. delay 5.000 sec
Pulse 38.6 degrees
Acq. time 1.892 sec
Width 6002.4 Hz
32 repetitions
OBSERVE H1, 499.7707095 MHz
DATA PROCESSING
FT size 32768
Total time 14 min, 43 sec

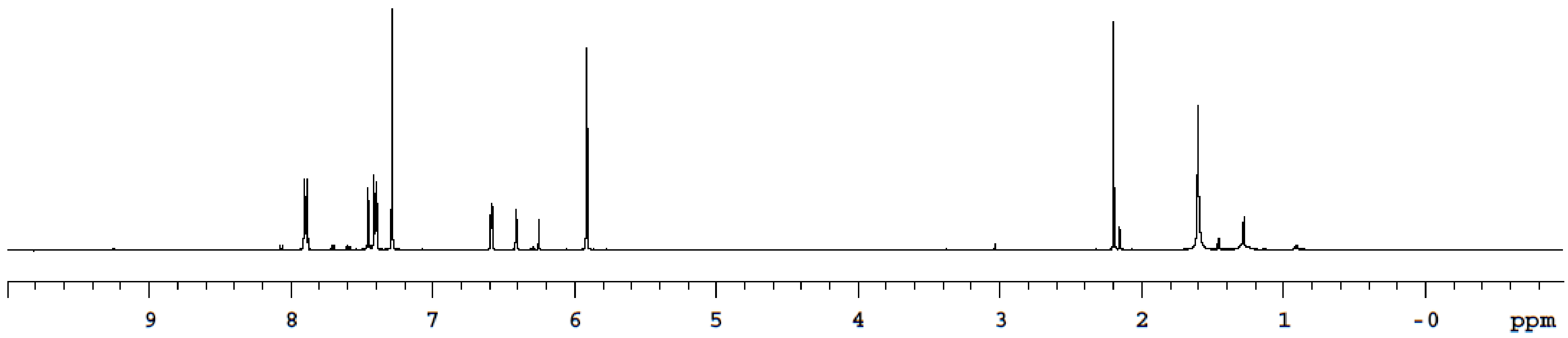

MKS-1205-11-74-CDCl3-2ndCC

Pulse Sequence: s2pul

Solvent: cdcl3
Temp. 25.0 C / 298.1 K
Operator: mkl
File: MKS-1205-11-74-CDCl3-2ndCC
INOVA-500 "riga"

Pulse 45.0 degrees
Acq. time 1.892 sec
Width 8000.0 Hz
80 repetitions
OBSERVE H1, 499.7707217 MHz
DATA PROCESSING
FT size 32768
Total time 6 min, 20 sec

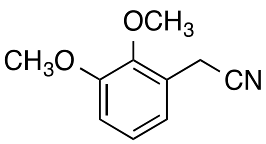

14

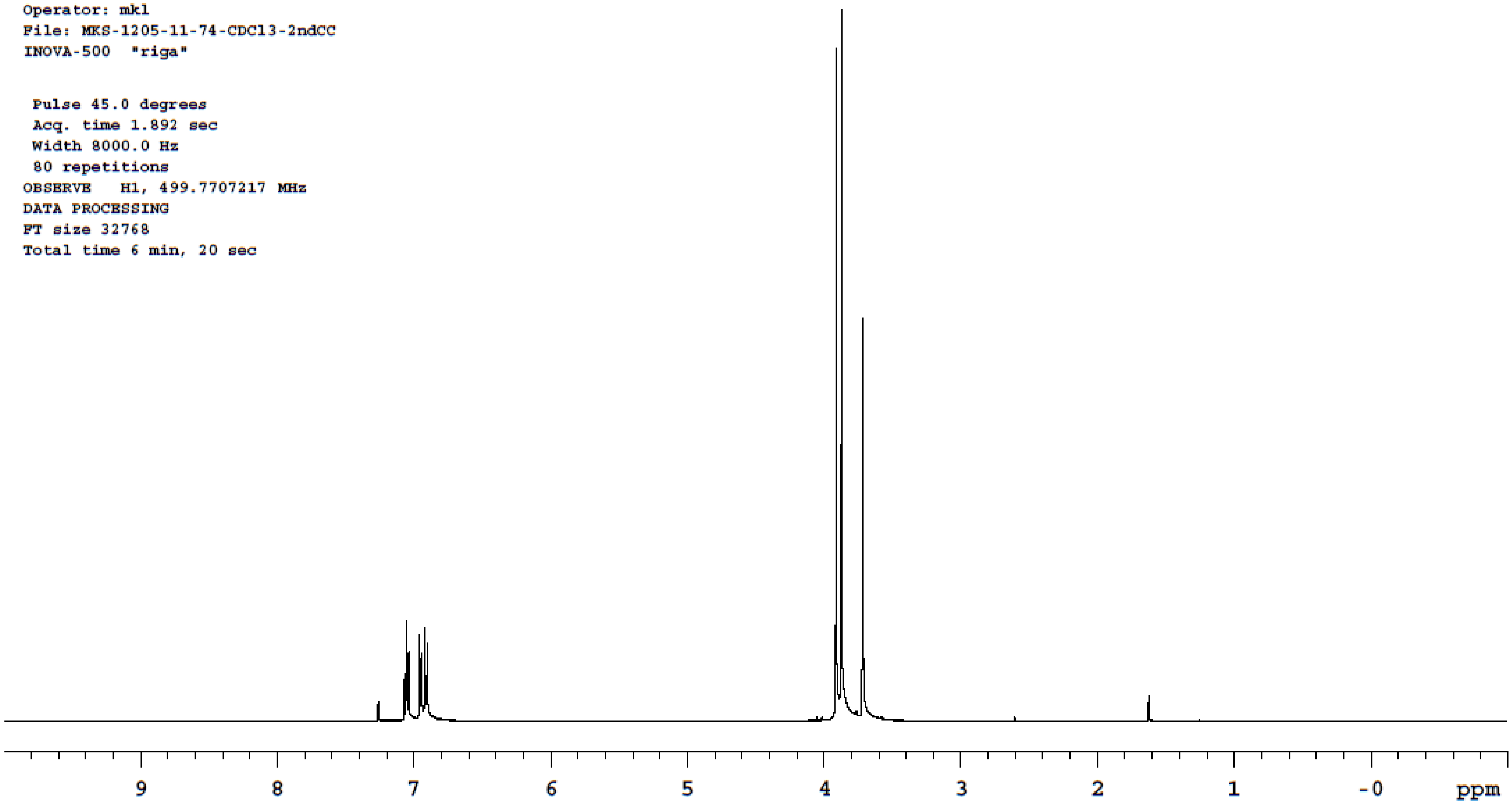

MKS-1205-11-74-CDCl3-13C-CC

Pulse Sequence: s2pul

Solvent: CDCl3
Temp. 25.0 C / 298.1 K
Operator: mkl
File: MKS-1205-11-74-CDCl3-13C-CC
INOVA-500 "riga"

Relax. delay 3.000 sec
Pulse 45.0 degrees
Acq. time 1.300 sec
Width 25000.0 Hz
780 repetitions
OBSERVE C13, 125.6674271 MHz
DECOUPLE H1, 499.7730084 MHz
Power 39 dB
continuously on
WALTZ-16 modulated
DATA PROCESSING
Line broadening 0.2 Hz
FT size 65536
Total time 2 hr, 27 min, 5 sec

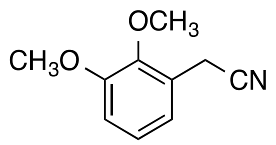

14

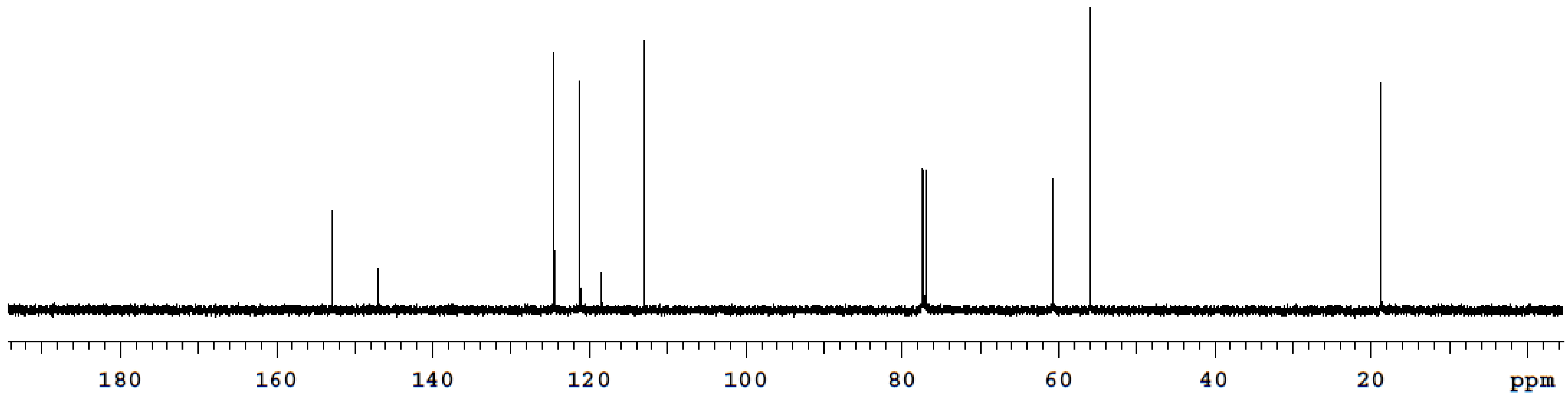

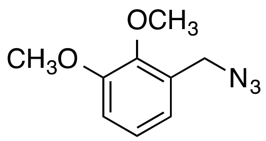

15

```
MKS-1205-12-36-CDC13-CC

Pulse Sequence: s2pul
 Solvent: cdcl3
 Temp. 25.0 C / 298.1 K
Operator: mkl
File: MKS-1205-12-36-CDC13-CC
INOVA-500  "riga"

 Pulse 45.0 degrees
 Acq. time 1.892 sec
 Width 8000.0 Hz
 44 repetitions
OBSERVE   H1, 499.7707212 MHz
DATA PROCESSING
 Line broadening 0.2 Hz
FT size 32768
Total time 1 hr, 3 min, 21 sec
```

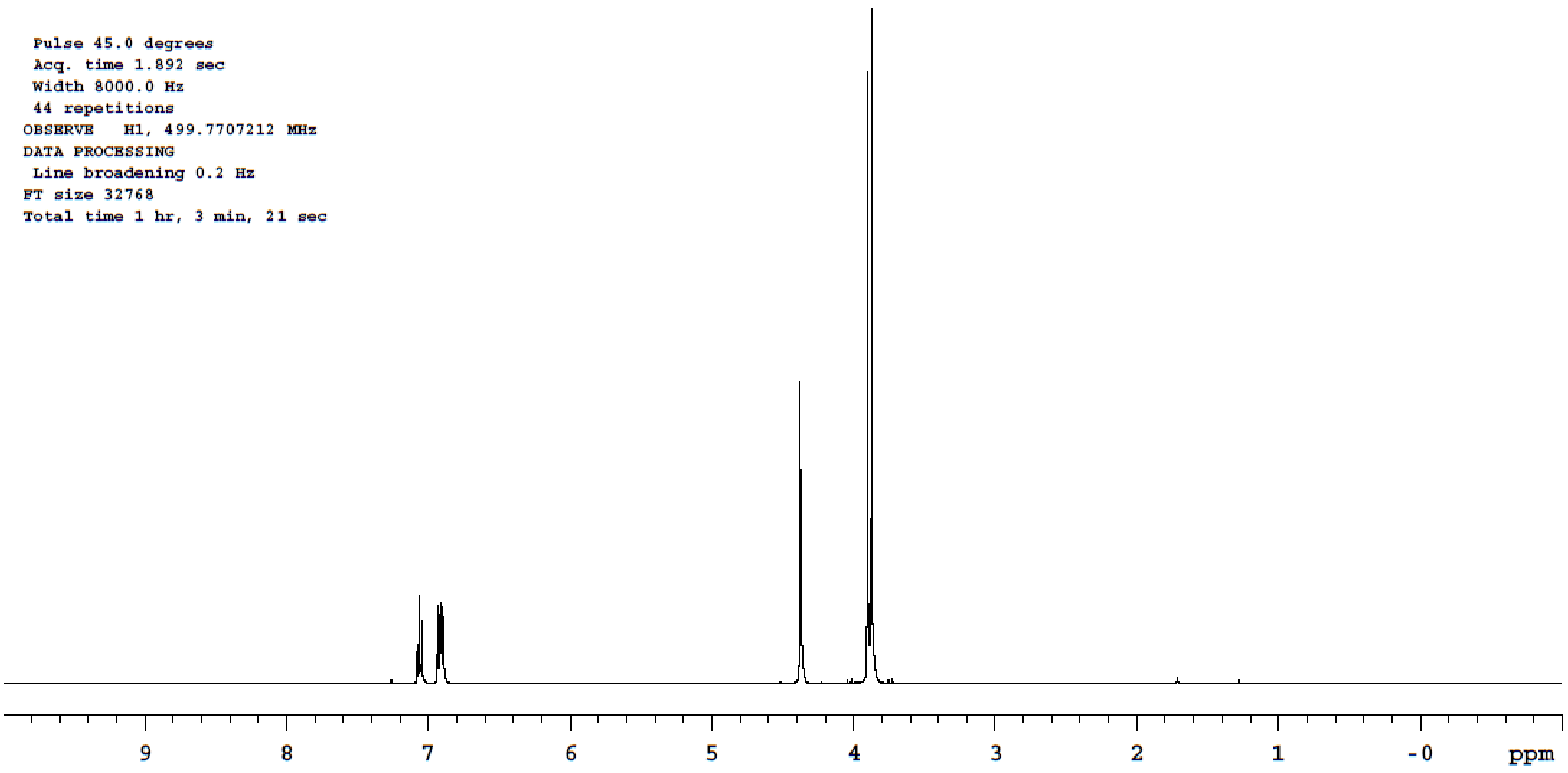

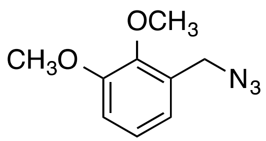

15

MKS-1205-12-36-13C-CDCl3-CC

Pulse Sequence: s2pul

Solvent: CDCl3
Temp. 25.0 C / 298.1 K
Operator: mkl
File: MKS-1205-12-36-13C-CDCl3-CC
INOVA-500 "riga"

Relax. delay 4.000 sec
Pulse 52.1 degrees
Acq. time 1.300 sec
Width 29996.3 Hz
268 repetitions
OBSERVE C13, 125.6674360 MHz
DECOUPLE H1, 499.7732084 MHz
Power 42 dB
on during acquisition
WALTZ-16 modulated
DATA PROCESSING
Line broadening 2.0 Hz
FT size 131072
Total time 294 hr, 58 min, 21 sec

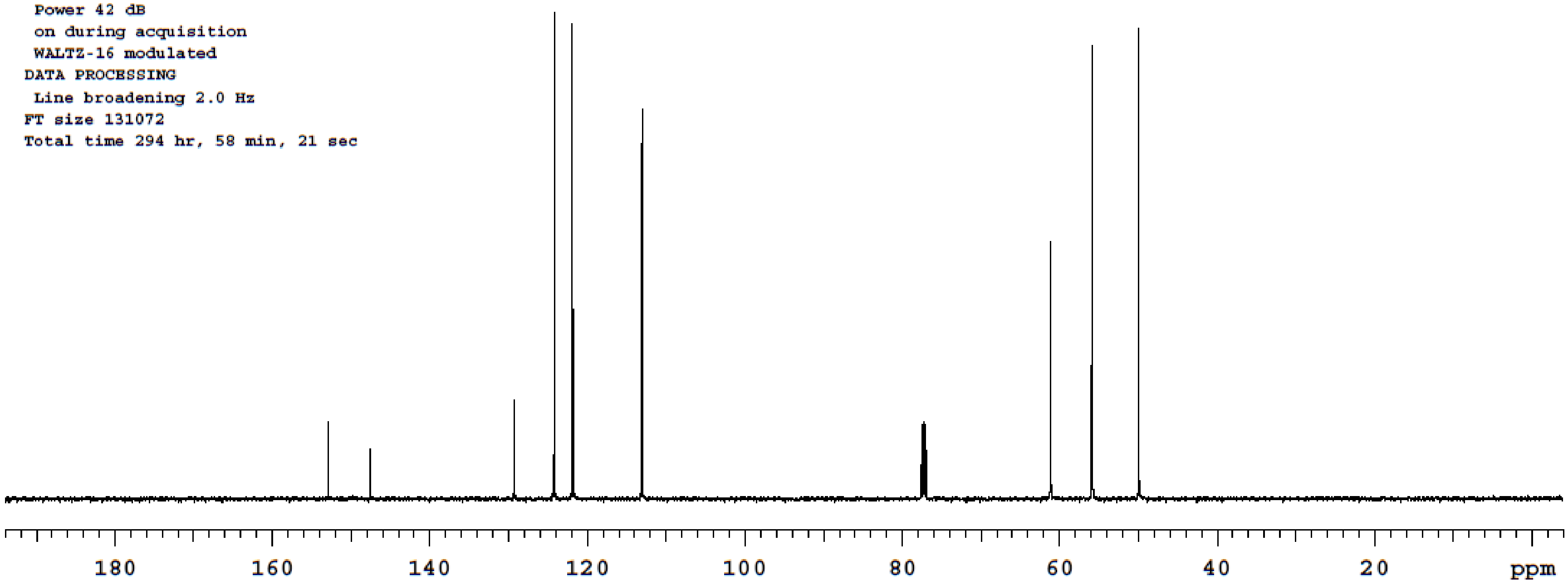

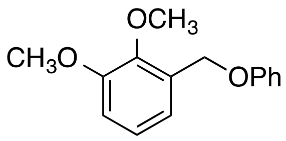

16

```
MKS-1205-12-32-CDCl3-1stFrac-CC

Pulse Sequence: s2pul

 Solvent: cdcl3
 Temp. 25.0 C / 298.1 K
Operator: mkl
File: MKS-1205-12-32-CDCl3-1stFrac-CC
INOVA-500  "riga"

 Pulse 45.0 degrees
 Acq. time 1.892 sec
 Width 8000.0 Hz
 60 repetitions
OBSERVE   H1, 499.7707095 MHz
DATA PROCESSING
 Line broadening 0.2 Hz
FT size 32768
Total time 1 hr, 3 min, 21 sec
```

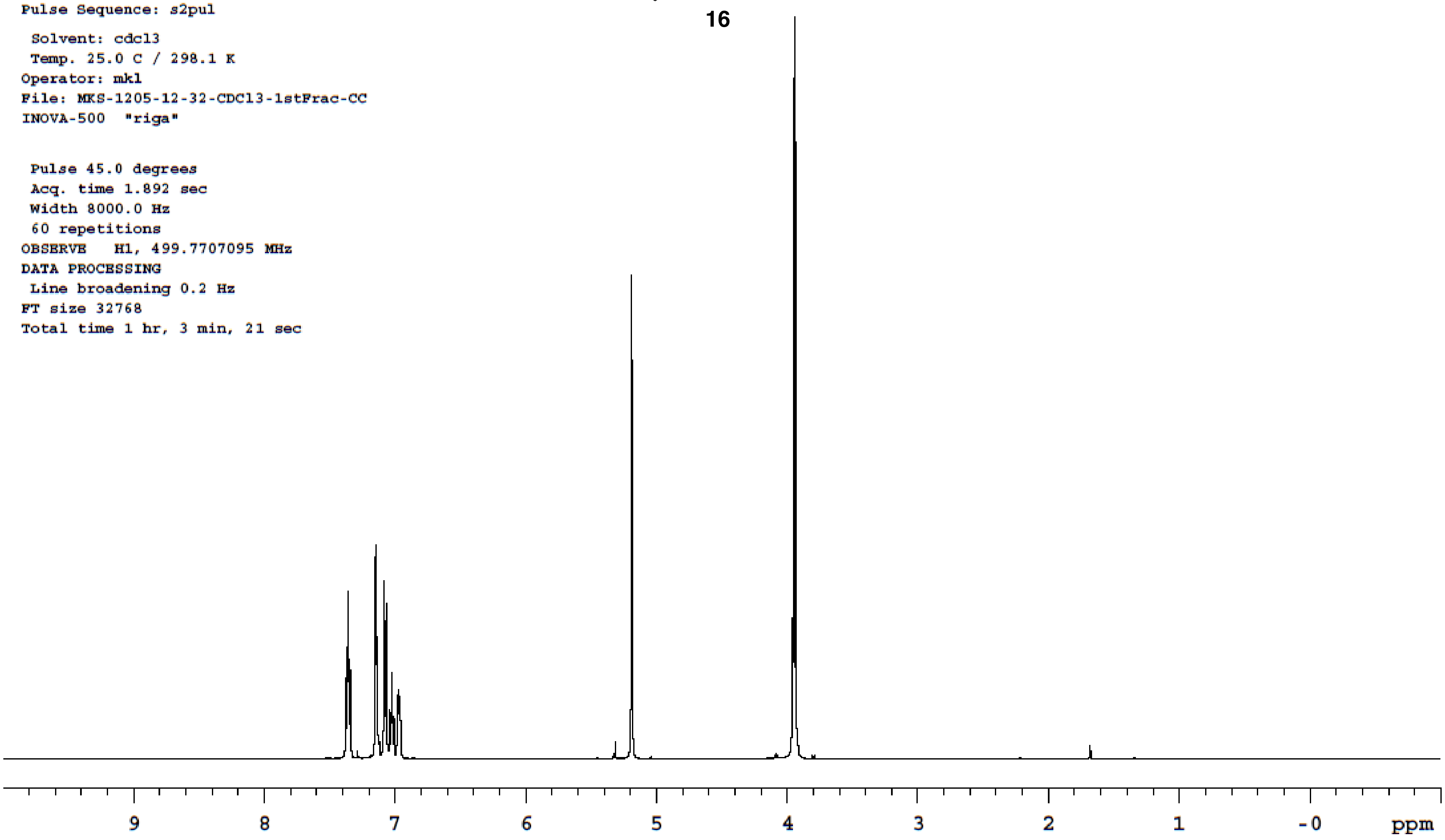

MKS-1205-12-32-CDCl3-13C-1st-Frac-CC

Pulse Sequence: s2pul

Solvent: CDCl3
Temp. 25.0 C / 298.1 K
Operator: mkl
File: MKS-1205-12-32-CDCl3-13C-1st-Frac-CC
INOVA-500 "riga"

Relax. delay 3.000 sec
Pulse 45.0 degrees
Acq. time 1.300 sec
Width 25000.0 Hz
560 repetitions
OBSERVE C13, 125.6674324 MHz
DECOUPLE H1, 499.7730084 MHz
Power 39 dB
continuously on
WALTZ-16 modulated
DATA PROCESSING
Line broadening 0.2 Hz
FT size 65536
Total time 2 hr, 27 min, 5 sec

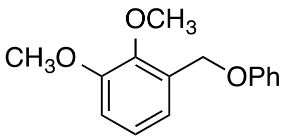

16

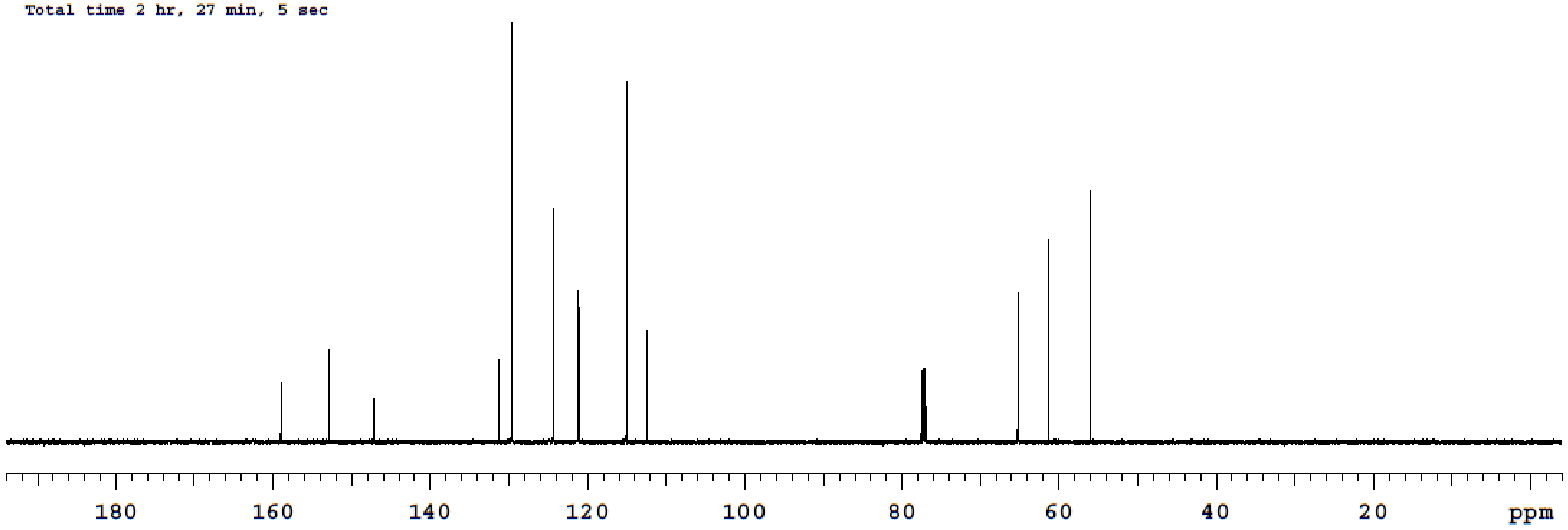

MKS-1205-12-30-CDCl3-2ndFrac-CC-Repeat

Pulse Sequence: s2pul

Solvent: cdcl3
Temp. 24.0 C / 297.1 K
Operator: mkl
File: MKS-1205-12-30-CDCl3-2ndFrac-CC-Repeat
INOVA-500 "riga"

Pulse 45.0 degrees
Acq. time 1.892 sec
Width 8000.0 Hz
56 repetitions
OBSERVE H1, 499.7707217 MHz
DATA PROCESSING
FT size 32768
Total time 6 min, 20 sec

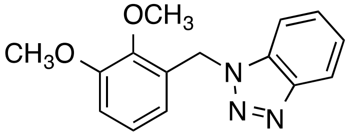

17a

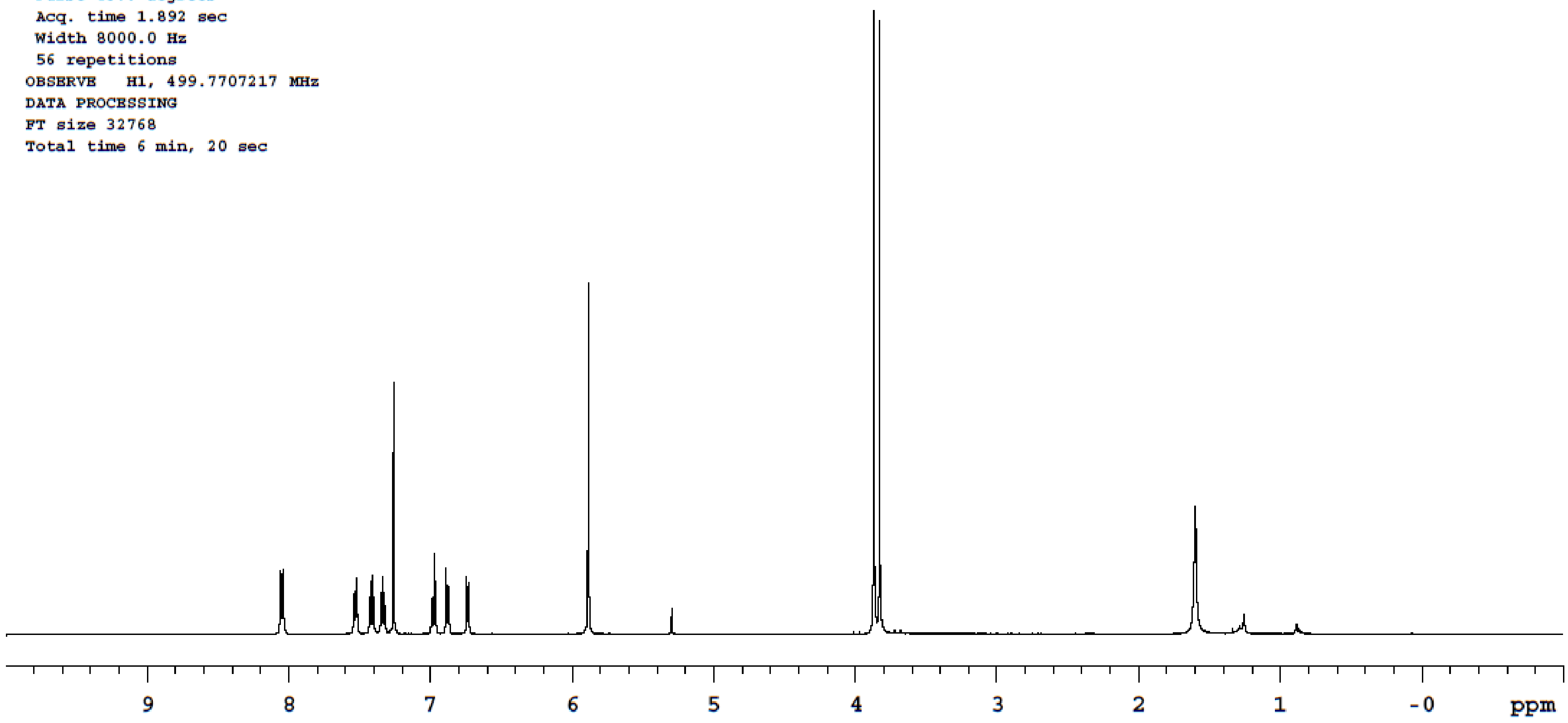

MKS-1205-12-30-CDCl3-13C-2nd-Frac-CC-repeat

Pulse Sequence: s2pul

Solvent: CDCl3
Temp. 24.0 C / 297.1 K
Operator: mkl
File: MKS-1205-12-30-CDCl3-13C-2nd-Frac-CC-repeat
INOVA-500 "riga"

Relax. delay 3.000 sec
Pulse 45.0 degrees
Acq. time 1.300 sec
Width 25000.0 Hz
2048 repetitions
OBSERVE C13, 125.6674194 MHz
DECOUPLE H1, 499.7730084 MHz
Power 39 dB
continuously on
WALTZ-16 modulated
DATA PROCESSING
Line broadening 0.2 Hz
FT size 65536
Total time 2 hr, 27 min, 5 sec

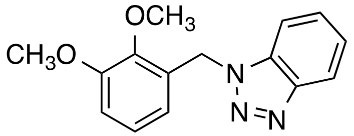

17a

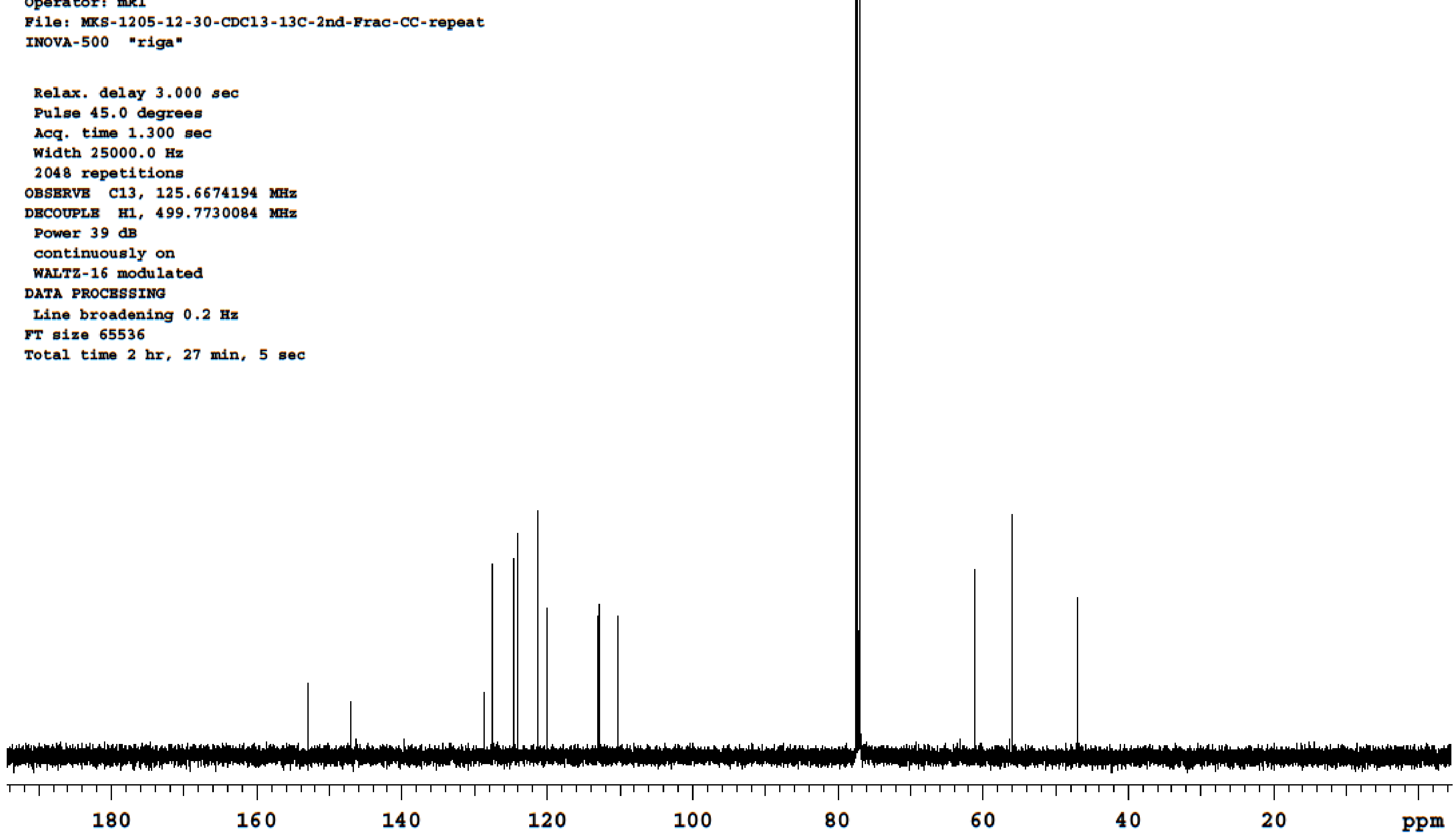

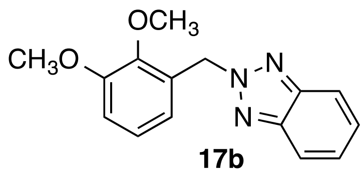

17b

MKS-1205-12-30-CDCl3-1stFrac-CC

Pulse Sequence: s2pul

Solvent: cdcl3
Temp. 25.0 C / 298.1 K
Operator: mkl
File: MKS-1205-12-30-CDCl3-1stFrac-CC
INOVA-500 "riga"

Pulse 45.0 degrees
Acq. time 1.892 sec
Width 8000.0 Hz
60 repetitions
OBSERVE H1, 499.7707217 MHz
DATA PROCESSING
FT size 32768
Total time 6 min, 20 sec

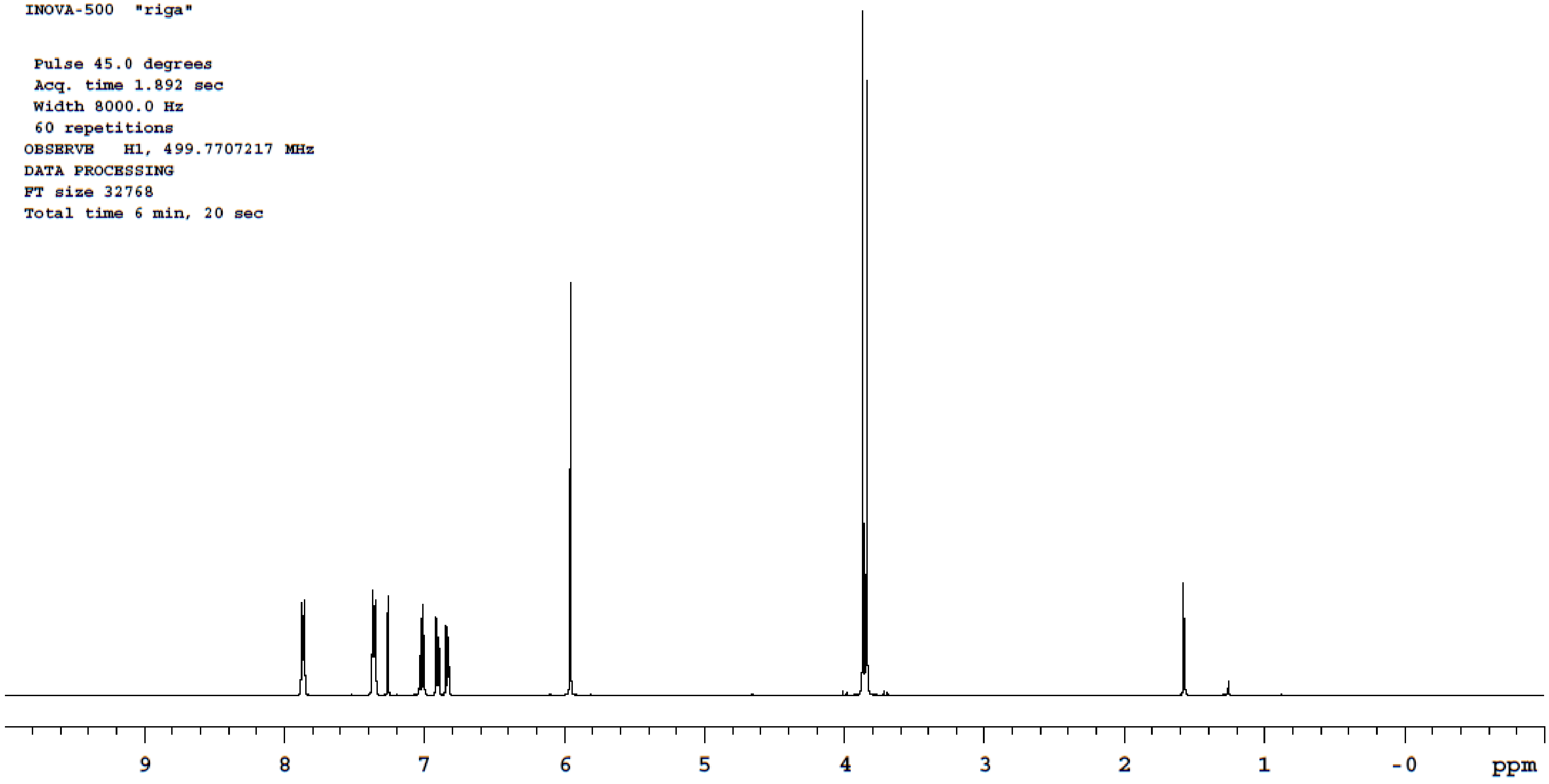

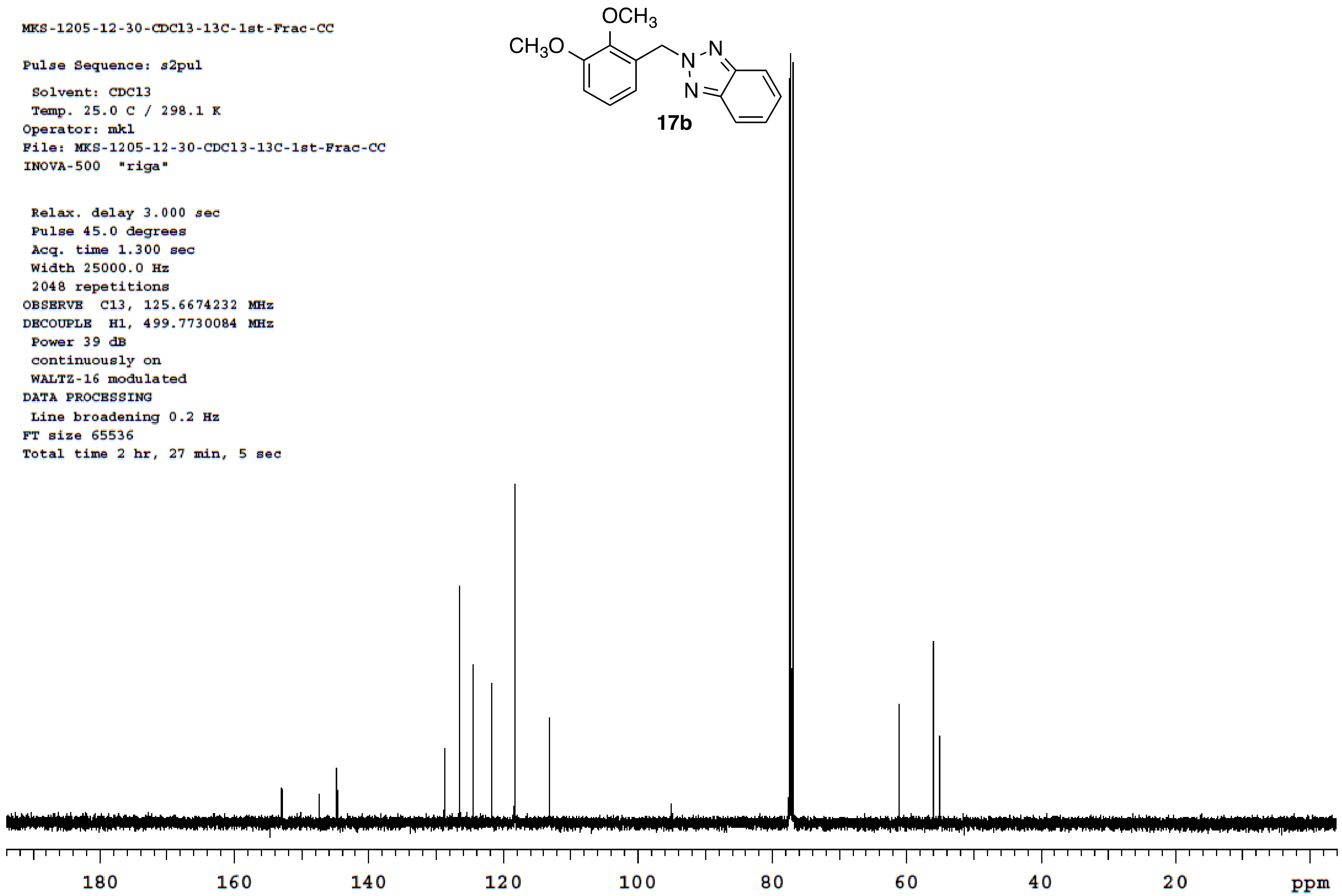
MKS-1205-12-30-CDCl3-13C-1st-Frac-CC
Pulse Sequence: s2pul
Solvent: CDCl3
Temp. 25.0 C / 298.1 K
Operator: mkl
File: MKS-1205-12-30-CDCl3-13C-1st-Frac-CC
INOVA-500 "riga"
Relax. delay 3.000 sec
Pulse 45.0 degrees
Acq. time 1.300 sec
Width 25000.0 Hz
2048 repetitions
OBSERVE C13, 125.6674232 MHz
DECOUPLE H1, 499.7730084 MHz
Power 39 dB
continuously on
WALTZ-16 modulated
DATA PROCESSING
Line broadening 0.2 Hz
FT size 65536
Total time 2 hr, 27 min, 5 sec
OCH3
CH3O
N
N
N
17b
180
160
140
120
100
80
60
40
20
ppm

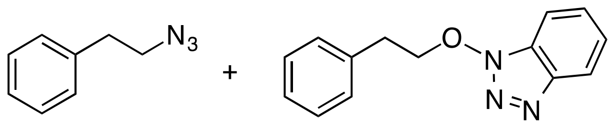

Ratio = 7.3:1

1205-Vijay-04-25-1H-CDCl3

Pulse Sequence: s2pul

Solvent: CDCl3
Temp. 25.0 C / 298.1 K
Operator: mkl
File: 1205-Vijay-04-25-completedry-1H-CDCl3
INOVA-500 "riga"

Relax. delay 5.000 sec
Pulse 38.6 degrees
Acq. time 1.892 sec
Width 6002.4 Hz
20 repetitions
OBSERVE H1, 499.7707215 MHz
DATA PROCESSING
FT size 32768
Total time 3 min, 40 sec

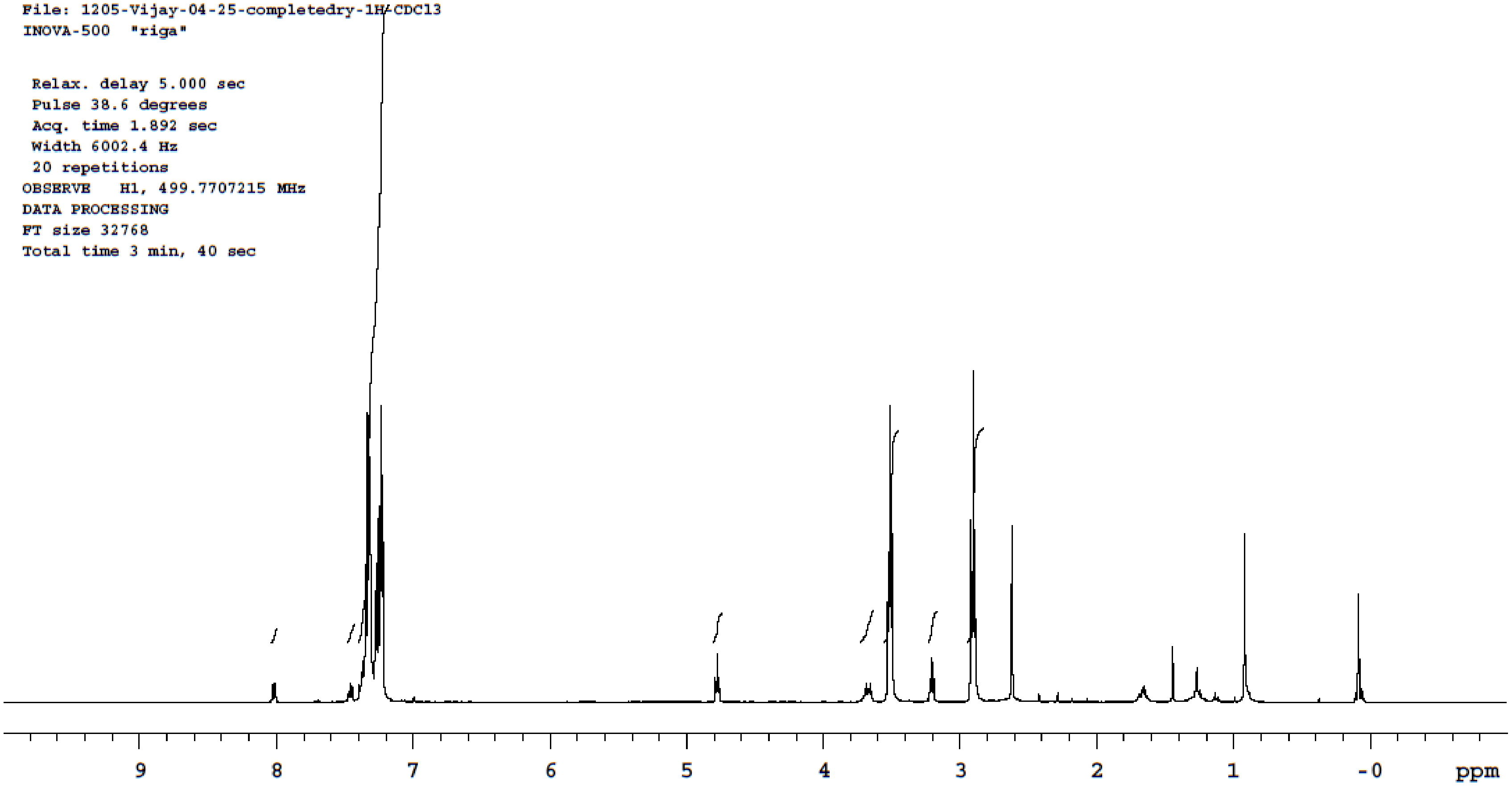

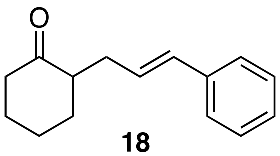

18

RRC-1205-IV-300-HNMR-CDCl3

Pulse Sequence: s2pul

Solvent: CDCl3
Temp. 25.0 C / 298.1 K
Operator: Raghu
File: RRC-1205-IV-300-HNMR-CDCl3
INOVA-500 "riga"

Relax. delay 5.000 sec
Pulse 45.0 degrees
Acq. time 1.892 sec
Width 8000.0 Hz
64 repetitions
OBSERVE H1, 499.7707212 MHz
DATA PROCESSING
Resol. enhancement 0.5 Hz
Gauss apodization 0.500 sec
FT size 32768
Total time 7 min, 21 sec

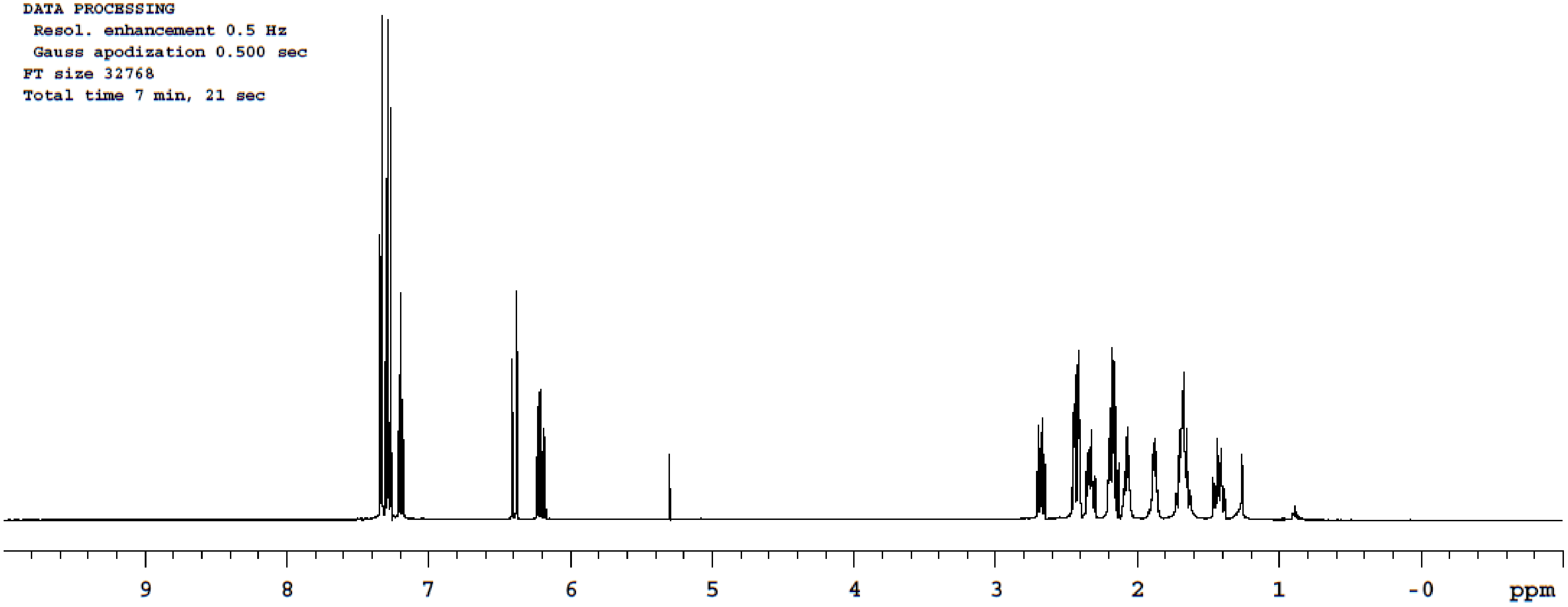

RRC-1205-IV-300-HNMR-CDCl3

Pulse Sequence: s2pul

Solvent: CDCl3
Temp. 25.0 C / 298.1 K
Operator: Raghu
File: RRC-1205-IV-300-HNMR-CDCl3
INOVA-500 "riga"

Relax. delay 5.000 sec
Pulse 45.0 degrees
Acq. time 1.892 sec
Width 8000.0 Hz
64 repetitions
OBSERVE H1, 499.7707212 MHz
DATA PROCESSING
Resol. enhancement 0.5 Hz
Gauss apodization 0.500 sec
FT size 32768
Total time 7 min, 21 sec

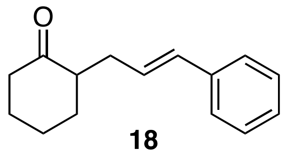

18
Expanded spectrum

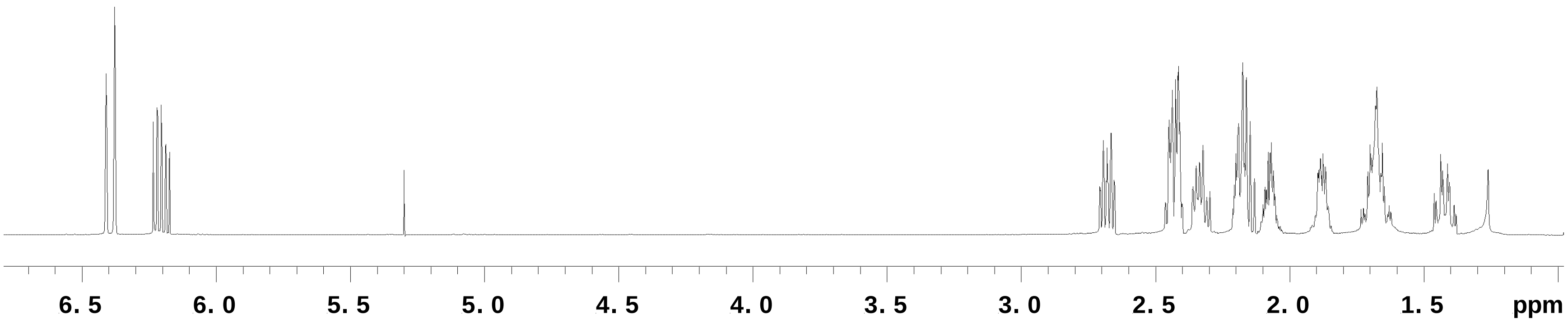

RRC-1205-IV-300-CNMR-CDCl3

Pulse Sequence: s2pul

Solvent: CDCl3
Temp. 25.0 C / 298.1 K
Operator: Raghu
File: RRC-1205-IV-300-CNMR-CDCl3
INOVA-500 "riga"

Relax. delay 3.000 sec
Pulse 45.0 degrees
Acq. time 1.300 sec
Width 29996.3 Hz
12810 repetitions
OBSERVE C13, 125.6674200 MHz
DECOUPLE H1, 499.7730084 MHz
Power 40 dB
continuously on
WALTZ-16 modulated
DATA PROCESSING
Line broadening 2.0 Hz
FT size 131072
Total time 23 hr, 56 min, 28 sec

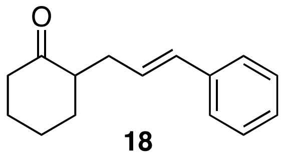

18

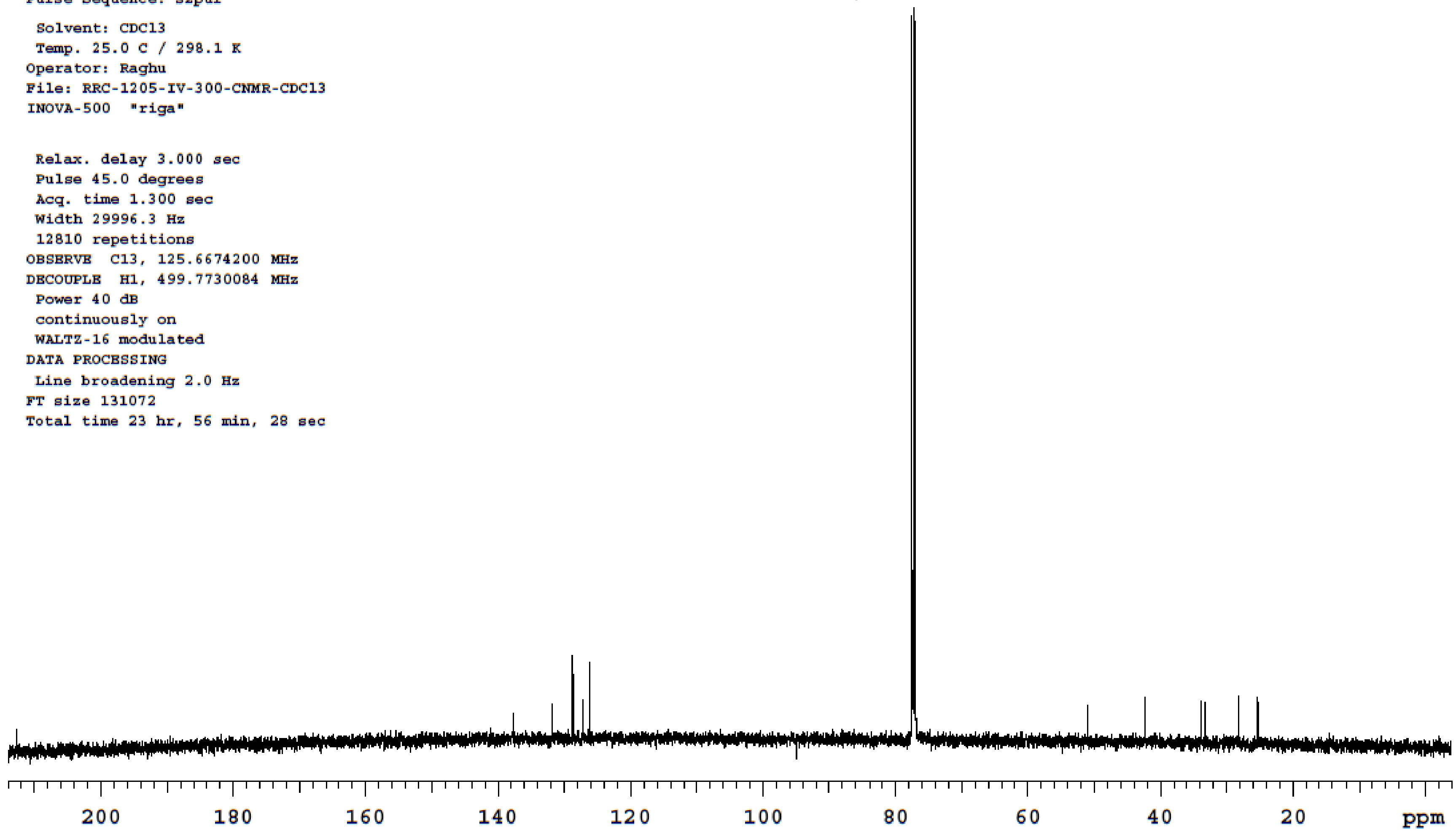

RRC-1205-IV-301-HNMR-CDCl3

Pulse Sequence: s2pul

Solvent: CDCl3
Temp. 25.0 C / 298.1 K
Operator: Raghu
File: RRC-1205-IV-301-HNMR-CDCl3
INOVA-500 "riga"

Relax. delay 5.000 sec
Pulse 45.0 degrees
Acq. time 1.892 sec
Width 8000.0 Hz
48 repetitions
OBSERVE H1, 499.7707212 MHz
DATA PROCESSING
Resol. enhancement 0.5 Hz
Gauss apodization 0.500 sec
FT size 32768
Total time 7 min, 21 sec

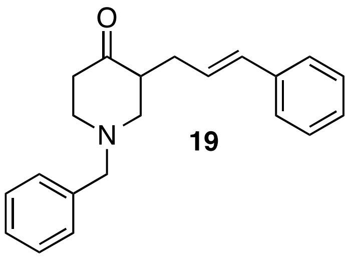

19

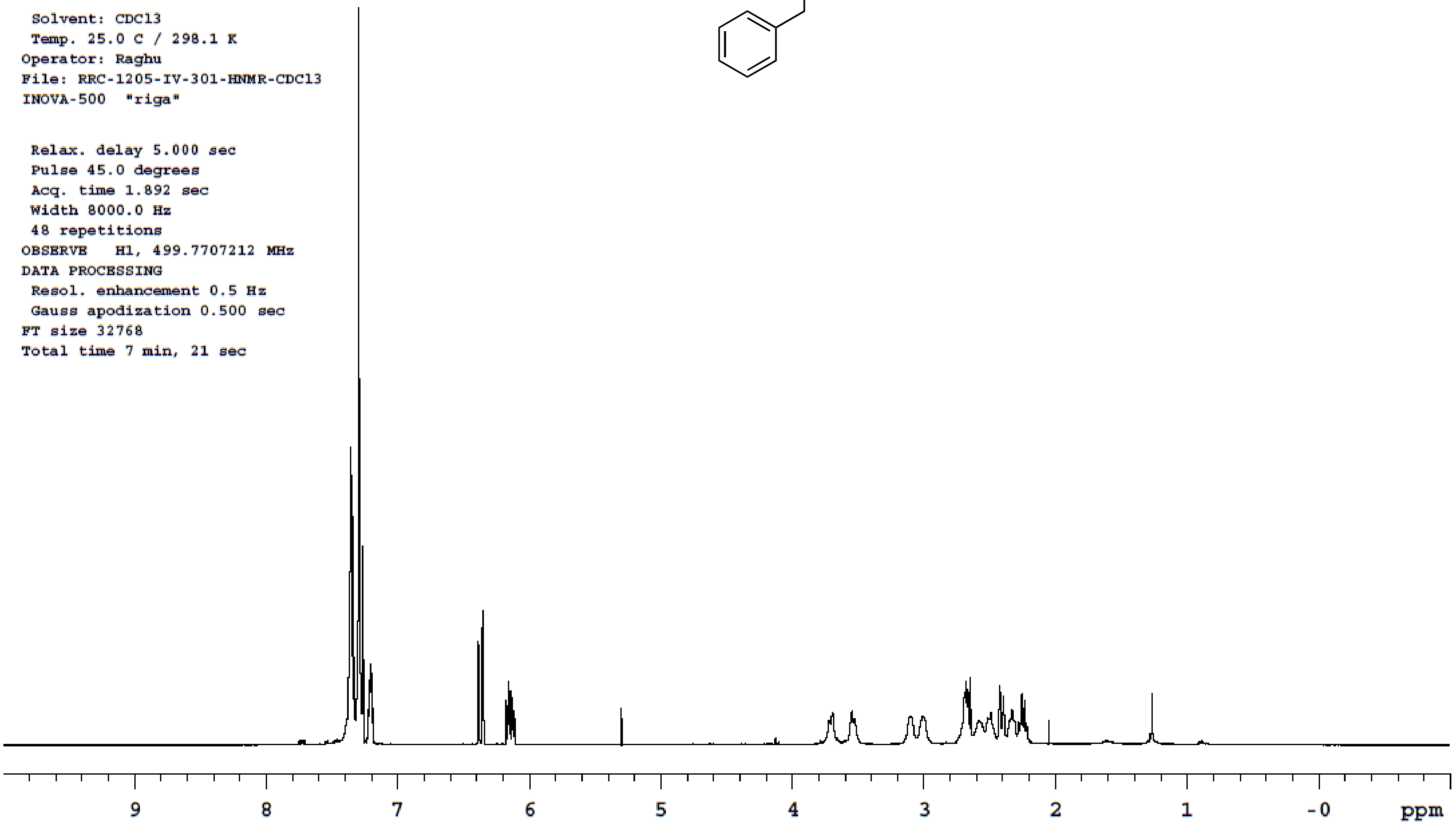

RRC-1205-IV-301-HNMR-CDCl3

Pulse Sequence: s2pul

Solvent: CDCl3
Temp. 25.0 C / 298.1 K
Operator: Raghu
File: RRC-1205-IV-301-HNMR-CDCl3
INOVA-500 "riga"

Relax. delay 5.000 sec
Pulse 45.0 degrees
Acq. time 1.892 sec
Width 8000.0 Hz
48 repetitions
OBSERVE H1, 499.7707212 MHz
DATA PROCESSING
Resol. enhancement 0.5 Hz
Gauss apodization 0.500 sec
FT size 32768
Total time 7 min, 21 sec

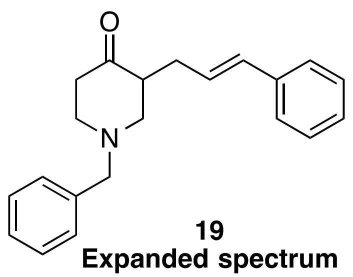

19
Expanded spectrum

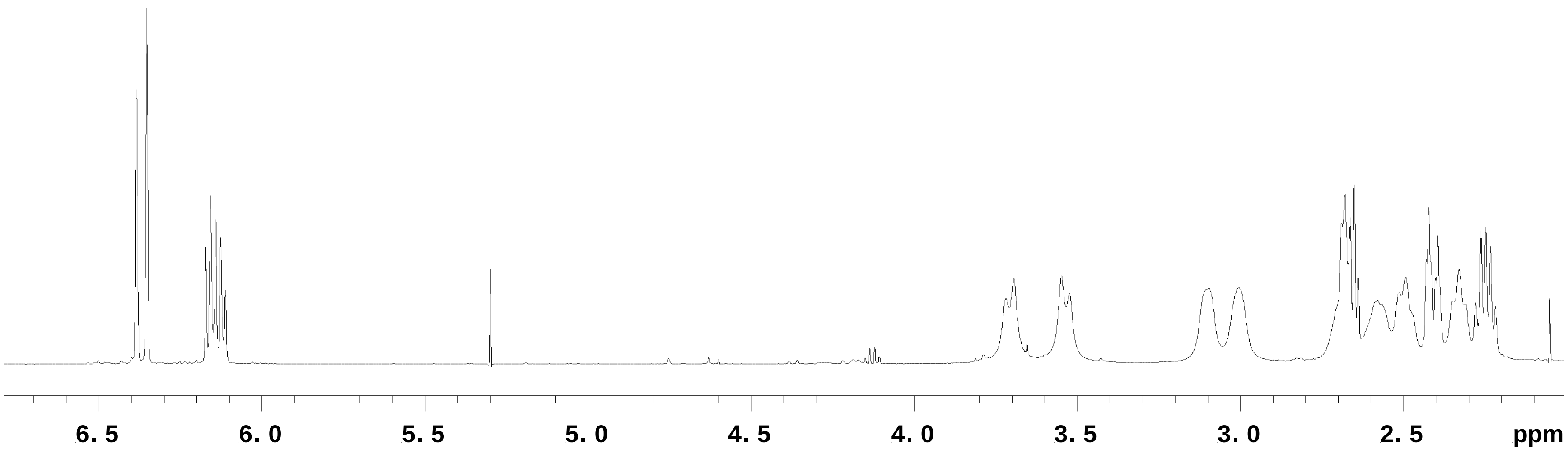

RRC-1205-IV-301-CNMR-CDCl3

Pulse Sequence: s2pul

Solvent: CDCl3
Temp. 25.0 C / 298.1 K
Operator: Raghu
File: RRC-1205-IV-301-CNMR-CDCl3
INOVA-500 "riga"

Relax. delay 3.000 sec
Pulse 45.0 degrees
Acq. time 1.300 sec
Width 29996.3 Hz
12700 repetitions
OBSERVE C13, 125.6674214 MHz
DECOUPLE H1, 499.7730084 MHz
Power 40 dB
continuously on
WALTZ-16 modulated
DATA PROCESSING
Line broadening 2.0 Hz
FT size 131072
Total time 23 hr, 56 min, 28 sec

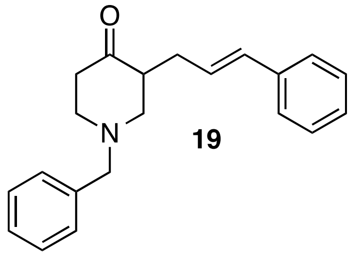

19

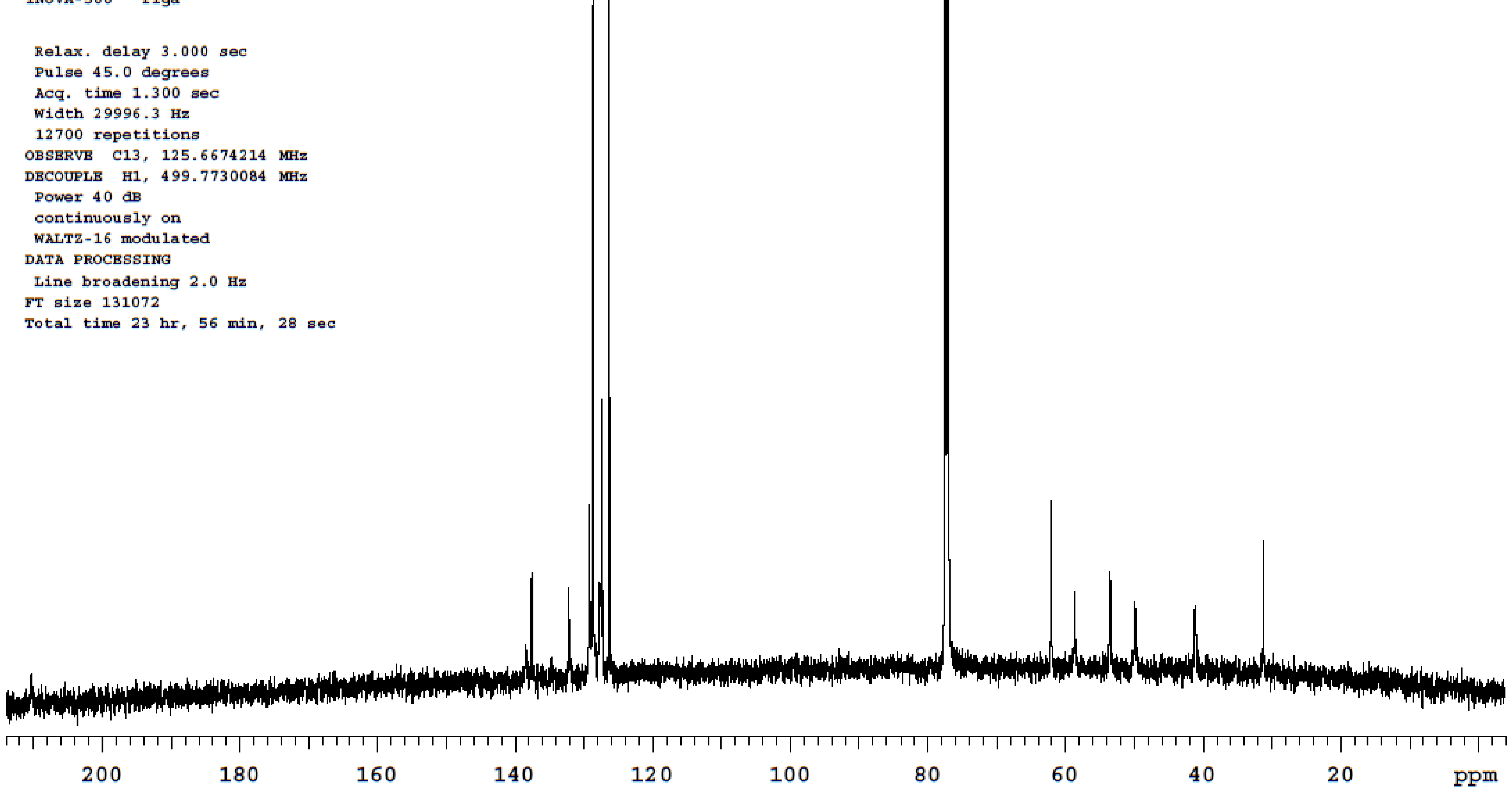

RRC-1205-IV-298-HNMR-CDCl3

Pulse Sequence: s2pul

Solvent: CDCl3
Temp. 25.0 C / 298.1 K
Operator: Raghu
File: RRC-1205-IV-298-HNMR-CDCl3
INOVA-500 "riga"

Relax. delay 5.000 sec
Pulse 45.0 degrees
Acq. time 1.892 sec
Width 8000.0 Hz
64 repetitions
OBSERVE H1, 499.7707212 MHz
DATA PROCESSING
Resol. enhancement 0.5 Hz
Gauss apodization 0.500 sec
FT size 32768
Total time 7 min, 21 sec

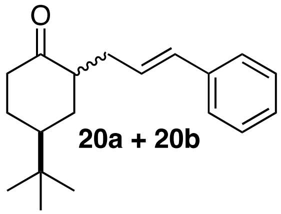

20a + 20b

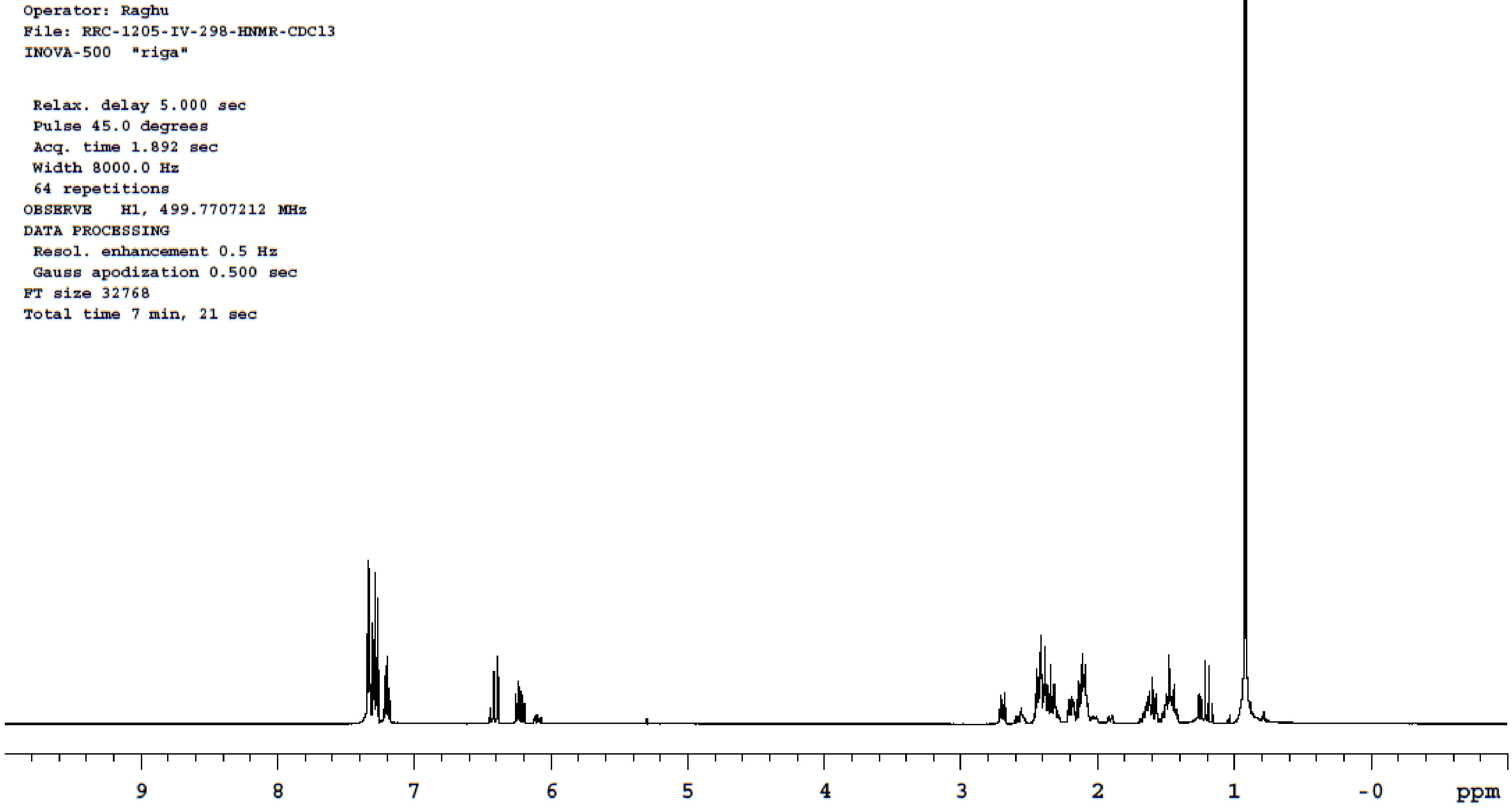

RRC-1205-IV-298-HNMR-CDCl3

Pulse Sequence: s2pul

Solvent: CDCl3
Temp. 25.0 C / 298.1 K
Operator: Raghu
File: RRC-1205-IV-298-HNMR-CDCl3
INOVA-500 "riga"

Relax. delay 5.000 sec
Pulse 45.0 degrees
Acq. time 1.892 sec
Width 8000.0 Hz
64 repetitions
OBSERVE H1, 499.7707212 MHz
DATA PROCESSING
Resol. enhancement 0.5 Hz
Gauss apodization 0.500 sec
FT size 32768
Total time 7 min, 21 sec

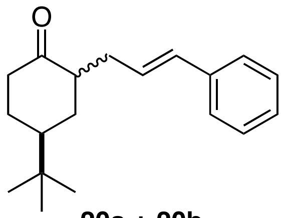

20a + 20b
Expanded spectrum

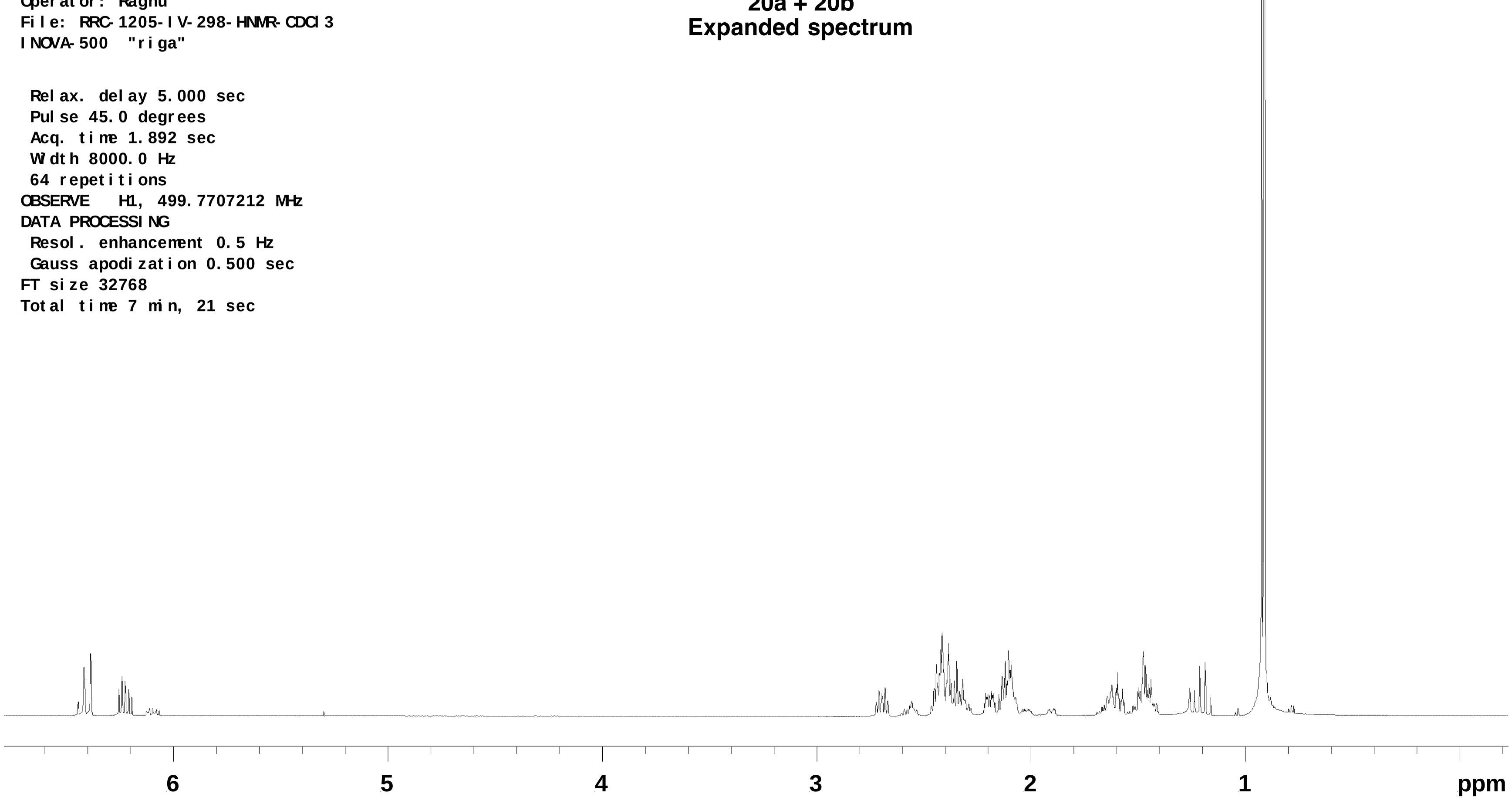

RRC-1205-IV-302-HNMR-CDCl3-majordiastereomer

Pulse Sequence: s2pul

Solvent: CDCl3
Temp. 25.0 C / 298.1 K
Operator: Raghu
File: RRC-1205-IV-302-HNMR-CDCl3-majordiastereomer
INOVA-500 "riga"

Relax. delay 5.000 sec
Pulse 45.0 degrees
Acq. time 1.892 sec
Width 8000.0 Hz
64 repetitions
OBSERVE H1, 499.7707212 MHz
DATA PROCESSING
Resol. enhancement 0.5 Hz
Gauss apodization 0.500 sec
FT size 32768
Total time 7 min, 21 sec

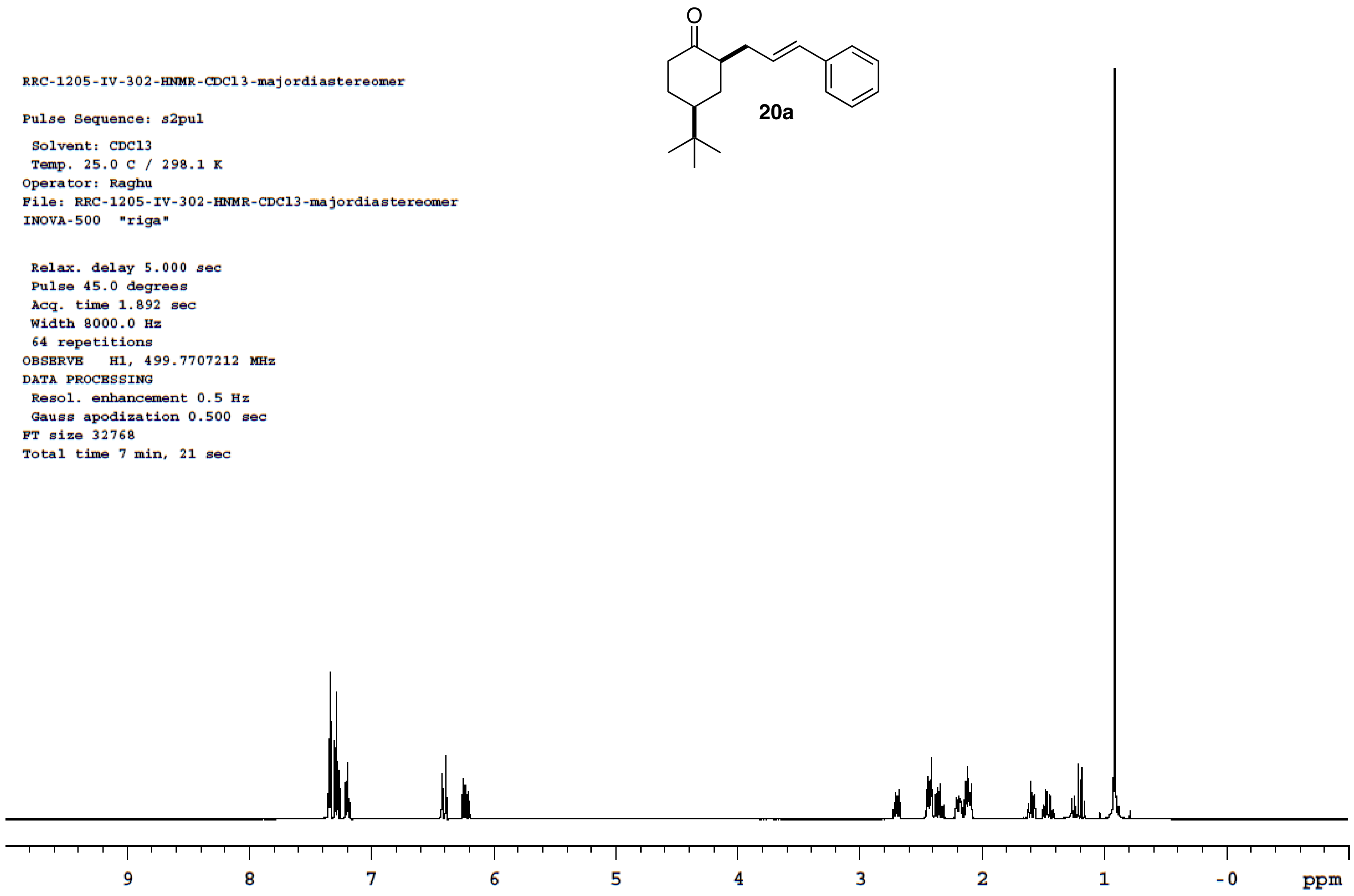

20a

RRC-1205-IV-302-HNMR-CDCl3-major diastereomer

Pulse Sequence: s2pul

Solvent: CDCl3
Temp. 25.0 C / 298.1 K
Operator: Raghu
File: RRC-1205-IV-302-HNMR-CDCl3-major diastereomer
INOVA-500 "riga"

Relax. delay 5.000 sec
Pulse 45.0 degrees
Acq. time 1.892 sec
Width 8000.0 Hz
64 repetitions
OBSERVE H1, 499.7707212 MHz
DATA PROCESSING
Resol. enhancement 0.5 Hz
Gauss apodization 0.500 sec
FT size 32768
Total time 7 min, 21 sec

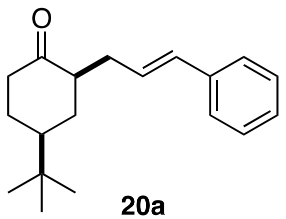

20a
Expanded spectrum

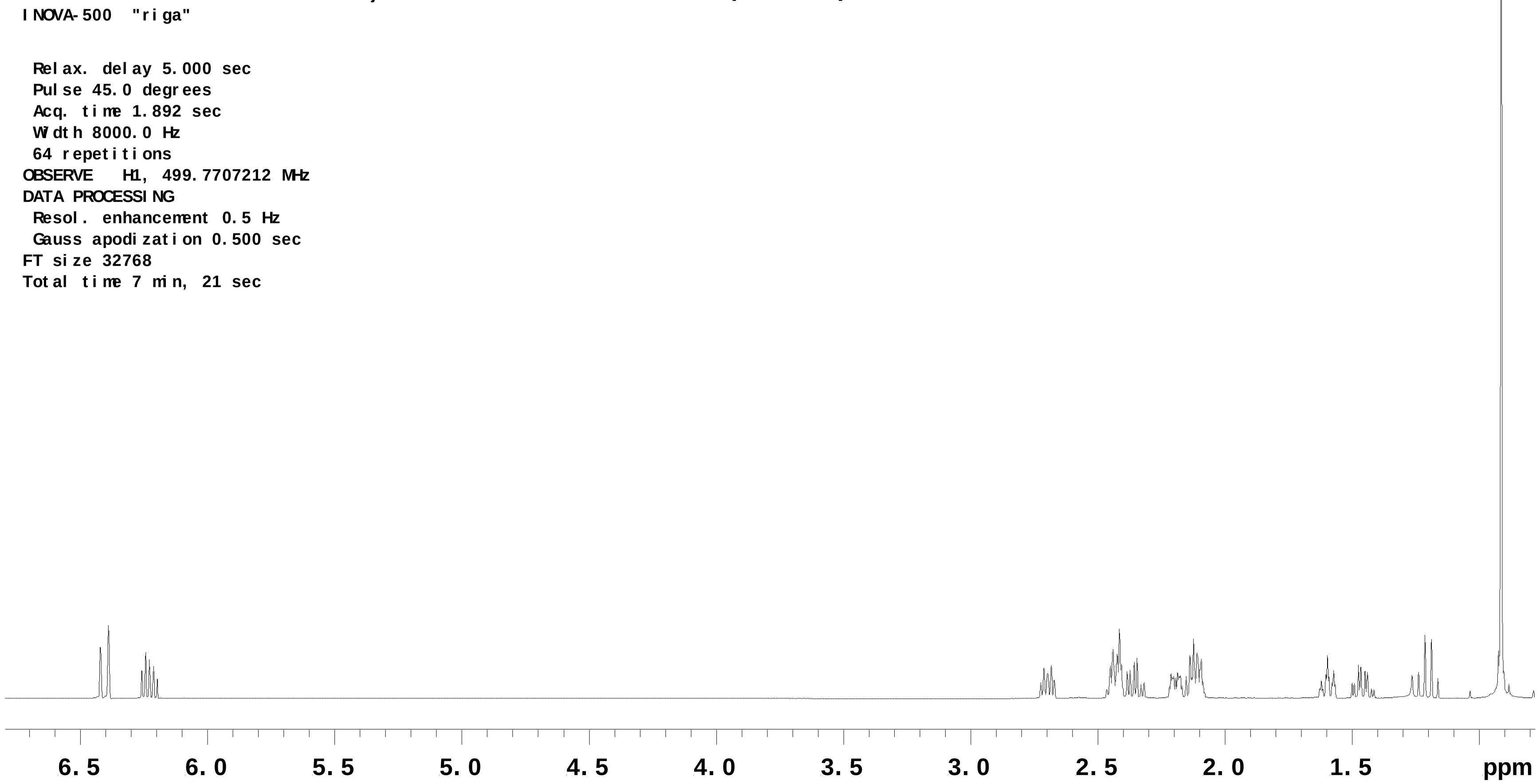

RRC-1205-IV-302-CNMR-CDCl3-majordiastereomer

Pulse Sequence: s2pul

Solvent: CDCl3
Temp. 25.0 C / 298.1 K
Operator: Raghu
File: RRC-1205-IV-302-CNMR-CDCl3-majordiastereomer
INOVA-500 "riga"

Relax. delay 3.000 sec
Pulse 45.0 degrees
Acq. time 1.300 sec
Width 30499.4 Hz
13270 repetitions
OBSERVE C13, 125.6674231 MHz
DECOUPLE H1, 499.7730084 MHz
Power 40 dB
continuously on
WALTZ-16 modulated
DATA PROCESSING
Line broadening 2.0 Hz
FT size 131072
Total time 23 hr, 56 min, 27 sec

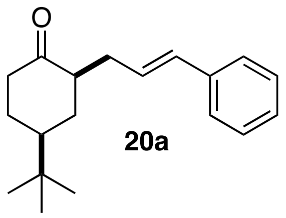

20a

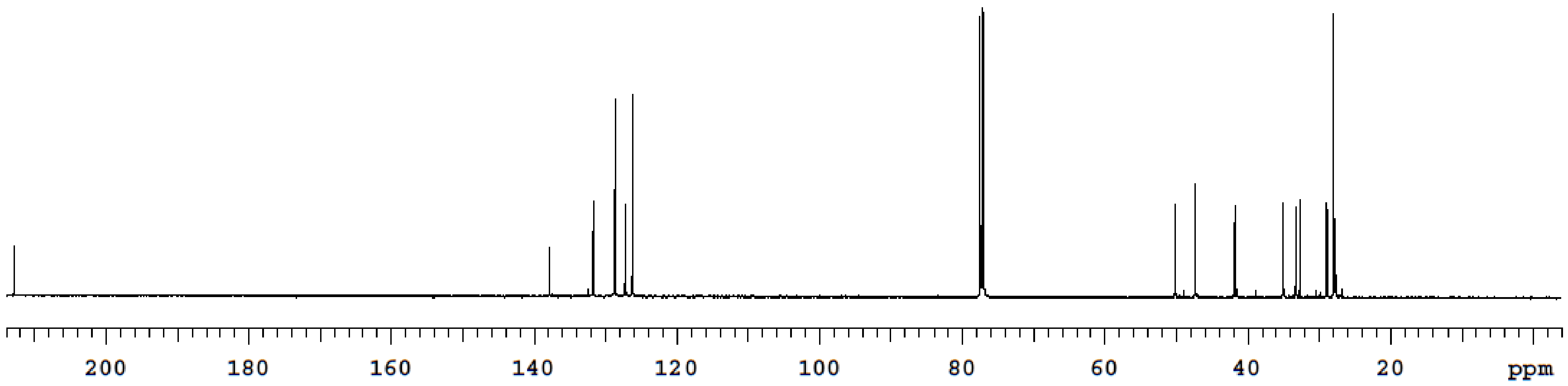

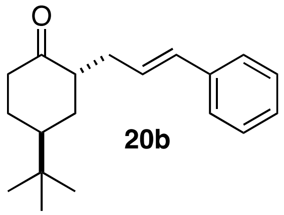

20b

RRC-1205-IV-302-HNMR-CDCl3-minordiastereomer

Pulse Sequence: s2pul

Solvent: CDCl3
Temp. 25.0 C / 298.1 K
Operator: Raghu
File: RRC-1205-IV-302-HNMR-CDCl3-minordiastereomer
INOVA-500 "riga"

Relax. delay 5.000 sec
Pulse 45.0 degrees
Acq. time 1.892 sec
Width 8000.0 Hz
64 repetitions
OBSERVE H1, 499.7707212 MHz
DATA PROCESSING
Resol. enhancement 0.5 Hz
Gauss apodization 0.500 sec
FT size 32768
Total time 7 min, 21 sec

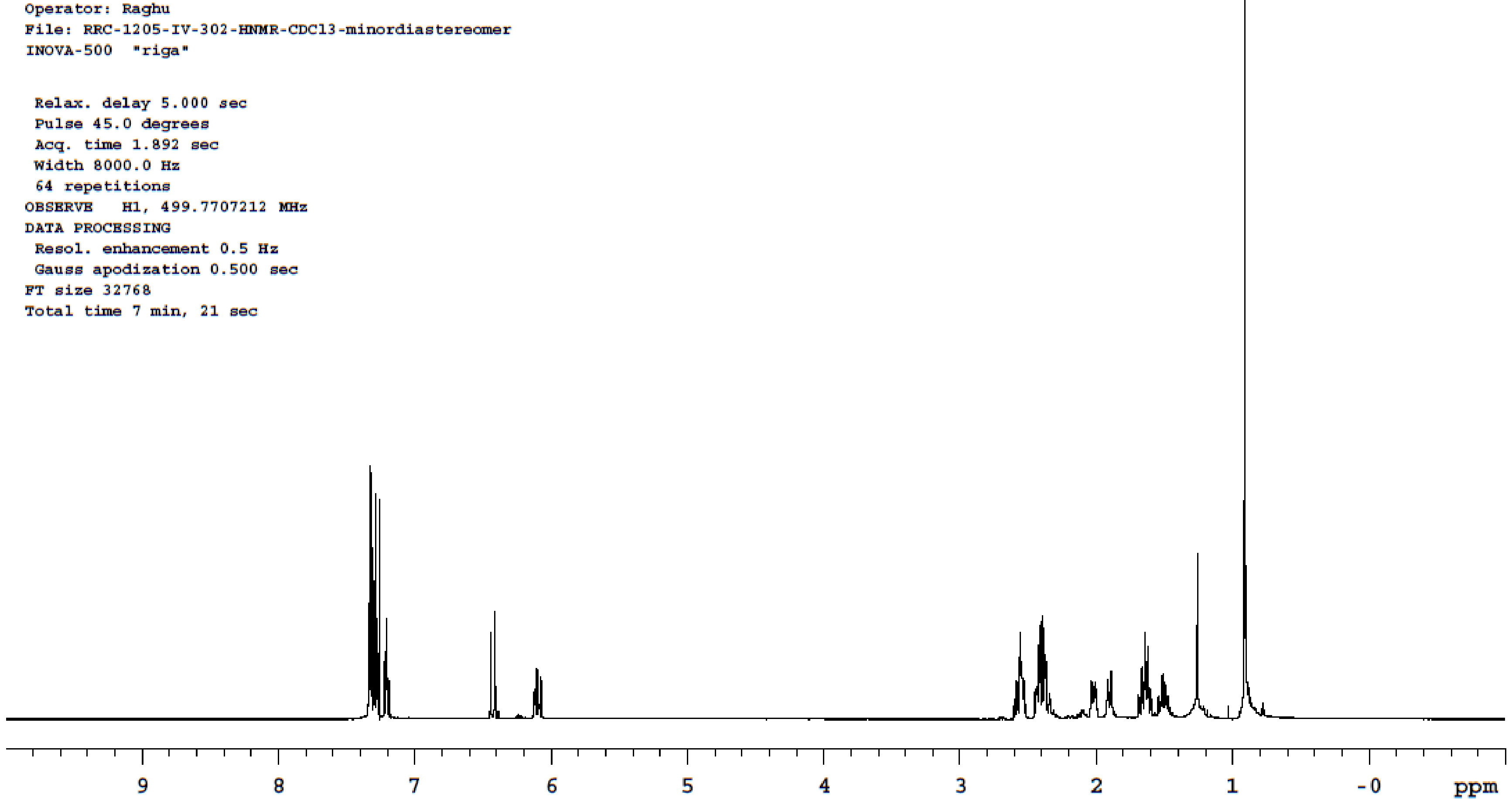

RRC-1205-IV-302-HNMR-CDCl3-minordiastereomer

Pulse Sequence: s2pul

Solvent: CDCl3
Temp. 25.0 C / 298.1 K
Operator: Raghu
File: RRC-1205-IV-302-HNMR-CDCl3-minordiastereomer
INOVA-500 "riga"

Relax. delay 5.000 sec
Pulse 45.0 degrees
Acq. time 1.892 sec
Width 8000.0 Hz
64 repetitions
OBSERVE H1, 499.7707212 MHz
DATA PROCESSING
Resol. enhancement 0.5 Hz
Gauss apodization 0.500 sec
FT size 32768
Total time 7 min, 21 sec

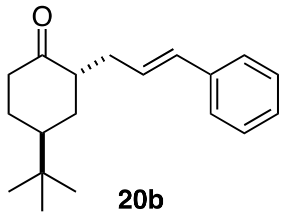

20b
Expanded spectrum

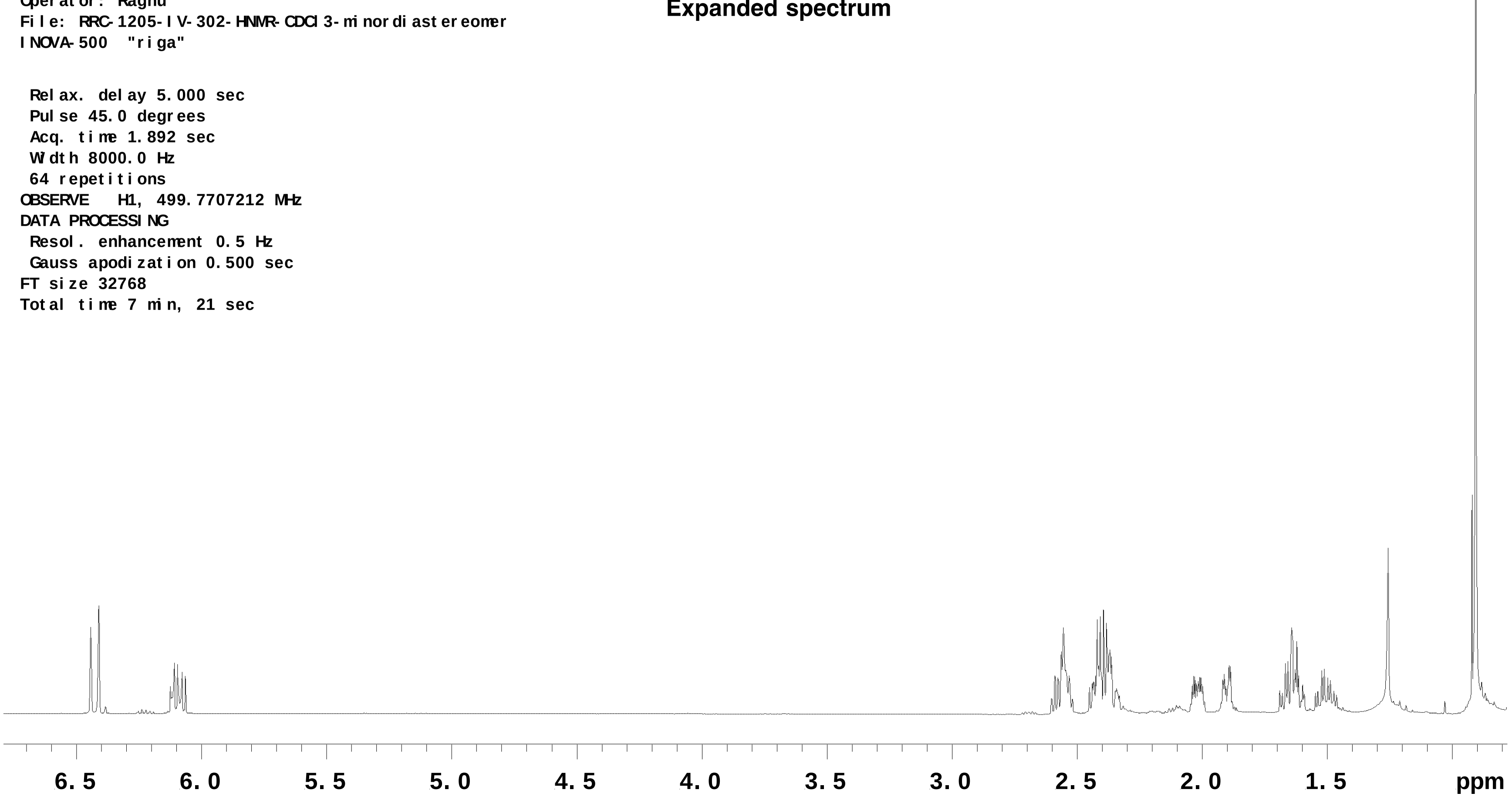

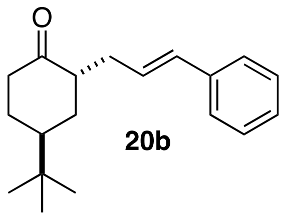

20b

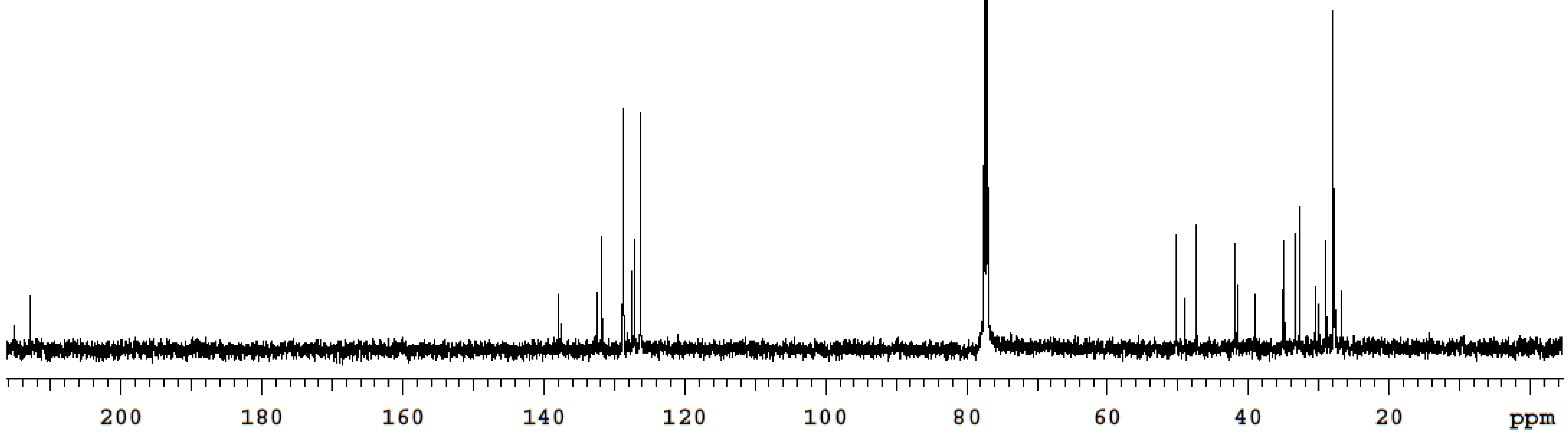
RRC-1205-IV-302-CNMR-minordiastereomer
Pulse Sequence: s2pul
Solvent: CDCl3
Temp. 25.0 C / 298.1 K
Operator: Raghu
File: RRC-1205-IV-302-CNMR-minordiastereomer
INOVA-500 "riga"
Relax. delay 3.000 sec
Pulse 45.0 degrees
Acq. time 1.300 sec
Width 30499.4 Hz
3330 repetitions
OBSERVE C13, 125.6674198 MHz
DECOUPLE H1, 499.7730084 MHz
Power 40 dB
continuously on
WALTZ-16 modulated
DATA PROCESSING
Line broadening 2.0 Hz
FT size 131072
Total time 23 hr, 56 min, 27 sec
200
180
160
140
120
100
80
60
40
20
ppm

500 MHz gCOSY NMR spectrum of **20a**

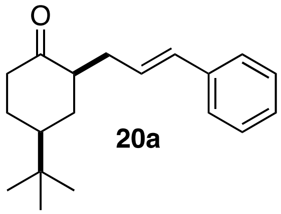

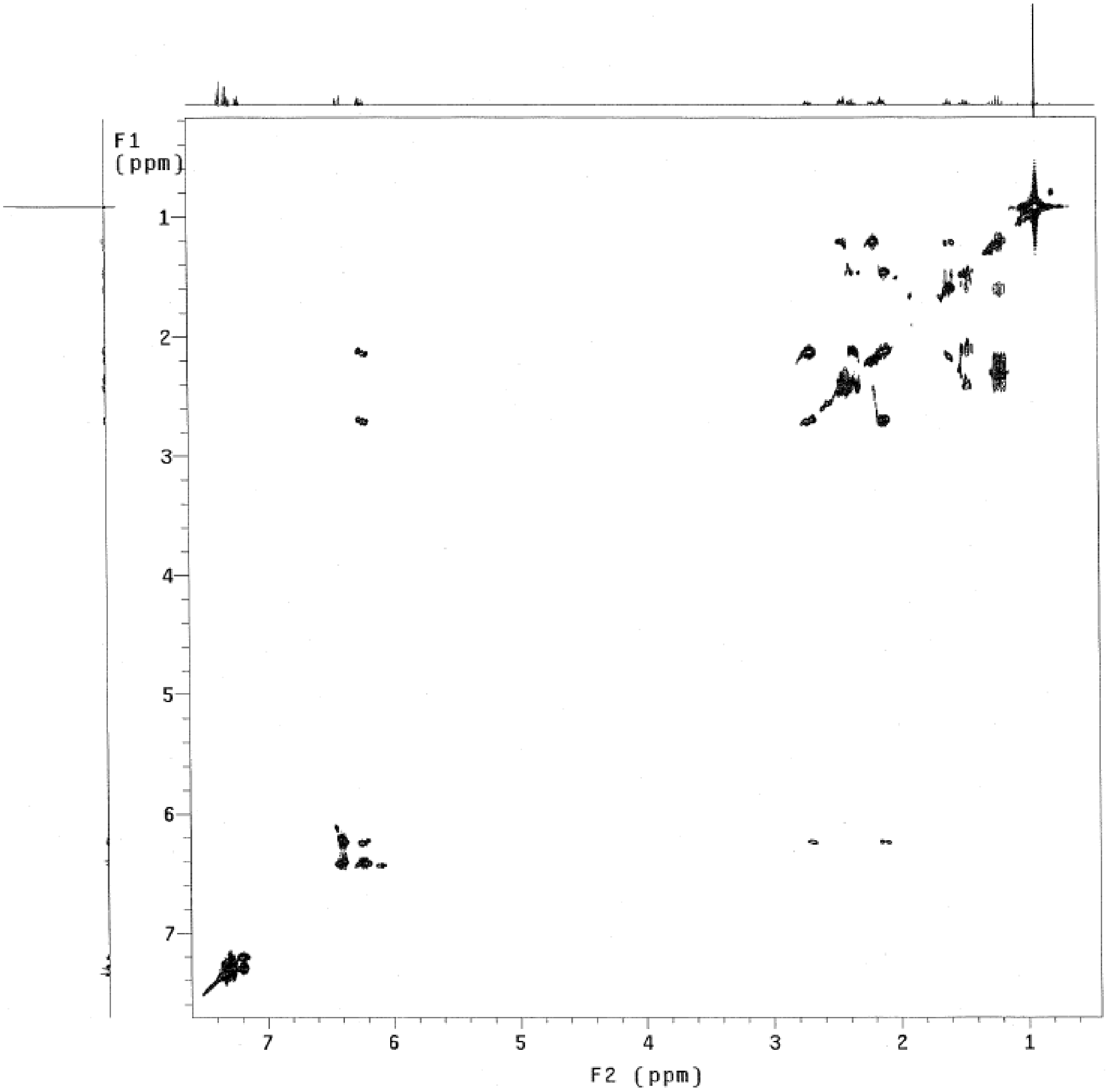

500 MHz NOESY NMR spectrum of **20a**

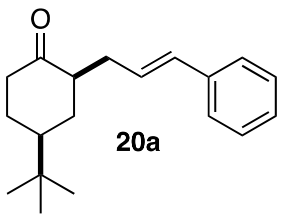

20a

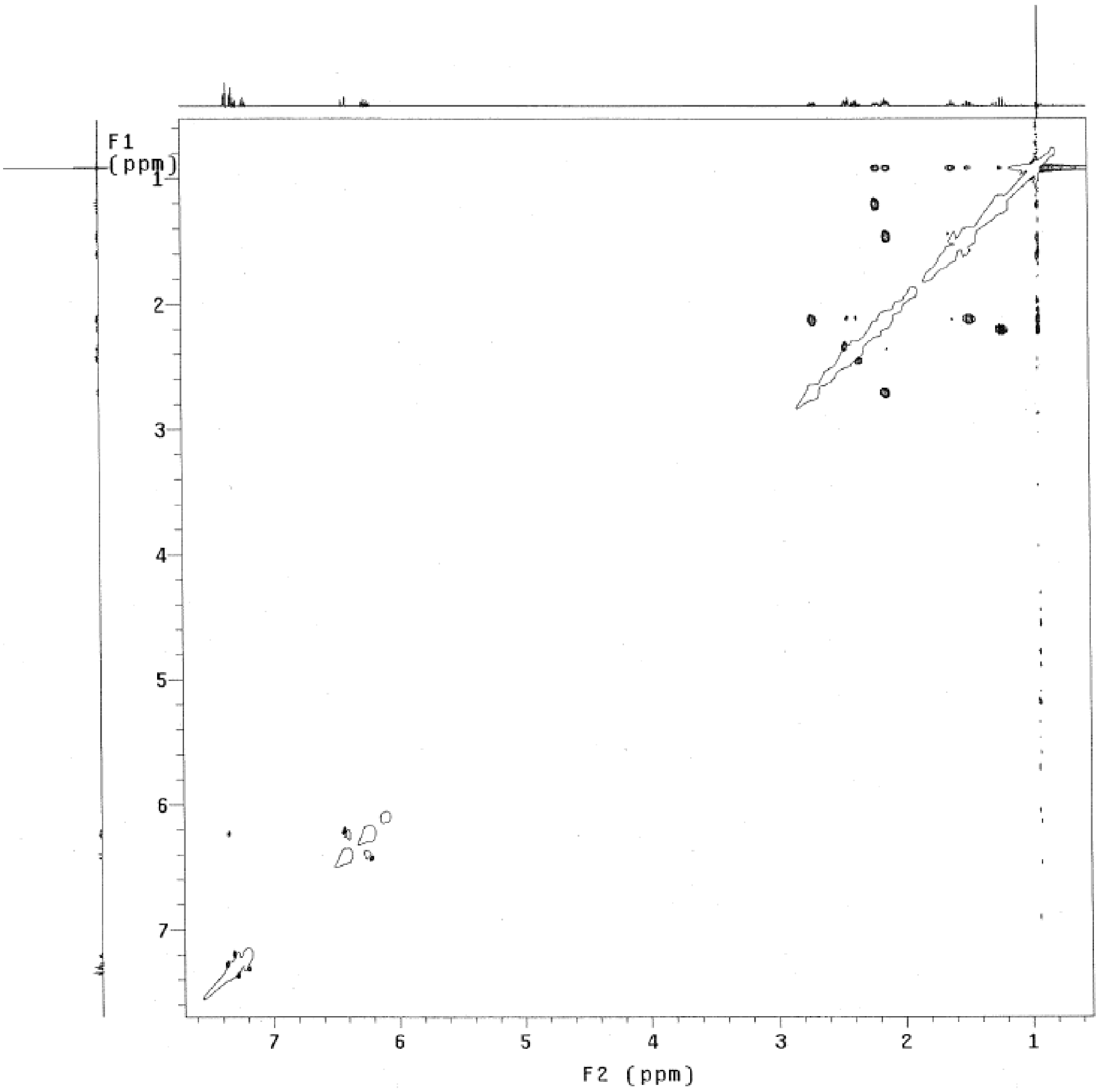

500 MHz gHMQC NMR spectrum of **20a**

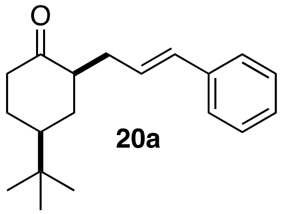

20a

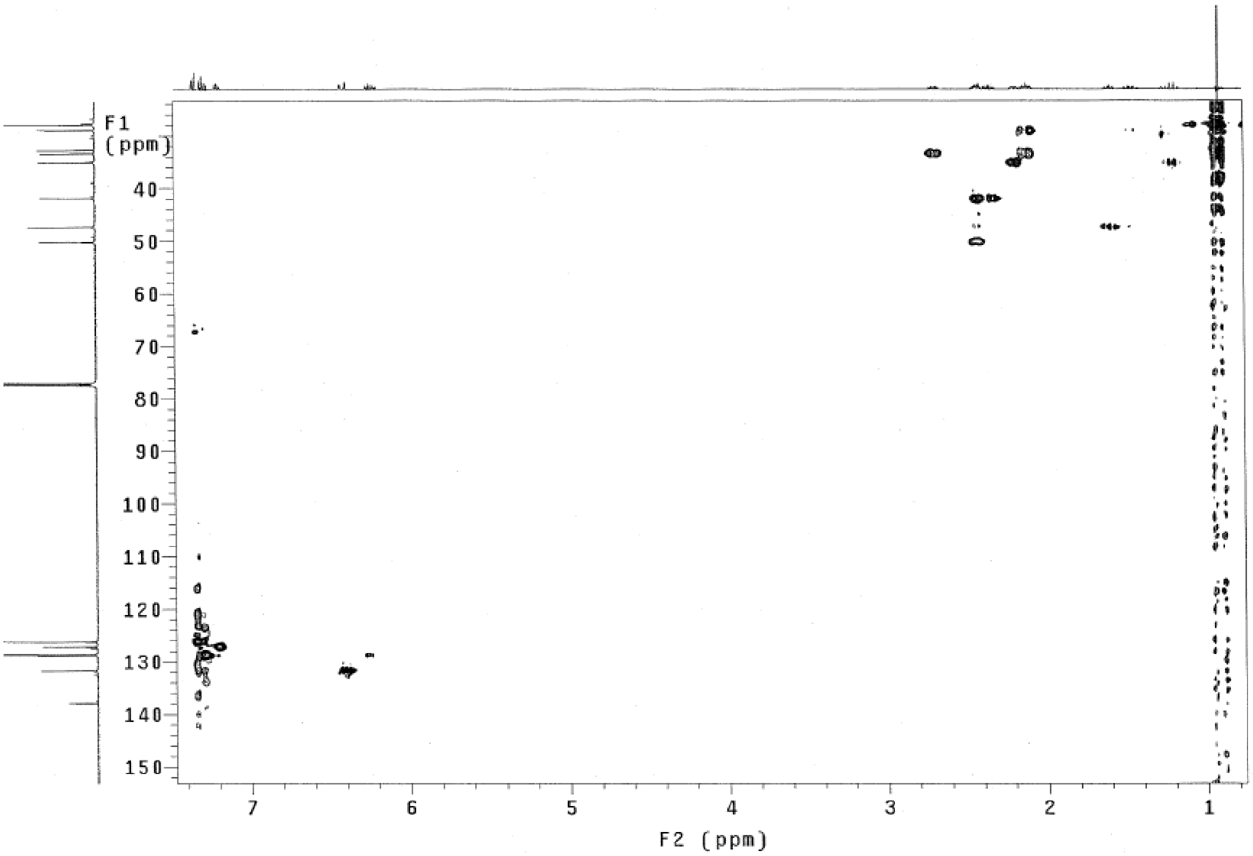

Supplement: File 2 — NMR spectra. [file Beilstein_J_Org_Chem-10-1919-s002.pdf]
